# Accuracy of Across-Environment Genome-Wide Prediction in Maize Nested Association Mapping Populations

**DOI:** 10.1534/g3.112.005066

**Published:** 2013-02-01

**Authors:** Zhigang Guo, Dominic M. Tucker, Daolong Wang, Christopher J. Basten, Elhan Ersoz, William H. Briggs, Jianwei Lu, Min Li, Gilles Gay

**Affiliations:** * Syngenta Biotechnology, Inc., Research Triangle Park, North Carolina 27709; † Syngenta, Inc., Clinton, Illinois 61727; ‡ Syngenta, Inc., Stanton, Minnesota 55018; § Syngenta Seeds B.V., 1601 BK Enkhuizen, The Netherlands

**Keywords:** best linear unbiased prediction, GenPred, genetic correlation, maize, ridge regression, shared data resources

## Abstract

Most of previous empirical studies with genome-wide prediction were focused on within-environment prediction based on a single-environment (SE) model. In this study, we evaluated accuracy improvements of across-environment prediction by using genetic and residual covariance across correlated environments. Predictions with a multienvironment (ME) model were evaluated for two corn polygenic leaf structure traits, leaf length and leaf width, based on within-population (WP) and across-population (AP) experiments using a large maize nested association mapping data set consisting of 25 populations of recombinant inbred-lines. To make our study more applicable to plant breeding, two cross-validation schemes were used by evaluating accuracies of (CV1) predicting unobserved phenotypes of untested lines and (CV2) predicting unobserved phenotypes of lines that have been evaluated in some environments but not others. We concluded that (1) genome-wide prediction provided greater prediction accuracies than traditional quantitative trait loci-based prediction in both WP and AP and provided more advantages over quantitative trait loci -based prediction for WP than for AP. (2) Prediction accuracy with ME was significantly greater than that attained by SE in CV1 and CV2, and gains with ME over SE were greater in CV2 than in CV1. These gains were also greater in WP than in AP in both CV1 and CV2. (3) Gains with ME over SE attributed to genetic correlation between environments, with little effect from residual correlation. Impacts of marker density on predictions also were investigated in this study.

Traditional quantitative trait loci (QTL)-based prediction (QP) in marker-assisted selection (MAS) is defined as a two-step process to predict breeding values (BVs) of untested lines for traits of interest based on QTL identified from a typical biparental plant breeding population. In the first step, QTL are identified based on phenotypic and genotypic data via various QTL mapping methods such as stepwise regression (Lande and Thompson 1990), interval mapping (Lander and Botstein 1989; Haley and Knott 1992), composite interval mapping (CIM) (Jansen 1993; Zeng 1993, 1994; Wang *et al.* 1999), and inclusive CIM (Li *et al.* 2007). In the next step, identified QTL passing a particular significance threshold are included in a single model, and the combined effects of all QTL alleles are estimated simultaneously by either maximum likelihood or multiple linear regression-based methodologies (Edwards and Johnson 1994; Meuwissen *et al.* 2001; Bernardo and Yu 2007). These effects are then used in MAS schemes to predict BVs of untested individuals based solely on genotypic data in a subsequent off-season nursery or green house selection (Lorenzana and Bernardo 2009).

The accuracy of QP is contingent on the numbers of QTL identified and their respective estimated effects. Although it is relatively easy to detect QTL with large phenotypic effects, it is difficult to identify QTL with intermediate or small effects (Lander and Botstein 1989; Xu 2003). This difficulty may be attributed to limited sample sizes of mapping populations and low heritabilities of target traits. Lack of sufficient replication and precision phenotyping also was indicated as possible reasons for low power of QTL mapping. Regardless, the power of QTL discovery determines the prediction accuracy during MAS and is responsible for the concomitant decrease in overall MAS efficiency. It is well known that QTL discovery in biparental mapping populations with a limited number of progeny (< 300) tends to overestimate individual QTL effects, regardless of statistical methodologies for effect assignment (the Beavis effect, see Beavis 1994). In severe situations, QTL effects cannot be estimated when genotypes of QTL are highly correlated or when population sizes are too small relative to the total number of putative QTL considered in the model. Therefore, from the perspective of statistical genetics, QP has focused on two key aspects: improving QTL mapping power and increasing the accuracy of QTL effects estimation. Although many statistical approaches have been developed for both purposes, improvement of QP accuracy for quantitative traits controlled by numerous small-effects QTL remains a current problem (Bernardo 2001; Heffner *et al.* 2009).

Simulation and empirical studies have shown that genome-wide prediction (GWP) (Meuwissen *et al.* 2001) provides improved accuracy in predicting BVs of untested lines over QP by the use of genomic marker trait associations in plants (Piyasatian *et al.* 2007; Lorenzana and Bernardo 2009; Zhong *et al.* 2009; Crossa *et al.* 2010; Heffner *et al.* 2011; Guo *et al.* 2012; Riedelsheimer *et al.* 2012; Zhao *et al.* 2012), animals (Lee *et al.* 2008; Legarra *et al.* 2008; Hayes *et al.* 2009a; Luan *et al.* 2009; Moser *et al.* 2009; Rolf *et al.* 2010; Mujibi *et al.* 2011; Wolc *et al.* 2011), and humans (Makowsky *et al.* 2011). With GWP, previously unidentified QTL with small effects in QP are captured in generated predictive models. This was shown to lead to significant increases of accuracies of derived predictions. In addition, to reduce the inflation of effect estimation of QTL, effects of each marker are treated as random draws from a common prior density in GWP. In practice, these assumptions of a prior probability distribution are leveraged to limit large fluctuations in marker effect estimates, inducing shrinkage estimates of marker effects. While ridge regression-best linear unbiased prediction (RR-BLUP), BayesA and BayesB approaches originally were developed to improve assessments of QTL effects for GWP strategies (Meuwissen *et al.* 2001), more attention has been directed toward extending and improving these methods to accommodate various types of study populations and to improve predictive ability (Lee *et al.* 2008; de los Campos *et al.* 2009, 2010; Meuwissen *et al.* 2009; Piepho 2009; Hayes *et al.* 2009b; Hayashi and Iwata 2010; Habier *et al.* 2011; Ober *et al.* 2011; González-Camacho *et al.* 2012).

However, the majority of the aforementioned studies apply to a univariate single-environment (SE) prediction model in which phenotypic records or means of a training sample and a validation sample are obtained from the same set of environments by using the same year and location effects, with no consideration of genetic and residual correlation across environments. Recent studies have illustrated that the use of genetic and residual covariance across correlated environments may better the accuracy of across-environment GWP using a multienvironment (ME) model (Calus and Veerkamp 2011; Burgueño *et al.* 2012). However, it is still not clear what the actual gain in prediction accuracy with ME over SE in typical biparental populations in plant breeding would be and what is the key factor contributing to the gains. Therefore, objectives in this study are threefold: (1) Evaluate the accuracies of across-environment predictions with QP and GWP based on within-population (WP) and across-population (AP) experiments with SE and ME models by using data from a large NAM data set consisting of 25 bi-parental half-sib populations; (2) assess influences of different genetic and residual covariance structures on the prediction accuracy of GWP with different ME models; and (3) conduct an assessment the effects of variation in marker density on GWP with the SE and ME models. To make our study more practical and applicable to plant breeding, two cross-validation schemes were employed to achieve the above objectives by evaluating the accuracies of (CV1) predicting unobserved phenotypes of untested lines and (CV2) predicting unobserved phenotypes of lines that have been evaluated in some environments but not others.

## Material and Methods

### NAM population

We retrieved phenotype and genotype data of 4131 recombinant inbred lines (RILs) from maize NAM populations derived from crosses between 25 genetically diverse inbreds and the maize elite parent B73 (Table 1) from the Panzea website (http://www.panzea.org). A total of 1106 single-nucleotide polymorphism (SNP) markers were used for genotyping each RIL, covering a genetic map of 1439 cM. The coding rules used for genotypic data were identical to those used in the within-environment prediction shown in Guo *et al.* (2012). To summarize, for individual populations, genotypes of each SNP of each progeny were coded −1 if both alleles were from a diverse parental line, 1 if from the common parent B73, and 0 otherwise. Based on coding rules, when several SNPs in complete linkage disequilibrium (LD) were available for a chromosomal region, only one SNP was selected. In each NAM population, selected SNPs varied from 785 to 895 whereas the genome coverage ranged from 1371 to 1397 cM. In the current study, we used phenotypic data of each RIL evaluated for leaf structure traits leaf length (LL) and leaf width (LW) from two locations, Aurora (AU), NY, and Champaign-Urbana (CU), IL, over 2 yr (2006 and 2007). A total of four environments were then defined as E1: AU at 2006; E2: CU at 2006; E3: AU at 2007; and E4: CU at 2007. Both LL and LW traits were found to have a genetic architecture affected by a large number of QTL of small effect (Tian *et al.* 2011). Based on phenotypic information from 2 yr and two locations, the broad sense heritability *H*^2^ on a mean basis (Supporting Information, File S1) ranged from 0.60 to 0.84 for the LL trait and from 0.60 to 0.82 for the LW trait (Table S1). Over 25 NAM populations, the average of *H*^2^ was 0.74 for both traits, reflecting a high precision of phenotyping efforts.

**Table 1:** Population information for the 25 NAM populations used for analysis in the current study

PopId	Crosses	Sample Size	Marker Number	Genomic Coverage, cM
1	B73×B97	167	805	1386
2	B73×CML103	173	813	1396
3	B73×CML228	180	889	1396
4	B73×CML247	165	828	1397
5	B73×CML277	156	820	1385
6	B73×CML322	173	838	1394
7	B73×CML333	165	827	1388
8	B73×CML52	163	834	1390
9	B73×CML69	176	840	1389
10	B73×Hp301	158	794	1387
11	B73×IIL4H	152	825	1389
12	B73×Ki11	170	822	1386
13	B73×Ki3	103	791	1397
14	B73×Ky21	182	817	1395
15	B73×M162W	163	827	1378
16	B73×M37W	172	788	1389
17	B73×Mo18W	178	818	1386
18	B73×MS71	171	771	1371
19	B73×NC350	176	827	1388
20	B73×NC358	156	809	1395
21	B73×Oh43	164	811	1389
22	B73×Oh7B	156	789	1396
23	B73×P39	161	828	1378
24	B73×Tx303	168	807	1394
25	B73×Tzi8	183	859	1381
Mean		165	819	1389

NAM, nested association mapping.

### Models for estimation of marker effects

Two methods, CIM and RR-BLUP, were used to estimate marker effects based on genotypic and phenotypic data in a training data set in CV1 with balanced phenotypic records and CV2 with unbalanced phenotypic records which were discussed in details in next section. Prediction accuracies with CIM and RR-BLUP were used to measure estimates with QP and GWP strategies, respectively. Two models, SE and ME, were applied in both methods based on training sets obtained from WP and AP. In total, there were four models developed for each method: AP-ME, AP-SE, WP-ME, and WP-SE. In the discussion of each method, we start with the most complex model, AP-ME, and then simplify to obtain others.

The AP-ME model with CIM was extended from multi-trait QTL mapping (Jiang and Zeng 1995) to accommodate multipopulation analysis, which can be written as

(1) Y=Xβ+Ws+Qα+ε

where **Y** = [**y**_1_, **y**_2_, Δ, **y***_n_*]^T^ is an *n* × *m* matrix of phenotypic data for *n* lines from *p* NAM populations and *m* environments, and **y**_1_, **y**_2_,Δ,**y***_n_* are vectors composed of observations across *m* environments; **X** is an *n* × (1 + *p*) incidence matrix for *n* lines from *p* populations representing population structure with the first column ones; **β** is a (1 + *p*) × *m* matrix of individual population effects with the first column representing the overall mean; **W** is an *n* × *f* matrix of genotypes of cofactor markers; **s** is an *f* × *m* matrix of cofactor marker effects with *f* the number of cofactor markers; **Q** is an *n* × 1 matrix of QTL genotypes; **α** is an 1 × *m* matrix of additive effects of a putative QTL at a tested position; and **ε =** [**ε**_1_, **ε**_2_, **Δ, ε**_n_]^T^ is a matrix of residuals **ε***_i_* (*i* = 1, 2, Δ, *n*), which is assumed to be correlated between environments and to follow a multivariate normal distribution with means zero and covariance matrix

$$\text{C}=\left[\begin{array}{cccc} e_{11}^{2} & e_{12}^{2} & \cdots & e_{1m}^{2}\\ e_{21}^{2} & e_{22}^{2} & \cdots & e_{2m}^{2}\\ \vdots & \vdots & \ddots & \vdots \\ e_{m1}^{2} & e_{m2}^{2} & \cdots & e_{mm}^{2} \end{array}\right].$$

In this model, the population effects **β**, cofactor effects **s**, and QTL additive effects **α** were treated as fixed effects and residual **ε** as random effects. QTL genotype **Q** was not observed and was replaced with its expected value obtained from the probability distribution of QTL genotypes conditional on the closest flanking markers (Haley and Knott 1992). Cofactor markers were selected by a modified stepwise selection procedure (Buckler *et al.* 2009). Extensions of this model to others were straightforward. For WP-ME, model (1) with population structure excluded from **X** became a single-population ME analysis (Jiang and Zeng 1995). AP-SE was similar to model (1) but was reduced to a univariate regression model for SE analysis, which was further simplified into a WP-SE model without population structure (Zeng 1993, 1994). To deal with unbalanced phenotypic records in a training data set, the modified CIM method developed by Guo and Nelson (2008) was applied to WP-ME and AP-ME in CV2 by iteratively imputing missing phenotypic data conditional on their posterior distributions.

On the basis of the aforementioned models, CIM was performed by scanning whole genomes with a fixed step size of 1 cM based on genotypic and phenotypic data in a training data set at each replicate of CV1 and CV2. A QTL was identified at the position in which the test statistic logarithm of odds (LODs) score assumed its maximum in the region under consideration with a LOD threshold (Utz *et al.* 2000). Cofactor selection in CIM requires a significance level that we estimated to be 0.0001 based on permutation tests for AP-SE and AP-ME (Buckler *et al.* 2009; Tian *et al.* 2011) and relaxed to 0.01 with WP-SE and WP-ME due to the decreased training sample size. Another key requirement was to determine LOD thresholds for identifying QTL. In the current study, LOD thresholds were estimated from 5000 permutation tests based on a genome-wide significance level 0.05 (Doerge and Churchill 1996). In WP-SE and AP-SE, phenotypic records for each RIL were randomized within each NAM population, whereas in WP-ME and AP-ME, permutation tests were performed by shuffling phenotypic records for multiple environments at once to preserve their correlation structure. Note that permutation tests were conducted for each model in CV1 and CV2 based on full data sets from each of the 25 NAM populations. Once QTL were identified using empirical LOD thresholds, effects of QTL were estimated by a multivariate multiple regression model with population structure.

The AP-ME model with RR-BLUP was an extension from the model proposed by Burgueño *et al.* (2012) as

$$\left[\begin{array}{c} {\text{y}}_{1}\\ {\text{y}}_{2}\\ \vdots \\ {\text{y}}_{i}\\ \vdots \\ {\text{y}}_{m} \end{array}\right]=\left[\begin{array}{cccccc} {\text{X}}_{1} & 0 & \cdots & 0 & \cdots & 0\\ 0 & {\text{X}}_{2} & \cdots & 0 & \cdots & 0\\ \vdots & \vdots & \ddots & \vdots & \vdots & \vdots \\ 0 & 0 & \cdots & {\text{X}}_{i} & \cdots & 0\\ \vdots & \vdots & \vdots & \vdots & \ddots & \vdots \\ 0 & 0 & \cdots & 0 & \cdots & {\text{X}}_{m} \end{array}\right]\left[\begin{array}{c} \beta_{1}\\ \beta_{2}\\ \vdots \\ \beta_{i}\\ \vdots \\ \beta_{m} \end{array}\right]+\left[\begin{array}{cccccc} {\text{Z}}_{1} & 0 & \cdots & 0 & \cdots & 0\\ 0 & {\text{Z}}_{2} & \cdots & 0 & \cdots & 0\\ \vdots & \vdots & \ddots & \vdots & \vdots & \vdots \\ 0 & 0 & \cdots & {\text{Z}}_{i} & \cdots & 0\\ \vdots & \vdots & \vdots & \vdots & \ddots & \vdots \\ 0 & 0 & \cdots & 0 & \cdots & {\text{Z}}_{m} \end{array}\right]\left[\begin{array}{c} \alpha_{1}\\ \alpha_{2}\\ \vdots \\ \alpha_{i}\\ \vdots \\ \alpha_{m} \end{array}\right]+\left[\begin{array}{c} \varepsilon_{1}\\ \varepsilon_{2}\\ \vdots \\ \varepsilon_{i}\\ \vdots \\ \varepsilon_{m} \end{array}\right]$$

where **y***_i_* (*i* = 1, 2,Δ, *m*) is a vector of phenotypic data for *n* lines from *p* NAM populations at the environment *i*; *m* is the total number of environments; **X***_i_* is an *n* × (1 + *p*) incidence matrix for *n* lines and *p* populations representing the population structure with the first column ones; **β***_i_* is a vector of individual population effects (including the overall mean); **Z***_i_* is an *n* × *k* matrix of marker genotypes with *k* the total number of markers; **α***_i_* is a vector of marker effects; and **ε***_i_* is a vector of residuals. The above model can be rewritten in reduced matrix form as

(2) Y=Xβ+Zα+ε

where **Y** = [**y**_1_^T^, **y**_2_^T^, Δ, **y***_m_*^T^]^T^; **β** = [**β**_1_^T^, **β**_2_^T^, Δ, **β***_m_*^T^]^T^; **α** = [**α**_1_^T^, **α**_2_^T^, Δ, **α***_m_*^T^]^T^; and **ε**^T^ = [**ε**_1_^T^, **ε**_2_^T^, Δ, **ε***_m_*^T^]^T^. In this model, the population effects **β** were treated as fixed effects. The marker effects **α** were treated as random effects following a multivariate normal distribution $N(0,{\text{G}}_{0}⊗{\text{I}}_{K})$with **G**_0_ the additive genetic covariance matrix between **α** and **α**′, **I***_K_* a *k* × *k* identity matrix, and ⊗ the Kronecker product of matrices. **G**_0_ can be defined as

$${\text{G}}_{0}=\left[\begin{array}{cccc} \sigma_{11}^{2} & \rho_{12}\sigma_{11}\sigma_{22} & \cdots & \rho_{1m}\sigma_{11}\sigma_{mm}\\ \rho_{21}\sigma_{22}\sigma_{11} & \sigma_{22}^{2} & \cdots & \rho_{2m}\sigma_{22}\sigma_{mm}\\ \vdots & \vdots & \ddots & \vdots \\ \rho_{m1}\sigma_{mm}\sigma_{11} & \rho_{m2}\sigma_{mm}\sigma_{22} & \cdots & \sigma_{mm}^{2} \end{array}\right]$$

where *σ_ii_*^2^ is the genetic variance of lines in environment *i* (*i* = 1, 2, Δ, *m*), *ρ_ij_* (*i* = 1, 2, Δ, *m*, and *j* = 1, 2, Δ, *m*) is the genetic correlation of lines between environment *i* and *j*; and *ρ_ij_* = *ρ_ji_*. The genetic covariance matrix of **Y** can be defined as

$$\text{G}={\text{G}}_{0}⊗\text{A}$$

where **A** is an *n* × *n* genetic relationship matrix, which can be calculated using genome-wide markers as

$$\text{A}=\text{M}{\text{M}}^{T}/2\sum_{i}^{k}p_{i}(1-p_{i})$$

where **M** is an *n* × *k* matrix of marker genotypes and *p_i_* is the frequency of an allele at locus *i* (*i* = 1, 2, Δ, *k*) (VanRaden 2008). Note that the matrix **M** is equivalent to **Z***_i_* (*i* = 1, 2, Δ, *m*) in the CV1 scheme. The residuals **ε** were considered as random effects following a multivariate normal distribution *N*(0, **R**) where **R** is a residual covariance matrix calculated by

$$\text{R}={\text{R}}_{0}⊗{\text{I}}_{N}$$

where **I***_N_* is an *n* × *n* identity matrix, and **R**_0_ is an *m* × *m* matrix of the residuals in different environments, written as

$${\text{R}}_{0}=\left[\begin{array}{cccc} e_{11}^{2} & r_{11}e_{11}e_{22} & \cdots & r_{1m}e_{11}e_{mm}\\ r_{21}e_{22}e_{11} & e_{22}^{2} & \cdots & r_{2m}e_{22}e_{mm}\\ \vdots & \vdots & \ddots & \vdots \\ r_{m1}e_{mm}e_{11} & r_{m2}e_{mm}e_{22} & \cdots & e_{mm}^{2} \end{array}\right]$$

where *e_ii_*^2^ is the residual variance in environment *i* (*i* = 1, 2, Δ, *m*), *r_ij_* (*i* = 1, 2, Δ, *m*; *j* = 1, 2, Δ, *m*) is the residual correlation of lines between environment *i* and *j*, and *r_ij_* = *r_ji_*. When model (2) was modified into AP-SE, it was reduced to a univariate model, and **G**_0_ and **R**_0_ became a special case of the multivariate model with diagonal entries denoting the genetic and residual variances, respectively. In WP-SE and WP-ME, the population structure **X** was reduced to the column vector of ones. These models were applied to estimate marker effects based on training data sets with balanced phenotypic records in CV1. For training sets with unbalanced phenotypic records in CV2, marker genotype matrix **Z***_i_* in environment *i* may not be equal to **Z***_j_* in environment *j*. The genetic covariance between line *l* and *q* across environment *i* and *j* in **G** was modified into **G***_ijlq_* = {**Z***_il_***Z***_jq_^T^*σ*_ij_*^2^/2∑*p_h_*(1 - *p_h_*)} where **Z***_il_* is the genome-wide marker genotype row vector for line *l* in environment *i*, **Z***_jq_* is the genome-wide marker genotype row vector for line *q* in environment *j*, *σ_ij_*^2^ is the entry of **G**_0_ at row *i* and column *j*, and *p_h_* is the frequency of an allele at locus *h* (*h* = 1, 2, Δ, *k*). The residual covariance was **R***_ijlq_* = {*e_ij_*^2^} with *e_ij_*^2^ the element at the *i*th row and the *j*th column of **R**_0_ if line *l* in environment *i* and line *q* in environment *j* are from the same genotype, and zero otherwise,

A key requirement in model (2) was to estimate the covariance matrix **G**_0_ and **R**_0._ In this study, **G**_0_ and **R**_0_ were estimated by the multivariate restricted maximum likelihood (REML) approach proposed by Vattikuti *et al.* (2012) based on model (2). Because estimates of variance components from multivariate REML were sensitive to initial values, estimates from univariate and bivariate REML were used as the initial values for the multivariate REML analysis. Although **G**_0_ and **R**_0_ were estimated in each NAM population for WP, they were estimated once by combining full data sets from all NAM populations for AP. Once estimation for **G**_0_ and **R**_0_ were done, environment-specific marker effects **α** along with the population effects **β** were estimated by solving Henderson’s mixed-model equations (Henderson 1984).

### Cross-validation

**Figure 1:**
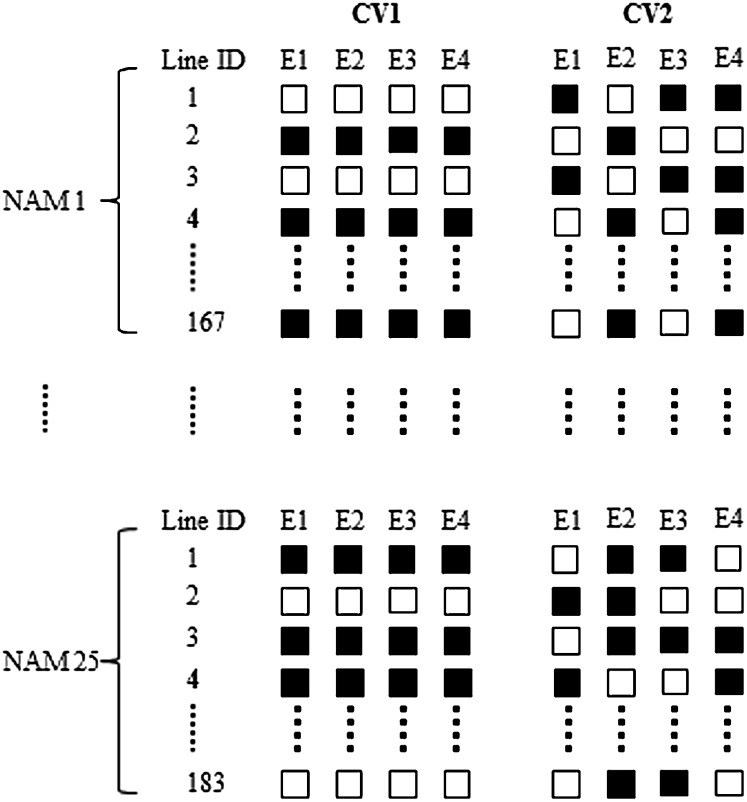
Example of one replicate of cross-validation in CV1 and CV2. White boxes represent observed phenotypic records, and black ones represent missing phenotypic records.

We used validation methods to estimate the prediction accuracy of each model described previoulsy. Cross-validation is a statistical technique of splitting data into training and validation sets by using the validation set to evaluate prediction ability of the trained model. Two cross-validation schemes, CV1 and CV2 (Figure 1), were considered in the study based on different prediction purposes relevant to practical breeding problems (Calus and Veerkamp 2011; Burgueño *et al.* 2012). In CV1, the goal was to predict unobserved phenotypes of untested lines or newly generated lines based solely on genotypes; and in CV2, missing phenotypes of lines in an environment were predicted with genotypes and phenotypes from other environments. A total of 100 replicates of cross-validation were performed for each scheme, and the validation results were averaged over each replicate.

In each replicate of CV1 (Figure 1), RILs in each population were randomly split into a training data set comprising 60% of the samples and a validation data set of the remainder (Legarra *et al.* 2008; Lorenzana and Bernardo 2009; Guo *et al.* 2012). Phenotypic records of each line in the validation set were set to missing for all environments. In CV2 (Figure 1), a specific proportion (40%) of phenotypic records for each environment independently were set to missing at random. Therefore, phenotypic information was balanced for training sets generated in CV1, and unbalanced in CV2. Lines lacking phenotypic data for all environments were dropped in CV2. Theoretically, the proportion of these lines in each NAM population was (0.40)^4^ = 2.56% across four environments. Excluding these lines had little effect on CV2 analysis.

In both CV1 and CV2 described above, training and validation sets were from a single population by the use of WP information. As an alternative practice, AP information can be used for prediction in the absence of WP information (Legarra *et al.* 2008; Zhao *et al.* 2012), mimicking a framework of using historical data to predict line performances of a cross. In CV1, the AP training set was generated by combining all RILs from the other 24 NAM populations. In CV2, the AP training set was generated with RILs of 25 NAM populations including the test population. In this case, each line in the training set contained unbalanced phenotypic records. It is worthy of mentioning that both WP and AP were used to predict performances of lines from the identical validation set in CV1 and CV2, respectively.

Although training was performed with WP and AP approaches in CV1 and CV2, validation was conducted within each NAM population for each environment. In each replicate, prediction accuracies were measured as the correlation coefficient between estimated BVs and observed phenotypes of lines in the validation set using

$${\hat{y}}_{i}=\hat{\mu }+\sum_{j=1}^{k}{\text{z}}_{ij}{\hat{\alpha }}_{j}$$

where *ŷ_i_* is the estimated estimated BV of individual *i* in the validation sample; $\hat{\mu }$ and ${\hat{\alpha }}_{j}$ are environment-specific overall mean and marker effects estimated from a training sample using the methods discussed in the last section; and **z***_ij_* is the genotype of marker *j* for line *i* in the validation set. Note that only QTL were used in the model with the QP strategy, while all the markers were included for the GWP strategy. The final reported prediction accuracy was in fact the mean of the 100 predictions generated across the replicate runs. Overall accuracies between various models were compared with a pairwise *t*-test (*α* = 0.05) based on identical validation sets within each NAM population. Gains in prediction accuracy with one model (*e.g.*, model *A*) over another one (*e.g.*, model *B*) were calculated using (*R_A_* – *R_B_*) / *R_B_*, where *R_A_* represents prediction accuracy with model *A* and *R_B_* prediction accuracy with model *B*.

### Impacts of genetic and residual covariance structure on GWP

Impacts of genetic and residual covariance on GWP with ME were assessed by comparing four different models: (1) structured genetic and residual covariance (SG-SR); (2) structured genetic and unstructured residual variance (SG-UR); (3) unstructured genetic and structured residual variance (UG-SR); and (4) unstructured genetic and residual covariance (UG-UR). SG-SR was the simplest model among them, in which each of the nondiagonal entries in **G**_0_ and **R**_0_ estimated above were set to zero, imposing independence on genetic and residual correlations. This model was equivalent to fitting each SE model separately with GWP. SG-UR imposed independence on genetic correlation by setting non-diagonal entries to zeros in **G**_0_, while UG-SR removed residual correlation by setting non-diagonal entries to zeros in **R**_0_. UG-UR was completely unstructured and estimated in the previous section. Influences of residual covariance were evaluated by comparing SG-SR with SG-UR, whereas impacts of genetic covariance were assessed by comparing UG-SR with SG-SR. Finally, effects of residual covariance given the genetic covariance were evaluated by comparing UG-UR with UG-SR.

### Effect of marker density on prediction accuracy of GWP

Effects of marker densities on GWP were tested by setting a conditional genetic distance criterion (*c*) between two flanking markers to ensure that each chromosome was evenly covered by a set of SNPs. For each NAM population, varying numbers of markers were obtained by selecting values of *c* (*c* = 1.6, 5, 10, 15, 20, 25, and 30 cM) without compromising the overall genomic coverage using the method described by Guo *et al.* (2012). This was done to observe the influence of using smaller marker sets than all available data from the original NAM study. All the identified polymorphic markers were included in the model at *c* = 1.6 cM, meaning that the mean marker distance of the entire marker set was ~1.6 cM. Note that a training sample proportion 0.6 was always used when assessing the effect of different marker densities to ensure different densities were applied to the same training and validation sets at each replicate of 100 cross-validations.

## Results

Accuracies of predictions for traits LL and LW are shown in Table 2. Prediction accuracy in each cell in this table was the average of prediction accuracies over 25 NAM populations (Table S7, Table S8, Table S9, Table S10, Table S11, Table S12, Table S13, Table S14, Table S15, Table S16, Table S17, Table S18, Table S19, Table S20, Table S21, and Table S22). As discussed previously, QTL for QP were identified using the empirical LOD thresholds estimated by permutation tests (Table S2). With GWP, the genetic and residual covariances obtained from multivariate REML for traits LL and LW (Table S3, Table S4, Table S5, and Table S6) were used to estimate environment-specific marker effects with RR-BLUP. We first compared GWP with QP in CV1 and CV2. Overall, GWP gave consistently greater accuracies than QP. Increase in accuracy with GWP over QP was statistically significant in 99% of predictions generated (Table S7, Table S8, Table S9, Table S10, Table S11, Table S12, Table S13, Table S14, Table S15, Table S16, Table S17, Table S18, Table S19, Table S20, Table S21, and Table S22). Gains in accuracies with GWP over QP were larger for WP than for AP were attributed to two factors: (1) GWP with WP gave greater accuracies than that with AP; and (2) QP with WP gave lower accuracies than that with AP due to a decreased number of QTL identified in WP. Although AP gave improved accuracies for QP over WP, it still yielded lower predictive power than GWP, which was also lower than the accuracies with GWP in WP.

**Table 2:** Prediction accuracy with QP and GWP using SE and ME models in CV1 and CV2 for traits LL and LW based on 25 NAM population

			LL				LW			
			SE		ME		SE		ME	
Scheme	Approach	Envi	QP*^a^*	GWP*^b^*	QP*^c^*	GWP*^d^*	QP*^a^*	GWP*^b^*	QP*^c^*	GWP*^d^*
CV1	WP	E1	0.20 (2.2)	0.38 (0.93)	0.17 (2.4, −0.10)	0.42 (1.40, 0.10)	0.19 (2.2)	0.40 (1.11)	0.20 (2.6, 0.06)	0.46 (1.29, 0.15)
		E2	0.19 (2.4)	0.41 (1.15)	0.17 (2.4, −0.12)	0.44 (1.60, 0.06)	0.24 (2.4)	0.46 (0.92)	0.23 (2.6, −0.05)	0.49 (1.18, 0.08)
		E3	0.17 (2.1)	0.38 (1.24)	0.16 (2.4, −0.04)	0.42 (1.57, 0.10)	0.15 (2.0)	0.37 (1.40)	0.18 (2.6, 0.18)	0.43 (1.37, 0.16)
		E4	0.16 (2.0)	0.37 (1.27)	0.16 (2.4, 0.00)	0.41 (1.64, 0.10)	0.27 (2.7)	0.48 (0.78)	0.24 (2.6, −0.10)	0.52 (1.15, 0.08)
		Mean	0.18 (2.2)	0.39 (1.00)	0.17 (2.4, −0.05)	0.42 (1.47, 0.08)	0.21 (2.3)	0.43 (1.05)	0.21 (2.6, 0.00)	0.48 (1.29, 0.12)
	AP	E1	0.27 (10.4)	0.31 (0.15)	0.29 (14.0, 0.09)	0.31 (0.07, 0.00)	0.32 (13.0)	0.36 (0.13)	0.34 (12.1, 0.08)	0.37 (0.09, 0.04)
		E2	0.28 (13.1)	0.32 (0.14)	0.29 (14.0, 0.01)	0.33 (0.14, 0.01)	0.37 (12.8)	0.42 (0.14)	0.39 (12.1, 0.06)	0.43 (0.10, 0.02)
		E3	0.24 (12.8)	0.29 (0.21)	0.25 (14.0, 0.04)	0.30 (0.20, 0.01)	0.30 (10.3)	0.34 (0.13)	0.32 (12.1, 0.09)	0.35 (0.09, 0.03)
		E4	0.25 (14.0)	0.30 (0.20)	0.23 (14.0, −0.07)	0.30 (0.30, 0.00)	0.38 (12.7)	0.42 (0.11)	0.39 (12.1, 0.03)	0.43 (0.10, 0.01)
		Mean	0.26 (12.6)	0.31 (0.19)	0.27 (14.0, 0.04)	0.32 (0.19, 0.03)	0.34 (12.2)	0.39 (0.15)	0.36 (12.1. 0.06)	0.40 (0.11, 0.03)
CV2	WP	E1	0.20 (2.1)	0.39 (0.98)	0.24 (1.8, 0.25)	0.53 (1.19, 0.38)	0.19 (2.2)	0.41 (1.14)	0.27 (2.0, 0.42)	0.55 (1.05, 0.36)
		E2	0.20 (2.3)	0.41 (1.10)	0.24 (1.8, 0.23)	0.56 (1.30, 0.35)	0.24 (2.5)	0.46 (0.92)	0.31 (2.0, 0.28)	0.59 (0.91, 0.27)
		E3	0.18 (2.0)	0.38 (1.17)	0.23 (1.8, 0.33)	0.52 (1.24, 0.37)	0.16 (2.0)	0.37 (1.36)	0.25 (2.0, 0.63)	0.52 (1.03, 0.40)
		E4	0.16 (2.0)	0.38 (1.38)	0.24 (1.8, 0.49)	0.53 (1.23, 0.39)	0.27 (2.7)	0.48 (0.78)	0.32 (2.0, 0.18)	0.61 (0.93, 0.28)
		Mean	0.19 (2.1)	0.39 (1.05)	0.24 (1.8, 0.26)	0.54 (1.30, 0.40)	0.22 (2.4)	0.43 (0.95)	0.29 (2.0, 0.32)	0.57 (0.96, 0.33)
	AP	E1	0.27 (8.0)	0.32 (0.19)	0.29 (9.5, 0.09)	0.36 (0.24, 0.12)	0.31 (8.5)	0.36 (0.16)	0.35 (11.1, 0.13)	0.40 (0.14, 0.10)
		E2	0.28 (9.8)	0.34 (0.21)	0.30 (9.5, 0.05)	0.37 (0.23, 0.10)	0.38 (10.6)	0.43 (0.13)	0.40 (11.1, 0.06)	0.46 (0.15, 0.07)
		E3	0.23 (7.8)	0.31 (0.35)	0.26 (9.5, 0.13)	0.35 (0.35, 0.12)	0.30 (7.3)	0.35 (0.17)	0.33 (11.1, 0.12)	0.38 (0.15, 0.09)
		E4	0.23 (7.4)	0.31 (0.35)	0.26 (9.5, 0.15)	0.35 (0.35, 0.12)	0.39 (11.0)	0.43 (0.10)	0.41 (11.1, 0.04)	0.46 (0.12, 0.07)
		Mean	0.25 (8.3)	0.32 (0.14)	0.28 (9.5, 0.12)	0.36 (0.29, 0.13)	0.35 (9.4)	0.39 (0.11)	0.37 (11.1, 0.06)	0.43 (0.16, 0.10)

QP, quantitative trait loci-based prediction; GWP, genome-wide prediction; SE, single environment; ME, multienvironment; LL, leaf length; LW, leaf width; NAM, nested association mapping; Envi, environment; WP, within population; AP, across population.

a In parentheses is the number of QTL identified by QP based on the SE model.

b In parentheses is the gain in prediction accuracy with GWP over QP based on the SE model.

c The first value in parentheses is the number of QTL identified by QP based on the ME model; and the second one the gain with ME over SE for QP.

d The first value in parentheses is the gain in accuracy with GWP over QP based on the ME model; and the second one is the gain in accuracy with ME over SE using GWP.

Afterward we compared prediction accuracies between the SE and ME models for GWP. The ME model provided greater prediction accuracies than the SM model in both CV1 and CV2. Increases in accuracies were significant in most cases (Table S7, Table S8, Table S9, Table S10, Table S11, Table S12, Table S13, Table S14, Table S15, Table S16, Table S17, Table S18, Table S19, Table S20, Table S21, and Table S22). For WP in CV1, across all populations and environments, average gains in accuracies with ME over SE were 8% and 12% for traits LL and LW, respectively. Gains attained a maximum of 48% in some populations in CV1 (Table S7). For WP in CV2, average increases with ME over SE reached 40% and 33% for LL and LW traits, respectively. For AP, gains with ME over SE were reduced to 3% in CV1 and 10–13% in CV2 for LL and LW traits. On average, across all populations, environments, and traits, gains with ME over SE were 10% and 37% for WP, and 3% and 11.5% for AP in CV1 and CV2, respectively. The aforementioned gains were observed based on the average genetic correlation of 0.77 and 0.87 and the average residual correlation of 0.31 and 0.39 for WP and AP, respectively (Table S3, Table S4, Table S5, and Table S6). With different training and validation sets, it may not be appropriate to evaluate performances of GWP between CV1 and CV2 on the same ground in this study. However, given similar accuracies with the SE model obtained from CV1 and CV2, comparison between them suggested, but did not prove, that gains with ME over SE were greater in CV2 than that in CV1 at WP and AP.

Prediction accuracies for GWP using ME with different genetic and residual variance structures are summarized in Table 3. Similar to Table 2, each of the accuracies shown in this table was the average of prediction accuracies over 25 NAM populations. Detailed results can be found in Table S23, Table S24, Table S25, Table S26, Table S27, Table S28, Table S29, Table S30, Table S31, Table S32, Table S33, Table S34, Table S35, Table S36, Table S37, and Table S38. In CV1 and CV2, SG-UR gave prediction accuracies comparable to or less than that attained by SG-SR, suggesting that addition of residual covariance did not benefit predictions, and may have worsened them. In contrast, prediction accuracies with UG-SR were greater than that with SG-SR, suggesting that modeling genetic covariance improved predictions with the ME model. Finally, UG-UR gave comparable prediction accuracies to UG-SR, suggesting that adding residual covariance had little effect on predictions in the presence of the genetic covariance.

**Table 3:** Prediction accuracies with ME GWP based on SG-SR, SG-UR, UG-SR, and UG-UR for traits LL and LW based on 25 NAM populations

			LL				LW			
Scheme	Approach	Envi	SG-SR	SG-UR*^a^*	UG-SR*^b^*	UG-UR*^c^*	SG-SR	SG-UR*^a^*	UG-SR*^b^*	UG-UR*^c^*
CV1	WP	E1	0.38	0.33 (–0.14)	0.42 (0.10)	0.42 (0.00)	0.40	0.37 (–0.09)	0.46 (0.14)	0.46 (0.00)
		E2	0.41	0.36 (–0.12)	0.45 (0.08)	0.44 (–0.02)	0.46	0.42 (–0.08)	0.50 (0.09)	0.49 (–0.01)
		E3	0.38	0.32 (–0.16)	0.43 (0.12)	0.42 (–0.02)	0.37	0.33 (–0.11)	0.43 (0.16)	0.43 (0.00)
		E4	0.37	0.31 (–0.16)	0.42 (0.13)	0.41 (–0.02)	0.48	0.45 (–0.07)	0.52 (0.08)	0.52 (0.00)
		Mean	0.39	0.33 (–0.18)	0.43 (0.10)	0.42 (–0.02)	0.43	0.39 (–0.09)	0.48 (0.12)	0.48 (0.00)
	AP	E1	0.31	0.30 (–0.03)	0.31 (0.02)	0.31 (0.00)	0.36	0.35 (–0.02)	0.37 (0.04)	0.37 (0.00)
		E2	0.32	0.32 (–0.02)	0.33 (0.01)	0.33 (0.00)	0.42	0.41 (–0.01)	0.42 (0.01)	0.43 (0.01)
		E3	0.29	0.29 (0.00)	0.30 (0.01)	0.30 (0.00)	0.34	0.33 (–0.02)	0.35 (0.03)	0.35 (0.00)
		E4	0.30	0.29 (–0.03)	0.30 (0.00)	0.30 (0.00)	0.42	0.41 (–0.02)	0.43 (0.01)	0.43 (0.00)
		Mean	0.31	0.30 (–0.03)	0.31 (0.00)	0.31 (0.00)	0.39	0.38 (–0.03)	0.39 (0.00)	0.40 (0.01)
CV2	WP	E1	0.39	0.37 (–0.04)	0.54 (0.39)	0.53 (–0.01)	0.41	0.39 (–0.02)	0.55 (0.36)	0.55 (0.00)
		E2	0.41	0.40 (–0.03)	0.56 (0.35)	0.56 (0.00)	0.46	0.45 (–0.02)	0.59 (0.27)	0.59 (0.00)
		E3	0.38	0.36 (–0.06)	0.53 (0.38)	0.52 (–0.01)	0.37	0.36 (–0.03)	0.52 (0.40)	0.52 (0.00)
		E4	0.38	0.36 (–0.05)	0.54 (0.43)	0.53 (–0.01)	0.48	0.47 (–0.02)	0.61 (0.28)	0.61 (0.00)
		Mean	0.39	0.37 (–0.05)	0.54 (0.38)	0.54 (0.00)	0.43	0.42 (–0.02)	0.57 (0.33)	0.57 (0.00)
	AP	E1	0.32	0.32 (0.00)	0.36 (0.12)	0.36 (0.00)	0.36	0.36 (0.00)	0.40 (0.10)	0.40 (0.00)
		E2	0.34	0.34 (0.00)	0.37 (0.10)	0.37 (0.00)	0.43	0.42 (–0.01)	0.46 (0.07)	0.46 (0.00)
		E3	0.31	0.31 (0.00)	0.35 (0.12)	0.35 (0.00)	0.35	0.35 (0.00)	0.38 (0.09)	0.38 (0.00)
		E4	0.31	0.31 (0.00)	0.35 (0.12)	0.35 (0.00)	0.43	0.43 (0.00)	0.46 (0.07)	0.46 (0.00)
		Mean	0.32	0.32 (0.00)	0.36 (0.13)	0.36 (0.00)	0.39	0.39 (0.00)	0.43 (0.10)	0.43 (0.00)

ME, multi-environment; GWP, genome-wide prediction; SG-SR, structured genetic and residual covariance; SG-UR, Structured genetic and unstructured residual covariance; UG-SR, Unstructured genetic and structured residual covariance; UG-UR, Unstructured genetic and residual covariance; LL, leaf length; LW, leaf width; NAM, nested association mapping; Envi, environment; WP, within population; AP, across population.

a In parentheses is the gain in prediction accuracy with SG-UR over SG-SR.

b In parentheses is the gain in accuracy with UG-SR over SG-SR.

c In parentheses is the gain in accuracy with UG-UR over UG-SR.

**Figure 2:**
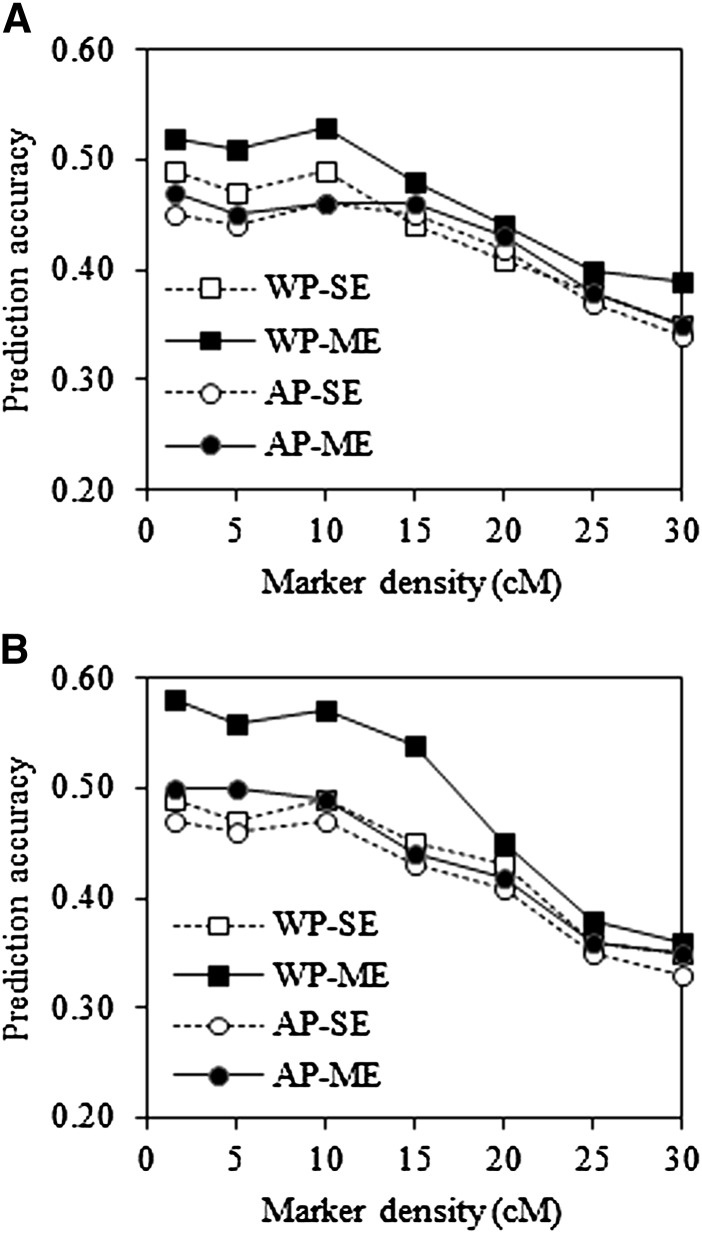
Prediction accuracy in environment E1 for the trait LL using SE and ME models in GWP with different levels of marker densities. The training sample proportion is 0.6 in NAM population B73×CML322. (A) CV1; (B) CV2.

Marker densities of 1.6, 5, 10, 15, 20, 25, and 30 cM were used corresponding to different numbers of markers in each training sample via methods described in the previous section. As expected, prediction accuracies for GWP with SE and ME models improved with increasing marker density in the example using the NAM population B73×CML322 (Figure 2). These improvements tended to diminish to zero when marker density exceeded a threshold of 10 cM, suggesting that the density selected based on an interval size of 10 cM was sufficient to capture LD between QTL and markers in these bi-parental segregating populations, similar to reports by Guo *et al.* (2012).

## Discussion

GWP provides a significant predictive advantage over QP in WP and AP, with increased gains in WP compared with AP. For almost all cases in WP, GWP shows significantly greater accuracy than QP in each of the NAM populations. This conclusion is consistent with the results from previous studies (Lorenzana and Bernardo 2009; Guo *et al.* 2012; Zhao *et al.* 2012). More interestingly, when training sets are changed from WP to AP, estimations with GWP are reduced, similar to previous reports in mice (Legarra *et al.* 2008) and maize (Zhao *et al.* 2012). The loss of accuracy from WP to AP for GWP may attribute to the differences of genetic backgrounds between training and validation populations. The differences may also affect QP and could reduce prediction accuracy from WP to AP for QP. However, in this study, we found a gain in accuracy with AP over WP. This could be explained by the different genetic basis used for prediction between GWP and QP. Although GWP relies on genetic relationship derived from genome-wide markers, QP uses a small subset of markers and focuses more on LD between these markers and QTL. As a result, QP may be less affected by different genetic backgrounds between training and validation sets than GWP. The potential loss caused by the different genetic backgrounds in QP could be compensated by the gain obtained from AP by improved QTL mapping power by utilizing multiple populations (Yi and Xu 2002; Blanc *et al.* 2006; Buckler *et al.* 2009; Tian *et al.* 2011). However, even with increased prediction accuracy with AP, QP still cannot achieve the high prediction accuracies attained by GWP with WP. This finding suggests, in practical breeding, to achieve high prediction accuracies, even with a high cost, it is still worthwhile to phenotype a proportion of lines from a breeding population to predict performances of genotypes generated from the subsequent intercross or backcross generations (Bernardo and Yu 2007).

The use of across-environment information improves prediction with GWP. Two main results were obtained from this study. First, modeling covariances between correlated environments with the ME model gives better predictions compared with SE in both the CV1 and CV2 schemes. At an average genetic correlation of 0.77, gains of 10% in CV1 and 37% in CV2 were observed for WP, greater than that of 3% in CV1 and 11.5% in CV2 for AP at a correlation of 0.87. Although the superiority of ME over SE in CV2 can be explained by borrowing information from the same lines across environments (Burgueño *et al.* 2012), gains in CV1 may likely attribute to more accurate estimates of environment-specific marker effects by utilizing genetic correlation. Gains in CV1 and CV2 also were reported based on a simulation study in animal breeding with the multitrait model, similar to the ME model tested in this study. This gave increases in accuracies of 0.03 to 0.14 over the single-trait prediction model that is similar to the SE model at genetic correlation levels of 0.25 and 0.75 (Calus and Veerkamp 2011). Furthermore, we found that the gain in CV2 with ME over SE is greater than that in CV1. This conclusion is also consistent with previously reported results (Calus and Veerkamp 2011; Burgueño *et al.* 2012). Second, by modeling and comparing different genetic and residual covariance structures, we found that gains with ME over SE are attributed to genetic covariance in CV1 and CV2, with little or negative contribution from the residual covariance. This finding indicates that accurately estimating the genetic covariance structure between correlated environments is critical to improve prediction accuracy with ME models. Although these results were obtained with relatively simple covariance structures from only four environments, more studies need to be conducted to confirm if these gains reflect a general superiority of the ME model with a large number of environments.

Impacts of marker density also were evaluated for GWP with the SE and ME models. Overall, our conclusions were consistent with previous work from within-environment predictions (Lorenzana and Bernardo 2009; Heffner *et al.* 2011; Guo *et al.* 2012; Zhao *et al.* 2012). Results indicate that a marker density of 10 cM, approximately corresponding to 150 markers, should be used when designing a predictive breeding strategy with GWP in a biparental breeding population. Although this result is consistent with findings in the previous studies in plant breeding (Lorenzana and Bernardo 2009; Guo *et al.* 2012), it has to be kept in mind that conclusions were drawn based on the typical biparental segregating populations with extensive LD caused by limited recombination. In the context of animal and human GWP where the populations studied are generally composed of complicated pedigreed individuals, a greater marker density is required to capture the small LD caused by accumulated historical recombinations. It was reported that at least 80,000 SNPs in human (Makowsky *et al.* 2011) and 5000 to 7500 SNPs in animals (Vazquez *et al.* 2010) are needed to reach a high prediction accuracy with GWP. Therefore, marker densities may be determined mainly by LD structure in different types of breeding populations. Also the goals and resources of a breeding project may affect decisions.

In practical plant breeding, GWP needs to be integrated into the appropriate genome-wide selection schemes where several cycles of intercrossing may be required to increase genetic gains (Bernardo and Yu 2007; Jannink *et al.* 2010). The CV1 scheme discussed in this study will mainly serve this purpose, and BVs of newly generated genotypes will be predicted and used for selection. Though there is no need for QTL identification in GWP, this does not suggest that the QTL ideotype construction strategy deployed in marker-assisted recurrent selection scheme cannot be implemented via GWP (Nakaya and Isobe 2012). At its simplest, the construction of QTL ideotypes may be accounted for by BVs obtained from GWP. For example, these BVs can be used in the QTL introgression process to efficiently pyramid 3 to 30 QTL as demonstrated by Bernardo (2009). In contrast, the CV2 scheme investigated in the current paper may be used to predict missing phenotypes caused by random missingness for a same set of genotypes evaluated across multiple environments. Overall, when the focus of a complex trait study is migrated from traditional line performances to allelic effect evaluation (Heffner *et al.* 2011), prediction of BVs with GWP may be further improved in CV1 and CV2 schemes. To maximize genetic gains in varied breeding programs per unit time and cost, it will be critical to design different genome-wide selection strategies to fully take advantage of the high accuracy of BVs at key stages of marker-assisted breeding.

## Supplementary Material

Supporting Information
