# Supplementary material for: Accuracy of Across-Environment Genome-Wide Prediction in Maize Nested Association Mapping Populations
Source: G3 (Bethesda). 2013 Feb 1;3(2):263–72. doi: 10.1534/g3.112.005066 (PMC3564986; doi:10.1534/g3.112.005066)
Supplement: Supporting Information [file supp_3_2_263__index.html]

Supporting Information 

# Accuracy of Across-Environment Genome-Wide Prediction in Maize Nested Association Mapping Populations

## Supporting Information for Guo *et al.*, 2013

**Files in this Data Supplement:**

- Supporting Information - File S1 and Tables S1-S38 (PDF, 576 KB)
- File S1 - Method for estimation of variance components and broad sense heritability (H2) (PDF, 72 KB)
- Table S1 - Variance components for traits LL and LW based on 25 NAM populations (PDF, 68 KB)
- Table S2 - Estimates of empirical LOD thresholds of CIM based on permutation tests (PDF, 59 KB)
- Table S3 - Estimates of genetic and residual covariance and correlation (in the lower diagonal) based on individual NAM populations 1-10 (PDF, 79 KB)
- Table S4 - Estimates of genetic and residual covariances and correlations (in the lower diagonal) based on individual NAM populations 11-20 (PDF, 79 KB)
- Table S5 - Estimates of genetic and residual covariances and correlations (in the lower diagonal) based on individual NAM populations 21-25 (PDF, 69 KB)
- Table S6 - Estimates of genetic and residual covariance and correlation (in the lower diagonal) based on a full data set of 25 NAM populations (PDF, 61 KB)
- Table S7 - Accuracy of WP prediction for environment E1 with QP and GWP in CV1 (PDF, 68 KB)
- Table S8 - Accuracy of WP prediction for environment E2 with QP and GWP in CV1 (PDF, 69 KB)
- Table S9 - Accuracy of WP prediction for environment E3 with QP and GWP in CV1 (PDF, 68 KB)
- Table S10 - Accuracy of WP prediction for environment E4 with QP and GWP in CV1 (PDF, 68 KB)
- Table S11 - Accuracy of AP prediction for environment E1 with QP and GWP in CV1 (PDF, 69 KB)
- Table S12 - Accuracy of AP prediction for environment E2 with QP and GWP in CV1 (PDF, 69 KB)
- Table S13 - Accuracy of AP prediction for environment E3 with QP and GWP in CV1 (PDF, 69 KB)
- Table S14 - Accuracy of AP prediction for environment E4 with QP and GWP in CV1 (PDF, 69 KB)
- Table S15 - Accuracy of WP prediction for environment E1 with QP and GWP in CV2 (PDF, 68 KB)
- Table S16 - Accuracy of WP prediction for environment E2 with QP and GWP in CV2 (PDF, 68 KB)
- Table S17 - Accuracy of WP prediction for environment E3 with QP and GWP in CV2 (PDF, 68 KB)
- Table S18 - Accuracy of WP prediction for environment E4 with QP and GWP in CV2 (PDF, 68 KB)
- Table S19 - Accuracy of AP prediction for environment E1 with QP and GWP in CV2 (PDF, 68 KB)
- Table S20 - Accuracy of AP prediction for environment E2 with QP and GWP in CV2 (PDF, 68 KB)
- Table S21 - Accuracy of AP prediction for environment E3 with QP and GWP in CV2 (PDF, 68 KB)
- Table S22 - Accuracy of AP prediction for environment E4 with QP and GWP in CV2 (PDF, 68 KB)
- Table S23 - Accuracy of WP prediction for environment E1 with four ME GWP models in CV1 (PDF, 67 KB)
- Table S24 - Accuracy of WP prediction for environment E2 with four ME GWP models in CV1 (PDF, 66 KB)
- Table S25 - Accuracy of WP prediction for environment E3 with four ME GWP models in CV1 (PDF, 66 KB)
- Table S26 - Accuracy of WP prediction for environment E4 with four ME GWP models in CV1 (PDF, 66 KB)
- Table S27 - Accuracy of AP prediction for environment E1 with four ME GWP models in CV1 (PDF, 66 KB)
- Table S28 - Accuracy of AP prediction for environment E2 with four ME GWP models in CV1 (PDF, 66 KB)
- Table S29 - Accuracy of AP prediction for environment E3 with four ME GWP models in CV1 (PDF, 66 KB)
- Table S30 - Accuracy of AP prediction for environment E4 with four ME GWP models in CV1 (PDF, 66 KB)
- Table S31 - Accuracy of WP prediction for environment E1 with four ME GWP models in CV2 (PDF, 66 KB)
- Table S32 - Accuracy of WP prediction for environment E2 with four ME GWP models in CV2 (PDF, 66 KB)
- Table S33 - Accuracy of WP prediction for environment E3 with four ME GWP models in CV2 (PDF, 66 KB)
- Table S34 - Accuracy of WP prediction for environment E4 with four ME GWP models in CV2 (PDF, 66 KB)
- Table S35 - Accuracy of AP prediction for environment E1 with four ME GWP models in CV2 (PDF, 66 KB)
- Table S36 - Accuracy of AP prediction for environment E2 with four ME GWP models in CV2 (PDF, 66 KB)
- Table S37 - Accuracy of AP prediction for environment E3 with four ME GWP models in CV2 (PDF, 66 KB)
- Table S38 - Accuracy of AP prediction for environment E4 with four ME GWP models in CV2 (PDF, 66 KB)
