# Supplementary material for: Accuracy of Across-Environment Genome-Wide Prediction in Maize Nested Association Mapping Populations
Source: G3 (Bethesda). 2013 Feb 1;3(2):263–72. doi: 10.1534/g3.112.005066 (PMC3564986; doi:10.1534/g3.112.005066)
Supplement: Supporting Information [file supp_3.2.263_005066SI.pdf]

G3: Genes | Genomes | Genetics

Accuracy of Across-environment Genome-wide Prediction in Maize Nested Association Mapping Populations

Zhigang Guo*[1], Dominic M. Tucker[§], Daolong Wang*, Christopher J. Basten*, Elhan Ersoz**, William H. Briggs[§§], Jianwei Lu*, Min Li*, and Gilles Gay*

Address:

*Syngenta Biotechnology, Inc., 3054 E Cornwallis Rd., Research Triangle Park, NC 27709, USA

[§]Syngenta, Inc., 12101Thorps Road, Clinton, IL 61727, USA

**Syngenta, Inc., 317 330th Street, Stanton, MN 55018, USA

[§§]Syngenta Seeds B.V., Westeinde 62, 1601 BK Enkhuizen, The Netherlands

1 *Corresponding Author:* Syngenta Biotechnology, Inc., 3054 E Cornwallis Rd., Research Triangle Park, NC 27709, USA. E-mail: zhigang.guo@syngenta.com

File S1

Method for estimation of variance components and broad sense heritability (H^2)

In this study, variance components and the broad sense heritability on the mean basis were analyzed for each NAM population and trait using phenotypic data from two locations (AU and CU) and two years (2006 and 2007) based on the following mixed model

$$y_{ijk} = \mu + G_i + L_j + Y_k + L_j \times Y_k + G_i \times L_j + G_i \times Y_k + e_{ijk},$$

where y_{ijk} is the phenotypic value of line i at location j and year k, μ is the overall mean of the phenotype, G_i is the genotypic effect of the line i (i = 1, 2, …, N), L_j (j = 1, 2) is the location effect, Y_k (k = 1, 2) is the year effect, $L_j \times Y_k$ is the interaction effect between location j and year k, $G_i \times L_j$ is the interaction effect between genotype i and location j, $G_i \times Y_k$ is the interaction effect between genotype i and year k, and e_{ijk} is the residual error following a normal distribution N(0, V_E). It was assumed that G_i, L_j and Y_k were random effects, following N(0, V_G), N(0, V_L), and N(0, V_Y). Based on these assumptions, the interaction terms $G_i \times L_j$ and $G_i \times Y_k$ were also random effects, following N(0, $V_{G \times L}$) and N(0, $V_{G \times Y}$), respectively. The variance components V_G, V_L, V_Y, $V_{G \times L}$, $V_{G \times Y}$, and V_E were estimated using SAS PROC mixed. Given these variance components, the broad sense heritability H^2 on entry mean basis was estimated as $H^2 = V_G / (V_G + V_{G \times L} / 2 + V_{G \times Y} / 2 + V_E / 4)$.

Table S1 Variance components for traits LL and LW based on 25 NAM populations

	LL							LW						
PopId	V_G	V_E	V_L	V_Y	V_{GL}	V_{GY}	H^2	V_G	V_E	V_L	V_Y	V_{GL}	V_{GY}	H^2
1	3712.69	3415.65	**24.84**	**5.32**	**0.00**	570.36	0.77	49.15	85.97	**12.87**	**0.00**	**0.00**	**0.12**	0.70
2	2828.92	3184.92	**229.33**	**0.00**	**0.00**	**0.00**	0.78	86.33	84.06	**0.95**	**1.57**	**0.00**	**0.58**	0.80
3	1899.01	3922.08	**0.00**	**4.60**	**186.65**	**363.68**	0.60	86.55	90.13	**0.20**	**3.44**	**0.00**	**3.10**	0.78
4	3117.24	2906.74	**82.00**	**132.28**	**101.53**	**0.00**	0.80	42.18	58.18	**5.95**	**0.00**	**0.00**	**0.00**	0.74
5	2640.95	2618.83	**606.87**	**20.66**	**266.01**	**0.00**	0.77	53.61	57.78	**2.28**	**0.00**	**1.00**	**0.00**	0.78
6	2394.58	3030.18	**17.28**	**91.56**	**185.98**	**0.00**	0.74	30.49	89.14	**1.92**	**0.00**	**0.00**	**0.00**	0.58
7	2693.34	2201.03	**90.44**	**401.90**	**53.80**	**0.00**	0.82	80.22	72.46	**0.80**	**16.53**	**0.00**	**0.00**	0.82
8	2643.83	2778.52	**8.20**	**317.28**	**0.00**	**336.46**	0.75	41.57	64.57	**0.89**	**4.29**	**0.00**	**0.00**	0.72
9	2202.03	3048.93	**36.66**	**394.20**	**10.21**	**0.00**	0.74	55.58	78.83	**5.55**	**1.37**	**0.00**	**0.00**	0.74
10	2841.07	2128.14	**28.96**	**136.27**	**0.00**	**63.59**	0.83	64.56	50.93	**0.69**	**4.66**	**1.58**	**7.40**	0.79
11	2923.63	3782.49	**0.00**	**223.94**	**357.97**	**0.00**	0.72	38.51	89.52	**12.38**	**4.27**	**0.00**	**0.00**	0.63
12	3043.85	2414.63	**195.07**	**198.70**	414.83	**0.00**	0.79	54.25	71.07	**0.48**	**0.33**	**2.64**	**0.00**	0.74
13	3096.70	2391.68	**380.26**	**38.62**	**0.00**	**0.00**	0.84	60.14	68.16	**17.25**	**0.00**	**0.00**	**0.00**	0.78
14	2432.85	2367.77	**10.74**	**457.33**	**0.00**	**165.20**	0.78	49.85	53.55	**0.25**	**0.03**	**0.00**	**0.00**	0.79
15	2872.87	2381.87	**0.00**	**43.40**	**259.27**	**215.36**	0.78	62.33	85.35	**0.84**	**0.25**	**0.00**	6.75	0.72
16	1949.81	3066.43	**0.00**	**686.50**	**0.00**	**0.00**	0.72	60.48	78.03	**0.99**	**12.48**	**0.00**	**2.92**	0.74
17	1605.53	3316.92	**1388.21**	**247.90**	**220.81**	**0.00**	0.63	71.48	53.51	**0.00**	**14.17**	**0.00**	5.12	0.82
18	1195.92	2718.24	**86.83**	**104.09**	**81.95**	**0.00**	0.62	26.61	68.20	**0.33**	**5.75**	**0.00**	**1.53**	0.60
19	2329.66	3426.67	**121.82**	**0.00**	**194.02**	521.18	0.66	55.51	55.54	**1.95**	**0.00**	**1.42**	**0.00**	0.79
20	2076.13	1945.32	**364.59**	**95.58**	**50.04**	513.25	0.73	59.99	59.68	**0.00**	**0.97**	**0.00**	**0.00**	0.80
21	3077.63	2317.01	**0.00**	**0.00**	**32.56**	**0.00**	0.84	44.40	64.85	**0.98**	**6.78**	11.19	**0.00**	0.67
22	1987.63	2808.07	**235.30**	**1353.64**	**152.09**	468.61	0.66	66.60	94.38	**0.04**	**29.04**	**6.41**	**0.00**	0.71
23	2171.32	3692.89	**76.03**	**11.10**	**0.00**	**0.00**	0.70	49.86	70.49	**0.36**	**18.46**	**1.59**	**0.00**	0.73
24	2489.81	3212.06	**0.00**	950.74	**0.00**	**0.00**	0.76	58.50	53.30	**0.16**	**2.97**	**3.58**	**0.00**	0.79
25	2212.55	2730.97	301.91	641.40	352.19	**137.16**	0.70	58.45	61.95	**0.14**	6.18	**0.00**	**0.00**	0.79
Mean	2497.58	2872.32	171.41	262.28	116.80	134.19	0.74	56.29	70.39	2.73	5.34	1.18	1.10	0.74

Bold in the table indicates the variance component is not significant at α = 0.05.

Table S2 Estimates of empirical LOD thresholds of CIM based on permutation tests

	LL		LW	
Approach	SE	ME	SE	ME
WP	2.85(0.08)	5.25(0.09)	2.90(0.07)	5.30(0.08)
AP	3.10	6.15	3.13	6.22

For WP-SE, the average of LOD thresholds across 25 NAM populations is illustrated and used for QTL identification with CIM, and in the parenthesis is standard deviation (SD) for LOD threshold estimates across 25 populations

Table S3 Estimates of genetic and residual covariance and correlation (in the lower diagonal) based on individual NAM populations 1 – 10

		LL								LW							
		Genetic covariance				Residual covariance				Genetic covariance				Residual covariance			
PopId	Envi	E1	E2	E3	E4	E1	E2	E3	E4	E1	E2	E3	E4	E1	E2	E3	E4
1	E1	1038.6	1107.1	958.5	959.7	6397.5	700.9	1076.7	1196.0	22.3	15.2	13.7	24.5	155.0	17.7	22.6	18.0
	E2	0.78	1927.2	1460.7	1500.4	0.21	1793.2	593.7	638.0	0.70	21.2	14.2	22.6	0.18	60.3	8.9	12.6
	E3	0.78	0.88	1446.4	1385.8	0.20	0.21	4380.9	1013.4	0.76	0.81	14.4	16.3	0.18	0.12	96.6	14.8
	E4	0.76	0.88	0.93	1526.6	0.21	0.21	0.21	5184.2	0.99	0.94	0.82	27.2	0.19	0.21	0.20	58.4
2	E1	545.9	514.1	489.3	451.8	6560.8	1651.7	2176.5	1953.7	40.9	29.0	29.8	27.1	193.0	34.3	26.4	38.0
	E2	0.66	1125.4	766.9	837.0	0.41	2483.8	1345.4	1217.4	0.93	24.0	23.7	21.7	0.28	79.5	17.0	30.3
	E3	0.57	0.62	1362.7	766.3	0.40	0.41	4424.8	1584.9	0.90	0.93	26.8	22.7	0.22	0.22	76.3	25.4
	E4	0.60	0.77	0.64	1043.2	0.41	0.42	0.41	3429.8	0.86	0.90	0.89	24.2	0.33	0.41	0.35	69.3
3	E1	767.2	352.2	426.1	160.5	6004.0	1815.0	2026.1	1729.6	72.3	43.2	25.5	35.5	127.2	19.7	39.2	24.3
	E2	0.56	523.3	216.3	135.9	0.35	4491.1	1735.4	1504.6	0.88	33.7	22.0	28.9	0.23	57.6	14.7	19.7
	E3	0.59	0.36	684.8	376.3	0.35	0.34	5663.1	1670.5	0.74	0.93	16.6	19.4	0.31	0.17	127.9	31.5
	E4	0.26	0.26	0.63	515.1	0.35	0.35	0.35	4082.0	0.84	0.99	0.95	25.4	0.22	0.26	0.28	96.5
4	E1	1595.5	1091.4	1201.5	1301.4	3903.4	889.7	1143.3	1160.5	21.0	15.6	8.9	18.9	85.1	10.8	16.9	11.2
	E2	0.74	1363.8	1182.9	1348.0	0.33	1828.2	808.4	807.9	0.89	14.7	8.8	17.2	0.22	29.4	11.9	10.7
	E3	0.72	0.77	1746.1	1369.4	0.33	0.34	3141.9	988.2	0.84	0.99	5.3	10.7	0.21	0.25	77.8	15.4
	E4	0.78	0.87	0.78	1751.5	0.33	0.34	0.32	3100.5	0.88	0.96	0.99	21.8	0.16	0.26	0.23	55.8
5	E1	718.2	693.8	823.1	605.4	3447.5	915.5	1391.7	1276.8	11.9	11.4	10.6	15.9	108.0	22.8	44.7	30.3
	E2	0.75	1206.1	943.4	821.1	0.37	1802.2	991.2	956.8	0.86	14.7	15.8	19.7	0.41	28.4	20.9	15.5
	E3	0.73	0.64	1790.9	877.2	0.37	0.37	4060.1	1392.7	0.65	0.87	22.6	21.5	0.41	0.37	110.7	29.4
	E4	0.73	0.76	0.67	963.6	0.36	0.38	0.37	3563.5	0.82	0.92	0.81	31.4	0.40	0.40	0.38	53.9
6	E1	1248.4	909.1	944.9	874.5	3144.4	1001.7	1134.8	1055.9	18.8	8.9	12.8	14.1	109.0	9.3	11.2	9.8
	E2	0.68	1454.5	764.3	956.2	0.35	2592.1	1006.6	982.0	0.64	10.4	7.0	5.8	0.10	73.4	9.3	10.0
	E3	0.87	0.65	952.3	843.9	0.35	0.34	3392.5	1092.8	0.71	0.52	17.3	13.7	0.12	0.12	81.7	11.6
	E4	0.75	0.76	0.83	1092.3	0.34	0.35	0.34	3079.2	0.93	0.51	0.94	12.2	0.12	0.15	0.16	65.0
7	E1	1281.0	882.0	1057.2	1233.8	2408.2	600.7	773.0	606.7	29.6	31.0	24.8	33.8	98.7	19.4	21.1	20.9
	E2	0.86	815.7	802.5	1051.2	0.28	1903.5	674.4	562.0	0.94	37.0	24.8	39.0	0.24	63.9	26.8	15.2
	E3	0.83	0.78	1283.1	1240.9	0.28	0.28	3085.2	697.0	0.99	0.89	21.3	30.0	0.22	0.35	94.5	9.8

	E4	0.81	0.87	0.82	1791.4	0.28	0.29	0.28	1985.8	0.95	0.98	0.99	42.8	0.28	0.25	0.13	58.5
8	E1	1103.5	869.4	696.9	834.2	3801.7	1592.2	1779.6	1405.0	11.9	8.9	13.7	9.5	112.3	30.5	18.1	24.1
	E2	0.69	1451.4	765.1	818.9	0.43	3556.5	1715.7	1318.3	0.80	10.3	10.3	8.5	0.31	86.0	12.9	22.0
	E3	0.57	0.55	1359.0	862.1	0.43	0.42	4620.6	1545.3	0.88	0.71	20.4	11.4	0.23	0.19	56.3	13.1
	E4	0.79	0.68	0.74	1011.5	0.44	0.42	0.43	2749.9	0.82	0.85	0.75	11.3	0.31	0.32	0.24	54.6
9	E1	747.8	479.2	686.4	603.7	2915.3	1007.4	1334.3	1373.2	5.3	9.6	9.3	10.4	161.2	17.4	20.2	19.7
	E2	0.62	802.0	567.7	578.8	0.40	2229.4	1141.7	1228.9	0.72	34.7	22.9	19.3	0.18	56.2	15.8	9.4
	E3	0.75	0.60	1134.5	869.7	0.39	0.38	4005.1	1608.7	0.80	0.77	25.7	12.7	0.18	0.24	76.1	12.6
	E4	0.64	0.59	0.75	1195.1	0.39	0.40	0.39	4303.9	0.62	0.86	0.34	52.9	0.19	0.16	0.18	64.8
10	E1	1184.2	1040.6	975.7	787.3	2403.3	1083.1	1457.7	1187.1	20.8	15.9	23.1	20.4	95.7	34.7	22.8	15.9
	E2	0.82	1377.4	1035.8	938.8	0.48	2132.2	1377.2	1075.3	0.66	27.9	32.2	15.5	0.40	78.7	14.8	19.1
	E3	0.78	0.77	1323.6	930.3	0.47	0.47	3963.7	1481.9	0.74	0.90	46.6	30.2	0.29	0.21	64.8	15.6
	E4	0.66	0.73	0.74	1192.8	0.48	0.46	0.47	2549.0	0.89	0.49	0.75	35.1	0.25	0.33	0.30	43.2

Envi: environment

Table S4 Estimates of genetic and residual covariances and correlations (in the lower diagonal) based on individual NAM populations 11 – 20

		LL								LW							
		Genetic covariance				Residual covariance				Genetic covariance				Residual covariance			
PopId	Envi	E1	E2	E3	E4	E1	E2	E3	E4	E1	E2	E3	E4	E1	E2	E3	E4
11	E1	1477.8	995.6	1208.6	1038.0	5423.9	734.5	904.8	1294.2	22.7	13.8	18.2	20.7	93.7	2.8	5.3	4.0
	E2	0.78	1090.6	880.4	956.9	0.22	2096.7	551.5	809.9	0.72	16.0	15.4	18.9	0.04	51.0	4.0	7.5
	E3	0.84	0.71	1419.1	973.4	0.22	0.22	3096.9	978.2	0.89	0.90	18.4	19.8	0.07	0.07	63.3	5.0
	E4	0.78	0.84	0.75	1196.9	0.22	0.22	0.22	6486.6	0.88	0.95	0.93	24.7	0.05	0.14	0.08	58.4
12	E1	1842.3	1351.7	1272.0	1598.7	3464.1	907.2	1383.5	1123.5	35.2	24.6	20.7	24.2	81.4	12.4	23.4	17.8
	E2	0.86	1330.3	1000.0	1460.9	0.37	1785.6	939.0	800.1	0.83	25.0	13.9	22.5	0.21	43.9	7.8	8.7
	E3	0.85	0.78	1223.1	1194.3	0.37	0.35	3973.9	1185.4	0.95	0.76	13.5	14.9	0.24	0.11	117.8	17.6
	E4	0.83	0.89	0.76	2015.1	0.36	0.36	0.35	2833.8	0.84	0.92	0.83	23.8	0.27	0.18	0.22	52.2
13	E1	1157.0	820.0	1085.7	561.1	2534.7	1538.2	1589.9	2216.2	44.6	20.8	21.8	27.3	89.9	31.5	28.3	18.7
	E2	0.79	926.8	1079.4	635.1	0.61	2482.2	1508.1	2118.0	0.72	18.7	12.5	22.4	0.43	59.0	32.6	17.3
	E3	0.79	0.88	1618.3	638.2	0.58	0.56	2922.8	2239.8	0.89	0.78	13.6	17.1	0.32	0.45	88.0	23.9
	E4	0.69	0.87	0.66	579.5	0.61	0.59	0.58	5145.9	0.87	1.00	0.98	22.3	0.22	0.25	0.29	79.5
14	E1	1044.0	1056.3	541.5	712.2	3710.3	1648.1	1699.0	1545.3	21.6	17.1	13.6	19.1	60.6	21.4	16.2	22.6
	E2	0.80	1660.8	665.6	973.8	0.51	2809.6	1441.5	1360.5	0.93	15.6	12.9	15.1	0.29	88.9	26.7	16.2
	E3	0.68	0.66	607.9	521.3	0.50	0.48	3177.0	1443.4	0.80	0.89	13.5	15.1	0.22	0.30	86.7	20.1
	E4	0.70	0.76	0.67	995.7	0.49	0.50	0.50	2680.9	0.98	0.92	0.98	17.5	0.42	0.25	0.31	48.3
15	E1	1116.9	701.5	432.1	712.4	5458.0	1562.6	2514.5	2265.9	29.7	18.9	15.2	25.0	209.5	22.0	32.3	28.5
	E2	0.76	761.6	505.7	723.7	0.49	1846.1	1451.3	1417.0	0.77	20.4	18.6	25.5	0.25	35.9	8.1	13.3
	E3	0.52	0.73	626.9	613.2	0.49	0.48	4888.6	2165.8	0.54	0.80	26.4	30.3	0.24	0.14	89.1	22.6
	E4	0.68	0.84	0.79	970.6	0.48	0.52	0.49	4079.6	0.77	0.94	0.99	35.7	0.24	0.27	0.29	70.4
16	E1	750.8	418.4	556.6	387.0	3625.6	1213.3	1309.3	1502.7	36.9	27.9	25.6	35.2	121.8	8.3	7.3	8.4
	E2	0.62	614.7	441.7	418.7	0.38	2802.1	1151.5	1343.1	0.79	33.9	22.5	31.9	0.10	54.0	7.4	6.4
	E3	0.67	0.59	924.5	429.5	0.37	0.37	3396.9	1410.2	0.98	0.90	18.5	25.2	0.07	0.11	87.8	12.8
	E4	0.67	0.80	0.67	451.7	0.38	0.39	0.37	4257.6	0.97	0.92	0.98	35.7	0.10	0.12	0.18	55.9
17	E1	585.4	119.0	414.2	386.1	3786.1	1236.2	1156.0	1077.8	36.4	34.5	22.6	35.2	62.6	10.4	15.7	16.7
	E2	0.34	210.5	136.6	285.0	0.30	4471.4	1226.4	1175.0	0.94	37.0	25.1	40.1	0.20	45.2	15.2	9.5
	E3	0.75	0.41	526.9	499.8	0.30	0.29	3945.9	1067.2	0.83	0.91	20.5	27.6	0.21	0.24	85.8	25.2

	E4	0.59	0.73	0.81	720.8	0.31	0.31	0.30	3305.8	0.87	0.99	0.91	44.7	0.26	0.17	0.34	65.3
18	E1	741.1	316.8	168.0	257.1	2964.9	994.9	1188.7	1167.9	26.1	15.7	8.8	9.5	82.3	5.9	5.5	7.0
	E2	0.61	366.0	191.7	278.7	0.36	2524.1	1079.0	1068.2	0.93	11.0	9.2	7.3	0.08	64.3	8.2	5.6
	E3	0.38	0.62	258.9	158.5	0.36	0.35	3702.0	1267.1	0.50	0.80	12.0	7.4	0.07	0.13	67.6	5.9
	E4	0.40	0.62	0.42	559.8	0.36	0.36	0.35	3536.1	0.61	0.72	0.70	9.4	0.11	0.10	0.10	49.0
19	E1	1183.4	691.2	1095.4	608.9	3515.3	1627.3	1454.4	1619.9	22.8	15.4	19.3	19.4	75.5	25.7	24.1	27.8
	E2	0.56	1273.4	731.1	542.0	0.40	4657.2	1637.7	1863.9	0.77	17.4	14.3	16.4	0.34	77.7	19.3	16.4
	E3	0.81	0.52	1566.3	827.9	0.39	0.38	3944.8	1744.0	0.88	0.74	21.3	19.4	0.31	0.25	78.5	17.2
	E4	0.65	0.56	0.77	742.2	0.39	0.39	0.40	4893.3	0.89	0.86	0.92	20.8	0.44	0.25	0.26	53.8
20	E1	1688.2	1084.4	752.9	981.9	2754.9	972.3	1250.2	1241.4	24.4	20.0	19.2	19.4	91.0	23.9	15.0	27.8
	E2	0.80	1098.6	560.2	920.7	0.48	1508.4	942.4	912.0	0.97	17.5	15.3	20.9	0.36	49.8	22.8	19.3
	E3	0.67	0.62	748.9	759.5	0.44	0.45	2875.9	1313.2	0.83	0.78	21.9	21.8	0.18	0.36	79.5	17.9
	E4	0.63	0.73	0.73	1444.0	0.45	0.45	0.46	2789.6	0.78	0.99	0.92	25.6	0.40	0.38	0.28	52.7

Envi: environment

Table S5 Estimates of genetic and residual covariances and correlations (in the lower diagonal) based on individual NAM populations 21-25

		LL								LW							
		Genetic covariance				Residual covariance				Genetic covariance				Residual covariance			
PopId	Envi	E1	E2	E3	E4	E1	E2	E3	E4	E1	E2	E3	E4	E1	E2	E3	E4
21	E1	1153.0	1104.6	913.2	905.8	2485.8	1047.1	1313.0	965.1	32.4	16.3	18.8	17.0	69.6	4.7	15.0	11.0
	E2	0.81	1603.0	1203.6	1269.5	0.44	2329.9	1258.4	943.2	0.68	17.6	9.5	9.2	0.07	69.84	7.07	12.5
	E3	0.79	0.88	1167.9	1097.4	0.43	0.43	3690.1	1186.7	0.65	0.45	25.8	13.9	0.18	0.09	98.2	6.8
	E4	0.72	0.85	0.86	1391.6	0.41	0.42	0.42	2212.9	0.82	0.60	0.75	13.4	0.16	0.19	0.09	64.7
22	E1	1000.1	1000.2	477.3	375.1	4400.6	1041.3	1010.4	996.9	30.4	25.9	18.9	20.9	243.8	17.8	28.6	15.5
	E2	0.71	1972.3	625.6	694.1	0.29	2934.1	831.3	839.4	0.84	31.5	16.6	25.0	0.15	56.6	15.2	13.3
	E3	0.57	0.53	707.1	552.6	0.29	0.29	2788.5	808.0	0.95	0.82	13.0	14.4	0.19	0.21	92.2	11.8
	E4	0.42	0.56	0.74	786.1	0.29	0.29	0.29	2780.3	0.83	0.98	0.87	21.0	0.14	0.24	0.17	52.1
23	E1	1329.2	842.1	765.1	831.6	3094.9	776.0	778.0	872.5	17.0	13.4	18.3	19.9	102.6	27.2	21.4	20.8
	E2	0.70	1097.0	633.3	703.5	0.24	3333.9	800.1	905.6	0.69	22.3	14.0	18.0	0.27	96.7	21.1	19.7
	E3	0.85	0.78	603.7	622.1	0.24	0.24	3317.6	905.5	0.98	0.65	20.5	14.3	0.27	0.27	62.0	15.0
	E4	0.72	0.67	0.80	995.9	0.24	0.24	0.24	4239.4	0.99	0.78	0.65	23.5	0.27	0.27	0.25	56.6
24	E1	1495.4	541.3	724.0	537.2	3241.0	1437.8	1738.8	1528.7	39.5	22.4	24.7	27.0	72.2	12.9	19.4	19.1
	E2	0.78	318.5	427.3	306.8	0.43	3479.4	1815.5	1611.7	0.78	20.8	18.2	17.6	0.19	66.6	18.2	18.5
	E3	0.53	0.68	1242.5	610.6	0.42	0.43	5179.5	1917.9	0.93	0.94	18.1	17.1	0.25	0.24	83.8	13.8
	E4	0.54	0.67	0.67	664.0	0.42	0.43	0.42	4031.1	0.95	0.85	0.88	20.6	0.31	0.31	0.21	52.6
25	E1	1607.2	918.9	1127.7	697.3	2857.2	1072.6	1288.7	1393.5	29.9	18.1	16.0	20.1	85.2	28.3	26.8	22.6
	E2	0.73	978.6	727.8	766.6	0.41	2432.8	1174.9	1302.6	0.59	31.8	9.3	30.3	0.44	47.6	26.7	26.9
	E3	0.77	0.63	1348.0	666.3	0.40	0.39	3725.9	1592.9	0.85	0.48	12.0	11.8	0.28	0.37	110.2	29.9
	E4	0.59	0.82	0.61	883.6	0.40	0.40	0.40	4337.6	0.61	0.89	0.56	36.5	0.32	0.51	0.37	59.4

Envi: environment

Table S6 Estimates of genetic and residual covariance and correlation (in the lower diagonal) based on a full data set of 25 NAM populations.

	LL								LW							
	Genetic covariance				Residual covariance				Genetic covariance				Residual covariance			
Envi	E1	E2	E3	E4	E1	E2	E3	E4	E1	E2	E3	E4	E1	E2	E3	E4
E1	401.98	357.82	364.59	345.25	5097.97	2021.13	2029.87	1977.42	12.94	9.743	9.70	10.97	137.20	38.03	35.99	42.44
E2	0.83	460.65	351.99	393.84	0.45	3882.87	1719.01	1992.78	0.90	8.97	7.72	9.44	0.36	81.45	81.45	30.95
E3	0.88	0.79	427.45	373.01	0.40	0.38	5261.99	2016.00	0.91	0.87	8.74	8.92	0.30	0.34	104.49	35.47
E4	0.82	0.87	0.86	440.38	0.40	0.46	0.40	4872.04	0.92	0.95	0.91	11.09	0.39	0.44	0.38	85.04

Envi: environment

Table S7 Accuracy of WP prediction for environment E1 with QP and GWP in CV1

	LL				LW			
	SE		ME		SE		ME	
PopId	QP[a]	GWP[b]	QP[c]	GWP[d]	QP[a]	GWP[b]	QP[c]	GWP[d]
1	0.25(1.5)	0.33(0.32)	0.34(3.3, 0.36)	0.42(0.24, 0.27)	0.06(1.4)	0.29(3.83)	0.11(2.1, 0.83)	0.39(2.55, 0.34)
2	0.02(1.1)	0.17(7.50)	0.01(1.6, **-0.50**)	0.24(23.00, 0.41)	0.25(2.0)	0.40(0.60)	0.18(1.8, -0.28)	0.45(1.50, 0.12)
3	0.09(1.2)	0.28(2.11)	0.08(1.7, **-0.11**)	0.28(2.50, **0.00**)	0.39(3.2)	0.55(0.41)	0.36(3.0, -0.08)	0.59(0.64, 0.07)
4	0.24(2.8)	0.48(1.00)	0.22(3.3, **-0.08**)	0.50(1.27, 0.04)	0.25(2.6)	0.49(0.96)	0.21(2.4, -0.16)	0.53(1.52, 0.08)
5	0.16(2.0)	0.32(1.00)	0.22(3.0, 0.38)	0.39(0.77, 0.22)	0.05(1.4)	0.20(3.00)	0.03(1.6, **-0.40**)	0.29(8.67, 0.45)
6	0.26(2.4)	0.50(0.92)	0.22(2.8, -0.15)	0.53(1.41, 0.06)	0.03(1.4)	0.32(9.67)	0.06(1.6, 1.00)	0.38(5.33, 0.19)
7	0.33(2.7)	0.52(0.58)	0.29(2.4, -0.12)	0.57(0.97, 0.10)	0.26(2.8)	0.48(0.85)	0.22(2.5, -0.15)	0.52(1.36, 0.08)
8	0.07(1.6)	0.31(3.43)	0.08(2.0, **0.14**)	0.35(3.37, 0.13)	0.06(1.4)	0.31(4.17)	0.16(1.9, 1.67)	0.38(1.38, 0.23)
9	0.31(2.3)	0.34(0.10)	0.25(2.5, -0.19)	0.38(0.52, 0.12)	0.02(1.1)	0.17(7.50)	0.13(2.1, 5.50)	0.36(1.77, 1.12)
10	0.26(2.4)	0.47(0.81)	0.22(2.8, -0.15)	0.49(1.23, 0.04)	0.20(2.5)	0.39(0.95)	0.17(3.1, **-0.15**)	0.52(2.06, 0.33)
11	0.26(2.4)	0.37(0.42)	0.27(3.0, **0.04**)	0.45(0.67, 0.22)	0.13(2.5)	0.39(2.00)	0.15(3.1, **0.15**)	0.42(1.80, 0.08)
12	0.24(3.0)	0.49(1.04)	0.20(2.6, -0.17)	0.54(1.70, 0.10)	0.29(3.1)	0.54(0.86)	0.31(3.3, 0.07)	0.57(0.84, 0.06)
13	0.20(2.7)	0.45(1.25)	0.16(2.9, -0.20)	0.47(1.94, 0.04)	0.34(3.0)	0.50(0.47)	0.28(3.5, -0.18)	0.52(0.86, 0.04)
14	0.13(2.0)	0.38(1.92)	0.14(2.3, **0.08**)	0.42(2.00, 0.11)	0.20(2.4)	0.47(1.35)	0.14(1.9, -0.30)	0.50(2.57, 0.06)
15	0.09(1.6)	0.28(2.11)	0.07(1.9, **-0.22**)	0.31(3.43, 0.11)	0.13(1.8)	0.30(1.31)	0.12(2.8, **-0.08**)	0.34(1.83, 0.13)
16	0.22(1.9)	0.33(0.50)	0.18(2.0, -0.18)	0.36(1.00, 0.09)	0.24(2.4)	0.46(0.92)	0.31(3.2, 0.29)	0.54(0.74, 0.17)
17	0.03(1.2)	0.27(8.00)	0.04(1.2, **0.33**)	0.29(6.25, 0.07)	0.33(3.0)	0.55(0.67)	0.33(3.7, **0.00**)	0.59(0.79, 0.07)
18	0.08(1.2)	0.28(2.50)	0.03(1.2, -0.62)	0.28(8.33, **0.00**)	0.19(2.1)	0.39(1.05)	0.23(2.5, 0.21)	0.43(0.87, 0.10)
19	0.29(2.4)	0.37(0.28)	0.20(2.1, -0.31)	0.40(1.00, 0.08)	0.29(2.2)	0.41(0.41)	0.28(2.7, **-0.03**)	0.44(0.57, 0.07)
20	0.15(2.6)	0.47(2.13)	0.09(2.4, -0.40)	0.48(4.33, 0.02)	0.21(2.2)	0.47(1.24)	0.29(2.7, 0.38)	0.51(0.76, 0.09)
21	0.35(3.1)	0.49(0.40)	0.32(3.4, **-0.09**)	0.51(0.59, 0.04)	0.19(2.2)	0.47(1.47)	0.11(2.1, -0.42)	0.48(3.36, 0.02)
22	0.16(1.5)	0.35(1.19)	0.19(2.4, **0.19**)	0.41(1.16, 0.17)	0.03(1.2)	0.32(9.67)	0.12(2.0, 3.00)	0.42(2.50, 0.31)
23	0.31(3.1)	0.42(0.35)	0.24(2.6, -0.23)	0.46(0.92, 0.10)	0.06(1.8)	0.29(3.83)	0.17(2.9, 1.83)	0.43(1.53, 0.48)
24	0.18(2.7)	0.45(1.50)	0.11(1.8, -0.39)	0.44(3.00, -0.02)	0.33(3.1)	0.50(0.52)	0.36(3.0, 0.09)	0.53(0.47, 0.06)
25	0.20(2.8)	0.50(1.50)	0.20(2.6, **0.00**)	0.51(1.55, 0.02)	0.26(2.9)	0.45(0.73)	0.25(2.4, **-0.04**)	0.48(0.92, 0.07)
Mean	0.20(2.2)	0.38(0.93)	0.17(2.4, -0.10)	0.42(1.40, 0.10)	0.19(2.2)	0.40(1.11)	0.20(2.6, 0.06)	0.46(1.29, 0.15)

[a] In parentheses is the number of QTL identified by QP based on the SE model; [b] In parentheses is the gain in prediction accuracy with GWP over QP based on the SE model; [c] The first value in parentheses is the number of QTL identified by QP based on the ME model; and the second one the gain

with ME over SE for QP; [d] The first value in parentheses is the gain in accuracy with GWP over QP based on the ME model; and the second one is the gain with ME over SE using GWP. Bold in parentheses indicates the number is not significant at $\alpha = 0.05$.

Table S8 Accuracy of WP prediction for environment E2 with QP and GWP in CV1

	LL				LW			
	SE		ME		SE		ME	
PopId	QP[a]	GWP[b]	QP[c]	GWP[d]	QP[a]	GWP[b]	QP[c]	GWP[d]
1	0.50(4.0)	0.67(0.34)	0.48(3.3, **-0.04**)	0.67(0.40, **0.00**)	0.17(2.5)	0.46(1.71)	0.16(2.1, **-0.06**)	0.50(2.12, 0.09)
2	0.11(2.2)	0.40(2.64)	0.05(1.6, -0.55)	0.40(7.00, **0.00**)	0.21(2.5)	0.49(1.33)	0.17(1.8, -0.19)	0.53(2.12, 0.08)
3	0.21(1.8)	0.17(-0.19)	0.09(1.7, -0.57)	0.18(1.00, 0.06)	0.35(3.3)	0.59(0.69)	0.35(3.0, **0.00**)	0.61(0.74, 0.03)
4	0.20(2.9)	0.56(1.80)	0.25(3.3, 0.25)	0.57(1.28, 0.02)	0.41(3.0)	0.59(0.44)	0.28(2.4, -0.32)	0.62(1.21, 0.05)
5	0.27(3.0)	0.50(0.85)	0.25(3.0, **-0.07**)	0.51(1.04, 0.02)	0.07(2.4)	0.45(5.43)	0.05(1.6, **-0.29**)	0.46(8.20, 0.02)
6	0.22(2.6)	0.49(1.23)	0.17(2.8, -0.23)	0.50(1.94, 0.02)	0.07(1.2)	0.33(3.71)	0.09(1.6, **0.29**)	0.34(2.78, 0.03)
7	0.25(2.0)	0.42(0.68)	0.25(2.4, **0.00**)	0.51(1.04, 0.21)	0.35(3.0)	0.58(0.66)	0.27(2.5, -0.23)	0.62(1.30, 0.07)
8	0.10(1.9)	0.35(2.50)	0.11(2.0, **0.10**)	0.37(2.36, 0.06)	0.21(1.6)	0.35(0.67)	0.21(1.9, **0.00**)	0.42(1.00, 0.20)
9	0.07(2.0)	0.30(3.29)	0.11(2.5, 0.57)	0.32(1.91, 0.07)	0.22(2.3)	0.44(1.00)	0.25(2.1, 0.14)	0.43(0.72, -0.02)
10	0.19(2.7)	0.50(1.63)	0.18(2.8, **-0.05**)	0.51(1.83, 0.02)	0.14(1.9)	0.36(1.57)	0.18(3.1, 0.29)	0.36(1.00, **0.00**)
11	0.26(2.8)	0.52(1.00)	0.31(3.0, 0.19)	0.55(0.77, 0.06)	0.27(2.0)	0.40(0.48)	0.25(3.1, **-0.07**)	0.43(0.72, 0.07)
12	0.22(3.0)	0.56(1.55)	0.21(2.6, **-0.05**)	0.59(1.81, 0.05)	0.27(3.4)	0.58(1.15)	0.30(3.3, 0.11)	0.61(1.03, 0.05)
13	0.21(2.4)	0.40(0.90)	0.19(2.9, **-0.10**)	0.45(1.37, 0.12)	0.18(2.1)	0.39(1.17)	0.19(3.5, **0.06**)	0.47(1.47, 0.21)
14	0.32(2.9)	0.42(0.31)	0.19(2.3, -0.41)	0.42(1.21, **0.00**)	0.05(1.4)	0.27(4.40)	0.07(1.9, **0.40**)	0.36(4.14, 0.33)
15	0.25(3.0)	0.46(0.84)	0.15(1.9, -0.40)	0.46(2.07, **0.00**)	0.21(2.9)	0.55(1.62)	0.18(2.8, -0.14)	0.58(2.22, 0.05)
16	0.19(2.1)	0.31(0.63)	0.17(2.0, **-0.11**)	0.32(0.88, 0.03)	0.44(3.3)	0.56(0.27)	0.36(3.2, -0.18)	0.59(0.64, 0.05)
17	0.04(0.9)	0.13(2.25)	0.02(1.2, **-0.50**)	0.19(8.50, 0.46)	0.40(3.5)	0.61(0.52)	0.38(3.7, **-0.05**)	0.64(0.68, 0.05)
18	0.02(1.1)	0.29(13.50)	0.03(1.2, **0.50**)	0.31(9.33, 0.07)	0.18(1.9)	0.30(0.67)	0.27(2.5, 0.50)	0.43(0.59, 0.43)
19	0.08(1.5)	0.28(2.50)	0.05(2.1, -0.38)	0.30(5.00, 0.07)	0.20(2.3)	0.40(1.00)	0.25(2.7, 0.25)	0.46(0.84, 0.15)
20	0.11(2.2)	0.52(3.73)	0.10(2.4, **-0.09**)	0.54(4.40, 0.04)	0.31(3.0)	0.53(0.71)	0.31(2.7, **0.00**)	0.56(0.81, 0.06)
21	0.35(3.3)	0.58(0.66)	0.35(3.4, **0.00**)	0.61(0.74, 0.05)	0.14(1.8)	0.35(1.50)	0.08(2.1, -0.43)	0.40(4.00, 0.14)
22	0.34(2.6)	0.49(0.44)	0.22(2.4, -0.35)	0.50(1.27, 0.02)	0.32(2.9)	0.58(0.81)	0.23(2.0, -0.28)	0.60(1.61, 0.03)
23	0.16(2.4)	0.43(1.69)	0.16(2.6, **0.00**)	0.46(1.88, 0.07)	0.24(2.0)	0.36(0.50)	0.21(2.9, **-0.12**)	0.41(0.95, 0.14)
24	0.01(1.2)	0.16(15.00)	0.06(1.8, 5.00)	0.25(3.17, 0.56)	0.25(2.2)	0.42(0.68)	0.32(3.0, 0.28)	0.48(0.50, 0.14)
25	0.10(2.2)	0.38(2.80)	0.05(2.6, -0.50)	0.42(7.40, 0.11)	0.29(2.7)	0.47(0.62)	0.27(2.4, **-0.07**)	0.45(0.67, -0.04)
Mean	0.19(2.4)	0.41(1.15)	0.17(2.4, -0.12)	0.44(1.60, 0.06)	0.24(2.4)	0.46(0.92)	0.23(2.6, -0.05)	0.49(1.18, 0.08)

[a] In parentheses is the number of QTL identified by QP based on the SE model; [b] In parentheses is the gain in prediction accuracy with GWP over QP based on the SE model; [c] The first value in parentheses is the number of QTL identified by QP based on the ME model; and the second one the gain

with ME over SE for QP; [d] The first value in parentheses is the gain in accuracy with GWP over QP based on the ME model; and the second one is the gain with ME over SE using GWP. Bold in parentheses indicates the number is not significant at $\alpha = 0.05$.

Table S9 Accuracy of WP prediction for environment E3 with QP and GWP in CV1

	LL				LW			
	SE		ME		SE		ME	
PopId	QP[a]	GWP[b]	QP[c]	GWP[d]	QP[a]	GWP[b]	QP[c]	GWP[d]
1	0.32(2.8)	0.51(0.59)	0.34(3.3, **0.06**)	0.55(0.62, 0.08)	0.12(1.5)	0.36(2.00)	0.13(2.1, 0.08)	0.41(2.15, 0.14)
2	0.09(1.7)	0.35(2.89)	0.04(1.6, -0.56)	0.36(8.00, 0.03)	0.23(2.8)	0.52(1.26)	0.16(1.8, -0.30)	0.55(2.44, 0.06)
3	0.07(1.3)	0.24(2.43)	0.06(1.7, **-0.14**)	0.26(3.33, 0.08)	0.27(2.1)	0.27(0.00)	0.27(3.0, **0.00**)	0.37(0.37, 0.37)
4	0.30(2.9)	0.50(0.67)	0.28(3.3, **-0.07**)	0.54(0.93, 0.08)	0.01(1.3)	0.18(17.00)	0.05(2.4, 4.00)	0.29(4.80, 0.61)
5	0.23(2.7)	0.44(0.91)	0.21(3.0, **-0.09**)	0.46(1.19, 0.05)	0.04(1.4)	0.30(6.50)	0.04(1.6, 0.00)	0.39(8.75, 0.30)
6	0.36(2.5)	0.47(0.31)	0.24(2.8, -0.33)	0.54(1.25, 0.15)	0.09(1.8)	0.31(2.44)	0.06(1.6, -0.33)	0.35(4.83, 0.13)
7	0.26(2.4)	0.49(0.88)	0.25(2.4, **-0.04**)	0.55(1.20, 0.12)	0.19(2.0)	0.42(1.21)	0.21(2.5, **0.11**)	0.50(1.38, 0.19)
8	0.06(1.7)	0.32(4.33)	0.07(2.0, **0.17**)	0.35(4.00, 0.09)	0.14(2.2)	0.48(2.43)	0.15(1.9, **0.07**)	0.49(2.27, 0.02)
9	0.18(2.3)	0.42(1.33)	0.22(2.5, 0.22)	0.45(1.05, 0.07)	0.17(2.6)	0.45(1.65)	0.25(2.1, 0.47)	0.48(0.92, 0.07)
10	0.11(2.6)	0.42(2.82)	0.12(2.8, **0.09**)	0.48(3.00, 0.14)	0.33(3.3)	0.56(0.70)	0.25(3.1, -0.24)	0.56(1.24, **0.00**)
11	0.31(2.7)	0.54(0.74)	0.31(3.0, **0.00**)	0.56(0.81, 0.04)	0.10(2.1)	0.36(2.60)	0.11(3.1, **0.10**)	0.41(2.73, 0.14)
12	0.18(2.3)	0.43(1.39)	0.17(2.6, **-0.06**)	0.50(1.94, 0.16)	0.07(1.3)	0.28(3.00)	0.17(3.3, 1.43)	0.38(1.24, 0.36)
13	0.19(2.8)	0.41(1.16)	0.19(2.9, **0.00**)	0.43(1.26, 0.05)	0.06(2.0)	0.26(3.33)	0.15(3.5, 1.50)	0.35(1.33, 0.35)
14	0.16(2.1)	0.31(0.94)	0.12(2.3, -0.25)	0.35(1.92, 0.13)	0.10(1.6)	0.32(2.20)	0.12(1.9, **0.20**)	0.42(2.50, 0.31)
15	0.05(1.4)	0.25(4.00)	0.08(1.9, **0.60**)	0.30(2.75, 0.20)	0.16(2.0)	0.40(1.50)	0.18(2.8, **0.12**)	0.48(1.67, 0.20)
16	0.10(1.9)	0.32(2.20)	0.07(2.0, -0.30)	0.33(3.71, 0.03)	0.08(1.7)	0.36(3.50)	0.21(3.2, 1.62)	0.45(1.14, 0.25)
17	0.07(1.5)	0.32(3.57)	0.07(1.2, **0.00**)	0.37(4.29, 0.16)	0.12(2.0)	0.38(2.17)	0.23(3.7, 0.92)	0.45(0.96, 0.18)
18	0.01(1.0)	0.17(16.00)	0.03(1.2, **2.00**)	0.21(6.00, 0.24)	0.33(2.4)	0.33(0.00)	0.30(2.5, -0.09)	0.39(0.30, 0.18)
19	0.25(2.8)	0.46(0.84)	0.20(2.1, -0.20)	0.47(1.35, 0.02)	0.21(2.1)	0.44(1.10)	0.29(2.7, 0.38)	0.46(0.59, 0.05)
20	0.13(1.9)	0.34(1.62)	0.05(2.4, -0.62)	0.39(6.80, 0.15)	0.15(2.1)	0.43(1.87)	0.24(2.7, 0.60)	0.46(0.92, 0.07)
21	0.24(2.6)	0.47(0.96)	0.33(3.4, 0.38)	0.54(0.64, 0.15)	0.21(2.5)	0.38(0.81)	0.11(2.1, -0.48)	0.42(2.82, 0.11)
22	0.21(1.9)	0.38(0.81)	0.22(2.4, **0.05**)	0.40(0.82, 0.05)	0.09(1.4)	0.35(2.89)	0.11(2.0, **0.22**)	0.43(2.91, 0.23)
23	0.21(1.8)	0.32(0.52)	0.21(2.6, **0.00**)	0.42(1.00, 0.31)	0.14(2.1)	0.38(1.71)	0.20(2.9, 0.43)	0.42(1.10, 0.11)
24	0.04(1.6)	0.26(5.50)	0.05(1.8, **0.25**)	0.27(4.40, 0.04)	0.34(2.2)	0.41(0.21)	0.38(3.0, 0.12)	0.48(0.26, 0.17)
25	0.11(2.0)	0.37(2.36)	0.15(2.6, 0.36)	0.40(1.67, 0.08)	0.11(1.6)	0.29(1.64)	0.16(2.4, 0.45)	0.34(1.12, 0.17)
Mean	0.17(2.1)	0.38(1.24)	0.16(2.4, -0.04)	0.42(1.57, 0.10)	0.15(2.0)	0.37(1.40)	0.18(2.6, 0.18)	0.43(1.37, 0.16)

[a] In parentheses is the number of QTL identified by QP based on the SE model; [b] In parentheses is the gain in prediction accuracy with GWP over QP based on the SE model; [c] The first value in parentheses is the number of QTL identified by QP based on the ME model; and the second one the gain

with ME over SE for QP; [d] The first value in parentheses is the gain in accuracy with GWP over QP based on the ME model; and the second one is the gain with ME over SE using GWP. Bold in parentheses indicates the number is not significant at $\alpha = 0.05$.

Table S10 Accuracy of WP prediction for environment E4 with QP and GWP in CV1

	LL				LW			
	SE		ME		SE		ME	
PopId	QP[a]	GWP[b]	QP[c]	GWP[d]	QP[a]	GWP[b]	QP[c]	GWP[d]
1	0.31(2.5)	0.47(0.52)	0.36(3.3, 0.16)	0.51(0.42, 0.09)	0.26(2.7)	0.50(0.92)	0.21(2.1, -0.19)	0.54(1.57, 0.08)
2	0.06(1.7)	0.30(4.00)	0.02(1.6, -0.67)	0.32(15.00, 0.07)	0.22(2.4)	0.48(1.18)	0.14(1.8, -0.36)	0.51(2.64, 0.06)
3	0.08(1.3)	0.26(2.25)	0.04(1.7, -0.50)	0.27(5.75, 0.04)	0.29(2.5)	0.42(0.45)	0.30(3.0, **0.03**)	0.52(0.73, 0.24)
4	0.31(3.0)	0.52(0.68)	0.32(3.3, **0.03**)	0.56(0.75, 0.08)	0.35(2.8)	0.51(0.46)	0.26(2.4, -0.26)	0.54(1.08, 0.06)
5	0.08(1.8)	0.35(3.37)	0.12(3.0, 0.50)	0.39(2.25, 0.11)	0.08(2.1)	0.45(4.62)	0.04(1.6, -0.50)	0.47(10.75, 0.04)
6	0.18(2.6)	0.45(1.50)	0.23(2.8, 0.28)	0.51(1.22, 0.13)	0.08(1.7)	0.30(2.75)	0.03(1.6, -0.62)	0.36(11.00, 0.20)
7	0.34(2.5)	0.60(0.76)	0.29(2.4, -0.15)	0.62(1.14, 0.03)	0.39(3.4)	0.62(0.59)	0.29(2.5, -0.26)	0.65(1.24, 0.05)
8	0.21(2.1)	0.40(0.90)	0.15(2.0, -0.29)	0.43(1.87, 0.07)	0.27(2.3)	0.40(0.48)	0.22(1.9, -0.19)	0.45(1.05, 0.12)
9	0.10(1.9)	0.34(2.40)	0.10(2.5, **0.00**)	0.37(2.70, 0.09)	0.34(2.6)	0.47(0.38)	0.32(2.1, **-0.06**)	0.47(0.47, **0.00**)
10	0.16(2.4)	0.41(1.56)	0.15(2.8, **-0.06**)	0.43(1.87, 0.05)	0.26(3.2)	0.63(1.42)	0.24(3.1, **-0.08**)	0.64(1.67, 0.02)
11	0.14(2.1)	0.29(1.07)	0.25(3.0, 0.79)	0.38(0.52, 0.31)	0.18(2.6)	0.44(1.44)	0.23(3.1, 0.28)	0.49(1.13, 0.11)
12	0.17(2.7)	0.50(1.94)	0.18(2.6, **0.06**)	0.54(2.00, 0.08)	0.35(3.4)	0.57(0.63)	0.34(3.3, **-0.03**)	0.60(0.76, 0.05)
13	0.11(1.3)	0.24(1.18)	0.13(2.9, **0.18**)	0.34(1.62, 0.42)	0.17(1.9)	0.42(1.47)	0.23(3.5, 0.35)	0.49(1.13, 0.17)
14	0.08(2.2)	0.34(3.25)	0.11(2.3, 0.38)	0.37(2.36, 0.09)	0.25(2.3)	0.45(0.80)	0.15(1.9, -0.40)	0.49(2.27, 0.09)
15	0.20(1.8)	0.36(0.80)	0.11(1.9, -0.45)	0.37(2.36, 0.03)	0.30(3.2)	0.53(0.77)	0.23(2.8, -0.23)	0.56(1.43, 0.06)
16	0.07(1.3)	0.20(1.86)	0.13(2.0, 0.86)	0.28(1.15, 0.40)	0.29(2.9)	0.53(0.83)	0.30(3.2, **0.03**)	0.57(0.90, 0.08)
17	0.14(1.8)	0.34(1.43)	0.06(1.2, -0.57)	0.36(5.00, 0.06)	0.32(3.3)	0.56(0.75)	0.34(3.7, **0.06**)	0.61(0.79, 0.09)
18	0.05(1.2)	0.26(4.20)	0.02(1.2, -0.60)	0.28(13.00, 0.08)	0.20(2.0)	0.31(0.55)	0.28(2.5, 0.40)	0.38(0.36, 0.23)
19	0.06(1.7)	0.26(3.33)	0.09(2.1, 0.50)	0.31(2.44, 0.19)	0.42(3.4)	0.51(0.21)	0.35(2.7, -0.17)	0.55(0.57, 0.08)
20	0.13(2.5)	0.46(2.54)	0.10(2.4, -0.23)	0.48(3.80, 0.04)	0.49(2.6)	0.52(0.06)	0.39(2.7, -0.20)	0.55(0.41, 0.06)
21	0.37(3.1)	0.61(0.65)	0.37(3.4, **0.00**)	0.64(0.73, 0.05)	0.08(1.8)	0.36(3.50)	0.05(2.1, -0.38)	0.41(7.20, 0.14)
22	0.28(2.2)	0.47(0.68)	0.23(2.4, -0.18)	0.48(1.09, 0.02)	0.27(2.8)	0.55(1.04)	0.19(2.0, -0.30)	0.59(2.11, 0.07)
23	0.25(2.0)	0.39(0.56)	0.22(2.6, **-0.12**)	0.45(1.05, 0.15)	0.27(3.0)	0.48(0.78)	0.27(2.9, **0.00**)	0.51(0.89, 0.06)
24	0.19(1.8)	0.26(0.37)	0.06(1.8, -0.68)	0.29(3.83, 0.12)	0.31(3.2)	0.47(0.52)	0.33(3.0, **0.06**)	0.51(0.55, 0.09)
25	0.04(1.3)	0.26(5.50)	0.06(2.6, **0.50**)	0.33(4.50, 0.27)	0.27(2.4)	0.48(0.78)	0.28(2.4, **0.04**)	0.47(0.68, -0.02)
Mean	0.16(2.0)	0.37(1.27)	0.16(2.4, 0.00)	0.41(1.64, 0.10)	0.27(2.7)	0.48(0.78)	0.24(2.6, -0.10)	0.52(1.15, 0.08)

[a] In parentheses is the number of QTL identified by QP based on the SE model; [b] In parentheses is the gain in prediction accuracy with GWP over QP based on the SE model; [c] The first value in parentheses is the number of QTL identified by QP based on the ME model; and the second one the gain

with ME over SE for QP; [d] The first value in parentheses is the gain in accuracy with GWP over QP based on the ME model; and the second one is the gain with ME over SE using GWP. Bold in parentheses indicates the number is not significant at $\alpha = 0.05$.

Table S11 Accuracy of AP prediction for environment E1 with QP and GWP in CV1

	SE		ME		SE		ME	
PopId	QP[a]	GWP[b]	QP[c]	GWP[d]	QP[a]	GWP[b]	QP[c]	GWP[d]
1	0.21(9.0)	0.31(0.48)	0.28(14.0, 0.33)	0.33(0.18, 0.06)	0.19(13.0)	0.25(0.32)	0.21(12.0, 0.11)	0.26(0.24, 0.04)
2	0.20(9.0)	0.19(-0.05)	0.21(14.0, **0.05**)	0.20(-0.05, 0.05)	0.37(13.0)	0.42(0.14)	0.39(12.0, 0.05)	0.42(0.08, **0.00**)
3	0.21(10.0)	0.21(**0.00**)	0.20(15.0, **-0.05**)	0.20(**0.00**, **-0.05**)	0.41(13.0)	0.54(0.32)	0.46(11.0, 0.12)	0.55(0.20, 0.02)
4	0.37(12.0)	0.41(0.11)	0.43(12.0, 0.16)	0.41(-0.05, **0.00**)	0.28(14.0)	0.29(0.04)	0.22(12.0, -0.21)	0.28(0.27, -0.03)
5	0.31(11.0)	0.28(-0.10)	0.23(14.0, -0.26)	0.26(0.13, -0.07)	0.16(13.0)	0.23(0.44)	0.10(12.0, -0.38)	0.24(1.40, 0.04)
6	0.40(10.0)	0.46(0.15)	0.46(13.0, 0.15)	0.47(0.02, 0.02)	0.36(13.0)	0.37(0.03)	0.40(12.0, 0.11)	0.39(-0.03, 0.05)
7	0.23(11.0)	0.24(0.04)	0.27(14.0, 0.17)	0.26(-0.04, 0.08)	0.34(12.0)	0.36(0.06)	0.36(12.0, 0.06)	0.39(0.08, 0.08)
8	0.41(9.0)	0.47(0.15)	0.44(15.0, 0.07)	0.47(0.07, **0.00**)	0.32(12.0)	0.39(0.22)	0.32(12.0, **0.00**)	0.39(0.22, **0.00**)
9	0.36(10.0)	0.36(**0.00**)	0.33(13.0, -0.08)	0.38(0.15, 0.06)	0.18(15.0)	0.16(-0.11)	0.22(13.0, 0.22)	0.17(-0.23, 0.06)
10	0.30(10.0)	0.37(0.23)	0.37(13.0, 0.23)	0.38(0.03, 0.03)	0.42(12.0)	0.46(0.10)	0.48(13.0, 0.14)	0.48(**0.00**, 0.04)
11	0.03(10.0)	0.07(1.33)	0.08(15.0, 1.67)	0.09(0.13, 0.29)	0.11(13.0)	0.17(0.55)	0.19(11.0, 0.73)	0.18(-0.05, 0.06)
12	0.34(9.0)	0.39(0.15)	0.37(15.0, 0.09)	0.40(0.08, 0.03)	0.51(12.0)	0.59(0.16)	0.54(11.0, 0.06)	0.61(0.13, 0.03)
13	0.28(12.0)	0.29(0.04)	0.35(13.0, 0.25)	0.32(-0.09, 0.10)	0.49(14.0)	0.42(-0.14)	0.50(12.0, **0.02**)	0.43(-0.14, 0.02)
14	0.35(10.0)	0.40(0.14)	0.38(12.0, 0.09)	0.40(0.05, **0.00**)	0.39(14.0)	0.36(-0.08)	0.41(12.0, 0.05)	0.38(-0.07, 0.06)
15	0.22(12.0)	0.24(0.09)	0.20(15.0, -0.09)	0.24(0.20, **0.00**)	0.27(14.0)	0.31(0.15)	0.23(12.0, -0.15)	0.31(0.35, **0.00**)
16	0.18(11.0)	0.27(0.50)	0.23(13.0, 0.28)	0.28(0.22, 0.04)	0.45(12.0)	0.52(0.16)	0.49(12.0, 0.09)	0.52(0.06, **0.00**)
17	0.29(11.0)	0.29(**0.00**)	0.35(13.0, 0.21)	0.30(-0.14, 0.03)	0.38(10.0)	0.43(0.13)	0.47(13.0, 0.24)	0.46(-0.02, 0.07)
18	0.16(10.0)	0.14(-0.13)	0.12(13.0, -0.25)	0.14(0.17, **0.00**)	0.21(14.0)	0.30(0.43)	0.30(13.0, 0.43)	0.31(0.03, 0.03)
19	0.29(12.0)	0.30(0.03)	0.32(15.0, 0.10)	0.30(-0.06, **0.00**)	0.21(14.0)	0.22(0.05)	0.26(12.0, 0.24)	0.23(-0.12, 0.05)
20	0.30(9.0)	0.42(0.40)	0.35(13.0, 0.17)	0.43(0.23, 0.02)	0.40(13.0)	0.43(0.07)	0.34(13.0, -0.15)	0.44(0.29, 0.02)
21	0.31(10.0)	0.33(0.06)	0.30(14.0, **-0.03**)	0.35(0.17, 0.06)	0.24(13.0)	0.32(0.33)	0.33(12.0, 0.38)	0.34(0.03, 0.06)
22	0.29(10.0)	0.35(0.21)	0.31(17.0, 0.07)	0.34(0.10, -0.03)	0.24(13.0)	0.29(0.21)	0.22(12.0, -0.08)	0.30(0.36, 0.03)
23	0.16(9.0)	0.21(0.31)	0.16(14.0, **0.00**)	0.20(0.25, -0.05)	0.13(14.0)	0.15(0.15)	0.23(12.0, 0.77)	0.18(-0.22, 0.20)
24	0.18(11.0)	0.27(0.50)	0.17(15.0, **-0.06**)	0.25(0.47, -0.07)	0.42(12.0)	0.45(0.07)	0.45(12.0, 0.07)	0.45(**0.00**, **0.00**)
25	0.35(12.0)	0.42(0.20)	0.41(15.0, 0.17)	0.42(0.02, **0.00**)	0.41(14.0)	0.47(0.15)	0.40(13.0, **-0.02**)	0.47(0.18, **0.00**)
Mean	0.27(10.4)	0.31(0.15)	0.29(14.0, 0.09)	0.31(0.07, 0.00)	0.32(13.0)	0.36(0.13)	0.34(12.1, 0.08)	0.37(0.09, 0.04)

[a] In parentheses is the number of QTL identified by QP based on the SE model; [b] In parentheses is the gain in prediction accuracy with GWP over QP based on the SE model; [c] The first value in parentheses is the number of QTL identified by QP based on the ME model; and the second one the gain

with ME over SE for QP; [d] The first value in parentheses is the gain in accuracy with GWP over QP based on the ME model; and the second one is the gain with ME over SE using GWP. Bold in parentheses indicates the number is not significant at $\alpha = 0.05$.

Table S12 Accuracy of AP prediction for environment E2 with QP and GWP in CV1

	LL				LW			
	SE		ME		SE		ME	
PopId	QP[a]	GWP[b]	QP[c]	GWP[d]	QP[a]	GWP[b]	QP[c]	GWP[d]
1	0.27(12.0)	0.39(0.44)	0.34(14.0, 0.26)	0.38(0.12, -0.03)	0.13(11.0)	0.21(0.62)	0.22(12.0, 0.69)	0.24(0.09, 0.14)
2	0.27(13.0)	0.28(0.04)	0.26(14.0, -0.04)	0.29(0.12, 0.04)	0.31(11.0)	0.44(0.42)	0.35(12.0, 0.13)	0.45(0.29, 0.02)
3	0.21(15.0)	0.29(0.38)	0.28(15.0, 0.33)	0.28(**0.00**, -0.03)	0.42(15.0)	0.54(0.29)	0.51(11.0, 0.21)	0.54(0.06, **0.00**)
4	0.32(14.0)	0.38(0.19)	0.34(12.0, 0.06)	0.39(0.15, 0.03)	0.31(13.0)	0.34(0.10)	0.28(12.0, -0.10)	0.36(0.29, 0.06)
5	0.27(12.0)	0.33(0.22)	0.25(14.0, -0.07)	0.32(0.28, -0.03)	0.37(12.0)	0.47(0.27)	0.38(12.0, **0.03**)	0.48(0.26, 0.02)
6	0.42(14.0)	0.47(0.12)	0.42(13.0, **0.00**)	0.47(0.12, **0.00**)	0.30(13.0)	0.28(-0.07)	0.32(12.0, 0.07)	0.30(-0.06, 0.07)
7	0.21(12.0)	0.20(-0.05)	0.19(14.0, -0.10)	0.21(0.11, 0.05)	0.50(11.0)	0.60(0.20)	0.47(12.0, -0.06)	0.59(0.26, -0.02)
8	0.34(13.0)	0.25(-0.26)	0.34(15.0, **0.00**)	0.26(-0.24, 0.04)	0.29(13.0)	0.34(0.17)	0.30(12.0, 0.03)	0.35(0.17, 0.03)
9	0.23(13.0)	0.23(**0.00**)	0.28(13.0, 0.22)	0.22(-0.21, -0.04)	0.31(13.0)	0.34(0.10)	0.33(13.0, 0.06)	0.34(0.03, **0.00**)
10	0.42(13.0)	0.47(0.12)	0.43(13.0, 0.02)	0.47(0.09, **0.00**)	0.33(14.0)	0.41(0.24)	0.42(13.0, 0.27)	0.45(0.07, 0.10)
11	0.24(15.0)	0.29(0.21)	0.28(15.0, 0.17)	0.30(0.07, 0.03)	0.34(13.0)	0.37(0.09)	0.36(11.0, 0.06)	0.37(0.03, **0.00**)
12	0.36(13.0)	0.41(0.14)	0.41(15.0, 0.14)	0.41(**0.00**, **0.00**)	0.51(13.0)	0.62(0.22)	0.55(11.0, 0.08)	0.63(0.15, 0.02)
13	0.37(12.0)	0.35(-0.05)	0.28(13.0, -0.24)	0.35(0.25, **0.00**)	0.42(13.0)	0.41(-0.02)	0.42(12.0, **0.00**)	0.4(-0.05, -0.02)
14	0.31(13.0)	0.24(-0.23)	0.26(12.0, -0.16)	0.26(**0.00**, 0.08)	0.37(13.0)	0.37(**0.00**)	0.38(12.0, 0.03)	0.39(0.03, 0.05)
15	0.24(13.0)	0.30(0.25)	0.20(15.0, -0.17)	0.29(0.45, -0.03)	0.46(12.0)	0.53(0.15)	0.48(12.0, 0.04)	0.52(0.08, -0.02)
16	0.33(12.0)	0.42(0.27)	0.27(13.0, -0.18)	0.42(0.56, **0.00**)	0.47(13.0)	0.53(0.13)	0.50(12.0, 0.06)	0.54(0.08, 0.02)
17	0.07(16.0)	0.11(0.57)	0.12(13.0, 0.71)	0.12(**0.00**, 0.09)	0.57(12.0)	0.60(0.05)	0.58(13.0, 0.02)	0.59(0.02, -0.02)
18	0.13(14.0)	0.20(0.54)	0.09(13.0, -0.31)	0.19(1.11, -0.05)	0.21(13.0)	0.23(0.10)	0.22(13.0, **0.05**)	0.22(**0.00**, -0.04)
19	0.36(12.0)	0.39(0.08)	0.43(15.0, 0.19)	0.40(-0.07, 0.03)	0.40(12.0)	0.40(**0.00**)	0.39(12.0, **-0.03**)	0.39(**0.00**, -0.03)
20	0.27(12.0)	0.41(0.52)	0.30(13.0, 0.11)	0.41(0.37, **0.00**)	0.39(13.0)	0.44(0.13)	0.32(13.0, -0.18)	0.43(0.34, -0.02)
21	0.43(12.0)	0.47(0.09)	0.37(14.0, -0.14)	0.47(0.27, **0.00**)	0.40(12.0)	0.45(0.13)	0.40(12.0, **0.00**)	0.45(0.13, **0.00**)
22	0.41(13.0)	0.43(0.05)	0.37(17.0, -0.10)	0.43(0.16, **0.00**)	0.37(14.0)	0.40(0.08)	0.39(12.0, 0.05)	0.42(0.08, 0.05)
23	0.28(13.0)	0.35(0.25)	0.25(14.0, -0.11)	0.35(0.40, **0.00**)	0.38(12.0)	0.38(**0.00**)	0.40(12.0, 0.05)	0.40(**0.00**, 0.05)
24	0.15(14.0)	0.21(0.40)	0.20(15.0, 0.33)	0.21(0.05, **0.00**)	0.42(15.0)	0.45(0.07)	0.46(12.0, 0.10)	0.45(-0.02, **0.00**)
25	0.18(13.0)	0.24(0.33)	0.18(15.0, **0.00**)	0.24(0.33, **0.00**)	0.25(13.0)	0.35(0.40)	0.31(13.0, 0.24)	0.37(0.19, 0.06)
Mean	0.28(13.1)	0.32(0.14)	0.29(14.0, 0.01)	0.33(0.14, 0.01)	0.37(12.8)	0.42(0.14)	0.39(12.1, 0.06)	0.43(0.10, 0.02)

[a] In parentheses is the number of QTL identified by QP based on the SE model; [b] In parentheses is the gain in prediction accuracy with GWP over QP based on the SE model; [c] The first value in parentheses is the number of QTL identified by QP based on the ME model; and the second one the gain

with ME over SE for QP; [d] The first value in parentheses is the gain in accuracy with GWP over QP based on the ME model; and the second one is the gain with ME over SE using GWP. Bold in parentheses indicates the number is not significant at $\alpha = 0.05$.

Table S13 Accuracy of AP prediction for environment E3 with QP and GWP in CV1

	LL				LW			
	SE		ME		SE		ME	
PopId	QP[a]	GWP[b]	QP[c]	GWP[d]	QP[a]	GWP[b]	QP[c]	GWP[d]
1	0.31(12.0)	0.37(0.19)	0.25(14.0, -0.19)	0.37(0.48, **0.00**)	0.24(10.0)	0.24(**0.00**)	0.27(12.0, 0.13)	0.25(-0.07, 0.04)
2	0.26(13.0)	0.27(0.04)	0.27(14.0, 0.04)	0.30(0.11, 0.11)	0.38(12.0)	0.46(0.21)	0.43(12.0, 0.13)	0.47(0.09, 0.02)
3	0.15(14.0)	0.22(0.47)	0.13(15.0, -0.13)	0.21(0.62, -0.05)	0.31(11.0)	0.37(0.19)	0.34(11.0, 0.10)	0.36(0.06, -0.03)
4	0.30(12.0)	0.42(0.40)	0.34(12.0, 0.13)	0.41(0.21, -0.02)	0.22(11.0)	0.22(**0.00**)	0.18(12.0, -0.18)	0.24(0.33, 0.09)
5	0.22(13.0)	0.20(-0.09)	0.17(14.0, -0.23)	0.21(0.24, 0.05)	0.37(10.0)	0.44(0.19)	0.37(12.0, **0.00**)	0.43(0.16, -0.02)
6	0.41(13.0)	0.47(0.15)	0.40(13.0, -0.02)	0.46(0.15, -0.02)	0.21(9.0)	0.26(0.24)	0.26(12.0, 0.24)	0.26(**0.00**, **0.00**)
7	0.21(13.0)	0.20(-0.05)	0.18(14.0, -0.14)	0.20(0.11, **0.00**)	0.21(8.0)	0.32(0.52)	0.27(12.0, 0.29)	0.33(0.22, 0.03)
8	0.27(14.0)	0.29(0.07)	0.35(15.0, 0.30)	0.29(-0.17, **0.00**)	0.23(11.0)	0.27(0.17)	0.26(12.0, 0.13)	0.28(0.08, 0.04)
9	0.25(13.0)	0.35(0.40)	0.34(13.0, 0.36)	0.36(0.06, 0.03)	0.31(10.0)	0.31(**0.00**)	0.32(13.0, 0.03)	0.30(-0.06, -0.03)
10	0.36(12.0)	0.47(0.31)	0.35(13.0, **-0.03**)	0.46(0.31, -0.02)	0.41(9.0)	0.56(0.37)	0.38(13.0, -0.07)	0.55(0.45, -0.02)
11	0.16(13.0)	0.22(0.38)	0.11(15.0, -0.31)	0.23(1.09, 0.05)	0.17(10.0)	0.26(0.53)	0.19(11.0, 0.12)	0.27(0.42, 0.04)
12	0.20(13.0)	0.32(0.60)	0.24(15.0, 0.20)	0.31(0.29, -0.03)	0.22(12.0)	0.34(0.55)	0.31(11.0, 0.41)	0.34(0.10, **0.00**)
13	0.39(13.0)	0.36(-0.08)	0.40(13.0, **0.03**)	0.39(-0.03, 0.08)	0.45(10.0)	0.4(-0.11)	0.45(12.0, **0.00**)	0.38(-0.16, -0.05)
14	0.19(13.0)	0.24(0.26)	0.17(12.0, -0.11)	0.24(0.41, **0.00**)	0.24(10.0)	0.26(0.08)	0.30(12.0, 0.25)	0.28(-0.07, 0.08)
15	0.20(13.0)	0.24(0.20)	0.18(15.0, -0.10)	0.25(0.39, 0.04)	0.44(9.0)	0.49(0.11)	0.45(12.0, 0.02)	0.50(0.11, 0.02)
16	0.16(12.0)	0.16(**0.00**)	0.16(13.0, **0.00**)	0.17(0.06, 0.06)	0.38(11.0)	0.46(0.21)	0.43(12.0, 0.13)	0.49(0.14, 0.07)
17	0.26(14.0)	0.29(0.12)	0.29(13.0, 0.12)	0.29(**0.00**, **0.00**)	0.26(10.0)	0.30(0.15)	0.28(13.0, 0.08)	0.33(0.18, 0.10)
18	0.06(13.0)	0.11(0.83)	0.17(13.0, 1.83)	0.13(-0.24, 0.18)	0.29(9.0)	0.32(0.10)	0.34(13.0, 0.17)	0.33(-0.03, 0.03)
19	0.39(13.0)	0.36(-0.08)	0.39(15.0, **0.00**)	0.35(-0.10, -0.03)	0.36(10.0)	0.29(-0.19)	0.35(12.0, -0.03)	0.30(-0.14, 0.03)
20	0.23(12.0)	0.36(0.57)	0.24(13.0, **0.04**)	0.38(0.58, 0.06)	0.39(12.0)	0.44(0.13)	0.41(13.0, 0.05)	0.46(0.12, 0.05)
21	0.26(13.0)	0.44(0.69)	0.32(14.0, 0.23)	0.46(0.44, 0.05)	0.38(9.0)	0.40(0.05)	0.40(12.0, 0.05)	0.41(0.02, 0.02)
22	0.22(13.0)	0.20(-0.09)	0.30(17.0, 0.36)	0.21(-0.30, 0.05)	0.20(11.0)	0.26(0.30)	0.25(12.0, 0.25)	0.26(0.04, **0.00**)
23	0.19(13.0)	0.22(0.16)	0.21(14.0, 0.11)	0.23(0.10, 0.05)	0.19(10.0)	0.27(0.42)	0.23(12.0, 0.21)	0.28(0.22, 0.04)
24	0.13(11.0)	0.19(0.46)	0.14(15.0, **0.08**)	0.19(0.36, **0.00**)	0.39(11.0)	0.36(-0.08)	0.41(12.0, 0.05)	0.38(-0.07, 0.06)
25	0.30(13.0)	0.39(0.30)	0.25(15.0, -0.17)	0.38(0.52, -0.03)	0.20(12.0)	0.24(0.20)	0.24(13.0, 0.20)	0.24(**0.00**, **0.00**)
Mean	0.24(12.8)	0.29(0.21)	0.25(14.0, 0.04)	0.30(0.20, 0.01)	0.30(10.3)	0.34(0.13)	0.32(12.1, 0.09)	0.35(0.09, 0.03)

[a] In parentheses is the number of QTL identified by QP based on the SE model; [b] In parentheses is the gain in prediction accuracy with GWP over QP based on the SE model; [c] The first value in parentheses is the number of QTL identified by QP based on the ME model; and the second one the gain

with ME over SE for QP; [d] The first value in parentheses is the gain in accuracy with GWP over QP based on the ME model; and the second one is the gain with ME over SE using GWP. Bold in parentheses indicates the number is not significant at $\alpha = 0.05$.

Table S14 Accuracy of AP prediction for environment E4 with QP and GWP in CV1

	LL				LW			
	SE		ME		SE		ME	
PopId	QP[a]	GWP[b]	QP[c]	GWP[d]	QP[a]	GWP[b]	QP[c]	GWP[d]
1	0.25(15.0)	0.26(0.04)	0.18(14.0, -0.28)	0.27(0.5, 0.04)	0.26(13.0)	0.31(0.19)	0.30(12.0, 0.15)	0.31(0.03, **0.00**)
2	0.28(15.0)	0.25(-0.11)	0.19(14.0, -0.32)	0.23(0.21, -0.08)	0.39(13.0)	0.43(0.10)	0.39(12.0, **0.00**)	0.43(0.10, **0.00**)
3	0.25(17.0)	0.24(-0.04)	0.15(15.0, -0.40)	0.25(0.67, 0.04)	0.38(12.0)	0.39(0.03)	0.35(11.0, -0.08)	0.40(0.14, 0.03)
4	0.40(13.0)	0.42(0.05)	0.36(12.0, -0.10)	0.43(0.19, 0.02)	0.25(13.0)	0.29(0.16)	0.26(12.0, **0.04**)	0.29(0.12, **0.00**)
5	0.23(15.0)	0.38(0.65)	0.23(14.0, **0.00**)	0.38(0.65, **0.00**)	0.31(13.0)	0.33(0.06)	0.29(12.0, -0.06)	0.36(0.24, 0.09)
6	0.35(15.0)	0.46(0.31)	0.42(13.0, 0.20)	0.48(0.14, 0.04)	0.27(13.0)	0.33(0.22)	0.29(12.0, 0.07)	0.33(0.14, **0.00**)
7	0.16(13.0)	0.32(1.00)	0.19(14.0, 0.19)	0.31(0.63, -0.03)	0.45(12.0)	0.52(0.16)	0.47(12.0, 0.04)	0.53(0.13, 0.02)
8	0.33(14.0)	0.34(0.03)	0.38(15.0, 0.15)	0.35(-0.08, 0.03)	0.26(12.0)	0.39(0.50)	0.30(12.0, 0.15)	0.40(0.33, 0.03)
9	0.34(15.0)	0.35(0.03)	0.32(13.0, -0.06)	0.36(0.13, 0.03)	0.34(13.0)	0.40(0.18)	0.35(13.0, 0.03)	0.41(0.17, 0.02)
10	0.40(16.0)	0.46(0.15)	0.34(13.0, -0.15)	0.48(0.41, 0.04)	0.47(13.0)	0.53(0.13)	0.47(13.0, **0.00**)	0.54(0.15, 0.02)
11	0.22(13.0)	0.27(0.23)	0.17(15.0, -0.23)	0.27(0.59, **0.00**)	0.35(12.0)	0.36(0.03)	0.34(11.0, -0.03)	0.36(0.06, **0.00**)
12	0.31(12.0)	0.31(0.00)	0.34(15.0, 0.10)	0.34(**0.00**, 0.10)	0.56(12.0)	0.57(0.02)	0.54(11.0, -0.04)	0.58(0.07, 0.02)
13	0.25(14.0)	0.16(-0.36)	0.20(13.0, -0.20)	0.16(-0.20, **0.00**)	0.32(13.0)	0.32(0.00)	0.37(12.0, 0.16)	0.35(-0.05, 0.09)
14	0.29(13.0)	0.35(0.21)	0.18(12.0, -0.38)	0.33(0.83, -0.06)	0.32(13.0)	0.33(0.03)	0.36(12.0, 0.12)	0.33(-0.08, **0.00**)
15	0.10(12.0)	0.20(1.00)	0.15(15.0, 0.50)	0.21(0.40, 0.05)	0.58(13.0)	0.60(0.03)	0.57(12.0, -0.02)	0.60(0.05, **0.00**)
16	0.17(12.0)	0.23(0.35)	0.15(13.0, -0.12)	0.24(0.60, 0.04)	0.47(12.0)	0.57(0.21)	0.52(12.0, 0.11)	0.58(0.12, 0.02)
17	0.15(16.0)	0.25(0.67)	0.12(13.0, -0.20)	0.24(1.00, -0.04)	0.55(13.0)	0.53(-0.04)	0.53(13.0, -0.04)	0.53(**0.00**, **0.00**)
18	0.13(14.0)	0.13(**0.00**)	0.16(13.0, 0.23)	0.13(-0.19, **0.00**)	0.40(13.0)	0.38(-0.05)	0.39(13.0, -0.03)	0.38(-0.03, **0.00**)
19	0.21(14.0)	0.19(-0.10)	0.22(15.0, **0.05**)	0.19(-0.14, **0.00**)	0.38(13.0)	0.36(-0.05)	0.43(12.0, 0.13)	0.36(-0.16, **0.00**)
20	0.30(12.0)	0.41(0.37)	0.33(13.0, 0.10)	0.41(0.24, **0.00**)	0.44(12.0)	0.52(0.18)	0.42(13.0, -0.05)	0.52(0.24, **0.00**)
21	0.45(14.0)	0.52(0.16)	0.42(14.0, -0.07)	0.52(0.24, **0.00**)	0.37(13.0)	0.41(0.11)	0.43(12.0, 0.16)	0.44(0.02, 0.07)
22	0.20(14.0)	0.25(0.25)	0.21(17.0, **0.05**)	0.24(0.14, -0.04)	0.31(12.0)	0.40(0.29)	0.31(12.0, **0.00**)	0.40(0.29, **0.00**)
23	0.13(17.0)	0.13(**0.00**)	0.10(14.0, -0.23)	0.13(0.30, **0.00**)	0.29(13.0)	0.30(0.03)	0.32(12.0, 0.10)	0.30(-0.06, **0.00**)
24	0.18(14.0)	0.29(0.61)	0.14(15.0, -0.22)	0.28(1.00, -0.03)	0.48(13.0)	0.50(0.04)	0.53(12.0, 0.10)	0.50(-0.06, **0.00**)
25	0.19(11.0)	0.29(0.53)	0.18(15.0, -0.05)	0.30(0.67, 0.03)	0.36(13.0)	0.44(0.22)	0.32(13.0, -0.11)	0.43(0.34, -0.02)
Mean	0.25(14.0)	0.30(0.20)	0.23(14.0, -0.07)	0.30(0.30, 0.00)	0.38(12.7)	0.42(0.11)	0.39(12.1, 0.03)	0.43(0.10, 0.01)

[a] In parentheses is the number of QTL identified by QP based on the SE model; [b] In parentheses is the gain in prediction accuracy with GWP over QP based on the SE model; [c] The first value in parentheses is the number of QTL identified by QP based on the ME model; and the second one the gain

with ME over SE for QP; [d] The first value in parentheses is the gain in accuracy with GWP over QP based on the ME model; and the second one is the gain with ME over SE using GWP. Bold in parentheses indicates the number is not significant at $\alpha = 0.05$.

Table S15 Accuracy of WP prediction for environment E1 with QP and GWP in CV2

	LL				LW			
	SE		ME		SE		ME	
PopId	QP[a]	GWP[b]	QP[c]	GWP[d]	QP[a]	GWP[b]	QP[c]	GWP[d]
1	0.23(1.6)	0.33(0.43)	0.41(3.4, 0.78)	0.45(0.10, 0.36)	0.06(1.3)	0.31(4.17)	0.18(1.6, 2.00)	0.49(1.72, 0.58)
2	0.03(1.1)	0.18(5.00)	0.06(0.8, 1.00)	0.42(6.00, 1.33)	0.21(2.0)	0.40(0.90)	0.24(1.8, **0.14**)	0.55(1.29, 0.38)
3	0.09(1.2)	0.26(1.89)	0.06(0.8, **-0.33**)	0.37(5.17, 0.42)	0.39(3.3)	0.56(0.44)	0.43(2.6, 0.10)	0.68(0.58, 0.21)
4	0.24(2.8)	0.49(1.04)	0.34(3.2, 0.42)	0.60(0.76, 0.22)	0.23(2.4)	0.50(1.17)	0.23(1.6, **0.00**)	0.61(1.65, 0.22)
5	0.14(1.8)	0.33(1.36)	0.31(1.9, 1.21)	0.54(0.74, 0.64)	0.06(1.3)	0.21(2.50)	0.10(0.9, 0.67)	0.46(3.60, 1.19)
6	0.25(2.4)	0.50(1.00)	0.31(2.3, 0.24)	0.59(0.90, 0.18)	0.02(1.6)	0.31(14.50)	0.05(1.0, 1.50)	0.43(7.60, 0.39)
7	0.34(2.6)	0.52(0.53)	0.38(1.9, 0.12)	0.67(0.76, 0.29)	0.23(2.5)	0.48(1.09)	0.29(1.9, 0.26)	0.61(1.10, 0.27)
8	0.07(1.7)	0.28(3.00)	0.19(1.7, 1.71)	0.51(1.68, 0.82)	0.07(1.5)	0.30(3.29)	0.28(2.2, 3.00)	0.49(0.75, 0.63)
9	0.30(2.1)	0.35(0.17)	0.31(1.7, **0.03**)	0.50(0.61, 0.43)	0.02(1.0)	0.18(8.00)	0.21(1.7, 9.50)	0.42(1.00, 1.33)
10	0.26(2.5)	0.46(0.77)	0.32(2.1, 0.23)	0.64(1.00, 0.39)	0.15(2.2)	0.37(1.47)	0.27(2.4, 0.80)	0.62(1.30, 0.68)
11	0.30(2.5)	0.40(0.33)	0.41(2.6, 0.37)	0.56(0.37, 0.40)	0.12(2.3)	0.40(2.33)	0.16(1.7, 0.33)	0.48(2.00, 0.20)
12	0.25(3.0)	0.50(1.00)	0.33(2.9, 0.32)	0.65(0.97, 0.30)	0.30(3.1)	0.54(0.80)	0.40(2.8, 0.33)	0.64(0.60, 0.19)
13	0.21(2.7)	0.45(1.14)	0.27(1.6, 0.29)	0.64(1.37, 0.42)	0.30(2.6)	0.49(0.63)	0.33(1.7, **0.10**)	0.61(0.85, 0.24)
14	0.10(2.0)	0.36(2.60)	0.21(1.6, 1.10)	0.55(1.62, 0.53)	0.20(2.3)	0.44(1.20)	0.22(1.4, **0.10**)	0.62(1.82, 0.41)
15	0.08(1.7)	0.28(2.50)	0.17(1.4, 1.12)	0.47(1.76, 0.68)	0.13(1.7)	0.30(1.31)	0.25(2.5, 0.92)	0.43(0.72, 0.43)
16	0.20(1.7)	0.34(0.70)	0.23(1.2, 0.15)	0.48(1.09, 0.41)	0.25(2.3)	0.45(0.80)	0.38(2.7, 0.52)	0.56(0.47, 0.24)
17	0.05(1.2)	0.27(4.40)	0.09(0.8, 0.80)	0.39(3.33, 0.44)	0.32(3.0)	0.55(0.72)	0.43(3.5, 0.34)	0.68(0.58, 0.24)
18	0.10(1.4)	0.27(1.70)	0.06(0.6, -0.40)	0.36(5.00, 0.33)	0.19(2.0)	0.40(1.11)	0.22(1.6, **0.16**)	0.47(1.14, 0.17)
19	0.27(2.2)	0.37(0.37)	0.21(1.5, -0.22)	0.52(1.48, 0.41)	0.28(2.3)	0.41(0.46)	0.37(2.1, 0.32)	0.58(0.57, 0.41)
20	0.19(2.6)	0.47(1.47)	0.16(1.5, **-0.16**)	0.61(2.81, 0.30)	0.23(2.3)	0.48(1.09)	0.34(2.2, 0.48)	0.60(0.76, 0.25)
21	0.33(3.0)	0.48(0.45)	0.42(3.3, 0.27)	0.61(0.45, 0.27)	0.18(2.1)	0.47(1.61)	0.16(1.7, **-0.11**)	0.54(2.38, 0.15)
22	0.15(1.3)	0.36(1.40)	0.25(1.4, 0.67)	0.51(1.04, 0.42)	0.05(1.4)	0.31(5.20)	0.18(1.6, 2.60)	0.47(1.61, 0.52)
23	0.32(3.0)	0.44(0.38)	0.27(2.0, -0.16)	0.54(1.00, 0.23)	0.08(1.5)	0.30(2.75)	0.25(2.4, 2.12)	0.54(1.16, 0.80)
24	0.20(2.3)	0.48(1.40)	0.12(1.1, -0.40)	0.57(3.75, 0.19)	0.35(3.2)	0.50(0.43)	0.45(2.7, 0.29)	0.64(0.42, 0.28)
25	0.18(2.6)	0.50(1.78)	0.20(1.8, 0.11)	0.61(2.05, 0.22)	0.31(3.1)	0.47(0.52)	0.32(2.2, **0.03**)	0.60(0.87, 0.28)
Mean	0.20(2.1)	0.39(0.98)	0.24(1.8, 0.25)	0.53(1.19, 0.38)	0.19(2.2)	0.41(1.14)	0.27(2.0, 0.42)	0.55(1.05, 0.36)

[a] In parentheses is the number of QTL identified by QP based on the SE model; [b] In parentheses is the gain in prediction accuracy with GWP over QP based on the SE model; [c] The first value in parentheses is the number of QTL identified by QP based on the ME model; and the second one the gain

with ME over SE for QP; [d] The first value in parentheses is the gain in accuracy with GWP over QP based on the ME model; and the second one is the gain in accuracy with ME over SE using GWP. Bold in parentheses indicates the number is not significant at $\alpha = 0.05$.

Table S16 Accuracy of WP prediction for environment E2 with QP and GWP in CV2

	LL				LW			
	SE		ME		SE		ME	
PopId	QP[a]	GWP[b]	QP[c]	GWP[d]	QP[a]	GWP[b]	QP[c]	GWP[d]
1	0.50(3.8)	0.67(0.34)	0.58(3.4, 0.16)	0.75(0.29, 0.12)	0.20(2.5)	0.46(1.30)	0.24(1.6, **0.20**)	0.57(1.37, 0.24)
2	0.10(2.1)	0.40(3.00)	0.08(0.8, **-0.20**)	0.56(6.00, 0.40)	0.21(2.6)	0.48(1.29)	0.28(1.8, 0.33)	0.64(1.29, 0.33)
3	0.24(1.7)	0.20(-0.17)	0.08(0.8, -0.67)	0.30(2.75, 0.50)	0.34(3.3)	0.60(0.76)	0.45(2.6, 0.32)	0.70(0.56, 0.17)
4	0.22(3.0)	0.56(1.55)	0.35(3.2, 0.59)	0.68(0.94, 0.21)	0.41(3.1)	0.61(0.49)	0.32(1.6, -0.22)	0.70(1.19, 0.15)
5	0.27(2.8)	0.50(0.85)	0.31(1.9, 0.15)	0.64(1.06, 0.28)	0.08(2.3)	0.46(4.75)	0.15(0.9, 0.87)	0.64(3.27, 0.39)
6	0.22(2.8)	0.47(1.14)	0.25(2.3, **0.14**)	0.58(1.32, 0.23)	0.06(1.2)	0.31(4.17)	0.09(1.0, **0.50**)	0.37(3.11, 0.19)
7	0.27(2.1)	0.41(0.52)	0.33(1.9, 0.22)	0.62(0.88, 0.51)	0.34(2.9)	0.59(0.74)	0.35(1.9, **0.03**)	0.72(1.06, 0.22)
8	0.10(2.0)	0.34(2.40)	0.21(1.7, 1.10)	0.51(1.43, 0.50)	0.24(1.8)	0.36(0.50)	0.35(2.2, 0.46)	0.53(0.51, 0.47)
9	0.10(1.9)	0.33(2.30)	0.18(1.7, 0.80)	0.46(1.56, 0.39)	0.23(2.3)	0.47(1.04)	0.34(1.7, 0.48)	0.54(0.59, 0.15)
10	0.22(2.9)	0.51(1.32)	0.31(2.1, 0.41)	0.65(1.10, 0.27)	0.13(1.9)	0.36(1.77)	0.26(2.4, 1.00)	0.46(0.77, 0.28)
11	0.27(3.0)	0.52(0.93)	0.40(2.6, 0.48)	0.62(0.55, 0.19)	0.29(2.1)	0.41(0.41)	0.29(1.7, **0.00**)	0.54(0.86, 0.32)
12	0.24(3.1)	0.55(1.29)	0.36(2.9, 0.50)	0.68(0.89, 0.24)	0.29(3.6)	0.58(1.00)	0.38(2.8, 0.31)	0.65(0.71, 0.12)
13	0.22(2.3)	0.39(0.77)	0.32(1.6, 0.45)	0.65(1.03, 0.67)	0.18(2.2)	0.42(1.33)	0.31(1.7, 0.72)	0.60(0.94, 0.43)
14	0.32(2.7)	0.42(0.31)	0.25(1.6, -0.22)	0.58(1.32, 0.38)	0.04(1.4)	0.25(5.25)	0.12(1.4, 2.00)	0.45(2.75, 0.80)
15	0.26(3.0)	0.48(0.85)	0.27(1.4, **0.04**)	0.65(1.41, 0.35)	0.23(3.2)	0.55(1.39)	0.32(2.5, 0.39)	0.67(1.09, 0.22)
16	0.19(2.0)	0.33(0.74)	0.21(1.2, **0.11**)	0.46(1.19, 0.39)	0.42(3.3)	0.55(0.31)	0.46(2.7, 0.10)	0.64(0.39, 0.16)
17	0.05(0.8)	0.12(1.40)	0.09(0.8, 0.80)	0.29(2.22, 1.42)	0.41(3.6)	0.61(0.49)	0.47(3.5, 0.15)	0.73(0.55, 0.20)
18	0.02(1.3)	0.27(12.50)	0.07(0.6, 2.50)	0.40(4.71, 0.48)	0.17(1.7)	0.31(0.82)	0.24(1.6, 0.41)	0.50(1.08, 0.61)
19	0.07(1.7)	0.27(2.86)	0.11(1.5, 0.57)	0.40(2.64, 0.48)	0.20(2.2)	0.41(1.05)	0.34(2.1, 0.70)	0.57(0.68, 0.39)
20	0.12(2.3)	0.52(3.33)	0.16(1.5, 0.33)	0.66(3.12, 0.27)	0.32(2.6)	0.54(0.69)	0.40(2.2, 0.25)	0.69(0.72, 0.28)
21	0.35(3.5)	0.58(0.66)	0.48(3.3, 0.37)	0.70(0.46, 0.21)	0.13(1.9)	0.33(1.54)	0.20(1.7, 0.54)	0.44(1.20, 0.33)
22	0.32(2.6)	0.50(0.56)	0.24(1.4, -0.25)	0.60(1.50, 0.20)	0.32(3.0)	0.59(0.84)	0.27(1.6, -0.16)	0.68(1.52, 0.15)
23	0.16(2.3)	0.43(1.69)	0.19(2.0, **0.19**)	0.53(1.79, 0.23)	0.23(2.3)	0.37(0.61)	0.27(2.4, 0.17)	0.49(0.81, 0.32)
24	0.01(1.0)	0.16(15.00)	0.10(1.1, 9.00)	0.44(3.40, 1.75)	0.24(2.2)	0.45(0.88)	0.42(2.7, 0.75)	0.62(0.48, 0.38)
25	0.08(2.0)	0.40(4.00)	0.14(1.8, 0.75)	0.57(3.07, 0.42)	0.29(2.8)	0.48(0.66)	0.37(2.2, 0.28)	0.54(0.46, 0.13)
Mean	0.20(2.3)	0.41(1.10)	0.24(1.8, 0.23)	0.56(1.30, 0.35)	0.24(2.5)	0.46(0.92)	0.31(2.0, 0.28)	0.59(0.91, 0.27)

[a] In parentheses is the number of QTL identified by QP based on the SE model; [b] In parentheses is the gain in prediction accuracy with GWP over QP based on the SE model; [c] The first value in parentheses is the number of QTL identified by QP based on the ME model; and the second one the gain

with ME over SE for QP; [d] The first value in parentheses is the gain in accuracy with GWP over QP based on the ME model; and the second one is the gain in accuracy with ME over SE using GWP. Bold in parentheses indicates the number is not significant at $\alpha = 0.05$.

Table S17 Accuracy of WP prediction for environment E3 with QP and GWP in CV2

	LL				LW			
	SE		ME		SE		ME	
PopId	QP[a]	GWP[b]	QP[c]	GWP[d]	QP[a]	GWP[b]	QP[c]	GWP[d]
1	0.34(2.6)	0.51(0.50)	0.47(3.4, 0.38)	0.65(0.38, 0.27)	0.12(1.7)	0.36(2.00)	0.15(1.6, **0.25**)	0.45(2.00, 0.25)
2	0.12(1.9)	0.36(2.00)	0.08(0.8, -0.33)	0.51(5.38, 0.42)	0.22(2.7)	0.52(1.36)	0.26(1.8, **0.18**)	0.65(1.50, 0.25)
3	0.08(1.5)	0.23(1.88)	0.07(0.8, **-0.12**)	0.35(4.00, 0.52)	0.25(2.0)	0.26(0.04)	0.36(2.6, 0.44)	0.46(0.28, 0.77)
4	0.31(2.6)	0.51(0.65)	0.37(3.2, 0.19)	0.60(0.62, 0.18)	0.01(1.1)	0.19(18.00)	0.13(1.6, 12.00)	0.38(1.92, 1.00)
5	0.23(2.5)	0.46(1.00)	0.29(1.9, 0.26)	0.60(1.07, 0.30)	0.03(1.4)	0.30(9.00)	0.08(0.9, 1.67)	0.52(5.50, 0.73)
6	0.34(2.4)	0.47(0.38)	0.32(2.3, **-0.06**)	0.59(0.84, 0.26)	0.08(1.8)	0.31(2.87)	0.08(1.0, **0.00**)	0.43(4.38, 0.39)
7	0.28(2.3)	0.49(0.75)	0.31(1.9, **0.11**)	0.64(1.06, 0.31)	0.22(2.1)	0.42(0.91)	0.29(1.9, 0.32)	0.59(1.03, 0.40)
8	0.09(1.8)	0.32(2.56)	0.17(1.7, 0.89)	0.50(1.94, 0.56)	0.13(2.4)	0.46(2.54)	0.26(2.2, 1.00)	0.54(1.08, 0.17)
9	0.19(2.1)	0.42(1.21)	0.28(1.7, 0.47)	0.54(0.93, 0.29)	0.18(2.5)	0.45(1.50)	0.31(1.7, 0.72)	0.57(0.84, 0.27)
10	0.10(2.3)	0.42(3.20)	0.27(2.1, 1.70)	0.62(1.30, 0.48)	0.32(3.5)	0.55(0.72)	0.31(2.4, **-0.03**)	0.62(1.00, 0.13)
11	0.30(2.7)	0.53(0.77)	0.39(2.6, 0.30)	0.63(0.62, 0.19)	0.12(2.0)	0.38(2.17)	0.17(1.7, 0.42)	0.52(2.06, 0.37)
12	0.21(2.3)	0.42(1.00)	0.29(2.9, 0.38)	0.56(0.93, 0.33)	0.05(1.4)	0.27(4.40)	0.23(2.8, 3.60)	0.41(0.78, 0.52)
13	0.19(2.6)	0.43(1.26)	0.30(1.6, 0.58)	0.61(1.03, 0.42)	0.06(1.9)	0.27(3.50)	0.26(1.7, 3.33)	0.51(0.96, 0.89)
14	0.19(1.5)	0.33(0.74)	0.17(1.6, **-0.11**)	0.48(1.82, 0.45)	0.09(1.5)	0.33(2.67)	0.18(1.4, 1.00)	0.53(1.94, 0.61)
15	0.05(1.2)	0.25(4.00)	0.17(1.4, 2.40)	0.46(1.71, 0.84)	0.17(2.6)	0.39(1.29)	0.27(2.5, 0.59)	0.57(1.11, 0.46)
16	0.08(1.8)	0.33(3.12)	0.10(1.2, **0.25**)	0.43(3.30, 0.30)	0.09(1.8)	0.36(3.00)	0.31(2.7, 2.44)	0.52(0.68, 0.44)
17	0.10(1.6)	0.32(2.20)	0.12(0.8, **0.20**)	0.44(2.67, 0.38)	0.10(1.8)	0.37(2.70)	0.32(3.5, 2.20)	0.54(0.69, 0.46)
18	0.00(0.9)	0.18	0.05(0.6)	0.28(4.60, 0.56)	0.34(2.2)	0.35(0.03)	0.29(1.6, -0.15)	0.47(0.62, 0.34)
19	0.22(2.6)	0.46(1.09)	0.20(1.5, **-0.09**)	0.57(1.85, 0.24)	0.21(2.0)	0.41(0.95)	0.35(2.1, 0.67)	0.54(0.54, 0.32)
20	0.14(2.1)	0.32(1.29)	0.16(1.5, **0.14**)	0.49(2.06, 0.53)	0.17(2.2)	0.44(1.59)	0.33(2.2, 0.94)	0.58(0.76, 0.32)
21	0.25(2.7)	0.47(0.88)	0.46(3.3, 0.84)	0.63(0.37, 0.34)	0.21(2.4)	0.41(0.95)	0.21(1.7, **0.00**)	0.50(1.38, 0.22)
22	0.23(1.9)	0.39(0.70)	0.28(1.4, 0.22)	0.51(0.82, 0.31)	0.10(1.6)	0.34(2.40)	0.20(1.6, 1.00)	0.52(1.60, 0.53)
23	0.21(1.7)	0.31(0.48)	0.27(2.0, 0.29)	0.50(0.85, 0.61)	0.15(2.1)	0.40(1.67)	0.29(2.4, 0.93)	0.52(0.79, 0.30)
24	0.05(1.5)	0.26(4.20)	0.09(1.1, 0.80)	0.41(3.56, 0.58)	0.37(2.3)	0.41(0.11)	0.47(2.7, 0.27)	0.57(0.21, 0.39)
25	0.10(1.7)	0.36(2.60)	0.17(1.8, 0.70)	0.50(1.94, 0.39)	0.12(1.8)	0.29(1.42)	0.26(2.2, 1.17)	0.44(0.69, 0.52)
Mean	0.18(2.0)	0.38(1.17)	0.23(1.8, 0.33)	0.52(1.24, 0.37)	0.16(2.0)	0.37(1.36)	0.25(2.0, 0.63)	0.52(1.03, 0.40)

[a] In parentheses is the number of QTL identified by QP based on the SE model; [b] In parentheses is the gain in prediction accuracy with GWP over QP based on the SE model; [c] The first value in parentheses is the number of QTL identified by QP based on the ME model; and the second one the gain

with ME over SE for QP; [d] The first value in parentheses is the gain in accuracy with GWP over QP based on the ME model; and the second one is the gain in accuracy with ME over SE using GWP. Bold in parentheses indicates the number is not significant at $\alpha = 0.05$.

Table S18 Accuracy of WP prediction for environment E4 with QP and GWP in CV2

	LL				LW			
	SE		ME		SE		ME	
PopId	QP[a]	GWP[b]	QP[c]	GWP[d]	QP[a]	GWP[b]	QP[c]	GWP[d]
1	0.31(2.4)	0.46(0.48)	0.47(3.4, 0.52)	0.61(0.30, 0.33)	0.25(2.5)	0.51(1.04)	0.25(1.6, **0.00**)	0.62(1.48, 0.22)
2	0.05(2.0)	0.30(5.00)	0.08(0.8, **0.60**)	0.52(5.50, 0.73)	0.20(2.5)	0.46(1.30)	0.25(1.8, 0.25)	0.62(1.48, 0.35)
3	0.08(1.3)	0.27(2.38)	0.06(0.8, **-0.25**)	0.36(5.00, 0.33)	0.29(2.6)	0.41(0.41)	0.40(2.6, 0.38)	0.61(0.52, 0.49)
4	0.31(3.1)	0.53(0.71)	0.43(3.2, 0.39)	0.65(0.51, 0.23)	0.33(2.8)	0.52(0.58)	0.25(1.6, -0.24)	0.63(1.52, 0.21)
5	0.07(1.8)	0.37(4.29)	0.18(1.9, 1.57)	0.55(2.06, 0.49)	0.07(2.2)	0.44(5.29)	0.13(0.9, 0.86)	0.62(3.77, 0.41)
6	0.22(2.7)	0.47(1.14)	0.31(2.3, 0.41)	0.59(0.90, 0.26)	0.08(1.4)	0.31(2.87)	0.10(1.0, **0.25**)	0.46(3.60, 0.48)
7	0.33(2.6)	0.60(0.82)	0.38(1.9, 0.15)	0.73(0.92, 0.22)	0.38(3.2)	0.61(0.61)	0.35(1.9, **-0.08**)	0.72(1.06, 0.18)
8	0.18(2.2)	0.37(1.06)	0.26(1.7, 0.44)	0.59(1.27, 0.59)	0.28(2.3)	0.40(0.43)	0.37(2.2, 0.32)	0.57(0.54, 0.42)
9	0.10(1.9)	0.33(2.30)	0.18(1.7, 0.80)	0.48(1.67, 0.45)	0.37(2.6)	0.48(0.30)	0.39(1.7, **0.05**)	0.51(0.31, 0.06)
10	0.13(2.2)	0.42(2.23)	0.25(2.1, 0.92)	0.57(1.28, 0.36)	0.26(3.3)	0.62(1.38)	0.32(2.4, 0.23)	0.72(1.25, 0.16)
11	0.16(2.1)	0.29(0.81)	0.37(2.6, 1.31)	0.49(0.32, 0.69)	0.18(2.6)	0.46(1.56)	0.24(1.7, 0.33)	0.60(1.50, 0.30)
12	0.15(2.7)	0.50(2.33)	0.32(2.9, 1.13)	0.64(1.00, 0.28)	0.38(3.4)	0.57(0.50)	0.45(2.8, 0.18)	0.66(0.47, 0.16)
13	0.10(1.3)	0.24(1.40)	0.30(1.6, 2.00)	0.54(0.80, 1.25)	0.16(2.1)	0.42(1.62)	0.28(1.7, 0.75)	0.60(1.14, 0.43)
14	0.06(1.9)	0.35(4.83)	0.19(1.6, 2.17)	0.53(1.79, 0.51)	0.24(2.3)	0.44(0.83)	0.23(1.4, **-0.04**)	0.63(1.74, 0.43)
15	0.21(1.9)	0.37(0.76)	0.23(1.4, **0.10**)	0.57(1.48, 0.54)	0.29(3.0)	0.54(0.86)	0.35(2.5, 0.21)	0.68(0.94, 0.26)
16	0.08(1.3)	0.21(1.62)	0.19(1.2, 1.38)	0.42(1.21, 1.00)	0.30(3.0)	0.54(0.80)	0.43(2.7, 0.43)	0.66(0.53, 0.22)
17	0.12(1.6)	0.35(1.92)	0.13(0.8, **0.08**)	0.48(2.69, 0.37)	0.31(3.3)	0.56(0.81)	0.42(3.5, 0.35)	0.70(0.67, 0.25)
18	0.04(1.3)	0.26(5.50)	0.06(0.6, **0.50**)	0.37(5.17, 0.42)	0.22(2.0)	0.33(0.50)	0.28(1.6, 0.27)	0.45(0.61, 0.36)
19	0.06(1.5)	0.28(3.67)	0.13(1.5, 1.17)	0.45(2.46, 0.61)	0.42(3.4)	0.50(0.19)	0.42(2.1, **0.00**)	0.66(0.57, 0.32)
20	0.11(2.2)	0.47(3.27)	0.14(1.5, 0.27)	0.59(3.21, 0.26)	0.49(2.6)	0.53(0.08)	0.45(2.2, -0.08)	0.67(0.49, 0.26)
21	0.38(3.4)	0.61(0.61)	0.49(3.3, 0.29)	0.69(0.41, 0.13)	0.10(1.8)	0.36(2.60)	0.18(1.7, 0.80)	0.49(1.72, 0.36)
22	0.30(2.3)	0.46(0.53)	0.28(1.4, **-0.07**)	0.55(0.96, 0.20)	0.25(2.8)	0.54(1.16)	0.26(1.6, **0.04**)	0.67(1.58, 0.24)
23	0.26(2.0)	0.39(0.50)	0.29(2.0, 0.12)	0.50(0.72, 0.28)	0.29(3.0)	0.48(0.66)	0.32(2.4, **0.10**)	0.60(0.87, 0.25)
24	0.19(1.9)	0.23(0.21)	0.13(1.1, -0.32)	0.41(2.15, 0.78)	0.31(3.1)	0.46(0.48)	0.44(2.7, 0.42)	0.62(0.41, 0.35)
25	0.02(1.5)	0.24(11.00)	0.12(1.8, 5.00)	0.45(2.75, 0.88)	0.27(2.5)	0.49(0.81)	0.38(2.2, 0.41)	0.54(0.42, 0.10)
Mean	0.16(2.0)	0.38(1.38)	0.24(1.8, 0.49)	0.53(1.23, 0.39)	0.27(2.7)	0.48(0.78)	0.32(2.0, 0.18)	0.61(0.93, 0.28)

[a] In parentheses is the number of QTL identified by QP based on the SE model; [b] In parentheses is the gain in prediction accuracy with GWP over QP based on the SE model; [c] The first value in parentheses is the number of QTL identified by QP based on the ME model; and the second one the gain

with ME over SE for QP; [d] The first value in parentheses is the gain in accuracy with GWP over QP based on the ME model; and the second one is the gain in accuracy with ME over SE using GWP. Bold in parentheses indicates the number is not significant at $\alpha = 0.05$.

Table S19 Accuracy of AP prediction for environment E1 with QP and GWP in CV2

	LL				LW			
	SE		ME		SE		ME	
PopId	QP[a]	GWP[b]	QP[c]	GWP[d]	QP[a]	GWP[b]	QP[c]	GWP[d]
1	0.21(8.0)	0.31(0.48)	0.27(9.5, 0.29)	0.35(0.30, 0.13)	0.20(8.5)	0.27(0.35)	0.22(11.0, 0.10)	0.30(0.36, 0.11)
2	0.14(8.0)	0.17(0.21)	0.20(9.5, 0.43)	0.22(0.10, 0.29)	0.37(8.5)	0.41(0.11)	0.37(11.0, **0.00**)	0.44(0.19, 0.07)
3	0.20(8.0)	0.25(0.25)	0.22(9.5, **0.10**)	0.28(0.27, 0.12)	0.39(8.5)	0.55(0.41)	0.46(11.0, 0.18)	0.59(0.28, 0.07)
4	0.43(8.0)	0.45(0.05)	0.40(9.5, -0.07)	0.48(0.20, 0.07)	0.32(8.5)	0.33(0.03)	0.29(11.0, -0.09)	0.33(0.14, **0.00**)
5	0.26(8.0)	0.26(**0.00**)	0.21(9.5, -0.19)	0.28(0.33, 0.08)	0.14(8.5)	0.24(0.71)	0.15(11.0, **0.07**)	0.28(0.87, 0.17)
6	0.39(8.0)	0.47(0.21)	0.39(9.5, **0.00**)	0.51(0.31, 0.09)	0.33(8.5)	0.38(0.15)	0.39(11.0, 0.18)	0.41(0.05, 0.08)
7	0.27(8.0)	0.29(0.07)	0.32(9.5, 0.19)	0.36(0.13, 0.24)	0.33(8.5)	0.37(0.12)	0.39(11.0, 0.18)	0.44(0.13, 0.19)
8	0.36(8.0)	0.44(0.22)	0.40(9.5, 0.11)	0.49(0.23, 0.11)	0.28(8.5)	0.37(0.32)	0.33(11.0, 0.18)	0.40(0.21, 0.08)
9	0.33(8.0)	0.35(0.06)	0.36(9.5, 0.09)	0.39(0.08, 0.11)	0.16(8.5)	0.16(**0.00**)	0.18(11.0, 0.12)	0.18(**0.00**, 0.13)
10	0.32(8.0)	0.39(0.22)	0.39(9.5, 0.22)	0.44(0.13, 0.13)	0.39(8.5)	0.44(0.13)	0.44(11.0, 0.13)	0.50(0.14, 0.14)
11	0.09(8.0)	0.14(0.56)	0.11(9.5, 0.22)	0.20(0.82, 0.43)	0.16(8.5)	0.21(0.31)	0.23(11.0, 0.44)	0.24(0.04, 0.14)
12	0.35(8.0)	0.40(0.14)	0.40(9.5, 0.14)	0.46(0.15, 0.15)	0.50(8.5)	0.57(0.14)	0.53(11.0, 0.06)	0.60(0.13, 0.05)
13	0.25(8.0)	0.28(0.12)	0.38(9.5, 0.52)	0.38(**0.00**, 0.36)	0.41(8.5)	0.40(-0.02)	0.47(11.0, 0.15)	0.44(-0.06, 0.1)
14	0.37(8.0)	0.40(0.08)	0.39(9.5, 0.05)	0.44(0.13, 0.10)	0.37(8.5)	0.36(-0.03)	0.40(11.0, 0.08)	0.42(0.05, 0.17)
15	0.23(8.0)	0.25(0.09)	0.19(9.5, -0.17)	0.27(0.42, 0.08)	0.22(8.5)	0.33(0.50)	0.24(11.0, 0.09)	0.34(0.42, 0.03)
16	0.21(8.0)	0.30(0.43)	0.23(9.5, 0.10)	0.34(0.48, 0.13)	0.42(8.5)	0.51(0.21)	0.50(11.0, 0.19)	0.53(0.06, 0.04)
17	0.29(8.0)	0.29(**0.00**)	0.31(9.5, **0.07**)	0.33(0.06, 0.14)	0.37(8.5)	0.46(0.24)	0.46(11.0, 0.24)	0.51(0.11, 0.11)
18	0.14(8.0)	0.15(0.07)	0.14(9.5, **0.00**)	0.17(0.21, 0.13)	0.21(8.5)	0.30(0.43)	0.29(11.0, 0.38)	0.33(0.14, 0.10)
19	0.29(8.0)	0.32(0.10)	0.34(9.5, 0.17)	0.37(0.09, 0.16)	0.22(8.5)	0.26(0.18)	0.26(11.0, 0.18)	0.29(0.12, 0.12)
20	0.29(8.0)	0.42(0.45)	0.32(9.5, 0.10)	0.45(0.41, 0.07)	0.38(8.5)	0.44(0.16)	0.37(11.0, **-0.03**)	0.47(0.27, 0.07)
21	0.30(8.0)	0.33(0.10)	0.35(9.5, 0.17)	0.39(0.11, 0.18)	0.28(8.5)	0.32(0.14)	0.31(11.0, 0.11)	0.35(0.13, 0.09)
22	0.30(8.0)	0.34(0.13)	0.29(9.5, **-0.03**)	0.36(0.24, 0.06)	0.24(8.5)	0.31(0.29)	0.26(11.0, 0.08)	0.34(0.31, 0.10)
23	0.15(8.0)	0.22(0.47)	0.18(9.5, 0.20)	0.25(0.39, 0.14)	0.15(8.5)	0.17(0.13)	0.24(11.0, 0.60)	0.24(**0.00**, 0.41)
24	0.23(8.0)	0.32(0.39)	0.23(9.5, **0.00**)	0.32(0.39, **0.00**)	0.45(8.5)	0.47(0.04)	0.47(11.0, 0.04)	0.50(0.06, 0.06)
25	0.39(8.0)	0.43(0.10)	0.35(9.5, -0.10)	0.46(0.31, 0.07)	0.38(8.5)	0.48(0.26)	0.40(11.0, 0.05)	0.51(0.28, 0.06)
Mean	0.27(8.0)	0.32(0.19)	0.29(9.5, 0.09)	0.36(0.24, 0.12)	0.31(8.5)	0.36(0.16)	0.35(11.1, 0.13)	0.40(0.14, 0.10)

[a] In parentheses is the number of QTL identified by QP based on the SE model; [b] In parentheses is the gain in prediction accuracy with GWP over QP based on the SE model; [c] The first value in parentheses is the number of QTL identified by QP based on the ME model; and the second one the gain

with ME over SE for QP; [d] The first value in parentheses is the gain in accuracy with GWP over QP based on the ME model; the second one is the gain in accuracy with ME over SE using GWP; and the third one is the gain in accuracy with GWP over PP. Bold in parentheses indicates the number is not significant at $\alpha = 0.05$.

Table S20 Accuracy of AP prediction for environment E2 with QP and GWP in CV2

	LL				LW			
	SE		ME		SE		ME	
PopId	QP[a]	GWP[b]	QP[c]	GWP[d]	QP[a]	GWP[b]	QP[c]	GWP[d]
1	0.33(9.8)	0.45(0.36)	0.30(9.5, -0.09)	0.47(0.57, 0.04)	0.22(10.6)	0.25(0.14)	0.28(11.0, 0.27)	0.30(0.07, 0.20)
2	0.27(9.8)	0.30(0.11)	0.28(9.5, 0.04)	0.36(0.29, 0.20)	0.35(10.6)	0.45(0.29)	0.39(11.0, 0.11)	0.50(0.28, 0.11)
3	0.28(9.8)	0.30(0.07)	0.26(9.5, -0.07)	0.32(0.23, 0.07)	0.47(10.6)	0.56(0.19)	0.52(11.0, 0.11)	0.60(0.15, 0.07)
4	0.33(9.8)	0.42(0.27)	0.34(9.5, **0.03**)	0.46(0.35, 0.10)	0.35(10.6)	0.38(0.09)	0.38(11.0, 0.09)	0.42(0.11, 0.11)
5	0.28(9.8)	0.33(0.18)	0.28(9.5, **0.00**)	0.37(0.32, 0.12)	0.38(10.6)	0.47(0.24)	0.41(11.0, 0.08)	0.52(0.27, 0.11)
6	0.39(9.8)	0.46(0.18)	0.43(9.5, 0.10)	0.49(0.14, 0.07)	0.31(10.6)	0.28(-0.10)	0.31(11.0, **0.00**)	0.30(-0.03, 0.07)
7	0.23(9.8)	0.23(**0.00**)	0.25(9.5, 0.09)	0.28(0.12, 0.22)	0.47(10.6)	0.60(0.28)	0.48(11.0, 0.02)	0.62(0.29, 0.03)
8	0.30(9.8)	0.27(-0.10)	0.34(9.5, 0.13)	0.32(-0.06, 0.19)	0.30(10.6)	0.36(0.20)	0.34(11.0, 0.13)	0.38(0.12, 0.06)
9	0.21(9.8)	0.21(**0.00**)	0.27(9.5, 0.29)	0.24(-0.11, 0.14)	0.32(10.6)	0.36(0.13)	0.38(11.0, 0.19)	0.39(0.03, 0.08)
10	0.41(9.8)	0.49(0.20)	0.40(9.5, **-0.02**)	0.52(0.30, 0.06)	0.35(10.6)	0.42(0.20)	0.41(11.0, 0.17)	0.48(0.17, 0.14)
11	0.27(9.8)	0.34(0.26)	0.28(9.5, **0.04**)	0.39(0.39, 0.15)	0.32(10.6)	0.37(0.16)	0.35(11.0, 0.09)	0.40(0.14, 0.08)
12	0.38(9.8)	0.42(0.11)	0.44(9.5, 0.16)	0.46(0.05, 0.10)	0.51(10.6)	0.60(0.18)	0.54(11.0, 0.06)	0.63(0.17, 0.05)
13	0.32(9.8)	0.36(0.13)	0.29(9.5, -0.09)	0.40(0.38, 0.11)	0.38(10.6)	0.41(0.08)	0.42(11.0, 0.11)	0.44(0.05, 0.07)
14	0.27(9.8)	0.27(**0.00**)	0.33(9.5, 0.22)	0.33(**0.00**, 0.22)	0.35(10.6)	0.38(0.09)	0.36(11.0, **0.03**)	0.41(0.14, 0.08)
15	0.23(9.8)	0.33(0.43)	0.25(9.5, **0.09**)	0.37(0.48, 0.12)	0.47(10.6)	0.54(0.15)	0.46(11.0, **-0.02**)	0.55(0.20, 0.02)
16	0.30(9.8)	0.41(0.37)	0.31(9.5, **0.03**)	0.45(0.45, 0.10)	0.48(10.6)	0.54(0.13)	0.51(11.0, 0.06)	0.56(0.10, 0.04)
17	0.05(9.8)	0.10(1.00)	0.08(9.5, 0.60)	0.14(0.75, 0.40)	0.58(10.6)	0.61(0.05)	0.58(11.0, 0.00)	0.62(0.07, 0.02)
18	0.15(9.8)	0.20(0.33)	0.15(9.5, **0.00**)	0.22(0.47, 0.10)	0.24(10.6)	0.26(0.08)	0.22(11.0, -0.08)	0.27(0.23, 0.04)
19	0.35(9.8)	0.40(0.14)	0.39(9.5, 0.11)	0.43(0.10, 0.07)	0.42(10.6)	0.42(**0.00**)	0.41(11.0, **-0.02**)	0.44(0.07, 0.05)
20	0.26(9.8)	0.42(0.62)	0.26(9.5, **0.00**)	0.44(0.69, 0.05)	0.39(10.6)	0.44(0.13)	0.38(11.0, **-0.03**)	0.46(0.21, 0.05)
21	0.39(9.8)	0.48(0.23)	0.42(9.5, 0.08)	0.51(0.21, 0.06)	0.37(10.6)	0.44(0.19)	0.38(11.0, **0.03**)	0.45(0.18, 0.02)
22	0.44(9.8)	0.44(**0.00**)	0.45(9.5, **0.02**)	0.47(0.04, 0.07)	0.39(10.6)	0.43(0.10)	0.36(11.0, -0.08)	0.47(0.31, 0.09)
23	0.30(9.8)	0.36(0.20)	0.29(9.5, **-0.03**)	0.39(0.34, 0.08)	0.37(10.6)	0.39(0.05)	0.39(11.0, 0.05)	0.42(0.08, 0.08)
24	0.19(9.8)	0.21(0.11)	0.19(9.5, **0.00**)	0.25(0.32, 0.19)	0.44(10.6)	0.49(0.11)	0.49(11.0, 0.11)	0.51(0.04, 0.04)
25	0.15(9.8)	0.27(0.80)	0.16(9.5, **0.07**)	0.31(0.94, 0.15)	0.30(10.6)	0.37(0.23)	0.35(11.0, 0.17)	0.42(0.20, 0.14)
Mean	0.28(9.8)	0.34(0.21)	0.30(9.5, 0.05)	0.37(0.23, 0.10)	0.38(10.6)	0.43(0.13)	0.40(11.1, 0.06)	0.46(0.15, 0.07)

[a] In parentheses is the number of QTL identified by QP based on the SE model; [b] In parentheses is the gain in prediction accuracy with GWP over QP based on the SE model; [c] The first value in parentheses is the number of QTL identified by QP based on the ME model; and the second one the

gain with ME over SE for QP; [d] The first value in parentheses is the gain in accuracy with GWP over QP based on the ME model; the second one is the gain in accuracy with ME over SE using GWP; and the third one is the gain in accuracy with GWP over PP. Bold in parentheses indicates the number is not significant at $\alpha = 0.05$.

Table S21 Accuracy of AP prediction for environment E3 with QP and GWP in CV2

	LL				LW			
	SE		ME		SE		ME	
PopId	QP[a]	GWP[b]	QP[c]	GWP[d]	QP[a]	GWP[b]	QP[c]	GWP[d]
1	0.29(7.8)	0.41(0.41)	0.34(9.5, 0.17)	0.45(0.32, 0.10)	0.20(7.4)	0.23(0.15)	0.26(11.0, 0.30)	0.27(**0.01**, 0.17)
2	0.27(7.8)	0.29(0.07)	0.27(9.5, **0.00**)	0.35(0.30, 0.21)	0.41(7.4)	0.47(0.15)	0.44(11.0, 0.07)	0.52(0.18, 0.11)
3	0.11(7.8)	0.21(0.91)	0.14(9.5, 0.27)	0.23(0.64, 0.10)	0.34(7.4)	0.36(0.06)	0.32(11.0, -0.06)	0.38(0.19, 0.06)
4	0.29(7.8)	0.44(0.52)	0.31(9.5, **0.07**)	0.46(0.48, 0.05)	0.18(7.4)	0.22(0.22)	0.18(11.0, **0.00**)	0.26(0.44, 0.18)
5	0.22(7.8)	0.22(**0.00**)	0.24(9.5, 0.09)	0.27(0.13, 0.23)	0.38(7.4)	0.44(0.16)	0.37(11.0, **-0.03**)	0.46(0.24, 0.05)
6	0.39(7.8)	0.48(0.23)	0.37(9.5, -0.05)	0.50(0.35, 0.04)	0.19(7.4)	0.26(0.37)	0.20(11.0, **0.05**)	0.27(0.35, 0.04)
7	0.25(7.8)	0.25(**0.00**)	0.19(9.5, -0.24)	0.28(0.47, 0.12)	0.22(7.4)	0.32(0.45)	0.29(11.0, 0.32)	0.37(0.28, 0.16)
8	0.22(7.8)	0.28(0.27)	0.24(9.5, 0.09)	0.32(0.33, 0.14)	0.23(7.4)	0.29(0.26)	0.26(11.0, 0.13)	0.31(0.19, 0.07)
9	0.30(7.8)	0.36(0.20)	0.35(9.5, 0.17)	0.39(0.11, 0.08)	0.32(7.4)	0.33(0.03)	0.34(11.0, 0.06)	0.34(**0.00**, 0.03)
10	0.33(7.8)	0.48(0.45)	0.38(9.5, 0.15)	0.51(0.34, 0.06)	0.42(7.4)	0.55(0.31)	0.44(11.0, 0.05)	0.57(0.30, 0.04)
11	0.18(7.8)	0.27(0.50)	0.17(9.5, **-0.06**)	0.31(0.82, 0.15)	0.19(7.4)	0.27(0.42)	0.23(11.0, 0.21)	0.31(0.35, 0.15)
12	0.25(7.8)	0.32(0.28)	0.30(9.5, 0.20)	0.35(0.17, 0.09)	0.28(7.4)	0.32(0.14)	0.30(11.0, 0.07)	0.33(0.10, 0.03)
13	0.33(7.8)	0.38(0.15)	0.42(9.5, 0.27)	0.46(0.10, 0.21)	0.39(7.4)	0.40(0.03)	0.43(11.0, 0.10)	0.42(-0.02, 0.05)
14	0.19(7.8)	0.26(0.37)	0.19(9.5, **0.00**)	0.30(0.58, 0.15)	0.22(7.4)	0.26(0.18)	0.28(11.0, 0.27)	0.32(0.14, 0.23)
15	0.11(7.8)	0.25(1.27)	0.14(9.5, 0.27)	0.31(1.21, 0.24)	0.39(7.4)	0.49(0.26)	0.45(11.0, 0.15)	0.52(0.16, 0.06)
16	0.13(7.8)	0.20(0.54)	0.14(9.5, **0.08**)	0.24(0.71, 0.20)	0.37(7.4)	0.46(0.24)	0.44(11.0, 0.19)	0.52(0.18, 0.13)
17	0.25(7.8)	0.29(0.16)	0.24(9.5, **-0.04**)	0.31(0.29, 0.07)	0.21(7.4)	0.31(0.48)	0.31(11.0, 0.48)	0.37(0.19, 0.19)
18	0.08(7.8)	0.11(0.38)	0.18(9.5, 1.25)	0.15(-0.17, 0.36)	0.34(7.4)	0.37(0.09)	0.36(11.0, 0.06)	0.39(0.08, 0.05)
19	0.33(7.8)	0.39(0.18)	0.41(9.5, 0.24)	0.42(0.02, 0.08)	0.33(7.4)	0.30(-0.09)	0.33(11.0, **0.00**)	0.34(0.03, 0.13)
20	0.21(7.8)	0.35(0.67)	0.24(9.5, 0.14)	0.39(0.63, 0.11)	0.41(7.4)	0.46(0.12)	0.43(11.0, 0.05)	0.50(0.16, 0.09)
21	0.30(7.8)	0.46(0.53)	0.42(9.5, 0.40)	0.52(0.24, 0.13)	0.37(7.4)	0.42(0.14)	0.44(11.0, 0.19)	0.45(0.02, 0.07)
22	0.23(7.8)	0.21(-0.09)	0.31(9.5, 0.35)	0.26(-0.16, 0.24)	0.19(7.4)	0.27(0.42)	0.25(11.0, 0.32)	0.31(0.24, 0.15)
23	0.20(7.8)	0.23(0.15)	0.19(9.5, **-0.05**)	0.27(0.42, 0.17)	0.21(7.4)	0.29(0.38)	0.26(11.0, 0.24)	0.33(0.27, 0.14)
24	0.12(7.8)	0.20(0.67)	0.16(9.5, 0.33)	0.23(0.44, 0.15)	0.40(7.4)	0.39(-0.03)	0.44(11.0, 0.10)	0.43(-0.02, 0.10)
25	0.23(7.8)	0.38(0.65)	0.20(9.5, -0.13)	0.39(0.95, 0.03)	0.23(7.4)	0.26(0.13)	0.23(11.0, **0.00**)	0.28(0.22, 0.08)
Mean	0.23(7.8)	0.31(0.35)	0.26(9.5, 0.13)	0.35(0.35, 0.12)	0.30(7.3)	0.35(0.17)	0.33(11.1, 0.12)	0.38(0.15, 0.09)

[a] In parentheses is the number of QTL identified by QP based on the SE model; [b] In parentheses is the gain in prediction accuracy with GWP over QP based on the SE model; [c] The first value in parentheses is the number of QTL identified by QP based on the ME model; and the second one the gain

with ME over SE for QP; [d] The first value in parentheses is the gain in accuracy with GWP over QP based on the ME model; the second one is the gain in accuracy with ME over SE using GWP; and the third one is the gain in accuracy with GWP over PP. Bold in parentheses indicates the number is not significant at $\alpha = 0.05$.

Table S22 Accuracy of AP prediction for environment E4 with QP and GWP in CV2

	LL				LW			
	SE		ME		SE		ME	
PopId	QP[a]	GWP[b]	QP[c]	GWP[d]	QP[a]	GWP[b]	QP[c]	GWP[d]
1	0.22(7.4)	0.30(0.36)	0.24(9.5, **0.09**)	0.36(0.50, 0.20)	0.30(11.0)	0.34(0.13)	0.34(11.0, 0.13)	0.37(0.09, 0.09)
2	0.18(7.4)	0.25(0.39)	0.21(9.5, 0.17)	0.28(0.33, 0.12)	0.40(11.0)	0.44(0.10)	0.41(11.0, 0.02)	0.47(0.15, 0.07)
3	0.23(7.4)	0.26(0.13)	0.23(9.5, **0.00**)	0.30(0.30, 0.15)	0.37(11.0)	0.40(0.08)	0.37(11.0, **0.00**)	0.44(0.19, 0.10)
4	0.33(7.4)	0.45(0.36)	0.37(9.5, 0.12)	0.50(0.35, 0.11)	0.29(11.0)	0.31(0.07)	0.30(11.0, **0.03**)	0.34(0.13, 0.10)
5	0.23(7.4)	0.39(0.70)	0.26(9.5, 0.13)	0.42(0.62, 0.08)	0.28(11.0)	0.35(0.25)	0.32(11.0, 0.14)	0.40(0.25, 0.14)
6	0.38(7.4)	0.46(0.21)	0.42(9.5, 0.11)	0.52(0.24, 0.13)	0.31(11.0)	0.36(0.16)	0.31(11.0, **0.00**)	0.37(0.19, 0.03)
7	0.22(7.4)	0.36(0.64)	0.24(9.5, **0.09**)	0.39(0.63, 0.08)	0.47(11.0)	0.53(0.13)	0.50(11.0, 0.06)	0.57(0.14, 0.08)
8	0.24(7.4)	0.35(0.46)	0.32(9.5, 0.33)	0.40(0.25, 0.14)	0.32(11.0)	0.40(0.25)	0.36(11.0, 0.12)	0.44(0.22, 0.10)
9	0.27(7.4)	0.34(0.26)	0.30(9.5, 0.11)	0.37(0.23, 0.09)	0.38(11.0)	0.42(0.11)	0.41(11.0, 0.08)	0.46(0.12, 0.10)
10	0.35(7.4)	0.46(0.31)	0.34(9.5, **-0.03**)	0.51(0.50, 0.11)	0.46(11.0)	0.55(0.20)	0.46(11.0, **0.00**)	0.58(0.26, 0.05)
11	0.14(7.4)	0.27(0.93)	0.20(9.5, 0.43)	0.32(0.60, 0.19)	0.35(11.0)	0.37(0.06)	0.34(11.0, **-0.03**)	0.38(0.12, 0.03)
12	0.31(7.4)	0.34(0.10)	0.40(9.5, 0.29)	0.41(0.02, 0.21)	0.56(11.0)	0.58(0.04)	0.56(11.0, **0.00**)	0.60(0.07, 0.03)
13	0.18(7.4)	0.16(-0.11)	0.24(9.5, 0.33)	0.21(-0.13, 0.31)	0.29(11.0)	0.32(0.10)	0.37(11.0, 0.28)	0.38(0.03, 0.19)
14	0.25(7.4)	0.35(0.40)	0.28(9.5, 0.12)	0.38(0.36, 0.09)	0.33(11.0)	0.35(0.06)	0.36(11.0, 0.09)	0.38(0.06, 0.09)
15	0.17(7.4)	0.25(0.47)	0.21(9.5, 0.24)	0.30(0.43, 0.20)	0.56(11.0)	0.61(0.09)	0.57(11.0, 0.02)	0.63(0.11, 0.03)
16	0.18(7.4)	0.25(0.39)	0.20(9.5, 0.11)	0.30(0.50, 0.20)	0.49(11.0)	0.58(0.18)	0.53(11.0, 0.08)	0.60(0.13, 0.03)
17	0.14(7.4)	0.25(0.79)	0.15(9.5, **0.07**)	0.27(0.80, 0.08)	0.54(11.0)	0.55(0.02)	0.54(11.0, **0.00**)	0.57(0.06, 0.04)
18	0.12(7.4)	0.16(0.33)	0.19(9.5, 0.58)	0.18(-0.05, 0.13)	0.39(11.0)	0.41(0.05)	0.39(11.0, **0.00**)	0.42(0.08, 0.02)
19	0.22(7.4)	0.21(-0.05)	0.27(9.5, 0.23)	0.25(-0.07, 0.19)	0.40(11.0)	0.38(-0.05)	0.44(11.0, 0.10)	0.41(-0.07, 0.08)
20	0.27(7.4)	0.42(0.56)	0.32(9.5, 0.19)	0.46(0.44, 0.10)	0.47(11.0)	0.52(0.11)	0.47(11.0, 0.00)	0.56(0.19, 0.08)
21	0.43(7.4)	0.52(0.21)	0.48(9.5, 0.12)	0.55(0.15, 0.06)	0.35(11.0)	0.41(0.17)	0.40(11.0, 0.14)	0.45(0.13, 0.10)
22	0.30(7.4)	0.29(-0.03)	0.31(9.5, **0.03**)	0.31(**0.00**, 0.07)	0.34(11.0)	0.42(0.24)	0.33(11.0, -0.03)	0.45(0.36, 0.07)
23	0.06(7.4)	0.14(1.33)	0.05(9.5, -0.17)	0.17(2.40, 0.21)	0.31(11.0)	0.32(0.03)	0.30(11.0, -0.03)	0.35(0.17, 0.09)
24	0.18(7.4)	0.28(0.56)	0.21(9.5, 0.17)	0.29(0.38, 0.04)	0.46(11.0)	0.50(0.09)	0.49(11.0, 0.07)	0.52(0.06, 0.04)
25	0.15(7.4)	0.28(0.87)	0.17(9.5, 0.13)	0.33(0.94, 0.18)	0.36(11.0)	0.42(0.17)	0.34(11.0, -0.06)	0.44(0.29, 0.05)
Mean	0.23(7.4)	0.31(0.35)	0.26(9.5, 0.15)	0.35(0.35, 0.12)	0.39(11.0)	0.43(0.10)	0.41(11.1, 0.04)	0.46(0.12, 0.07)

[a] In parentheses is the number of QTL identified by QP based on the SE model; [b] In parentheses is the gain in prediction accuracy with GWP over QP based on the SE model; [c] The first value in parentheses is the number of QTL identified by QP based on the ME model; and the second one the gain

with ME over SE for QP; [d] The first value in parentheses is the gain in accuracy with GWP over QP based on the ME model; the second one is the gain in accuracy with ME over SE using GWP; and the third one is the gain in accuracy with GWP over PP. Bold in parentheses indicates the number is not significant at $\alpha = 0.05$.

Table S23 Accuracy of WP prediction for environment E1 with four ME GWP models in CV1

	LL				LW			
PopId	SG-SR	SG-UR[a]	UG-SR[b]	UG-UR[c]	SG-SR	SG-UR[a]	UG-SR[b]	UG-UR[c]
1	0.33	0.27(-0.17)	0.42(0.28)	0.42(**0.00**)	0.29	0.25(-0.16)	0.39(0.35)	0.39(**0.00**)
2	0.17	0.10(-0.42)	0.24(0.44)	0.24(**0.00**)	0.40	0.36(-0.10)	0.45(0.12)	0.45(**0.00**)
3	0.28	0.25(-0.08)	0.27(-**0.01**)	0.28(0.03)	0.55	0.52(-0.05)	0.59(0.06)	0.59(**0.00**)
4	0.48	0.44(-0.07)	0.50(0.05)	0.50(**0.00**)	0.49	0.47(-0.04)	0.53(0.08)	0.53(**0.00**)
5	0.32	0.24(-0.24)	0.39(0.22)	0.39(**0.00**)	0.20	0.12(-0.41)	0.30(0.49)	0.29(-0.02)
6	0.50	0.44(-0.11)	0.53(0.07)	0.53(**0.00**)	0.32	0.30(-0.07)	0.38(0.18)	0.38(**0.00**)
7	0.52	0.47(-0.09)	0.58(0.12)	0.57(-**0.01**)	0.48	0.44(-0.08)	0.52(0.09)	0.52(**0.00**)
8	0.31	0.24(-0.24)	0.37(0.19)	0.35(-0.05)	0.31	0.25(-0.19)	0.36(0.19)	0.38(0.04)
9	0.34	0.28(-0.19)	0.39(0.15)	0.38(-0.03)	0.17	0.12(-0.26)	0.35(1.11)	0.36(0.01)
10	0.47	0.42(-0.12)	0.50(0.05)	0.49(-**0.01**)	0.39	0.33(-0.15)	0.52(0.33)	0.52(**0.00**)
11	0.37	0.33(-0.12)	0.45(0.20)	0.45(**0.00**)	0.39	0.39(-0.02)	0.42(0.07)	0.42(**0.00**)
12	0.49	0.43(-0.11)	0.54(0.12)	0.54(**0.00**)	0.54	0.52(-0.05)	0.57(0.05)	0.57(**0.00**)
13	0.45	0.41(-0.09)	0.44(-0.03)	0.47(0.07)	0.50	0.46(-0.08)	0.52(0.05)	0.52(**0.00**)
14	0.38	0.31(-0.19)	0.43(0.11)	0.42(**0.00**)	0.47	0.43(-0.09)	0.50(0.07)	0.50(**0.00**)
15	0.28	0.20(-0.28)	0.32(0.12)	0.31(-0.03)	0.30	0.27(-0.11)	0.34(0.14)	0.34(**0.00**)
16	0.33	0.28(-0.15)	0.36(0.07)	0.36(**0.00**)	0.46	0.44(-0.04)	0.54(0.17)	0.54(**0.00**)
17	0.27	0.24(-0.10)	0.29(0.08)	0.29(**0.00**)	0.55	0.52(-0.05)	0.59(0.07)	0.59(**0.00**)
18	0.28	0.25(-0.12)	0.29(0.03)	0.28(-0.03)	0.39	0.38(-0.04)	0.43(0.10)	0.43(**0.00**)
19	0.37	0.31(-0.16)	0.41(0.12)	0.40(-0.04)	0.41	0.34(-0.16)	0.44(0.07)	0.44(**0.00**)
20	0.47	0.40(-0.15)	0.51(0.07)	0.48(-0.05)	0.47	0.44(-0.07)	0.50(0.06)	0.51(0.02)
21	0.49	0.44(-0.10)	0.50(0.02)	0.51(**0.01**)	0.47	0.46(-0.02)	0.47(**0.00**)	0.48(0.01)
22	0.35	0.30(-0.15)	0.42(0.19)	0.41(-**0.01**)	0.32	0.28(-0.11)	0.41(0.27)	0.42(0.02)
23	0.42	0.37(-0.12)	0.47(0.12)	0.46(-0.03)	0.29	0.22(-0.24)	0.43(0.47)	0.43(**0.00**)
24	0.45	0.41(-0.08)	0.45(**0.01**)	0.44(-0.02)	0.50	0.47(-0.06)	0.53(0.06)	0.53(**0.00**)
25	0.50	0.45(-0.10)	0.53(0.05)	0.51(-0.03)	0.45	0.42(-0.08)	0.48(0.06)	0.48(**0.00**)
Mean	0.38	0.33(-0.14)	0.42(0.10)	0.42(0.00)	0.40	0.37(-0.09)	0.46(0.14)	0.46(0.00)

[a] In parentheses is the gain in prediction accuracy with SG-UR over SG-SR; [b] In parentheses is the gain in prediction accuracy with UG-SR over SG-SR;

[c] In parentheses is the gain in prediction accuracy with UG-UR over UG-SR; Bold in parentheses indicates the number is not significant at $\alpha = 0.05$.

Table S24 Accuracy of WP prediction for environment E2 with four ME GWP models in CV1

	**LL**				**LW**			
**PopId**	**SG-SR**	**SG-UR**[a]	**UG-SR**[b]	**UG-UR**[c]	**SG-SR**	**SG-UR**[a]	**UG-SR**[b]	**UG-UR**[c]
1	0.67	0.67(**0.00**)	0.67(**0.00**)	0.67(**0.00**)	0.46	0.43(-0.06)	0.50(0.09)	0.50(**0.00**)
2	0.40	0.36(-0.10)	0.42(0.04)	0.40(-0.03)	0.49	0.46(-0.07)	0.53(0.07)	0.53(**0.00**)
3	0.17	0.13(-0.21)	0.19(0.14)	0.18(-0.06)	0.59	0.57(-0.04)	0.61(0.03)	0.61(**0.00**)
4	0.56	0.54(-0.04)	0.57(0.03)	0.57(**0.00**)	0.59	0.57(-0.03)	0.62(0.04)	0.62(**0.00**)
5	0.50	0.47(-0.07)	0.51(0.02)	0.51(**0.00**)	0.45	0.39(-0.12)	0.48(0.07)	0.46(-0.03)
6	0.49	0.43(-0.11)	0.51(0.06)	0.50(-0.03)	0.33	0.32(-0.04)	0.34(0.03)	0.34(**0.00**)
7	0.42	0.36(-0.14)	0.52(0.23)	0.51(-0.03)	0.58	0.54(-0.06)	0.61(0.06)	0.62(0.01)
8	0.35	0.27(-0.23)	0.40(0.14)	0.37(-0.08)	0.35	0.29(-0.17)	0.41(0.17)	0.42(0.03)
9	0.30	0.25(-0.19)	0.34(0.12)	0.32(-0.07)	0.44	0.41(-0.06)	0.44(**0.00**)	0.43(-0.02)
10	0.50	0.43(-0.12)	0.52(0.05)	0.51(-0.03)	0.36	0.26(-0.26)	0.39(0.08)	0.36(-0.07)
11	0.52	0.50(-0.05)	0.55(0.04)	0.55(**0.00**)	0.40	0.39(-0.03)	0.43(0.07)	0.43(**0.00**)
12	0.56	0.51(-0.10)	0.60(0.08)	0.59(-0.02)	0.58	0.56(-0.03)	0.61(0.05)	0.61(**0.00**)
13	0.40	0.32(-0.20)	0.44(0.08)	0.45(0.03)	0.39	0.32(-0.19)	0.47(0.20)	0.47(**0.00**)
14	0.42	0.38(-0.11)	0.44(0.05)	0.42(-0.04)	0.27	0.19(-0.29)	0.37(0.40)	0.36(-0.04)
15	0.46	0.43(-0.08)	0.45(-0.03)	0.46(0.02)	0.55	0.53(-0.04)	0.58(0.05)	0.58(**0.00**)
16	0.31	0.26(-0.16)	0.33(0.06)	0.32(-0.02)	0.56	0.55(-0.02)	0.58(0.03)	0.59(0.01)
17	0.13	0.08(-0.40)	0.20(0.52)	0.19(**-0.02**)	0.61	0.59(-0.03)	0.64(0.04)	0.64(**0.00**)
18	0.29	0.23(-0.20)	0.32(0.12)	0.31(-0.03)	0.30	0.27(-0.08)	0.42(0.42)	0.43(**0.01**)
19	0.28	0.20(-0.29)	0.34(0.20)	0.30(-0.12)	0.40	0.35(-0.11)	0.45(0.14)	0.46(0.02)
20	0.52	0.46(-0.12)	0.55(0.05)	0.54(-0.02)	0.53	0.50(-0.07)	0.56(0.04)	0.56(**0.00**)
21	0.58	0.54(-0.08)	0.61(0.04)	0.61(**0.00**)	0.35	0.32(-0.09)	0.41(0.19)	0.40(-0.02)
22	0.49	0.47(-0.05)	0.50(0.02)	0.50(**0.00**)	0.58	0.56(-0.03)	0.59(0.02)	0.60(0.01)
23	0.43	0.39(-0.10)	0.47(0.09)	0.46(**-0.01**)	0.36	0.32(-0.12)	0.40(0.11)	0.41(0.01)
24	0.16	0.08(-0.53)	0.27(0.63)	0.25(-0.06)	0.42	0.39(-0.08)	0.48(0.14)	0.48(**0.00**)
25	0.38	0.31(-0.20)	0.45(0.17)	0.42(-0.07)	0.47	0.42(-0.12)	0.48(0.01)	0.45(-0.05)
Mean	0.41	0.36(-0.12)	0.45(0.08)	0.44(-0.02)	0.46	0.42(-0.08)	0.50(0.09)	0.49(-0.01)

[a] In parentheses is the gain in prediction accuracy with SG-UR over SG-SR; [b] In parentheses is the gain in prediction accuracy with UG-SR over SG-SR;

[c] In parentheses is the gain in prediction accuracy with UG-UR over UG-SR; Bold in parentheses indicates the number is not significant at $\alpha = 0.05$.

Table S25 Accuracy of WP prediction for environment E3 with four ME GWP models in CV1

	LL				LW			
PopId	SG-SR	SG-UR[a]	UG-SR[b]	UG-UR[c]	SG-SR	SG-UR[a]	UG-SR[b]	UG-UR[c]
1	0.51	0.47(-0.06)	0.55(0.09)	0.55(**0.00**)	0.36	0.33(-0.08)	0.41(0.13)	0.41(**0.00**)
2	0.35	0.29(-0.17)	0.38(0.10)	0.36(-0.06)	0.52	0.49(-0.05)	0.55(0.06)	0.55(**0.00**)
3	0.24	0.20(-0.17)	0.26(0.10)	0.26(**0.00**)	0.27	0.23(-0.18)	0.37(0.35)	0.37(**0.00**)
4	0.50	0.45(-0.10)	0.55(0.10)	0.54(-0.02)	0.18	0.12(-0.32)	0.28(0.56)	0.29(0.00)
5	0.44	0.39(-0.11)	0.46(0.05)	0.46(**0.00**)	0.30	0.22(-0.26)	0.39(0.29)	0.39(**0.00**)
6	0.47	0.40(-0.15)	0.54(0.14)	0.54(**0.00**)	0.31	0.29(-0.07)	0.35(0.15)	0.35(**0.00**)
7	0.49	0.44(-0.10)	0.55(0.13)	0.55(**0.00**)	0.42	0.38(-0.10)	0.49(0.17)	0.50(0.01)
8	0.32	0.24(-0.26)	0.38(0.18)	0.35(-0.07)	0.48	0.44(-0.07)	0.49(0.03)	0.49(**0.00**)
9	0.42	0.36(-0.13)	0.45(0.07)	0.45(**0.00**)	0.45	0.43(-0.05)	0.48(0.07)	0.48(**0.00**)
10	0.42	0.34(-0.20)	0.48(0.13)	0.48(**0.00**)	0.56	0.52(-0.08)	0.58(0.04)	0.56(-0.04)
11	0.54	0.51(-0.05)	0.56(0.04)	0.56(**0.00**)	0.36	0.35(-0.03)	0.41(0.14)	0.41(**0.00**)
12	0.43	0.35(-0.17)	0.49(0.15)	0.50(0.02)	0.28	0.23(-0.17)	0.37(0.35)	0.38(0.00)
13	0.41	0.31(-0.25)	0.45(0.09)	0.43(-0.05)	0.26	0.16(-0.37)	0.35(0.38)	0.35(**0.00**)
14	0.31	0.23(-0.24)	0.35(0.15)	0.35(**0.00**)	0.32	0.27(-0.16)	0.42(0.28)	0.42(**0.00**)
15	0.25	0.15(-0.39)	0.30(0.21)	0.30(**0.00**)	0.40	0.37(-0.07)	0.47(0.19)	0.48(0.01)
16	0.32	0.28(-0.15)	0.34(0.05)	0.33(-0.02)	0.36	0.34(-0.06)	0.45(0.26)	0.45(**0.00**)
17	0.32	0.28(-0.13)	0.37(0.14)	0.37(**0.00**)	0.38	0.33(-0.13)	0.45(0.17)	0.45(**0.00**)
18	0.17	0.12(-0.28)	0.20(0.16)	0.21(**0.02**)	0.33	0.31(-0.06)	0.39(0.19)	0.39(**0.00**)
19	0.46	0.41(-0.11)	0.48(0.06)	0.47(-0.03)	0.44	0.40(-0.09)	0.46(0.05)	0.46(**0.00**)
20	0.34	0.25(-0.27)	0.40(0.19)	0.39(-0.02)	0.43	0.39(-0.09)	0.47(0.08)	0.46(-0.01)
21	0.47	0.41(-0.14)	0.53(0.12)	0.54(0.02)	0.38	0.37(-0.04)	0.42(0.11)	0.42(**0.00**)
22	0.38	0.34(-0.10)	0.41(0.08)	0.40(-0.01)	0.35	0.31(-0.09)	0.42(0.20)	0.43(0.02)
23	0.32	0.26(-0.19)	0.41(0.30)	0.42(0.01)	0.38	0.33(-0.13)	0.43(0.13)	0.42(-0.02)
24	0.26	0.19(-0.25)	0.30(0.16)	0.27(-0.09)	0.41	0.37(-0.10)	0.47(0.13)	0.48(0.02)
25	0.37	0.28(-0.25)	0.44(0.21)	0.40(-0.09)	0.29	0.24(-0.15)	0.33(0.15)	0.34(0.02)
Mean	0.38	0.32(-0.16)	0.43(0.12)	0.42(-0.02)	0.37	0.33(-0.11)	0.43(0.16)	0.43(0.00)

[a] In parentheses is the gain in prediction accuracy with SG-UR over SG-SR; [b] In parentheses is the gain in prediction accuracy with UG-SR over SG-SR;

[c] In parentheses is the gain in prediction accuracy with UG-UR over UG-SR; Bold in parentheses indicates the number is not significant at α = 0.05.

Table S26 Accuracy of WP prediction for environment E4 with four ME GWP models in CV1

	LL				LW			
PopId	SG-SR	SG-UR[a]	UG-SR[b]	UG-UR[c]	SG-SR	SG-UR[a]	UG-SR[b]	UG-UR[c]
1	0.47	0.44(-0.06)	0.51(0.09)	0.51(**0.00**)	0.50	0.48(-0.05)	0.54(0.08)	0.54(**0.00**)
2	0.30	0.25(-0.17)	0.34(0.13)	0.32(-0.05)	0.48	0.43(-0.10)	0.51(0.05)	0.51(**0.00**)
3	0.26	0.24(-0.10)	0.27(0.04)	0.27(**0.00**)	0.42	0.39(-0.07)	0.52(0.23)	0.52(**0.00**)
4	0.52	0.48(-0.08)	0.56(0.08)	0.56(**0.00**)	0.51	0.49(-0.05)	0.54(0.06)	0.54(**0.00**)
5	0.35	0.30(-0.15)	0.39(0.12)	0.39(**0.00**)	0.45	0.40(-0.11)	0.48(0.08)	0.47(-0.03)
6	0.45	0.38(-0.15)	0.52(0.16)	0.51(-0.02)	0.30	0.28(-0.08)	0.36(0.19)	0.36(**0.00**)
7	0.60	0.56(-0.06)	0.63(0.05)	0.62(-0.01)	0.62	0.60(-0.03)	0.64(0.04)	0.65(0.00)
8	0.40	0.32(-0.19)	0.44(0.12)	0.43(-0.04)	0.40	0.34(-0.14)	0.45(0.12)	0.45(**0.00**)
9	0.34	0.27(-0.19)	0.38(0.12)	0.37(-0.03)	0.47	0.45(-0.05)	0.48(0.01)	0.47(-0.02)
10	0.41	0.34(-0.18)	0.45(0.09)	0.43(-0.03)	0.63	0.59(-0.05)	0.65(0.04)	0.64(-0.01)
11	0.29	0.23(-0.18)	0.38(0.33)	0.38(**0.00**)	0.44	0.43(-0.03)	0.49(0.11)	0.49(**0.00**)
12	0.50	0.44(-0.12)	0.56(0.11)	0.54(-0.03)	0.57	0.54(-0.04)	0.59(0.05)	0.60(0.01)
13	0.24	0.08(-0.68)	0.30(0.28)	0.34(0.12)	0.42	0.37(-0.12)	0.49(0.17)	0.49(**0.00**)
14	0.34	0.27(-0.21)	0.39(0.13)	0.37(-0.05)	0.45	0.40(-0.11)	0.49(0.08)	0.49(**0.00**)
15	0.36	0.29(-0.19)	0.38(0.06)	0.37(-0.02)	0.53	0.51(-0.05)	0.55(0.04)	0.56(0.01)
16	0.20	0.13(-0.38)	0.28(0.39)	0.28(**0.00**)	0.53	0.51(-0.03)	0.57(0.08)	0.57(**0.00**)
17	0.34	0.30(-0.10)	0.37(0.10)	0.36(-0.03)	0.56	0.54(-0.05)	0.60(0.07)	0.61(0.01)
18	0.26	0.22(-0.17)	0.29(0.10)	0.28(-0.03)	0.31	0.29(-0.06)	0.38(0.22)	0.38(**0.00**)
19	0.26	0.18(-0.28)	0.32(0.26)	0.31(-0.02)	0.51	0.46(-0.09)	0.54(0.07)	0.55(0.01)
20	0.46	0.39(-0.15)	0.49(0.08)	0.48(-0.03)	0.52	0.49(-0.05)	0.54(0.03)	0.55(0.02)
21	0.61	0.56(-0.09)	0.64(0.04)	0.64(**0.00**)	0.36	0.34(-0.06)	0.41(0.13)	0.41(**0.00**)
22	0.47	0.44(-0.06)	0.48(0.02)	0.48(**0.00**)	0.55	0.52(-0.04)	0.59(0.07)	0.59(**0.00**)
23	0.39	0.33(-0.14)	0.45(0.17)	0.45(**0.00**)	0.48	0.44(-0.08)	0.51(0.06)	0.51(**0.00**)
24	0.26	0.18(-0.30)	0.32(0.23)	0.29(-0.07)	0.47	0.42(-0.10)	0.51(0.09)	0.51(**0.00**)
25	0.26	0.17(-0.33)	0.34(0.35)	0.33(-0.06)	0.48	0.44(-0.09)	0.49(0.01)	0.47(-0.04)
Mean	0.37	0.31(-0.16)	0.42(0.13)	0.41(-0.02)	0.48	0.45(-0.07)	0.52(0.08)	0.52(0.00)

[a] In parentheses is the gain in prediction accuracy with SG-UR over SG-SR; [b] In parentheses is the gain in prediction accuracy with UG-SR over SG-SR;

[c] In parentheses is the gain in prediction accuracy with UG-UR over UG-SR; Bold in parentheses indicates the number is not significant at $\alpha = 0.05$.

Table S27 Accuracy of AP prediction for environment E1 with four ME GWP models in CV1

	LL				LW			
PopId	SG-SR	SG-UR[a]	UG-SR[b]	UG-UR[c]	SG-SR	SG-UR[a]	UG-SR[b]	UG-UR[c]
1	0.31	0.30(-0.04)	0.34(0.07)	0.33(-0.02)	0.25	0.24(-0.07)	0.27(0.08)	0.26(-0.05)
2	0.19	0.17(-0.11)	0.21(0.12)	0.20(-0.06)	0.42	0.42(**0.00**)	0.42(**0.00**)	0.42(**0.00**)
3	0.21	0.21(**0.00**)	0.19(-0.05)	0.20(0.05)	0.54	0.53(-0.02)	0.54(**0.00**)	0.55(0.01)
4	0.41	0.41(**0.00**)	0.39(-0.04)	0.41(0.04)	0.29	0.30(0.01)	0.28(-0.04)	0.28(**0.00**)
5	0.28	0.29(0.04)	0.25(-0.08)	0.26(0.04)	0.23	0.22(-0.03)	0.24(0.04)	0.24(**0.00**)
6	0.46	0.44(-0.03)	0.47(0.02)	0.47(**0.00**)	0.37	0.37(**0.00**)	0.39(0.05)	0.39(**0.00**)
7	0.24	0.23(-0.07)	0.27(0.12)	0.26(-0.02)	0.36	0.34(-0.06)	0.41(0.14)	0.39(-0.04)
8	0.47	0.45(-0.04)	0.47(**0.00**)	0.47(**0.00**)	0.39	0.38(-0.02)	0.39(**0.00**)	0.39(**0.00**)
9	0.36	0.35(-0.03)	0.38(0.05)	0.38(**0.00**)	0.16	0.16(**0.00**)	0.16(**0.00**)	0.17(0.01)
10	0.37	0.37(**0.00**)	0.38(0.02)	0.38(**0.00**)	0.46	0.44(-0.04)	0.49(0.07)	0.48(-0.02)
11	0.07	0.05(-0.26)	0.11(0.51)	0.09(-0.16)	0.17	0.16(-0.04)	0.18(0.06)	0.18(**0.00**)
12	0.39	0.37(-0.03)	0.40(0.03)	0.40(**0.00**)	0.59	0.58(-0.02)	0.61(0.03)	0.61(**0.00**)
13	0.29	0.26(-0.11)	0.34(0.19)	0.32(-0.06)	0.42	0.42(**0.00**)	0.43(0.02)	0.43(**0.00**)
14	0.40	0.39(-0.02)	0.40(**0.00**)	0.40(**0.00**)	0.36	0.34(-0.04)	0.38(0.07)	0.38(**0.00**)
15	0.24	0.25(**0.01**)	0.23(-0.07)	0.24(0.04)	0.31	0.31(**0.00**)	0.32(**0.01**)	0.31(**-0.01**)
16	0.27	0.26(-0.04)	0.27(**0.00**)	0.28(**0.01**)	0.52	0.52(**0.00**)	0.52(**0.00**)	0.52(**0.00**)
17	0.29	0.28(-0.03)	0.29(**0.00**)	0.30(0.01)	0.43	0.42(-0.02)	0.45(0.05)	0.46(**0.01**)
18	0.14	0.13(-0.05)	0.14(**0.00**)	0.14(**0.00**)	0.30	0.28(-0.05)	0.33(0.10)	0.31(-0.04)
19	0.30	0.29(-0.04)	0.31(0.03)	0.30(-0.01)	0.22	0.21(-0.02)	0.23(0.06)	0.23(**0.00**)
20	0.42	0.41(-0.03)	0.43(0.02)	0.43(**0.00**)	0.43	0.43(**0.00**)	0.44(0.02)	0.44(**0.00**)
21	0.33	0.31(-0.08)	0.36(0.10)	0.35(-0.03)	0.32	0.30(-0.07)	0.35(0.10)	0.34(-0.04)
22	0.35	0.35(**0.00**)	0.34(-0.03)	0.34(**0.00**)	0.29	0.29(**0.00**)	0.30(0.02)	0.30(**0.00**)
23	0.21	0.22(0.03)	0.20(-0.05)	0.20(**0.00**)	0.15	0.13(-0.10)	0.19(0.28)	0.18(-0.04)
24	0.27	0.29(0.06)	0.23(-0.15)	0.25(0.07)	0.45	0.45(**0.00**)	0.45(**0.00**)	0.45(**0.00**)
25	0.42	0.42(**0.00**)	0.40(-0.05)	0.42(0.04)	0.47	0.45(-0.02)	0.47(**0.00**)	0.47(**0.00**)
Mean	0.31	0.30(-0.03)	0.31(0.02)	0.31(0.00)	0.36	0.35(-0.02)	0.37(0.04)	0.37(0.00)

[a] In parentheses is the gain in prediction accuracy with SG-UR over SG-SR; [b] In parentheses is the gain in prediction accuracy with UG-SR over SG-SR;

[c] In parentheses is the gain in prediction accuracy with UG-UR over UG-SR; Bold in parentheses indicates the number is not significant at α = 0.05.

Table S28 Accuracy of AP prediction for environment E2 with four ME GWP models in CV1

	LL				LW			
PopId	SG-SR	SG-UR[a]	UG-SR[b]	UG-UR[c]	SG-SR	SG-UR[a]	UG-SR[b]	UG-UR[c]
1	0.39	0.39(**0.00**)	0.37(-0.03)	0.38(0.01)	0.21	0.20(-0.07)	0.24(0.14)	0.24(**0.00**)
2	0.28	0.26(-0.09)	0.31(0.09)	0.29(-0.05)	0.44	0.43(-0.02)	0.46(0.04)	0.45(-0.01)
3	0.29	0.30(0.02)	0.27(-0.08)	0.28(0.05)	0.54	0.53(-0.01)	0.54(**0.00**)	0.54(**0.00**)
4	0.38	0.37(-0.03)	0.39(0.01)	0.39(**0.00**)	0.34	0.33(-0.03)	0.36(0.06)	0.36(**0.00**)
5	0.33	0.33(**0.00**)	0.32(-0.03)	0.32(**0.00**)	0.47	0.45(-0.03)	0.48(0.04)	0.48(**0.00**)
6	0.47	0.47(**0.00**)	0.47(**0.00**)	0.47(**0.00**)	0.28	0.28(**0.00**)	0.30(0.05)	0.30(**0.00**)
7	0.20	0.20(**0.00**)	0.21(**0.01**)	0.21(**0.00**)	0.60	0.60(**0.00**)	0.58(-0.03)	0.59(0.02)
8	0.25	0.23(-0.06)	0.27(0.08)	0.26(-0.03)	0.34	0.34(**0.00**)	0.34(**0.00**)	0.35(0.01)
9	0.23	0.22(-0.01)	0.22(-0.03)	0.22(**0.00**)	0.34	0.34(**0.00**)	0.34(**0.00**)	0.34(**0.00**)
10	0.47	0.46(-0.02)	0.46(-0.01)	0.47(0.02)	0.41	0.40(-0.04)	0.45(0.09)	0.45(**0.00**)
11	0.29	0.27(-0.07)	0.31(0.06)	0.30(-0.02)	0.37	0.37(**0.00**)	0.38(0.02)	0.37(-0.01)
12	0.41	0.40(-0.02)	0.41(**0.00**)	0.41(**0.00**)	0.62	0.60(-0.02)	0.62(**0.00**)	0.63(0.00)
13	0.35	0.35(**0.00**)	0.35(**0.00**)	0.35(**0.00**)	0.41	0.40(**-0.01**)	0.40(-0.03)	0.40(**0.00**)
14	0.24	0.22(-0.10)	0.28(0.15)	0.26(-0.05)	0.37	0.36(-0.03)	0.39(0.06)	0.39(**0.00**)
15	0.30	0.30(**0.00**)	0.27(-0.07)	0.29(0.06)	0.53	0.53(**0.00**)	0.52(-0.03)	0.52(**0.00**)
16	0.42	0.41(-0.02)	0.41(-0.02)	0.42(0.02)	0.53	0.53(**0.00**)	0.54(0.02)	0.54(**0.00**)
17	0.11	0.09(-0.17)	0.13(0.17)	0.12(-0.06)	0.60	0.60(**0.00**)	0.57(-0.05)	0.59(0.03)
18	0.20	0.20(**0.00**)	0.18(-0.08)	0.19(0.06)	0.23	0.23(**0.00**)	0.22(-0.06)	0.22(**0.00**)
19	0.39	0.37(-0.05)	0.41(0.03)	0.40(-0.01)	0.40	0.40(**0.00**)	0.39(-0.02)	0.39(**0.00**)
20	0.41	0.40(-0.01)	0.41(**0.00**)	0.41(**0.00**)	0.44	0.43(-0.01)	0.43(-0.02)	0.43(**0.00**)
21	0.47	0.46(-0.02)	0.47(**0.00**)	0.47(**0.00**)	0.45	0.44(-0.02)	0.45(**0.00**)	0.45(**0.00**)
22	0.43	0.42(-0.03)	0.44(0.02)	0.43(-0.01)	0.40	0.40(**0.00**)	0.42(0.04)	0.42(**0.00**)
23	0.35	0.34(**-0.01**)	0.34(-0.01)	0.35(0.02)	0.38	0.37(-0.04)	0.40(0.04)	0.40(**0.00**)
24	0.21	0.20(-0.03)	0.21(**0.00**)	0.21(**0.00**)	0.45	0.46(0.02)	0.44(-0.03)	0.45(0.03)
25	0.24	0.23(-0.04)	0.25(0.05)	0.24(-0.03)	0.35	0.33(-0.04)	0.37(0.07)	0.37(**0.00**)
Mean	0.32	0.32(-0.02)	0.33(0.01)	0.33(0.00)	0.42	0.41(-0.01)	0.42(0.01)	0.43(0.00)

[a] In parentheses is the gain in prediction accuracy with SG-UR over SG-SR; [b] In parentheses is the gain in prediction accuracy with UG-SR over SG-SR;

[c] In parentheses is the gain in prediction accuracy with UG-UR over UG-SR; Bold in parentheses indicates the number is not significant at α = 0.05.

Table S29 Accuracy of AP prediction for environment E3 with four ME GWP models in CV1

	LL				LW			
PopId	SG-SR	SG-UR[a]	UG-SR[b]	UG-UR[c]	SG-SR	SG-UR[a]	UG-SR[b]	UG-UR[c]
1	0.37	0.37(**0.00**)	0.38(0.01)	0.37(-0.01)	0.24	0.23(-0.04)	0.25(0.04)	0.25(**0.00**)
2	0.27	0.25(-0.09)	0.31(0.12)	0.30(-0.03)	0.46	0.45(-0.02)	0.47(0.03)	0.47(**0.00**)
3	0.22	0.23(0.04)	0.20(-0.11)	0.21(0.07)	0.37	0.37(**0.00**)	0.34(-0.07)	0.36(0.04)
4	0.42	0.42(**0.00**)	0.40(-0.04)	0.41(0.03)	0.22	0.21(-0.04)	0.24(0.08)	0.24(**0.00**)
5	0.20	0.19(-0.05)	0.21(0.07)	0.21(**0.00**)	0.44	0.44(**0.00**)	0.42(-0.03)	0.43(**0.01**)
6	0.47	0.46(-0.01)	0.46(**-0.01**)	0.46(**0.00**)	0.26	0.26(**0.00**)	0.27(**0.01**)	0.26(-0.02)
7	0.20	0.19(-0.03)	0.19(-0.06)	0.20(0.04)	0.32	0.31(-0.04)	0.34(0.06)	0.33(-0.02)
8	0.29	0.27(-0.04)	0.30(0.06)	0.29(-0.03)	0.27	0.27(**0.00**)	0.28(0.03)	0.28(**0.00**)
9	0.35	0.35(**0.00**)	0.35(**0.00**)	0.36(0.02)	0.31	0.32(0.02)	0.29(-0.09)	0.30(0.04)
10	0.47	0.46(-0.01)	0.45(-0.05)	0.46(0.04)	0.56	0.56(**0.00**)	0.55(-0.01)	0.55(**0.00**)
11	0.22	0.21(-0.06)	0.23(0.05)	0.23(**0.00**)	0.26	0.25(-0.02)	0.27(0.06)	0.27(**0.00**)
12	0.32	0.31(-0.02)	0.30(-0.04)	0.31(**0.01**)	0.34	0.34(**0.00**)	0.34(**0.00**)	0.34(**0.00**)
13	0.36	0.34(-0.06)	0.41(0.12)	0.39(-0.05)	0.40	0.40(**0.00**)	0.37(-0.08)	0.38(0.03)
14	0.24	0.24(**0.00**)	0.24(**0.00**)	0.24(**0.00**)	0.26	0.25(-0.05)	0.29(0.13)	0.28(-0.04)
15	0.24	0.23(-0.06)	0.26(0.06)	0.25(-0.01)	0.49	0.48(-0.01)	0.50(0.01)	0.50(**0.00**)
16	0.16	0.14(-0.09)	0.17(0.09)	0.17(**0.00**)	0.46	0.44(-0.04)	0.50(0.11)	0.49(-0.03)
17	0.29	0.28(-0.04)	0.29(**0.00**)	0.29(**0.00**)	0.30	0.28(-0.06)	0.34(0.13)	0.33(-0.03)
18	0.11	0.09(-0.14)	0.14(0.29)	0.13(-0.07)	0.32	0.32(**0.00**)	0.33(0.02)	0.33(**0.00**)
19	0.36	0.35(-0.01)	0.35(-0.02)	0.35(**0.00**)	0.29	0.28(-0.01)	0.30(0.05)	0.30(**0.00**)
20	0.36	0.34(-0.06)	0.38(0.05)	0.38(**0.00**)	0.44	0.42(-0.03)	0.46(0.06)	0.46(**0.00**)
21	0.44	0.42(-0.05)	0.48(0.08)	0.46(-0.03)	0.40	0.39(-0.02)	0.41(0.03)	0.41(**0.00**)
22	0.20	0.19(-0.08)	0.22(0.08)	0.21(-0.02)	0.26	0.25(-0.02)	0.27(0.04)	0.26(-0.01)
23	0.22	0.22(**0.00**)	0.22(**0.00**)	0.23(0.01)	0.27	0.26(-0.03)	0.28(0.05)	0.28(**0.00**)
24	0.19	0.19(**0.00**)	0.18(-0.04)	0.19(0.04)	0.36	0.35(-0.03)	0.39(0.08)	0.38(-0.03)
25	0.39	0.40(0.01)	0.37(-0.06)	0.38(0.02)	0.24	0.23(-0.02)	0.23(-0.04)	0.24(0.03)
Mean	0.29	0.29(0.00)	0.30(0.01)	0.30(0.00)	0.34	0.33(-0.02)	0.35(0.03)	0.35(0.00)

[a] In parentheses is the gain in prediction accuracy with SG-UR over SG-SR; [b] In parentheses is the gain in prediction accuracy with UG-SR over SG-SR;

[c] In parentheses is the gain in prediction accuracy with UG-UR over UG-SR; Bold in parentheses indicates the number is not significant at $\alpha = 0.05$.

Table S30 Accuracy of AP prediction for environment E4 with four ME GWP models in CV1

	LL				LW			
PopId	SG-SR	SG-UR[a]	UG-SR[b]	UG-UR[c]	SG-SR	SG-UR[a]	UG-SR[b]	UG-UR[c]
1	0.26	0.26(**0.00**)	0.26(**0.00**)	0.27(0.03)	0.31	0.30(-0.03)	0.31(**0.00**)	0.31(**0.00**)
2	0.25	0.24(-0.03)	0.23(-0.09)	0.23(**0.00**)	0.43	0.42(-0.01)	0.43(**0.00**)	0.43(**0.00**)
3	0.24	0.23(-0.06)	0.24(**0.00**)	0.25(0.02)	0.39	0.39(**0.00**)	0.39(**0.00**)	0.40(0.03)
4	0.42	0.42(**0.00**)	0.43(0.02)	0.43(**0.00**)	0.29	0.29(**0.00**)	0.30(0.03)	0.29(-0.02)
5	0.38	0.38(**0.00**)	0.36(-0.05)	0.38(0.04)	0.33	0.32(-0.04)	0.36(0.08)	0.36(**0.00**)
6	0.46	0.44(-0.03)	0.48(0.06)	0.48(**0.00**)	0.33	0.32(-0.02)	0.33(**0.00**)	0.33(**0.00**)
7	0.32	0.31(-0.03)	0.30(-0.06)	0.31(0.04)	0.52	0.51(-0.01)	0.53(0.02)	0.53(**0.00**)
8	0.34	0.32(-0.05)	0.37(0.07)	0.35(-0.04)	0.39	0.39**(0.00)**	0.40(0.02)	0.40(**0.00**)
9	0.35	0.35(**0.00**)	0.35(**0.00**)	0.36(0.02)	0.40	0.39(-0.04)	0.42(0.04)	0.41(-0.01)
10	0.46	0.44(-0.04)	0.47(0.02)	0.48(0.02)	0.53	0.52(-0.02)	0.55(0.02)	0.54(-0.01)
11	0.27	0.27(**0.00**)	0.27(**0.00**)	0.27(**0.00**)	0.36	0.36(**0.00**)	0.35(-0.03)	0.36(0.02)
12	0.31	0.28(-0.10)	0.35(0.13)	0.34(-0.03)	0.57	0.57(**0.00**)	0.58(0.01)	0.58(**0.00**)
13	0.16	0.14(-0.11)	0.18(0.14)	0.16(-0.08)	0.32	0.29(-0.07)	0.35(0.11)	0.35(**0.00**)
14	0.35	0.35(**0.00**)	0.34(-0.04)	0.33(-0.01)	0.33	0.33(**0.00**)	0.33(**0.00**)	0.33(**0.00**)
15	0.20	0.19(-0.07)	0.20(**0.00**)	0.21(0.02)	0.60	0.59(-0.00)	0.60(**0.00**)	0.60(**0.00**)
16	0.23	0.21(-0.07)	0.25(0.11)	0.24(-0.04)	0.57	0.56(-0.02)	0.58(0.02)	0.58(**0.00**)
17	0.25	0.25(**0.00**)	0.24(-0.06)	0.24(**0.00**)	0.53	0.53(**0.00**)	0.52(-0.01)	0.53(0.01)
18	0.13	0.12(-0.03)	0.13(**0.00**)	0.13(**0.00**)	0.38	0.38(**0.00**)	0.37(-0.02)	0.38(0.02)
19	0.19	0.18(-0.01)	0.19(**0.00**)	0.19(**0.00**)	0.36	0.37(0.01)	0.36(**0.00**)	0.36(**0.00**)
20	0.41	0.39(-0.04)	0.42(0.02)	0.41(-0.01)	0.52	0.51(-0.01)	0.53(0.03)	0.52(-0.01)
21	0.52	0.52(**0.00**)	0.51(-0.03)	0.52(0.01)	0.41	0.39(-0.07)	0.45(0.09)	0.44(-0.04)
22	0.25	0.25(**0.00**)	0.23(-0.05)	0.24(0.03)	0.40	0.40(**0.00**)	0.40(**0.00**)	0.40(**0.00**)
23	0.13	0.13(**0.00**)	0.12(-0.06)	0.13(0.07)	0.30	0.29(-0.01)	0.30(**0.00**)	0.30(**0.00**)
24	0.29	0.30(0.04)	0.26(-0.10)	0.28(0.06)	0.50	0.49(-0.02)	0.50(**0.00**)	0.50(**0.00**)
25	0.29	0.27(-0.07)	0.31(0.06)	0.30(-0.01)	0.44	0.45(0.01)	0.42(-0.05)	0.43(0.03)
Mean	0.30	0.29(-0.03)	0.30(0.00)	0.30(0.00)	0.42	0.41(-0.02)	0.43(0.01)	0.43(0.00)

[a] In parentheses is the gain in prediction accuracy with SG-UR over SG-SR; [b] In parentheses is the gain in prediction accuracy with UG-SR over SG-SR;

[c] In parentheses is the gain in prediction accuracy with UG-UR over UG-SR; Bold in parentheses indicates the number is not significant at α = 0.05.

Table S31 Accuracy of WP prediction for environment E1 with four ME GWP models in CV2

	LL				LW			
PopId	SG-SR	SG-UR[a]	UG-SR[b]	UG-UR[c]	SG-SR	SG-UR[a]	UG-SR[b]	UG-UR[c]
1	0.33	0.30(-0.08)	0.45(0.40)	0.45(**0.00**)	0.31	0.29(-0.07)	0.49(0.59)	0.49(**0.00**)
2	0.18	0.16(-0.08)	0.41(1.35)	0.42(**0.01**)	0.40	0.38(-0.04)	0.55(0.39)	0.55(**0.00**)
3	0.26	0.25(**-0.02**)	0.36(0.42)	0.37(**0.01**)	0.56	0.55(-0.02)	0.68(0.21)	0.68(**0.00**)
4	0.49	0.47(-0.03)	0.60(0.24)	0.60(**0.00**)	0.50	0.49(-0.02)	0.61(0.22)	0.61(**0.00**)
5	0.33	0.31(-0.06)	0.53(0.60)	0.54(0.01)	0.21	0.19(-0.08)	0.47(1.26)	0.46(-0.01)
6	0.50	0.49(-0.03)	0.59(0.18)	0.59(**0.00**)	0.31	0.30(-0.04)	0.43(0.40)	0.43(**0.00**)
7	0.52	0.51(-0.02)	0.67(0.30)	0.67(**0.00**)	0.48	0.47(-0.02)	0.61(0.28)	0.61(**0.00**)
8	0.28	0.28(**0.00**)	0.52(0.81)	0.51(**-0.01**)	0.30	0.27(-0.08)	0.49(0.64)	0.49(**0.00**)
9	0.35	0.33(-0.06)	0.50(0.43)	0.50(**0.00**)	0.18	0.16(-0.09)	0.42(1.37)	0.42(**0.00**)
10	0.46	0.47(**0.01**)	0.64(0.38)	0.64(**0.00**)	0.37	0.35(-0.04)	0.62(0.69)	0.62(**0.00**)
11	0.40	0.38(-0.04)	0.56(0.39)	0.56(**0.00**)	0.40	0.39(-0.01)	0.48(0.21)	0.48(**0.00**)
12	0.50	0.49(-0.02)	0.65(0.31)	0.65(**0.00**)	0.54	0.54(**0.00**)	0.64(0.18)	0.64(**0.00**)
13	0.45	0.45(**0.00**)	0.65(0.43)	0.64(**-0.01**)	0.49	0.48(-0.02)	0.62(0.26)	0.61(-0.01)
14	0.36	0.35(-0.03)	0.56(0.56)	0.55(-0.01)	0.44	0.44(**0.00**)	0.62(0.40)	0.62(**0.00**)
15	0.28	0.25(-0.11)	0.48(0.72)	0.47(-0.02)	0.30	0.29(-0.03)	0.44(0.45)	0.43(**-0.01**)
16	0.34	0.32(-0.05)	0.48(0.43)	0.48(**0.00**)	0.45	0.44(-0.02)	0.56(0.23)	0.56(**0.00**)
17	0.27	0.26(-0.03)	0.40(0.45)	0.39(**-0.01**)	0.55	0.55(**0.00**)	0.68(0.24)	0.68(**0.00**)
18	0.27	0.27(**0.00**)	0.36(0.31)	0.36(**0.00**)	0.40	0.40(**0.00**)	0.47(0.16)	0.47(**0.00**)
19	0.37	0.35(-0.06)	0.53(0.45)	0.52(-0.03)	0.41	0.40(-0.02)	0.58(0.42)	0.58(**0.00**)
20	0.47	0.45(-0.05)	0.61(0.30)	0.61(**0.00**)	0.48	0.48(**0.00**)	0.59(0.24)	0.60(0.01)
21	0.48	0.47(**-0.01**)	0.60(0.26)	0.61(0.01)	0.47	0.47(**0.00**)	0.53(0.14)	0.54(0.01)
22	0.36	0.34(-0.06)	0.51(0.42)	0.51(**0.00**)	0.31	0.30(-0.05)	0.47(0.52)	0.47(**0.00**)
23	0.44	0.42(-0.04)	0.55(0.25)	0.54(-0.01)	0.30	0.30(**0.00**)	0.54(0.80)	0.54(**0.00**)
24	0.48	0.47(-0.03)	0.58(0.21)	0.57(-0.02)	0.50	0.49(-0.02)	0.64(0.29)	0.64(**0.00**)
25	0.50	0.48(-0.03)	0.61(0.24)	0.61(**0.00**)	0.47	0.46(-0.01)	0.61(0.30)	0.60(-0.01)
Mean	0.39	0.37(-0.04)	0.54(0.39)	0.53(-0.01)	0.41	0.39(-0.02)	0.55(0.36)	0.55(0.00)

[a] In parentheses is the gain in prediction accuracy with SG-UR over SG-SR; [b] In parentheses is the gain in prediction accuracy with UG-SR over SG-SR;

[c] In parentheses is the gain in prediction accuracy with UG-UR over UG-SR; Bold in parentheses indicates the number is not significant at $\alpha = 0.05$.

Table S32 Accuracy of WP prediction for environment E2 with four ME GWP models in CV2

	LL				LW			
PopId	SG-SR	SG-UR[a]	UG-SR[b]	UG-UR[c]	SG-SR	SG-UR[a]	UG-SR[b]	UG-UR[c]
1	0.67	0.67(**0.00**)	0.74(0.11)	0.75(**0.00**)	0.46	0.45(-0.02)	0.57(0.25)	0.57(**0.00**)
2	0.40	0.39(-0.03)	0.57(0.42)	0.56(-0.02)	0.48	0.47(-0.02)	0.64(0.33)	0.64(**0.00**)
3	0.20	0.19(-0.05)	0.31(0.56)	0.30(**-0.01**)	0.60	0.59(-0.01)	0.70(0.17)	0.70(**0.00**)
4	0.56	0.56(**0.00**)	0.68(0.21)	0.68(**0.00**)	0.61	0.60(-0.01)	0.70(0.16)	0.70(**0.00**)
5	0.50	0.50(**0.00**)	0.64(0.28)	0.64(**0.00**)	0.46	0.44(-0.03)	0.65(0.43)	0.64(-0.02)
6	0.47	0.46(-0.03)	0.58(0.23)	0.58(**0.00**)	0.31	0.31(**0.00**)	0.36(0.17)	0.37(0.01)
7	0.41	0.39(-0.04)	0.62(0.53)	0.62(**0.00**)	0.59	0.57(-0.02)	0.72(0.22)	0.72(**0.00**)
8	0.34	0.31(-0.08)	0.53(0.55)	0.51(-0.03)	0.36	0.34(-0.06)	0.53(0.47)	0.53(**0.00**)
9	0.33	0.32(-0.04)	0.46(0.38)	0.46(**0.00**)	0.47	0.46(-0.02)	0.54(0.15)	0.54(**0.00**)
10	0.51	0.50(-0.02)	0.66(0.30)	0.65(-0.01)	0.36	0.32(-0.09)	0.47(0.32)	0.46(-0.01)
11	0.52	0.51(-0.02)	0.62(0.19)	0.62(**0.00**)	0.41	0.40(-0.01)	0.54(0.34)	0.54(**0.00**)
12	0.55	0.53(-0.03)	0.69(0.25)	0.68(-0.01)	0.58	0.57(-0.01)	0.65(0.13)	0.65(**0.00**)
13	0.39	0.37(-0.05)	0.66(0.67)	0.65(-0.01)	0.42	0.40(-0.06)	0.62(0.46)	0.60(-0.02)
14	0.42	0.41(-0.04)	0.60(0.41)	0.58(-0.03)	0.25	0.23(-0.07)	0.46(0.82)	0.45(-0.02)
15	0.48	0.48(**0.00**)	0.64(0.34)	0.65(0.01)	0.55	0.54(-0.01)	0.68(0.24)	0.67(-0.01)
16	0.33	0.31(-0.05)	0.47(0.43)	0.46(-0.01)	0.55	0.55(**0.00**)	0.64(0.16)	0.64(**0.00**)
17	0.12	0.10(-0.14)	0.29(1.44)	0.29(**0.00**)	0.61	0.61(**0.00**)	0.73(0.18)	0.73(**0.00**)
18	0.27	0.24(-0.10)	0.41(0.54)	0.40(-0.02)	0.31	0.30(-0.04)	0.50(0.60)	0.50(**0.00**)
19	0.27	0.24(-0.11)	0.41(0.51)	0.40(-0.03)	0.41	0.39(-0.03)	0.57(0.39)	0.57(**0.00**)
20	0.52	0.51(-0.02)	0.66(0.27)	0.66(**0.00**)	0.54	0.53(-0.01)	0.69(0.29)	0.69(**0.00**)
21	0.58	0.56(-0.02)	0.71(0.23)	0.70(-0.01)	0.33	0.33(**0.00**)	0.45(0.34)	0.44(-0.01)
22	0.50	0.49(-0.01)	0.61(0.21)	0.60(-0.01)	0.59	0.58(-0.01)	0.68(0.15)	0.68(**0.00**)
23	0.43	0.41(-0.04)	0.54(0.24)	0.53(-0.01)	0.37	0.37(**0.00**)	0.49(0.32)	0.49(**0.00**)
24	0.16	0.13(-0.17)	0.45(1.80)	0.44(-0.03)	0.45	0.44(-0.02)	0.63(0.39)	0.62(-0.01)
25	0.40	0.38(-0.05)	0.58(0.46)	0.57(-0.02)	0.48	0.48(**0.00**)	0.54(0.12)	0.54(**0.00**)
Mean	0.41	0.40(-0.03)	0.56(0.35)	0.56(0.00)	0.46	0.45(-0.02)	0.59(0.27)	0.59(0.00)

[a] In parentheses is the gain in prediction accuracy with SG-UR over SG-SR; [b] In parentheses is the gain in prediction accuracy with UG-SR over SG-SR;

[c] In parentheses is the gain in prediction accuracy with UG-UR over UG-SR; Bold in parentheses indicates the number is not significant at α = 0.05.

Table S33 Accuracy of WP prediction for environment E3 with four ME GWP models in CV2

	LL				LW			
PopId	SG-SR	SG-UR[a]	UG-SR[b]	UG-UR[c]	SG-SR	SG-UR[a]	UG-SR[b]	UG-UR[c]
1	0.51	0.50(-0.02)	0.65(0.27)	0.65(**0.00**)	0.36	0.35(-0.03)	0.45(0.25)	0.45(**0.00**)
2	0.36	0.34(-0.06)	0.52(0.46)	0.51(-0.03)	0.52	0.52(**0.00**)	0.65(0.23)	0.65(**0.00**)
3	0.23	0.22(-0.04)	0.35(0.48)	0.35(**0.00**)	0.26	0.25(-0.04)	0.46(0.75)	0.46(**0.00**)
4	0.51	0.49(-0.04)	0.61(0.19)	0.60(-0.01)	0.19	0.17(-0.10)	0.39(1.01)	0.38(-0.01)
5	0.46	0.44(-0.03)	0.60(0.32)	0.60(**0.00**)	0.30	0.28(-0.09)	0.53(0.76)	0.52(-0.02)
6	0.47	0.44(-0.06)	0.59(0.26)	0.59(**0.00**)	0.31	0.31(**0.00**)	0.43(0.37)	0.43(**0.00**)
7	0.49	0.48(-0.03)	0.64(0.30)	0.64(**0.00**)	0.42	0.41(-0.03)	0.59(0.41)	0.59(**0.00**)
8	0.32	0.31(-0.04)	0.50(0.54)	0.50(**0.00**)	0.46	0.44(-0.03)	0.54(0.18)	0.54(**0.00**)
9	0.42	0.40(-0.06)	0.54(0.28)	0.54(**0.00**)	0.45	0.44(-0.02)	0.57(0.26)	0.57(**0.00**)
10	0.42	0.40(-0.05)	0.62(0.47)	0.62(**0.00**)	0.55	0.54(-0.03)	0.63(0.14)	0.62(-0.01)
11	0.53	0.52(-0.02)	0.63(0.20)	0.63(**0.00**)	0.38	0.38(**0.00**)	0.52(0.37)	0.52(**0.00**)
12	0.42	0.39(-0.05)	0.56(0.34)	0.56(**0.00**)	0.27	0.26(-0.05)	0.41(0.51)	0.41(**0.00**)
13	0.43	0.39(-0.08)	0.64(0.50)	0.61(-0.04)	0.27	0.23(-0.13)	0.52(0.98)	0.51(-0.02)
14	0.33	0.31(-0.06)	0.48(0.46)	0.48(**0.00**)	0.33	0.31(-0.06)	0.53(0.59)	0.53(**0.00**)
15	0.25	0.22(-0.10)	0.46(0.83)	0.46(**0.00**)	0.39	0.39(**0.00**)	0.57(0.46)	0.57(**0.00**)
16	0.33	0.30(-0.07)	0.43(0.32)	0.43(**0.00**)	0.36	0.35(-0.02)	0.52(0.45)	0.52(**0.00**)
17	0.32	0.29(-0.07)	0.44(0.40)	0.44(**0.00**)	0.37	0.36(**-0.01**)	0.54(0.47)	0.54(**0.00**)
18	0.18	0.16(-0.11)	0.28(0.51)	0.28(**0.00**)	0.35	0.34(-0.02)	0.47(0.35)	0.47(**0.00**)
19	0.46	0.44(-0.05)	0.58(0.26)	0.57(-0.02)	0.41	0.40(-0.02)	0.54(0.31)	0.54(**0.00**)
20	0.32	0.29(-0.11)	0.50(0.56)	0.49(-0.01)	0.44	0.43(-0.02)	0.58(0.31)	0.58(**0.00**)
21	0.47	0.45(-0.03)	0.63(0.35)	0.63(**0.00**)	0.41	0.40(-0.01)	0.50(0.24)	0.50(**0.00**)
22	0.39	0.38(-0.02)	0.52(0.34)	0.51(-0.01)	0.34	0.33(-0.03)	0.52(0.53)	0.52(**0.00**)
23	0.31	0.28(-0.09)	0.50(0.60)	0.50(**0.00**)	0.40	0.40(**0.00**)	0.52(0.30)	0.52(**0.00**)
24	0.26	0.23(-0.11)	0.43(0.68)	0.41(-0.04)	0.41	0.39(-0.03)	0.56(0.38)	0.57(0.01)
25	0.36	0.32(-0.11)	0.52(0.44)	0.50(-0.04)	0.29	0.28(-0.03)	0.44(0.52)	0.44(**0.00**)
Mean	0.38	0.36(-0.06)	0.53(0.38)	0.52(-0.01)	0.37	0.36(-0.03)	0.52(0.40)	0.52(0.00)

[a] In parentheses is the gain in prediction accuracy with SG-UR over SG-SR; [b] In parentheses is the gain in prediction accuracy with UG-SR over SG-SR;

[c] In parentheses is the gain in prediction accuracy with UG-UR over UG-SR; Bold in parentheses indicates the number is not significant at $\alpha = 0.05$.

Table S34 Accuracy of WP prediction for environment E4 with four ME GWP models in CV2

	LL				LW			
PopId	SG-SR	SG-UR[a]	UG-SR[b]	UG-UR[c]	SG-SR	SG-UR[a]	UG-SR[b]	UG-UR[c]
1	0.46	0.45(-0.01)	0.61(0.33)	0.61(**0.00**)	0.51	0.50(-0.02)	0.63(0.23)	0.62(-0.01)
2	0.30	0.30(**0.00**)	0.53(0.77)	0.52(-0.02)	0.46	0.45(-0.02)	0.62(0.35)	0.62(**0.00**)
3	0.27	0.26(-0.03)	0.37(0.35)	0.36(**-0.01**)	0.41	0.40(-0.02)	0.62(0.52)	0.61(-0.01)
4	0.53	0.52(-0.02)	0.66(0.23)	0.65(-0.01)	0.52	0.51(-0.02)	0.64(0.23)	0.63(-0.01)
5	0.37	0.37(**0.00**)	0.55(0.47)	0.55(**0.00**)	0.44	0.43(-0.03)	0.63(0.43)	0.62(-0.02)
6	0.47	0.44(-0.06)	0.59(0.27)	0.59(**0.00**)	0.31	0.30(-0.02)	0.46(0.49)	0.46(**0.00**)
7	0.60	0.59(-0.01)	0.73(0.21)	0.73(**0.00**)	0.61	0.61(**0.00**)	0.72(0.18)	0.72(**0.00**)
8	0.37	0.35(-0.05)	0.59(0.60)	0.59(**0.00**)	0.40	0.37(-0.07)	0.56(0.42)	0.57(0.02)
9	0.33	0.31(-0.06)	0.49(0.47)	0.48(-0.01)	0.48	0.47(-0.02)	0.52(0.08)	0.51(-0.01)
10	0.42	0.41(-0.03)	0.58(0.39)	0.57(-0.01)	0.62	0.62(**0.00**)	0.73(0.17)	0.72(-0.01)
11	0.29	0.27(-0.08)	0.49(0.67)	0.49(**0.00**)	0.46	0.45(-0.01)	0.60(0.31)	0.60(**0.00**)
12	0.50	0.47(-0.05)	0.65(0.29)	0.64(-0.01)	0.57	0.56(-0.01)	0.66(0.17)	0.66(**0.00**)
13	0.24	0.19(-0.18)	0.52(1.21)	0.54(0.02)	0.42	0.39(-0.06)	0.60(0.44)	0.60(**0.00**)
14	0.35	0.32(-0.07)	0.54(0.55)	0.53(-0.03)	0.44	0.43(-0.02)	0.63(0.44)	0.63(**0.00**)
15	0.37	0.35(-0.04)	0.58(0.57)	0.57(-0.01)	0.54	0.53(-0.01)	0.68(0.26)	0.68(**0.00**)
16	0.21	0.19(-0.09)	0.42(0.99)	0.42(**0.00**)	0.54	0.53(-0.00)	0.66(0.23)	0.66(**0.00**)
17	0.35	0.34(-0.03)	0.49(0.38)	0.48(-0.02)	0.56	0.56(**0.00**)	0.70(0.25)	0.70(**0.00**)
18	0.26	0.24(-0.08)	0.37(0.40)	0.37(**0.00**)	0.33	0.32(-0.02)	0.45(0.39)	0.45(**0.00**)
19	0.28	0.25(-0.11)	0.45(0.61)	0.45(**0.00**)	0.50	0.48(-0.03)	0.66(0.31)	0.66(**0.00**)
20	0.47	0.46(-0.03)	0.60(0.27)	0.59(-0.01)	0.53	0.54(0.01)	0.66(0.25)	0.67(0.01)
21	0.61	0.60(-0.02)	0.69(0.13)	0.69(**0.00**)	0.36	0.36(**0.00**)	0.49(0.35)	0.49(**0.00**)
22	0.46	0.46(**0.00**)	0.55(0.18)	0.55(**0.00**)	0.54	0.53(-0.02)	0.67(0.26)	0.67(**0.00**)
23	0.39	0.37(-0.05)	0.50(0.28)	0.50(**0.00**)	0.48	0.48(**0.00**)	0.60(0.25)	0.60(**0.00**)
24	0.23	0.20(-0.14)	0.43(0.87)	0.41(-0.04)	0.46	0.45(-0.02)	0.62(0.35)	0.62(**0.00**)
25	0.24	0.21(-0.14)	0.46(0.87)	0.45(-0.02)	0.49	0.48(-0.02)	0.54(0.11)	0.54(**0.00**)
Mean	0.38	0.36(-0.05)	0.54(0.43)	0.53(-0.01)	0.48	0.47(-0.02)	0.61(0.28)	0.61(0.00)

[a] In parentheses is the gain in prediction accuracy with SG-UR over SG-SR; [b] In parentheses is the gain in prediction accuracy with UG-SR over SG-SR;

[c] In parentheses is the gain in prediction accuracy with UG-UR over UG-SR; Bold in parentheses indicates the number is not significant at α = 0.05.

Table S35 Accuracy of AP prediction for environment E1 with four ME GWP models in CV2

	LL				LW			
PopId	SG-SR	SG-UR[a]	UG-SR[b]	UG-UR[c]	SG-SR	SG-UR[a]	UG-SR[b]	UG-UR[c]
1	0.31	0.30(-0.01)	0.36(0.16)	0.35(-0.01)	0.27	0.26(**-0.01**)	0.30(0.14)	0.30(**0.00**)
2	0.17	0.16(-0.05)	0.23(0.36)	0.22(-0.04)	0.41	0.42(0.01)	0.44(0.06)	0.44(**0.00**)
3	0.25	0.26(0.05)	0.27(0.09)	0.28(0.03)	0.55	0.54(**-0.01**)	0.59(0.07)	0.59(**0.00**)
4	0.45	0.46(0.02)	0.46(0.03)	0.48(0.03)	0.33	0.34(0.03)	0.33(**0.00**)	0.33(**0.00**)
5	0.26	0.27(0.07)	0.27(0.06)	0.28(0.04)	0.24	0.24(**0.00**)	0.28(0.18)	0.28(**0.00**)
6	0.47	0.47(**0.00**)	0.50(0.06)	0.51(0.01)	0.38	0.38(**0.00**)	0.41(0.07)	0.41(**0.00**)
7	0.29	0.28(-0.03)	0.37(0.25)	0.36(-0.02)	0.37	0.37(**0.00**)	0.45(0.22)	0.44(-0.02)
8	0.44	0.44(**0.00**)	0.49(0.10)	0.49(**0.00**)	0.37	0.37(**0.00**)	0.40(0.08)	0.40(**0.00**)
9	0.35	0.34(-0.01)	0.39(0.12)	0.39(**0.00**)	0.16	0.17(0.02)	0.18(0.11)	0.18(**0.00**)
10	0.39	0.39(**0.00**)	0.44(0.12)	0.44(**0.00**)	0.44	0.43(-0.01)	0.50(0.15)	0.50(**0.00**)
11	0.14	0.14(**0.00**)	0.20(0.43)	0.20(**0.00**)	0.21	0.21(**0.00**)	0.24(0.13)	0.24(**0.00**)
12	0.40	0.40(**0.00**)	0.46(0.14)	0.46(**0.00**)	0.57	0.56(-0.01)	0.60(0.06)	0.60(**0.00**)
13	0.28	0.27(-0.04)	0.39(0.36)	0.38(-0.02)	0.40	0.40(**0.00**)	0.44(0.11)	0.44(**0.00**)
14	0.40	0.41(**0.01**)	0.44(0.08)	0.44(**0.00**)	0.36	0.36(**0.00**)	0.42(0.17)	0.42(**0.00**)
15	0.25	0.27(0.04)	0.27(0.05)	0.27(**0.00**)	0.33	0.33(**0.00**)	0.34(0.05)	0.34(**0.00**)
16	0.30	0.30(**0.00**)	0.34(0.11)	0.34(**0.00**)	0.51	0.51(**0.00**)	0.53(0.04)	0.53(**0.00**)
17	0.29	0.29(**0.00**)	0.33(0.13)	0.33(**0.00**)	0.46	0.45(**-0.01**)	0.50(0.11)	0.51(0.00)
18	0.15	0.16(0.05)	0.16(0.07)	0.17(0.04)	0.30	0.30(**0.00**)	0.34(0.13)	0.33(-0.02)
19	0.32	0.32(**0.00**)	0.37(0.14)	0.37(**0.00**)	0.26	0.26(**0.00**)	0.29(0.15)	0.29(**0.00**)
20	0.42	0.41(**-0.01**)	0.45(0.07)	0.45(**0.00**)	0.44	0.44(**0.00**)	0.47(0.07)	0.47(**0.00**)
21	0.33	0.32(-0.04)	0.40(0.22)	0.39(-0.02)	0.32	0.31(-0.02)	0.36(0.13)	0.35(-0.02)
22	0.34	0.35(0.03)	0.36(0.05)	0.36(**0.00**)	0.31	0.31(**0.00**)	0.34(0.10)	0.34(**0.00**)
23	0.22	0.23(0.03)	0.25(0.12)	0.25(**0.00**)	0.17	0.16(-0.05)	0.25(0.47)	0.24(-0.03)
24	0.32	0.33(0.06)	0.31(-0.02)	0.32(0.04)	0.47	0.48(0.01)	0.50(0.05)	0.50(**0.00**)
25	0.43	0.44(0.01)	0.44(0.03)	0.46(0.03)	0.48	0.47(-0.01)	0.51(0.07)	0.51(**0.00**)
Mean	0.32	0.32(0.00)	0.36(0.12)	0.36(0.00)	0.36	0.36(0.00)	0.40(0.10)	0.40(0.00)

[a] In parentheses is the gain in prediction accuracy with SG-UR over SG-SR; [b] In parentheses is the gain in prediction accuracy with UG-SR over SG-SR;

[c] In parentheses is the gain in prediction accuracy with UG-UR over UG-SR; Bold in parentheses indicates the number is not significant at α = 0.05.

Table S36 Accuracy of AP prediction for environment E2 with four ME GWP models in CV2

	LL				LW			
PopId	SG-SR	SG-UR[a]	UG-SR[b]	UG-UR[c]	SG-SR	SG-UR[a]	UG-SR[b]	UG-UR[c]
1	0.45	0.46(0.02)	0.46(0.02)	0.47(0.02)	0.25	0.24(-0.02)	0.30(0.21)	0.30(**0.00**)
2	0.30	0.29(-0.03)	0.36(0.20)	0.36(**0.00**)	0.45	0.45(**0.00**)	0.50(0.11)	0.50(**0.00**)
3	0.30	0.31(0.04)	0.31(0.06)	0.32(0.02)	0.56	0.56(**0.00**)	0.60(0.06)	0.60(**0.00**)
4	0.42	0.42(**0.00**)	0.46(0.09)	0.46(**0.00**)	0.38	0.38(**0.00**)	0.42(0.12)	0.42(**0.00**)
5	0.33	0.34(0.03)	0.36(0.09)	0.37(0.02)	0.47	0.46(-0.01)	0.52(0.11)	0.52(**0.00**)
6	0.46	0.47(0.01)	0.48(0.04)	0.49(0.01)	0.28	0.28(**0.00**)	0.30(0.10)	0.30(**0.00**)
7	0.23	0.23(**0.00**)	0.28(0.21)	0.28(**0.00**)	0.60	0.60(**0.00**)	0.61(0.02)	0.62(0.02)
8	0.27	0.26(-0.02)	0.32(0.18)	0.32(**0.00**)	0.36	0.36(**0.00**)	0.38(0.07)	0.38(**0.00**)
9	0.21	0.22(0.03)	0.23(0.11)	0.24(**0.01**)	0.36	0.36(**0.00**)	0.40(0.11)	0.39(-0.01)
10	0.49	0.48(**-0.01**)	0.51(0.06)	0.52(0.01)	0.42	0.41(-0.02)	0.48(0.15)	0.48(**0.00**)
11	0.34	0.33(-0.02)	0.39(0.15)	0.39(**0.00**)	0.37	0.38(0.01)	0.40(0.08)	0.40(**0.00**)
12	0.42	0.42(**0.00**)	0.46(0.09)	0.46(**0.00**)	0.60	0.60(-0.01)	0.63(0.04)	0.63(**0.00**)
13	0.36	0.37(0.03)	0.39(0.07)	0.40(0.03)	0.41	0.42(0.01)	0.43(0.05)	0.44(0.01)
14	0.27	0.26(-0.03)	0.33(0.24)	0.33(**0.00**)	0.38	0.37(-0.02)	0.42(0.11)	0.41(-0.01)
15	0.33	0.35(0.06)	0.35(0.07)	0.37(0.05)	0.54	0.54(**0.00**)	0.55(0.02)	0.55(**0.00**)
16	0.41	0.41(**0.00**)	0.44(0.07)	0.45(0.02)	0.54	0.54(**0.00**)	0.56(0.03)	0.56(**0.00**)
17	0.10	0.10(**0.00**)	0.14(0.42)	0.14(**0.00**)	0.61	0.61(**0.00**)	0.61(**0.00**)	0.62(0.01)
18	0.20	0.21(0.05)	0.21(0.05)	0.22(0.05)	0.26	0.26(**0.00**)	0.27(0.03)	0.27(**0.00**)
19	0.40	0.39(-0.02)	0.43(0.09)	0.43(**0.00**)	0.42	0.43(0.02)	0.43(0.03)	0.44(0.01)
20	0.42	0.42(**0.00**)	0.44(0.05)	0.44(**0.00**)	0.44	0.45(0.01)	0.46(0.03)	0.46(**0.00**)
21	0.48	0.47(-0.01)	0.51(0.07)	0.51(**0.00**)	0.44	0.44(**0.00**)	0.45(0.03)	0.45(**0.00**)
22	0.44	0.44(**0.00**)	0.47(0.08)	0.47(**0.00**)	0.43	0.44(0.01)	0.46(0.06)	0.47(0.01)
23	0.36	0.37(0.02)	0.38(0.05)	0.39(0.02)	0.39	0.39(**0.00**)	0.42(0.08)	0.42(**0.00**)
24	0.21	0.21(**0.00**)	0.25(0.22)	0.25(**0.00**)	0.49	0.49(**0.00**)	0.50(0.03)	0.51(0.02)
25	0.27	0.27(**0.00**)	0.31(0.18)	0.31(**0.00**)	0.37	0.36(-0.01)	0.43(0.16)	0.42(-0.01)
Mean	0.34	0.34(0.00)	0.37(0.10)	0.37(0.00)	0.43	0.42(-0.01)	0.46(0.07)	0.46(0.00)

[a] In parentheses is the gain in prediction accuracy with SG-UR over SG-SR; [b] In parentheses is the gain in prediction accuracy with UG-SR over SG-SR;

[c] In parentheses is the gain in prediction accuracy with UG-UR over UG-SR; Bold in parentheses indicates the number is not significant at α = 0.05.

Table S37 Accuracy of AP prediction for environment E3 with four ME GWP models in CV2

	LL				LW			
PopId	SG-SR	SG-UR[a]	UG-SR[b]	UG-UR[c]	SG-SR	SG-UR[a]	UG-SR[b]	UG-UR[c]
1	0.41	0.41(**0.00**)	0.45(0.11)	0.45(**0.00**)	0.23	0.23(**0.00**)	0.27(0.15)	0.27(**0.00**)
2	0.29	0.28(-0.03)	0.36(0.22)	0.35(-0.01)	0.47	0.47(**0.00**)	0.52(0.10)	0.52(**0.00**)
3	0.21	0.22(0.04)	0.22(0.05)	0.23(0.02)	0.36	0.36(**0.00**)	0.38(0.05)	0.38(**0.00**)
4	0.44	0.45(0.02)	0.45(0.03)	0.46(0.02)	0.22	0.22(**0.00**)	0.25(0.16)	0.26(0.01)
5	0.22	0.22(**0.00**)	0.27(0.23)	0.27(**0.00**)	0.44	0.44(**0.00**)	0.46(0.04)	0.46(**0.00**)
6	0.48	0.49(**0.01**)	0.50(0.03)	0.50(**0.00**)	0.26	0.25(-0.01)	0.27(0.07)	0.27(**0.00**)
7	0.25	0.25(**0.00**)	0.27(0.07)	0.28(0.03)	0.32	0.32(**0.00**)	0.38(0.17)	0.37(-0.02)
8	0.28	0.28(**0.00**)	0.32(0.13)	0.32(**0.00**)	0.29	0.29(**0.00**)	0.31(0.08)	0.31(**0.00**)
9	0.36	0.37(0.01)	0.39(0.07)	0.39(**0.00**)	0.33	0.33(**0.00**)	0.34(0.03)	0.34(**0.00**)
10	0.48	0.48(**0.00**)	0.50(0.04)	0.51(0.01)	0.55	0.56(0.01)	0.57(0.03)	0.57(**0.00**)
11	0.27	0.27(**0.00**)	0.31(0.16)	0.31(**0.00**)	0.27	0.27(**0.00**)	0.31(0.14)	0.31(**0.00**)
12	0.32	0.32(**0.00**)	0.34(0.07)	0.35(0.01)	0.32	0.33(0.02)	0.32(**0.00**)	0.33(0.01)
13	0.38	0.37(-0.02)	0.47(0.24)	0.46(-0.02)	0.40	0.41(0.02)	0.41(**0.00**)	0.42(0.02)
14	0.26	0.26(**0.00**)	0.29(0.14)	0.30(**0.01**)	0.26	0.26(**0.00**)	0.33(0.23)	0.32(-0.02)
15	0.25	0.25(**0.00**)	0.31(0.24)	0.31(**0.00**)	0.49	0.48(-0.00)	0.52(0.06)	0.52(**0.00**)
16	0.20	0.19(**-0.02**)	0.24(0.20)	0.24(**0.00**)	0.46	0.45(-0.02)	0.52(0.14)	0.52(**0.00**)
17	0.29	0.28(-0.01)	0.31(0.07)	0.31(**0.00**)	0.31	0.30(-0.03)	0.38(0.22)	0.37(-0.01)
18	0.11	0.10(-0.09)	0.16(0.47)	0.15(-0.06)	0.37	0.36(-0.01)	0.38(0.05)	0.39(0.01)
19	0.39	0.40(0.01)	0.41(0.06)	0.42(0.02)	0.30	0.30(**0.00**)	0.34(0.13)	0.34(**0.00**)
20	0.35	0.34(-0.02)	0.39(0.13)	0.39(**0.00**)	0.46	0.46(**0.00**)	0.50(0.08)	0.50(**0.00**)
21	0.46	0.45(-0.02)	0.52(0.13)	0.52(**0.00**)	0.42	0.42(**0.00**)	0.45(0.08)	0.45(**0.00**)
22	0.21	0.20(-0.04)	0.27(0.28)	0.26(-0.02)	0.27	0.27(**0.00**)	0.31(0.15)	0.31(**0.00**)
23	0.23	0.23(**0.00**)	0.27(0.17)	0.27(**0.00**)	0.29	0.29(**0.00**)	0.33(0.14)	0.33(**0.00**)
24	0.20	0.20(**0.00**)	0.22(0.13)	0.23(0.02)	0.39	0.38(-0.01)	0.44(0.13)	0.43(-0.01)
25	0.38	0.39(0.03)	0.38(**0.00**)	0.39(0.03)	0.26	0.26(**0.00**)	0.27(0.05)	0.28(0.02)
Mean	0.31	0.31(0.00)	0.35(0.12)	0.35(0.00)	0.35	0.35(0.00)	0.38(0.09)	0.38(0.00)

[a] In parentheses is the gain in prediction accuracy with SG-UR over SG-SR; [b] In parentheses is the gain in prediction accuracy with UG-SR over SG-SR;

[c] In parentheses is the gain in prediction accuracy with UG-UR over UG-SR; Bold in parentheses indicates the number is not significant at α = 0.05.

Table S38 Accuracy of AP prediction for environment E4 with four ME GWP models in CV2

	LL				LW			
PopId	SG-SR	SG-UR[a]	UG-SR[b]	UG-UR[c]	SG-SR	SG-UR[a]	UG-SR[b]	UG-UR[c]
1	0.30	0.31(0.02)	0.35(0.16)	0.36(0.01)	0.34	0.34(**0.00**)	0.37(0.10)	0.37(**0.00**)
2	0.25	0.26(0.02)	0.28(0.11)	0.28(**0.00**)	0.44	0.44(**0.00**)	0.47(0.08)	0.47(**0.00**)
3	0.26	0.26(**0.00**)	0.29(0.14)	0.30(0.01)	0.40	0.41(**0.00**)	0.44(0.09)	0.44(**0.00**)
4	0.45	0.45(**0.00**)	0.50(0.11)	0.50(**0.00**)	0.31	0.31(**0.00**)	0.34(0.11)	0.34(**0.00**)
5	0.39	0.40(0.01)	0.42(0.06)	0.42(**0.00**)	0.35	0.35(**0.00**)	0.41(0.17)	0.40(-0.01)
6	0.46	0.46(**0.00**)	0.52(0.13)	0.52(**0.00**)	0.36	0.35(**-0.01**)	0.37(0.05)	0.37(**0.00**)
7	0.36	0.37(0.02)	0.38(0.05)	0.39(0.03)	0.53	0.54(0.01)	0.57(0.07)	0.57(**0.00**)
8	0.35	0.35(**0.00**)	0.41(0.15)	0.40(-0.01)	0.40	0.40(**0.00**)	0.43(0.08)	0.44(**0.01**)
9	0.34	0.35(0.02)	0.36(0.05)	0.37(0.03)	0.42	0.42(**0.00**)	0.47(0.11)	0.46(**-0.01**)
10	0.46	0.45(-0.02)	0.50(0.09)	0.51(0.02)	0.55	0.55(**0.00**)	0.58(0.06)	0.58(**0.00**)
11	0.27	0.27(**0.00**)	0.31(0.15)	0.32(0.01)	0.37	0.37(**0.00**)	0.38(0.03)	0.38(**0.00**)
12	0.34	0.32(-0.05)	0.42(0.25)	0.41(-0.02)	0.58	0.58(**0.00**)	0.60(0.04)	0.60(**0.00**)
13	0.16	0.16(**0.00**)	0.22(0.37)	0.21(-0.03)	0.32	0.31(-0.04)	0.39(0.20)	0.38(-0.01)
14	0.35	0.36(0.02)	0.38(0.09)	0.38(**0.00**)	0.35	0.35(**0.00**)	0.38(0.10)	0.38(**0.00**)
15	0.25	0.25(**0.00**)	0.29(0.19)	0.30(0.02)	0.61	0.60(**-0.01**)	0.63(0.05)	0.63(**0.00**)
16	0.25	0.25(**0.00**)	0.30(0.21)	0.30(**0.00**)	0.58	0.57(**-0.01**)	0.60(0.04)	0.60(**0.00**)
17	0.25	0.25(**0.00**)	0.27(0.09)	0.27(**0.00**)	0.55	0.56(0.01)	0.57(0.03)	0.57(**0.00**)
18	0.16	0.16(**0.00**)	0.18(0.12)	0.18(**0.00**)	0.41	0.40(**-0.01**)	0.41(0.02)	0.42(0.01)
19	0.21	0.21(**0.00**)	0.24(0.18)	0.25(0.01)	0.38	0.38(**0.00**)	0.40(0.08)	0.41(0.01)
20	0.42	0.42(**0.00**)	0.46(0.08)	0.46(**0.00**)	0.52	0.52(**0.00**)	0.56(0.07)	0.56(**0.00**)
21	0.52	0.53(0.01)	0.54(0.03)	0.55(0.01)	0.41	0.39(-0.03)	0.46(0.14)	0.45(-0.02)
22	0.29	0.30(0.04)	0.30(0.05)	0.31(0.03)	0.42	0.43(0.02)	0.45(0.06)	0.45(**0.00**)
23	0.14	0.15(0.04)	0.17(0.19)	0.17(**0.00**)	0.32	0.32(**0.00**)	0.35(0.08)	0.35(**0.00**)
24	0.28	0.29(0.04)	0.28(**0.00**)	0.29(0.04)	0.50	0.50(**0.00**)	0.52(0.05)	0.52(**0.00**)
25	0.28	0.27(-0.04)	0.34(0.21)	0.33(-0.01)	0.42	0.43(0.02)	0.43(0.02)	0.44(0.02)
Mean	0.31	0.31(0.00)	0.35(0.12)	0.35(0.00)	0.43	0.43(0.00)	0.46(0.07)	0.46(0.00)

[a] In parentheses is the gain in prediction accuracy with SG-UR over SG-SR; [b] In parentheses is the gain in prediction accuracy with UG-SR over SG-SR;

[c] In parentheses is the gain in prediction accuracy with UG-UR over UG-SR; Bold in parentheses indicates the number is not significant at α = 0.05.
