# Supplementary material for: Accuracy of Across-Environment Genome-Wide Prediction in Maize Nested Association Mapping Populations
Source: G3 (Bethesda). 2013 Feb 1;3(2):263–72. doi: 10.1534/g3.112.005066 (PMC3564986; doi:10.1534/g3.112.005066)
Supplement: Supporting Information [file supp_3.2.263_FileS1.pdf]

File S1

Method for estimation of variance components and broad sense heritability (H^2)

In this study, variance components and the broad sense heritability on the mean basis were analyzed for each NAM population and trait using phenotypic data from two locations (AU and CU) and two years (2006 and 2007) based on the following mixed model

$$y_{ijk} = \mu + G_i + L_j + Y_k + L_j \times Y_k + G_i \times L_j + G_i \times Y_k + e_{ijk},$$

where y_{ijk} is the phenotypic value of line i at location j and year k, μ is the overall mean of the phenotype, G_i is the genotypic effect of the line i (i = 1, 2, …, N), L_j (j = 1, 2) is the location effect, Y_k (k = 1, 2) is the year effect, $L_j \times Y_k$ is the interaction effect between location j and year k, $G_i \times L_j$ is the interaction effect between genotype i and location j, $G_i \times Y_k$ is the interaction effect between genotype i and year k, and e_{ijk} is the residual error following a normal distribution N(0, V_E). It was assumed that G_i, L_j and Y_k were random effects, following N(0, V_G), N(0, V_L), and N(0, V_Y). Based on these assumptions, the interaction terms $G_i \times L_j$ and $G_i \times Y_k$ were also random effects, following N(0, $V_{G\times L}$) and N(0, $V_{G\times Y}$), respectively. The variance components V_G, V_L, V_Y, $V_{G\times L}$, $V_{G\times Y}$, and V_E were estimated using SAS PROC mixed. Given these variance components, the broad sense heritability H^2 on entry mean basis was estimated as $H^2 = V_G / (V_G + V_{G\times L} / 2 + V_{G\times Y} / 2 + V_E / 4)$.
