# Supplementary material for: Accuracy of Across-Environment Genome-Wide Prediction in Maize Nested Association Mapping Populations
Source: G3 (Bethesda). 2013 Feb 1;3(2):263–72. doi: 10.1534/g3.112.005066 (PMC3564986; doi:10.1534/g3.112.005066)
Supplement: Supporting Information [file supp_3.2.263_TableS1.pdf]

Table S1 Variance components for traits LL and LW based on 25 NAM populations

	LL							LW						
PopId	V_G	V_E	V_L	V_Y	V_{GL}	V_{GY}	H^2	V_G	V_E	V_L	V_Y	V_{GL}	V_{GY}	H^2
1	3712.69	3415.65	**24.84**	**5.32**	**0.00**	570.36	0.77	49.15	85.97	**12.87**	**0.00**	**0.00**	**0.12**	0.70
2	2828.92	3184.92	**229.33**	**0.00**	**0.00**	**0.00**	0.78	86.33	84.06	**0.95**	**1.57**	**0.00**	**0.58**	0.80
3	1899.01	3922.08	**0.00**	**4.60**	**186.65**	**363.68**	0.60	86.55	90.13	**0.20**	**3.44**	**0.00**	**3.10**	0.78
4	3117.24	2906.74	**82.00**	**132.28**	**101.53**	**0.00**	0.80	42.18	58.18	**5.95**	**0.00**	**0.00**	**0.00**	0.74
5	2640.95	2618.83	**606.87**	**20.66**	**266.01**	**0.00**	0.77	53.61	57.78	**2.28**	**0.00**	**1.00**	**0.00**	0.78
6	2394.58	3030.18	**17.28**	**91.56**	**185.98**	**0.00**	0.74	30.49	89.14	**1.92**	**0.00**	**0.00**	**0.00**	0.58
7	2693.34	2201.03	**90.44**	**401.90**	**53.80**	**0.00**	0.82	80.22	72.46	**0.80**	**16.53**	**0.00**	**0.00**	0.82
8	2643.83	2778.52	**8.20**	**317.28**	**0.00**	**336.46**	0.75	41.57	64.57	**0.89**	**4.29**	**0.00**	**0.00**	0.72
9	2202.03	3048.93	**36.66**	**394.20**	**10.21**	**0.00**	0.74	55.58	78.83	**5.55**	**1.37**	**0.00**	**0.00**	0.74
10	2841.07	2128.14	**28.96**	**136.27**	**0.00**	**63.59**	0.83	64.56	50.93	**0.69**	**4.66**	**1.58**	**7.40**	0.79
11	2923.63	3782.49	**0.00**	**223.94**	**357.97**	**0.00**	0.72	38.51	89.52	**12.38**	**4.27**	**0.00**	**0.00**	0.63
12	3043.85	2414.63	**195.07**	**198.70**	414.83	**0.00**	0.79	54.25	71.07	**0.48**	**0.33**	**2.64**	**0.00**	0.74
13	3096.70	2391.68	**380.26**	**38.62**	**0.00**	**0.00**	0.84	60.14	68.16	**17.25**	**0.00**	**0.00**	**0.00**	0.78
14	2432.85	2367.77	**10.74**	**457.33**	**0.00**	**165.20**	0.78	49.85	53.55	**0.25**	**0.03**	**0.00**	**0.00**	0.79
15	2872.87	2381.87	**0.00**	**43.40**	**259.27**	**215.36**	0.78	62.33	85.35	**0.84**	**0.25**	**0.00**	6.75	0.72
16	1949.81	3066.43	**0.00**	**686.50**	**0.00**	**0.00**	0.72	60.48	78.03	**0.99**	**12.48**	**0.00**	**2.92**	0.74
17	1605.53	3316.92	**1388.21**	**247.90**	**220.81**	**0.00**	0.63	71.48	53.51	**0.00**	**14.17**	**0.00**	5.12	0.82
18	1195.92	2718.24	**86.83**	**104.09**	**81.95**	**0.00**	0.62	26.61	68.20	**0.33**	**5.75**	**0.00**	**1.53**	0.60
19	2329.66	3426.67	**121.82**	**0.00**	**194.02**	521.18	0.66	55.51	55.54	**1.95**	**0.00**	**1.42**	**0.00**	0.79
20	2076.13	1945.32	**364.59**	**95.58**	**50.04**	513.25	0.73	59.99	59.68	**0.00**	**0.97**	**0.00**	**0.00**	0.80
21	3077.63	2317.01	**0.00**	**0.00**	**32.56**	**0.00**	0.84	44.40	64.85	**0.98**	**6.78**	11.19	**0.00**	0.67
22	1987.63	2808.07	**235.30**	**1353.64**	**152.09**	468.61	0.66	66.60	94.38	**0.04**	**29.04**	**6.41**	**0.00**	0.71
23	2171.32	3692.89	**76.03**	**11.10**	**0.00**	**0.00**	0.70	49.86	70.49	**0.36**	**18.46**	**1.59**	**0.00**	0.73
24	2489.81	3212.06	**0.00**	950.74	**0.00**	**0.00**	0.76	58.50	53.30	**0.16**	**2.97**	**3.58**	**0.00**	0.79
25	2212.55	2730.97	301.91	641.40	352.19	**137.16**	0.70	58.45	61.95	**0.14**	6.18	**0.00**	**0.00**	0.79
Mean	2497.58	2872.32	171.41	262.28	116.80	134.19	0.74	56.29	70.39	2.73	5.34	1.18	1.10	0.74

Bold in the table indicates the variance component is not significant at α = 0.05.
