# Supplementary material for: Accuracy of Across-Environment Genome-Wide Prediction in Maize Nested Association Mapping Populations
Source: G3 (Bethesda). 2013 Feb 1;3(2):263–72. doi: 10.1534/g3.112.005066 (PMC3564986; doi:10.1534/g3.112.005066)
Supplement: Supporting Information [file supp_3.2.263_TableS2.pdf]

Table S2 Estimates of empirical LOD thresholds of CIM based on permutation tests

	LL		LW	
Approach	SE	ME	SE	ME
WP	2.85(0.08)	5.25(0.09)	2.90(0.07)	5.30(0.08)
AP	3.10	6.15	3.13	6.22

For WP-SE, the average of LOD thresholds across 25 NAM populations is illustrated and used for QTL identification with CIM, and in the parenthesis is standard deviation (SD) for LOD threshold estimates across 25 populations
