# Supplementary material for: Accuracy of Across-Environment Genome-Wide Prediction in Maize Nested Association Mapping Populations
Source: G3 (Bethesda). 2013 Feb 1;3(2):263–72. doi: 10.1534/g3.112.005066 (PMC3564986; doi:10.1534/g3.112.005066)
Supplement: Supporting Information [file supp_3.2.263_TableS3.pdf]

Table S3 Estimates of genetic and residual covariance and correlation (in the lower diagonal) based on individual NAM populations 1 – 10

		LL								LW							
		Genetic covariance				Residual covariance				Genetic covariance				Residual covariance			
PopId	Envi	E1	E2	E3	E4	E1	E2	E3	E4	E1	E2	E3	E4	E1	E2	E3	E4
1	E1	1038.6	1107.1	958.5	959.7	6397.5	700.9	1076.7	1196.0	22.3	15.2	13.7	24.5	155.0	17.7	22.6	18.0
	E2	0.78	1927.2	1460.7	1500.4	0.21	1793.2	593.7	638.0	0.70	21.2	14.2	22.6	0.18	60.3	8.9	12.6
	E3	0.78	0.88	1446.4	1385.8	0.20	0.21	4380.9	1013.4	0.76	0.81	14.4	16.3	0.18	0.12	96.6	14.8
	E4	0.76	0.88	0.93	1526.6	0.21	0.21	0.21	5184.2	0.99	0.94	0.82	27.2	0.19	0.21	0.20	58.4
2	E1	545.9	514.1	489.3	451.8	6560.8	1651.7	2176.5	1953.7	40.9	29.0	29.8	27.1	193.0	34.3	26.4	38.0
	E2	0.66	1125.4	766.9	837.0	0.41	2483.8	1345.4	1217.4	0.93	24.0	23.7	21.7	0.28	79.5	17.0	30.3
	E3	0.57	0.62	1362.7	766.3	0.40	0.41	4424.8	1584.9	0.90	0.93	26.8	22.7	0.22	0.22	76.3	25.4
	E4	0.60	0.77	0.64	1043.2	0.41	0.42	0.41	3429.8	0.86	0.90	0.89	24.2	0.33	0.41	0.35	69.3
3	E1	767.2	352.2	426.1	160.5	6004.0	1815.0	2026.1	1729.6	72.3	43.2	25.5	35.5	127.2	19.7	39.2	24.3
	E2	0.56	523.3	216.3	135.9	0.35	4491.1	1735.4	1504.6	0.88	33.7	22.0	28.9	0.23	57.6	14.7	19.7
	E3	0.59	0.36	684.8	376.3	0.35	0.34	5663.1	1670.5	0.74	0.93	16.6	19.4	0.31	0.17	127.9	31.5
	E4	0.26	0.26	0.63	515.1	0.35	0.35	0.35	4082.0	0.84	0.99	0.95	25.4	0.22	0.26	0.28	96.5
4	E1	1595.5	1091.4	1201.5	1301.4	3903.4	889.7	1143.3	1160.5	21.0	15.6	8.9	18.9	85.1	10.8	16.9	11.2
	E2	0.74	1363.8	1182.9	1348.0	0.33	1828.2	808.4	807.9	0.89	14.7	8.8	17.2	0.22	29.4	11.9	10.7
	E3	0.72	0.77	1746.1	1369.4	0.33	0.34	3141.9	988.2	0.84	0.99	5.3	10.7	0.21	0.25	77.8	15.4
	E4	0.78	0.87	0.78	1751.5	0.33	0.34	0.32	3100.5	0.88	0.96	0.99	21.8	0.16	0.26	0.23	55.8
5	E1	718.2	693.8	823.1	605.4	3447.5	915.5	1391.7	1276.8	11.9	11.4	10.6	15.9	108.0	22.8	44.7	30.3
	E2	0.75	1206.1	943.4	821.1	0.37	1802.2	991.2	956.8	0.86	14.7	15.8	19.7	0.41	28.4	20.9	15.5
	E3	0.73	0.64	1790.9	877.2	0.37	0.37	4060.1	1392.7	0.65	0.87	22.6	21.5	0.41	0.37	110.7	29.4
	E4	0.73	0.76	0.67	963.6	0.36	0.38	0.37	3563.5	0.82	0.92	0.81	31.4	0.40	0.40	0.38	53.9
6	E1	1248.4	909.1	944.9	874.5	3144.4	1001.7	1134.8	1055.9	18.8	8.9	12.8	14.1	109.0	9.3	11.2	9.8
	E2	0.68	1454.5	764.3	956.2	0.35	2592.1	1006.6	982.0	0.64	10.4	7.0	5.8	0.10	73.4	9.3	10.0
	E3	0.87	0.65	952.3	843.9	0.35	0.34	3392.5	1092.8	0.71	0.52	17.3	13.7	0.12	0.12	81.7	11.6
	E4	0.75	0.76	0.83	1092.3	0.34	0.35	0.34	3079.2	0.93	0.51	0.94	12.2	0.12	0.15	0.16	65.0
7	E1	1281.0	882.0	1057.2	1233.8	2408.2	600.7	773.0	606.7	29.6	31.0	24.8	33.8	98.7	19.4	21.1	20.9
	E2	0.86	815.7	802.5	1051.2	0.28	1903.5	674.4	562.0	0.94	37.0	24.8	39.0	0.24	63.9	26.8	15.2
	E3	0.83	0.78	1283.1	1240.9	0.28	0.28	3085.2	697.0	0.99	0.89	21.3	30.0	0.22	0.35	94.5	9.8

	E4	0.81	0.87	0.82	1791.4	0.28	0.29	0.28	1985.8	0.95	0.98	0.99	42.8	0.28	0.25	0.13	58.5
8	E1	1103.5	869.4	696.9	834.2	3801.7	1592.2	1779.6	1405.0	11.9	8.9	13.7	9.5	112.3	30.5	18.1	24.1
	E2	0.69	1451.4	765.1	818.9	0.43	3556.5	1715.7	1318.3	0.80	10.3	10.3	8.5	0.31	86.0	12.9	22.0
	E3	0.57	0.55	1359.0	862.1	0.43	0.42	4620.6	1545.3	0.88	0.71	20.4	11.4	0.23	0.19	56.3	13.1
	E4	0.79	0.68	0.74	1011.5	0.44	0.42	0.43	2749.9	0.82	0.85	0.75	11.3	0.31	0.32	0.24	54.6
9	E1	747.8	479.2	686.4	603.7	2915.3	1007.4	1334.3	1373.2	5.3	9.6	9.3	10.4	161.2	17.4	20.2	19.7
	E2	0.62	802.0	567.7	578.8	0.40	2229.4	1141.7	1228.9	0.72	34.7	22.9	19.3	0.18	56.2	15.8	9.4
	E3	0.75	0.60	1134.5	869.7	0.39	0.38	4005.1	1608.7	0.80	0.77	25.7	12.7	0.18	0.24	76.1	12.6
	E4	0.64	0.59	0.75	1195.1	0.39	0.40	0.39	4303.9	0.62	0.86	0.34	52.9	0.19	0.16	0.18	64.8
10	E1	1184.2	1040.6	975.7	787.3	2403.3	1083.1	1457.7	1187.1	20.8	15.9	23.1	20.4	95.7	34.7	22.8	15.9
	E2	0.82	1377.4	1035.8	938.8	0.48	2132.2	1377.2	1075.3	0.66	27.9	32.2	15.5	0.40	78.7	14.8	19.1
	E3	0.78	0.77	1323.6	930.3	0.47	0.47	3963.7	1481.9	0.74	0.90	46.6	30.2	0.29	0.21	64.8	15.6
	E4	0.66	0.73	0.74	1192.8	0.48	0.46	0.47	2549.0	0.89	0.49	0.75	35.1	0.25	0.33	0.30	43.2

Envi: environment
