# Supplementary material for: Accuracy of Across-Environment Genome-Wide Prediction in Maize Nested Association Mapping Populations
Source: G3 (Bethesda). 2013 Feb 1;3(2):263–72. doi: 10.1534/g3.112.005066 (PMC3564986; doi:10.1534/g3.112.005066)
Supplement: Supporting Information [file supp_3.2.263_TableS5.pdf]

Table S5 Estimates of genetic and residual covariances and correlations (in the lower diagonal) based on individual NAM populations 21-25

		LL								LW							
		Genetic covariance				Residual covariance				Genetic covariance				Residual covariance			
PopId	Envi	E1	E2	E3	E4	E1	E2	E3	E4	E1	E2	E3	E4	E1	E2	E3	E4
21	E1	1153.0	1104.6	913.2	905.8	2485.8	1047.1	1313.0	965.1	32.4	16.3	18.8	17.0	69.6	4.7	15.0	11.0
	E2	0.81	1603.0	1203.6	1269.5	0.44	2329.9	1258.4	943.2	0.68	17.6	9.5	9.2	0.07	69.84	7.07	12.5
	E3	0.79	0.88	1167.9	1097.4	0.43	0.43	3690.1	1186.7	0.65	0.45	25.8	13.9	0.18	0.09	98.2	6.8
	E4	0.72	0.85	0.86	1391.6	0.41	0.42	0.42	2212.9	0.82	0.60	0.75	13.4	0.16	0.19	0.09	64.7
22	E1	1000.1	1000.2	477.3	375.1	4400.6	1041.3	1010.4	996.9	30.4	25.9	18.9	20.9	243.8	17.8	28.6	15.5
	E2	0.71	1972.3	625.6	694.1	0.29	2934.1	831.3	839.4	0.84	31.5	16.6	25.0	0.15	56.6	15.2	13.3
	E3	0.57	0.53	707.1	552.6	0.29	0.29	2788.5	808.0	0.95	0.82	13.0	14.4	0.19	0.21	92.2	11.8
	E4	0.42	0.56	0.74	786.1	0.29	0.29	0.29	2780.3	0.83	0.98	0.87	21.0	0.14	0.24	0.17	52.1
23	E1	1329.2	842.1	765.1	831.6	3094.9	776.0	778.0	872.5	17.0	13.4	18.3	19.9	102.6	27.2	21.4	20.8
	E2	0.70	1097.0	633.3	703.5	0.24	3333.9	800.1	905.6	0.69	22.3	14.0	18.0	0.27	96.7	21.1	19.7
	E3	0.85	0.78	603.7	622.1	0.24	0.24	3317.6	905.5	0.98	0.65	20.5	14.3	0.27	0.27	62.0	15.0
	E4	0.72	0.67	0.80	995.9	0.24	0.24	0.24	4239.4	0.99	0.78	0.65	23.5	0.27	0.27	0.25	56.6
24	E1	1495.4	541.3	724.0	537.2	3241.0	1437.8	1738.8	1528.7	39.5	22.4	24.7	27.0	72.2	12.9	19.4	19.1
	E2	0.78	318.5	427.3	306.8	0.43	3479.4	1815.5	1611.7	0.78	20.8	18.2	17.6	0.19	66.6	18.2	18.5
	E3	0.53	0.68	1242.5	610.6	0.42	0.43	5179.5	1917.9	0.93	0.94	18.1	17.1	0.25	0.24	83.8	13.8
	E4	0.54	0.67	0.67	664.0	0.42	0.43	0.42	4031.1	0.95	0.85	0.88	20.6	0.31	0.31	0.21	52.6
25	E1	1607.2	918.9	1127.7	697.3	2857.2	1072.6	1288.7	1393.5	29.9	18.1	16.0	20.1	85.2	28.3	26.8	22.6
	E2	0.73	978.6	727.8	766.6	0.41	2432.8	1174.9	1302.6	0.59	31.8	9.3	30.3	0.44	47.6	26.7	26.9
	E3	0.77	0.63	1348.0	666.3	0.40	0.39	3725.9	1592.9	0.85	0.48	12.0	11.8	0.28	0.37	110.2	29.9
	E4	0.59	0.82	0.61	883.6	0.40	0.40	0.40	4337.6	0.61	0.89	0.56	36.5	0.32	0.51	0.37	59.4

Envi: environment
