# Supplementary material for: Accuracy of Across-Environment Genome-Wide Prediction in Maize Nested Association Mapping Populations
Source: G3 (Bethesda). 2013 Feb 1;3(2):263–72. doi: 10.1534/g3.112.005066 (PMC3564986; doi:10.1534/g3.112.005066)
Supplement: Supporting Information [file supp_3.2.263_TableS6.pdf]

Table S6 Estimates of genetic and residual covariance and correlation (in the lower diagonal) based on a full data set of 25 NAM populations.

	LL								LW							
	Genetic covariance				Residual covariance				Genetic covariance				Residual covariance			
Envi	E1	E2	E3	E4	E1	E2	E3	E4	E1	E2	E3	E4	E1	E2	E3	E4
E1	401.98	357.82	364.59	345.25	5097.97	2021.13	2029.87	1977.42	12.94	9.743	9.70	10.97	137.20	38.03	35.99	42.44
E2	0.83	460.65	351.99	393.84	0.45	3882.87	1719.01	1992.78	0.90	8.97	7.72	9.44	0.36	81.45	81.45	30.95
E3	0.88	0.79	427.45	373.01	0.40	0.38	5261.99	2016.00	0.91	0.87	8.74	8.92	0.30	0.34	104.49	35.47
E4	0.82	0.87	0.86	440.38	0.40	0.46	0.40	4872.04	0.92	0.95	0.91	11.09	0.39	0.44	0.38	85.04

Envi: environment
