# Supplementary material for: Accuracy of Across-Environment Genome-Wide Prediction in Maize Nested Association Mapping Populations
Source: G3 (Bethesda). 2013 Feb 1;3(2):263–72. doi: 10.1534/g3.112.005066 (PMC3564986; doi:10.1534/g3.112.005066)
Supplement: Supporting Information [file supp_3.2.263_TableS7.pdf]

Table S7 Accuracy of WP prediction for environment E1 with QP and GWP in CV1

	LL				LW			
	SE		ME		SE		ME	
PopId	QP[a]	GWP[b]	QP[c]	GWP[d]	QP[a]	GWP[b]	QP[c]	GWP[d]
1	0.25(1.5)	0.33(0.32)	0.34(3.3, 0.36)	0.42(0.24, 0.27)	0.06(1.4)	0.29(3.83)	0.11(2.1, 0.83)	0.39(2.55, 0.34)
2	0.02(1.1)	0.17(7.50)	0.01(1.6, **-0.50**)	0.24(23.00, 0.41)	0.25(2.0)	0.40(0.60)	0.18(1.8, -0.28)	0.45(1.50, 0.12)
3	0.09(1.2)	0.28(2.11)	0.08(1.7, **-0.11**)	0.28(2.50, **0.00**)	0.39(3.2)	0.55(0.41)	0.36(3.0, -0.08)	0.59(0.64, 0.07)
4	0.24(2.8)	0.48(1.00)	0.22(3.3, **-0.08**)	0.50(1.27, 0.04)	0.25(2.6)	0.49(0.96)	0.21(2.4, -0.16)	0.53(1.52, 0.08)
5	0.16(2.0)	0.32(1.00)	0.22(3.0, 0.38)	0.39(0.77, 0.22)	0.05(1.4)	0.20(3.00)	0.03(1.6, **-0.40**)	0.29(8.67, 0.45)
6	0.26(2.4)	0.50(0.92)	0.22(2.8, -0.15)	0.53(1.41, 0.06)	0.03(1.4)	0.32(9.67)	0.06(1.6, 1.00)	0.38(5.33, 0.19)
7	0.33(2.7)	0.52(0.58)	0.29(2.4, -0.12)	0.57(0.97, 0.10)	0.26(2.8)	0.48(0.85)	0.22(2.5, -0.15)	0.52(1.36, 0.08)
8	0.07(1.6)	0.31(3.43)	0.08(2.0, **0.14**)	0.35(3.37, 0.13)	0.06(1.4)	0.31(4.17)	0.16(1.9, 1.67)	0.38(1.38, 0.23)
9	0.31(2.3)	0.34(0.10)	0.25(2.5, -0.19)	0.38(0.52, 0.12)	0.02(1.1)	0.17(7.50)	0.13(2.1, 5.50)	0.36(1.77, 1.12)
10	0.26(2.4)	0.47(0.81)	0.22(2.8, -0.15)	0.49(1.23, 0.04)	0.20(2.5)	0.39(0.95)	0.17(3.1, **-0.15**)	0.52(2.06, 0.33)
11	0.26(2.4)	0.37(0.42)	0.27(3.0, **0.04**)	0.45(0.67, 0.22)	0.13(2.5)	0.39(2.00)	0.15(3.1, **0.15**)	0.42(1.80, 0.08)
12	0.24(3.0)	0.49(1.04)	0.20(2.6, -0.17)	0.54(1.70, 0.10)	0.29(3.1)	0.54(0.86)	0.31(3.3, 0.07)	0.57(0.84, 0.06)
13	0.20(2.7)	0.45(1.25)	0.16(2.9, -0.20)	0.47(1.94, 0.04)	0.34(3.0)	0.50(0.47)	0.28(3.5, -0.18)	0.52(0.86, 0.04)
14	0.13(2.0)	0.38(1.92)	0.14(2.3, **0.08**)	0.42(2.00, 0.11)	0.20(2.4)	0.47(1.35)	0.14(1.9, -0.30)	0.50(2.57, 0.06)
15	0.09(1.6)	0.28(2.11)	0.07(1.9, **-0.22**)	0.31(3.43, 0.11)	0.13(1.8)	0.30(1.31)	0.12(2.8, **-0.08**)	0.34(1.83, 0.13)
16	0.22(1.9)	0.33(0.50)	0.18(2.0, -0.18)	0.36(1.00, 0.09)	0.24(2.4)	0.46(0.92)	0.31(3.2, 0.29)	0.54(0.74, 0.17)
17	0.03(1.2)	0.27(8.00)	0.04(1.2, **0.33**)	0.29(6.25, 0.07)	0.33(3.0)	0.55(0.67)	0.33(3.7, **0.00**)	0.59(0.79, 0.07)
18	0.08(1.2)	0.28(2.50)	0.03(1.2, -0.62)	0.28(8.33, **0.00**)	0.19(2.1)	0.39(1.05)	0.23(2.5, 0.21)	0.43(0.87, 0.10)
19	0.29(2.4)	0.37(0.28)	0.20(2.1, -0.31)	0.40(1.00, 0.08)	0.29(2.2)	0.41(0.41)	0.28(2.7, **-0.03**)	0.44(0.57, 0.07)
20	0.15(2.6)	0.47(2.13)	0.09(2.4, -0.40)	0.48(4.33, 0.02)	0.21(2.2)	0.47(1.24)	0.29(2.7, 0.38)	0.51(0.76, 0.09)
21	0.35(3.1)	0.49(0.40)	0.32(3.4, **-0.09**)	0.51(0.59, 0.04)	0.19(2.2)	0.47(1.47)	0.11(2.1, -0.42)	0.48(3.36, 0.02)
22	0.16(1.5)	0.35(1.19)	0.19(2.4, **0.19**)	0.41(1.16, 0.17)	0.03(1.2)	0.32(9.67)	0.12(2.0, 3.00)	0.42(2.50, 0.31)
23	0.31(3.1)	0.42(0.35)	0.24(2.6, -0.23)	0.46(0.92, 0.10)	0.06(1.8)	0.29(3.83)	0.17(2.9, 1.83)	0.43(1.53, 0.48)
24	0.18(2.7)	0.45(1.50)	0.11(1.8, -0.39)	0.44(3.00, -0.02)	0.33(3.1)	0.50(0.52)	0.36(3.0, 0.09)	0.53(0.47, 0.06)
25	0.20(2.8)	0.50(1.50)	0.20(2.6, **0.00**)	0.51(1.55, 0.02)	0.26(2.9)	0.45(0.73)	0.25(2.4, **-0.04**)	0.48(0.92, 0.07)
Mean	0.20(2.2)	0.38(0.93)	0.17(2.4, -0.10)	0.42(1.40, 0.10)	0.19(2.2)	0.40(1.11)	0.20(2.6, 0.06)	0.46(1.29, 0.15)

[a] In parentheses is the number of QTL identified by QP based on the SE model; [b] In parentheses is the gain in prediction accuracy with GWP over QP based on the SE model; [c] The first value in parentheses is the number of QTL identified by QP based on the ME model; and the second one the gain

with ME over SE for QP; [d] The first value in parentheses is the gain in accuracy with GWP over QP based on the ME model; and the second one is the gain with ME over SE using GWP. Bold in parentheses indicates the number is not significant at $\alpha = 0.05$.
