# Supplementary material for: Accuracy of Across-Environment Genome-Wide Prediction in Maize Nested Association Mapping Populations
Source: G3 (Bethesda). 2013 Feb 1;3(2):263–72. doi: 10.1534/g3.112.005066 (PMC3564986; doi:10.1534/g3.112.005066)
Supplement: Supporting Information [file supp_3.2.263_TableS8.pdf]

Table S8 Accuracy of WP prediction for environment E2 with QP and GWP in CV1

	LL				LW			
	SE		ME		SE		ME	
PopId	QP[a]	GWP[b]	QP[c]	GWP[d]	QP[a]	GWP[b]	QP[c]	GWP[d]
1	0.50(4.0)	0.67(0.34)	0.48(3.3, **-0.04**)	0.67(0.40, **0.00**)	0.17(2.5)	0.46(1.71)	0.16(2.1, **-0.06**)	0.50(2.12, 0.09)
2	0.11(2.2)	0.40(2.64)	0.05(1.6, -0.55)	0.40(7.00, **0.00**)	0.21(2.5)	0.49(1.33)	0.17(1.8, -0.19)	0.53(2.12, 0.08)
3	0.21(1.8)	0.17(-0.19)	0.09(1.7, -0.57)	0.18(1.00, 0.06)	0.35(3.3)	0.59(0.69)	0.35(3.0, **0.00**)	0.61(0.74, 0.03)
4	0.20(2.9)	0.56(1.80)	0.25(3.3, 0.25)	0.57(1.28, 0.02)	0.41(3.0)	0.59(0.44)	0.28(2.4, -0.32)	0.62(1.21, 0.05)
5	0.27(3.0)	0.50(0.85)	0.25(3.0, **-0.07**)	0.51(1.04, 0.02)	0.07(2.4)	0.45(5.43)	0.05(1.6, **-0.29**)	0.46(8.20, 0.02)
6	0.22(2.6)	0.49(1.23)	0.17(2.8, -0.23)	0.50(1.94, 0.02)	0.07(1.2)	0.33(3.71)	0.09(1.6, **0.29**)	0.34(2.78, 0.03)
7	0.25(2.0)	0.42(0.68)	0.25(2.4, **0.00**)	0.51(1.04, 0.21)	0.35(3.0)	0.58(0.66)	0.27(2.5, -0.23)	0.62(1.30, 0.07)
8	0.10(1.9)	0.35(2.50)	0.11(2.0, **0.10**)	0.37(2.36, 0.06)	0.21(1.6)	0.35(0.67)	0.21(1.9, **0.00**)	0.42(1.00, 0.20)
9	0.07(2.0)	0.30(3.29)	0.11(2.5, 0.57)	0.32(1.91, 0.07)	0.22(2.3)	0.44(1.00)	0.25(2.1, 0.14)	0.43(0.72, -0.02)
10	0.19(2.7)	0.50(1.63)	0.18(2.8, **-0.05**)	0.51(1.83, 0.02)	0.14(1.9)	0.36(1.57)	0.18(3.1, 0.29)	0.36(1.00, **0.00**)
11	0.26(2.8)	0.52(1.00)	0.31(3.0, 0.19)	0.55(0.77, 0.06)	0.27(2.0)	0.40(0.48)	0.25(3.1, **-0.07**)	0.43(0.72, 0.07)
12	0.22(3.0)	0.56(1.55)	0.21(2.6, **-0.05**)	0.59(1.81, 0.05)	0.27(3.4)	0.58(1.15)	0.30(3.3, 0.11)	0.61(1.03, 0.05)
13	0.21(2.4)	0.40(0.90)	0.19(2.9, **-0.10**)	0.45(1.37, 0.12)	0.18(2.1)	0.39(1.17)	0.19(3.5, **0.06**)	0.47(1.47, 0.21)
14	0.32(2.9)	0.42(0.31)	0.19(2.3, -0.41)	0.42(1.21, **0.00**)	0.05(1.4)	0.27(4.40)	0.07(1.9, **0.40**)	0.36(4.14, 0.33)
15	0.25(3.0)	0.46(0.84)	0.15(1.9, -0.40)	0.46(2.07, **0.00**)	0.21(2.9)	0.55(1.62)	0.18(2.8, -0.14)	0.58(2.22, 0.05)
16	0.19(2.1)	0.31(0.63)	0.17(2.0, **-0.11**)	0.32(0.88, 0.03)	0.44(3.3)	0.56(0.27)	0.36(3.2, -0.18)	0.59(0.64, 0.05)
17	0.04(0.9)	0.13(2.25)	0.02(1.2, **-0.50**)	0.19(8.50, 0.46)	0.40(3.5)	0.61(0.52)	0.38(3.7, **-0.05**)	0.64(0.68, 0.05)
18	0.02(1.1)	0.29(13.50)	0.03(1.2, **0.50**)	0.31(9.33, 0.07)	0.18(1.9)	0.30(0.67)	0.27(2.5, 0.50)	0.43(0.59, 0.43)
19	0.08(1.5)	0.28(2.50)	0.05(2.1, -0.38)	0.30(5.00, 0.07)	0.20(2.3)	0.40(1.00)	0.25(2.7, 0.25)	0.46(0.84, 0.15)
20	0.11(2.2)	0.52(3.73)	0.10(2.4, **-0.09**)	0.54(4.40, 0.04)	0.31(3.0)	0.53(0.71)	0.31(2.7, **0.00**)	0.56(0.81, 0.06)
21	0.35(3.3)	0.58(0.66)	0.35(3.4, **0.00**)	0.61(0.74, 0.05)	0.14(1.8)	0.35(1.50)	0.08(2.1, -0.43)	0.40(4.00, 0.14)
22	0.34(2.6)	0.49(0.44)	0.22(2.4, -0.35)	0.50(1.27, 0.02)	0.32(2.9)	0.58(0.81)	0.23(2.0, -0.28)	0.60(1.61, 0.03)
23	0.16(2.4)	0.43(1.69)	0.16(2.6, **0.00**)	0.46(1.88, 0.07)	0.24(2.0)	0.36(0.50)	0.21(2.9, **-0.12**)	0.41(0.95, 0.14)
24	0.01(1.2)	0.16(15.00)	0.06(1.8, 5.00)	0.25(3.17, 0.56)	0.25(2.2)	0.42(0.68)	0.32(3.0, 0.28)	0.48(0.50, 0.14)
25	0.10(2.2)	0.38(2.80)	0.05(2.6, -0.50)	0.42(7.40, 0.11)	0.29(2.7)	0.47(0.62)	0.27(2.4, **-0.07**)	0.45(0.67, -0.04)
Mean	0.19(2.4)	0.41(1.15)	0.17(2.4, -0.12)	0.44(1.60, 0.06)	0.24(2.4)	0.46(0.92)	0.23(2.6, -0.05)	0.49(1.18, 0.08)

[a] In parentheses is the number of QTL identified by QP based on the SE model; [b] In parentheses is the gain in prediction accuracy with GWP over QP based on the SE model; [c] The first value in parentheses is the number of QTL identified by QP based on the ME model; and the second one the gain

with ME over SE for QP; [d] The first value in parentheses is the gain in accuracy with GWP over QP based on the ME model; and the second one is the gain with ME over SE using GWP. Bold in parentheses indicates the number is not significant at $\alpha = 0.05$.
