# Supplementary material for: Accuracy of Across-Environment Genome-Wide Prediction in Maize Nested Association Mapping Populations
Source: G3 (Bethesda). 2013 Feb 1;3(2):263–72. doi: 10.1534/g3.112.005066 (PMC3564986; doi:10.1534/g3.112.005066)
Supplement: Supporting Information [file supp_3.2.263_TableS9.pdf]

**Table S9 Accuracy of WP prediction for environment E3 with QP and GWP in CV1**

| | LL | | | | LW | | | |
|---|---|---|---|---|---|---|---|---|
| | SE | | ME | | SE | | ME | |
| PopId | QP[a] | GWP[b] | QP[c] | GWP[d] | QP[a] | GWP[b] | QP[c] | GWP[d] |
| 1 | 0.32(2.8) | 0.51(0.59) | 0.34(3.3, **0.06**) | 0.55(0.62, 0.08) | 0.12(1.5) | 0.36(2.00) | 0.13(2.1, 0.08) | 0.41(2.15, 0.14) |
| 2 | 0.09(1.7) | 0.35(2.89) | 0.04(1.6, -0.56) | 0.36(8.00, 0.03) | 0.23(2.8) | 0.52(1.26) | 0.16(1.8, -0.30) | 0.55(2.44, 0.06) |
| 3 | 0.07(1.3) | 0.24(2.43) | 0.06(1.7, **-0.14**) | 0.26(3.33, 0.08) | 0.27(2.1) | 0.27(0.00) | 0.27(3.0, **0.00**) | 0.37(0.37, 0.37) |
| 4 | 0.30(2.9) | 0.50(0.67) | 0.28(3.3, **-0.07**) | 0.54(0.93, 0.08) | 0.01(1.3) | 0.18(17.00) | 0.05(2.4, 4.00) | 0.29(4.80, 0.61) |
| 5 | 0.23(2.7) | 0.44(0.91) | 0.21(3.0, **-0.09**) | 0.46(1.19, 0.05) | 0.04(1.4) | 0.30(6.50) | 0.04(1.6, 0.00) | 0.39(8.75, 0.30) |
| 6 | 0.36(2.5) | 0.47(0.31) | 0.24(2.8, -0.33) | 0.54(1.25, 0.15) | 0.09(1.8) | 0.31(2.44) | 0.06(1.6, -0.33) | 0.35(4.83, 0.13) |
| 7 | 0.26(2.4) | 0.49(0.88) | 0.25(2.4, **-0.04**) | 0.55(1.20, 0.12) | 0.19(2.0) | 0.42(1.21) | 0.21(2.5, **0.11**) | 0.50(1.38, 0.19) |
| 8 | 0.06(1.7) | 0.32(4.33) | 0.07(2.0, **0.17**) | 0.35(4.00, 0.09) | 0.14(2.2) | 0.48(2.43) | 0.15(1.9, **0.07**) | 0.49(2.27, 0.02) |
| 9 | 0.18(2.3) | 0.42(1.33) | 0.22(2.5, 0.22) | 0.45(1.05, 0.07) | 0.17(2.6) | 0.45(1.65) | 0.25(2.1, 0.47) | 0.48(0.92, 0.07) |
| 10 | 0.11(2.6) | 0.42(2.82) | 0.12(2.8, **0.09**) | 0.48(3.00, 0.14) | 0.33(3.3) | 0.56(0.70) | 0.25(3.1, -0.24) | 0.56(1.24, **0.00**) |
| 11 | 0.31(2.7) | 0.54(0.74) | 0.31(3.0, **0.00**) | 0.56(0.81, 0.04) | 0.10(2.1) | 0.36(2.60) | 0.11(3.1, **0.10**) | 0.41(2.73, 0.14) |
| 12 | 0.18(2.3) | 0.43(1.39) | 0.17(2.6, **-0.06**) | 0.50(1.94, 0.16) | 0.07(1.3) | 0.28(3.00) | 0.17(3.3, 1.43) | 0.38(1.24, 0.36) |
| 13 | 0.19(2.8) | 0.41(1.16) | 0.19(2.9, **0.00**) | 0.43(1.26, 0.05) | 0.06(2.0) | 0.26(3.33) | 0.15(3.5, 1.50) | 0.35(1.33, 0.35) |
| 14 | 0.16(2.1) | 0.31(0.94) | 0.12(2.3, -0.25) | 0.35(1.92, 0.13) | 0.10(1.6) | 0.32(2.20) | 0.12(1.9, **0.20**) | 0.42(2.50, 0.31) |
| 15 | 0.05(1.4) | 0.25(4.00) | 0.08(1.9, **0.60**) | 0.30(2.75, 0.20) | 0.16(2.0) | 0.40(1.50) | 0.18(2.8, **0.12**) | 0.48(1.67, 0.20) |
| 16 | 0.10(1.9) | 0.32(2.20) | 0.07(2.0, -0.30) | 0.33(3.71, 0.03) | 0.08(1.7) | 0.36(3.50) | 0.21(3.2, 1.62) | 0.45(1.14, 0.25) |
| 17 | 0.07(1.5) | 0.32(3.57) | 0.07(1.2, **0.00**) | 0.37(4.29, 0.16) | 0.12(2.0) | 0.38(2.17) | 0.23(3.7, 0.92) | 0.45(0.96, 0.18) |
| 18 | 0.01(1.0) | 0.17(16.00) | 0.03(1.2, **2.00**) | 0.21(6.00, 0.24) | 0.33(2.4) | 0.33(0.00) | 0.30(2.5, -0.09) | 0.39(0.30, 0.18) |
| 19 | 0.25(2.8) | 0.46(0.84) | 0.20(2.1, -0.20) | 0.47(1.35, 0.02) | 0.21(2.1) | 0.44(1.10) | 0.29(2.7, 0.38) | 0.46(0.59, 0.05) |
| 20 | 0.13(1.9) | 0.34(1.62) | 0.05(2.4, -0.62) | 0.39(6.80, 0.15) | 0.15(2.1) | 0.43(1.87) | 0.24(2.7, 0.60) | 0.46(0.92, 0.07) |
| 21 | 0.24(2.6) | 0.47(0.96) | 0.33(3.4, 0.38) | 0.54(0.64, 0.15) | 0.21(2.5) | 0.38(0.81) | 0.11(2.1, -0.48) | 0.42(2.82, 0.11) |
| 22 | 0.21(1.9) | 0.38(0.81) | 0.22(2.4, **0.05**) | 0.40(0.82, 0.05) | 0.09(1.4) | 0.35(2.89) | 0.11(2.0, **0.22**) | 0.43(2.91, 0.23) |
| 23 | 0.21(1.8) | 0.32(0.52) | 0.21(2.6, **0.00**) | 0.42(1.00, 0.31) | 0.14(2.1) | 0.38(1.71) | 0.20(2.9, 0.43) | 0.42(1.10, 0.11) |
| 24 | 0.04(1.6) | 0.26(5.50) | 0.05(1.8, **0.25**) | 0.27(4.40, 0.04) | 0.34(2.2) | 0.41(0.21) | 0.38(3.0, 0.12) | 0.48(0.26, 0.17) |
| 25 | 0.11(2.0) | 0.37(2.36) | 0.15(2.6, 0.36) | 0.40(1.67, 0.08) | 0.11(1.6) | 0.29(1.64) | 0.16(2.4, 0.45) | 0.34(1.12, 0.17) |
| Mean | 0.17(2.1) | 0.38(1.24) | 0.16(2.4, -0.04) | 0.42(1.57, 0.10) | 0.15(2.0) | 0.37(1.40) | 0.18(2.6, 0.18) | 0.43(1.37, 0.16) |

[a] In parentheses is the number of QTL identified by QP based on the SE model; [b] In parentheses is the gain in prediction accuracy with GWP over QP based on the SE model; [c] The first value in parentheses is the number of QTL identified by QP based on the ME model; and the second one the gain

with ME over SE for QP; [d] The first value in parentheses is the gain in accuracy with GWP over QP based on the ME model; and the second one is the gain with ME over SE using GWP. Bold in parentheses indicates the number is not significant at $\alpha = 0.05$.
