# Supplementary material for: Accuracy of Across-Environment Genome-Wide Prediction in Maize Nested Association Mapping Populations
Source: G3 (Bethesda). 2013 Feb 1;3(2):263–72. doi: 10.1534/g3.112.005066 (PMC3564986; doi:10.1534/g3.112.005066)
Supplement: Supporting Information [file supp_3.2.263_TableS10.pdf]

Table S10 Accuracy of WP prediction for environment E4 with QP and GWP in CV1

	LL				LW			
	SE		ME		SE		ME	
PopId	QP[a]	GWP[b]	QP[c]	GWP[d]	QP[a]	GWP[b]	QP[c]	GWP[d]
1	0.31(2.5)	0.47(0.52)	0.36(3.3, 0.16)	0.51(0.42, 0.09)	0.26(2.7)	0.50(0.92)	0.21(2.1, -0.19)	0.54(1.57, 0.08)
2	0.06(1.7)	0.30(4.00)	0.02(1.6, -0.67)	0.32(15.00, 0.07)	0.22(2.4)	0.48(1.18)	0.14(1.8, -0.36)	0.51(2.64, 0.06)
3	0.08(1.3)	0.26(2.25)	0.04(1.7, -0.50)	0.27(5.75, 0.04)	0.29(2.5)	0.42(0.45)	0.30(3.0, **0.03**)	0.52(0.73, 0.24)
4	0.31(3.0)	0.52(0.68)	0.32(3.3, **0.03**)	0.56(0.75, 0.08)	0.35(2.8)	0.51(0.46)	0.26(2.4, -0.26)	0.54(1.08, 0.06)
5	0.08(1.8)	0.35(3.37)	0.12(3.0, 0.50)	0.39(2.25, 0.11)	0.08(2.1)	0.45(4.62)	0.04(1.6, -0.50)	0.47(10.75, 0.04)
6	0.18(2.6)	0.45(1.50)	0.23(2.8, 0.28)	0.51(1.22, 0.13)	0.08(1.7)	0.30(2.75)	0.03(1.6, -0.62)	0.36(11.00, 0.20)
7	0.34(2.5)	0.60(0.76)	0.29(2.4, -0.15)	0.62(1.14, 0.03)	0.39(3.4)	0.62(0.59)	0.29(2.5, -0.26)	0.65(1.24, 0.05)
8	0.21(2.1)	0.40(0.90)	0.15(2.0, -0.29)	0.43(1.87, 0.07)	0.27(2.3)	0.40(0.48)	0.22(1.9, -0.19)	0.45(1.05, 0.12)
9	0.10(1.9)	0.34(2.40)	0.10(2.5, **0.00**)	0.37(2.70, 0.09)	0.34(2.6)	0.47(0.38)	0.32(2.1, **-0.06**)	0.47(0.47, **0.00**)
10	0.16(2.4)	0.41(1.56)	0.15(2.8, **-0.06**)	0.43(1.87, 0.05)	0.26(3.2)	0.63(1.42)	0.24(3.1, **-0.08**)	0.64(1.67, 0.02)
11	0.14(2.1)	0.29(1.07)	0.25(3.0, 0.79)	0.38(0.52, 0.31)	0.18(2.6)	0.44(1.44)	0.23(3.1, 0.28)	0.49(1.13, 0.11)
12	0.17(2.7)	0.50(1.94)	0.18(2.6, **0.06**)	0.54(2.00, 0.08)	0.35(3.4)	0.57(0.63)	0.34(3.3, **-0.03**)	0.60(0.76, 0.05)
13	0.11(1.3)	0.24(1.18)	0.13(2.9, **0.18**)	0.34(1.62, 0.42)	0.17(1.9)	0.42(1.47)	0.23(3.5, 0.35)	0.49(1.13, 0.17)
14	0.08(2.2)	0.34(3.25)	0.11(2.3, 0.38)	0.37(2.36, 0.09)	0.25(2.3)	0.45(0.80)	0.15(1.9, -0.40)	0.49(2.27, 0.09)
15	0.20(1.8)	0.36(0.80)	0.11(1.9, -0.45)	0.37(2.36, 0.03)	0.30(3.2)	0.53(0.77)	0.23(2.8, -0.23)	0.56(1.43, 0.06)
16	0.07(1.3)	0.20(1.86)	0.13(2.0, 0.86)	0.28(1.15, 0.40)	0.29(2.9)	0.53(0.83)	0.30(3.2, **0.03**)	0.57(0.90, 0.08)
17	0.14(1.8)	0.34(1.43)	0.06(1.2, -0.57)	0.36(5.00, 0.06)	0.32(3.3)	0.56(0.75)	0.34(3.7, **0.06**)	0.61(0.79, 0.09)
18	0.05(1.2)	0.26(4.20)	0.02(1.2, -0.60)	0.28(13.00, 0.08)	0.20(2.0)	0.31(0.55)	0.28(2.5, 0.40)	0.38(0.36, 0.23)
19	0.06(1.7)	0.26(3.33)	0.09(2.1, 0.50)	0.31(2.44, 0.19)	0.42(3.4)	0.51(0.21)	0.35(2.7, -0.17)	0.55(0.57, 0.08)
20	0.13(2.5)	0.46(2.54)	0.10(2.4, -0.23)	0.48(3.80, 0.04)	0.49(2.6)	0.52(0.06)	0.39(2.7, -0.20)	0.55(0.41, 0.06)
21	0.37(3.1)	0.61(0.65)	0.37(3.4, **0.00**)	0.64(0.73, 0.05)	0.08(1.8)	0.36(3.50)	0.05(2.1, -0.38)	0.41(7.20, 0.14)
22	0.28(2.2)	0.47(0.68)	0.23(2.4, -0.18)	0.48(1.09, 0.02)	0.27(2.8)	0.55(1.04)	0.19(2.0, -0.30)	0.59(2.11, 0.07)
23	0.25(2.0)	0.39(0.56)	0.22(2.6, **-0.12**)	0.45(1.05, 0.15)	0.27(3.0)	0.48(0.78)	0.27(2.9, **0.00**)	0.51(0.89, 0.06)
24	0.19(1.8)	0.26(0.37)	0.06(1.8, -0.68)	0.29(3.83, 0.12)	0.31(3.2)	0.47(0.52)	0.33(3.0, **0.06**)	0.51(0.55, 0.09)
25	0.04(1.3)	0.26(5.50)	0.06(2.6, **0.50**)	0.33(4.50, 0.27)	0.27(2.4)	0.48(0.78)	0.28(2.4, **0.04**)	0.47(0.68, -0.02)
Mean	0.16(2.0)	0.37(1.27)	0.16(2.4, 0.00)	0.41(1.64, 0.10)	0.27(2.7)	0.48(0.78)	0.24(2.6, -0.10)	0.52(1.15, 0.08)

[a] In parentheses is the number of QTL identified by QP based on the SE model; [b] In parentheses is the gain in prediction accuracy with GWP over QP based on the SE model; [c] The first value in parentheses is the number of QTL identified by QP based on the ME model; and the second one the gain

with ME over SE for QP; [d] The first value in parentheses is the gain in accuracy with GWP over QP based on the ME model; and the second one is the gain with ME over SE using GWP. Bold in parentheses indicates the number is not significant at $\alpha = 0.05$.
