# Supplementary material for: Accuracy of Across-Environment Genome-Wide Prediction in Maize Nested Association Mapping Populations
Source: G3 (Bethesda). 2013 Feb 1;3(2):263–72. doi: 10.1534/g3.112.005066 (PMC3564986; doi:10.1534/g3.112.005066)
Supplement: Supporting Information [file supp_3.2.263_TableS11.pdf]

Table S11 Accuracy of AP prediction for environment E1 with QP and GWP in CV1

	SE		ME		SE		ME	
PopId	QP[a]	GWP[b]	QP[c]	GWP[d]	QP[a]	GWP[b]	QP[c]	GWP[d]
1	0.21(9.0)	0.31(0.48)	0.28(14.0, 0.33)	0.33(0.18, 0.06)	0.19(13.0)	0.25(0.32)	0.21(12.0, 0.11)	0.26(0.24, 0.04)
2	0.20(9.0)	0.19(-0.05)	0.21(14.0, **0.05**)	0.20(-0.05, 0.05)	0.37(13.0)	0.42(0.14)	0.39(12.0, 0.05)	0.42(0.08, **0.00**)
3	0.21(10.0)	0.21(**0.00**)	0.20(15.0, **-0.05**)	0.20(**0.00**, **-0.05**)	0.41(13.0)	0.54(0.32)	0.46(11.0, 0.12)	0.55(0.20, 0.02)
4	0.37(12.0)	0.41(0.11)	0.43(12.0, 0.16)	0.41(-0.05, **0.00**)	0.28(14.0)	0.29(0.04)	0.22(12.0, -0.21)	0.28(0.27, -0.03)
5	0.31(11.0)	0.28(-0.10)	0.23(14.0, -0.26)	0.26(0.13, -0.07)	0.16(13.0)	0.23(0.44)	0.10(12.0, -0.38)	0.24(1.40, 0.04)
6	0.40(10.0)	0.46(0.15)	0.46(13.0, 0.15)	0.47(0.02, 0.02)	0.36(13.0)	0.37(0.03)	0.40(12.0, 0.11)	0.39(-0.03, 0.05)
7	0.23(11.0)	0.24(0.04)	0.27(14.0, 0.17)	0.26(-0.04, 0.08)	0.34(12.0)	0.36(0.06)	0.36(12.0, 0.06)	0.39(0.08, 0.08)
8	0.41(9.0)	0.47(0.15)	0.44(15.0, 0.07)	0.47(0.07, **0.00**)	0.32(12.0)	0.39(0.22)	0.32(12.0, **0.00**)	0.39(0.22, **0.00**)
9	0.36(10.0)	0.36(**0.00**)	0.33(13.0, -0.08)	0.38(0.15, 0.06)	0.18(15.0)	0.16(-0.11)	0.22(13.0, 0.22)	0.17(-0.23, 0.06)
10	0.30(10.0)	0.37(0.23)	0.37(13.0, 0.23)	0.38(0.03, 0.03)	0.42(12.0)	0.46(0.10)	0.48(13.0, 0.14)	0.48(**0.00**, 0.04)
11	0.03(10.0)	0.07(1.33)	0.08(15.0, 1.67)	0.09(0.13, 0.29)	0.11(13.0)	0.17(0.55)	0.19(11.0, 0.73)	0.18(-0.05, 0.06)
12	0.34(9.0)	0.39(0.15)	0.37(15.0, 0.09)	0.40(0.08, 0.03)	0.51(12.0)	0.59(0.16)	0.54(11.0, 0.06)	0.61(0.13, 0.03)
13	0.28(12.0)	0.29(0.04)	0.35(13.0, 0.25)	0.32(-0.09, 0.10)	0.49(14.0)	0.42(-0.14)	0.50(12.0, **0.02**)	0.43(-0.14, 0.02)
14	0.35(10.0)	0.40(0.14)	0.38(12.0, 0.09)	0.40(0.05, **0.00**)	0.39(14.0)	0.36(-0.08)	0.41(12.0, 0.05)	0.38(-0.07, 0.06)
15	0.22(12.0)	0.24(0.09)	0.20(15.0, -0.09)	0.24(0.20, **0.00**)	0.27(14.0)	0.31(0.15)	0.23(12.0, -0.15)	0.31(0.35, **0.00**)
16	0.18(11.0)	0.27(0.50)	0.23(13.0, 0.28)	0.28(0.22, 0.04)	0.45(12.0)	0.52(0.16)	0.49(12.0, 0.09)	0.52(0.06, **0.00**)
17	0.29(11.0)	0.29(**0.00**)	0.35(13.0, 0.21)	0.30(-0.14, 0.03)	0.38(10.0)	0.43(0.13)	0.47(13.0, 0.24)	0.46(-0.02, 0.07)
18	0.16(10.0)	0.14(-0.13)	0.12(13.0, -0.25)	0.14(0.17, **0.00**)	0.21(14.0)	0.30(0.43)	0.30(13.0, 0.43)	0.31(0.03, 0.03)
19	0.29(12.0)	0.30(0.03)	0.32(15.0, 0.10)	0.30(-0.06, **0.00**)	0.21(14.0)	0.22(0.05)	0.26(12.0, 0.24)	0.23(-0.12, 0.05)
20	0.30(9.0)	0.42(0.40)	0.35(13.0, 0.17)	0.43(0.23, 0.02)	0.40(13.0)	0.43(0.07)	0.34(13.0, -0.15)	0.44(0.29, 0.02)
21	0.31(10.0)	0.33(0.06)	0.30(14.0, **-0.03**)	0.35(0.17, 0.06)	0.24(13.0)	0.32(0.33)	0.33(12.0, 0.38)	0.34(0.03, 0.06)
22	0.29(10.0)	0.35(0.21)	0.31(17.0, 0.07)	0.34(0.10, -0.03)	0.24(13.0)	0.29(0.21)	0.22(12.0, -0.08)	0.30(0.36, 0.03)
23	0.16(9.0)	0.21(0.31)	0.16(14.0, **0.00**)	0.20(0.25, -0.05)	0.13(14.0)	0.15(0.15)	0.23(12.0, 0.77)	0.18(-0.22, 0.20)
24	0.18(11.0)	0.27(0.50)	0.17(15.0, **-0.06**)	0.25(0.47, -0.07)	0.42(12.0)	0.45(0.07)	0.45(12.0, 0.07)	0.45(**0.00**, **0.00**)
25	0.35(12.0)	0.42(0.20)	0.41(15.0, 0.17)	0.42(0.02, **0.00**)	0.41(14.0)	0.47(0.15)	0.40(13.0, **-0.02**)	0.47(0.18, **0.00**)
Mean	0.27(10.4)	0.31(0.15)	0.29(14.0, 0.09)	0.31(0.07, 0.00)	0.32(13.0)	0.36(0.13)	0.34(12.1, 0.08)	0.37(0.09, 0.04)

[a] In parentheses is the number of QTL identified by QP based on the SE model; [b] In parentheses is the gain in prediction accuracy with GWP over QP based on the SE model; [c] The first value in parentheses is the number of QTL identified by QP based on the ME model; and the second one the gain

with ME over SE for QP; [d] The first value in parentheses is the gain in accuracy with GWP over QP based on the ME model; and the second one is the gain with ME over SE using GWP. Bold in parentheses indicates the number is not significant at $\alpha = 0.05$.
