# Supplementary material for: Accuracy of Across-Environment Genome-Wide Prediction in Maize Nested Association Mapping Populations
Source: G3 (Bethesda). 2013 Feb 1;3(2):263–72. doi: 10.1534/g3.112.005066 (PMC3564986; doi:10.1534/g3.112.005066)
Supplement: Supporting Information [file supp_3.2.263_TableS12.pdf]

Table S12 Accuracy of AP prediction for environment E2 with QP and GWP in CV1

	LL				LW			
	SE		ME		SE		ME	
PopId	QP[a]	GWP[b]	QP[c]	GWP[d]	QP[a]	GWP[b]	QP[c]	GWP[d]
1	0.27(12.0)	0.39(0.44)	0.34(14.0, 0.26)	0.38(0.12, -0.03)	0.13(11.0)	0.21(0.62)	0.22(12.0, 0.69)	0.24(0.09, 0.14)
2	0.27(13.0)	0.28(0.04)	0.26(14.0, -0.04)	0.29(0.12, 0.04)	0.31(11.0)	0.44(0.42)	0.35(12.0, 0.13)	0.45(0.29, 0.02)
3	0.21(15.0)	0.29(0.38)	0.28(15.0, 0.33)	0.28(**0.00**, -0.03)	0.42(15.0)	0.54(0.29)	0.51(11.0, 0.21)	0.54(0.06, **0.00**)
4	0.32(14.0)	0.38(0.19)	0.34(12.0, 0.06)	0.39(0.15, 0.03)	0.31(13.0)	0.34(0.10)	0.28(12.0, -0.10)	0.36(0.29, 0.06)
5	0.27(12.0)	0.33(0.22)	0.25(14.0, -0.07)	0.32(0.28, -0.03)	0.37(12.0)	0.47(0.27)	0.38(12.0, **0.03**)	0.48(0.26, 0.02)
6	0.42(14.0)	0.47(0.12)	0.42(13.0, **0.00**)	0.47(0.12, **0.00**)	0.30(13.0)	0.28(-0.07)	0.32(12.0, 0.07)	0.30(-0.06, 0.07)
7	0.21(12.0)	0.20(-0.05)	0.19(14.0, -0.10)	0.21(0.11, 0.05)	0.50(11.0)	0.60(0.20)	0.47(12.0, -0.06)	0.59(0.26, -0.02)
8	0.34(13.0)	0.25(-0.26)	0.34(15.0, **0.00**)	0.26(-0.24, 0.04)	0.29(13.0)	0.34(0.17)	0.30(12.0, 0.03)	0.35(0.17, 0.03)
9	0.23(13.0)	0.23(**0.00**)	0.28(13.0, 0.22)	0.22(-0.21, -0.04)	0.31(13.0)	0.34(0.10)	0.33(13.0, 0.06)	0.34(0.03, **0.00**)
10	0.42(13.0)	0.47(0.12)	0.43(13.0, 0.02)	0.47(0.09, **0.00**)	0.33(14.0)	0.41(0.24)	0.42(13.0, 0.27)	0.45(0.07, 0.10)
11	0.24(15.0)	0.29(0.21)	0.28(15.0, 0.17)	0.30(0.07, 0.03)	0.34(13.0)	0.37(0.09)	0.36(11.0, 0.06)	0.37(0.03, **0.00**)
12	0.36(13.0)	0.41(0.14)	0.41(15.0, 0.14)	0.41(**0.00**, **0.00**)	0.51(13.0)	0.62(0.22)	0.55(11.0, 0.08)	0.63(0.15, 0.02)
13	0.37(12.0)	0.35(-0.05)	0.28(13.0, -0.24)	0.35(0.25, **0.00**)	0.42(13.0)	0.41(-0.02)	0.42(12.0, **0.00**)	0.4(-0.05, -0.02)
14	0.31(13.0)	0.24(-0.23)	0.26(12.0, -0.16)	0.26(**0.00**, 0.08)	0.37(13.0)	0.37(**0.00**)	0.38(12.0, 0.03)	0.39(0.03, 0.05)
15	0.24(13.0)	0.30(0.25)	0.20(15.0, -0.17)	0.29(0.45, -0.03)	0.46(12.0)	0.53(0.15)	0.48(12.0, 0.04)	0.52(0.08, -0.02)
16	0.33(12.0)	0.42(0.27)	0.27(13.0, -0.18)	0.42(0.56, **0.00**)	0.47(13.0)	0.53(0.13)	0.50(12.0, 0.06)	0.54(0.08, 0.02)
17	0.07(16.0)	0.11(0.57)	0.12(13.0, 0.71)	0.12(**0.00**, 0.09)	0.57(12.0)	0.60(0.05)	0.58(13.0, 0.02)	0.59(0.02, -0.02)
18	0.13(14.0)	0.20(0.54)	0.09(13.0, -0.31)	0.19(1.11, -0.05)	0.21(13.0)	0.23(0.10)	0.22(13.0, **0.05**)	0.22(**0.00**, -0.04)
19	0.36(12.0)	0.39(0.08)	0.43(15.0, 0.19)	0.40(-0.07, 0.03)	0.40(12.0)	0.40(**0.00**)	0.39(12.0, **-0.03**)	0.39(**0.00**, -0.03)
20	0.27(12.0)	0.41(0.52)	0.30(13.0, 0.11)	0.41(0.37, **0.00**)	0.39(13.0)	0.44(0.13)	0.32(13.0, -0.18)	0.43(0.34, -0.02)
21	0.43(12.0)	0.47(0.09)	0.37(14.0, -0.14)	0.47(0.27, **0.00**)	0.40(12.0)	0.45(0.13)	0.40(12.0, **0.00**)	0.45(0.13, **0.00**)
22	0.41(13.0)	0.43(0.05)	0.37(17.0, -0.10)	0.43(0.16, **0.00**)	0.37(14.0)	0.40(0.08)	0.39(12.0, 0.05)	0.42(0.08, 0.05)
23	0.28(13.0)	0.35(0.25)	0.25(14.0, -0.11)	0.35(0.40, **0.00**)	0.38(12.0)	0.38(**0.00**)	0.40(12.0, 0.05)	0.40(**0.00**, 0.05)
24	0.15(14.0)	0.21(0.40)	0.20(15.0, 0.33)	0.21(0.05, **0.00**)	0.42(15.0)	0.45(0.07)	0.46(12.0, 0.10)	0.45(-0.02, **0.00**)
25	0.18(13.0)	0.24(0.33)	0.18(15.0, **0.00**)	0.24(0.33, **0.00**)	0.25(13.0)	0.35(0.40)	0.31(13.0, 0.24)	0.37(0.19, 0.06)
Mean	0.28(13.1)	0.32(0.14)	0.29(14.0, 0.01)	0.33(0.14, 0.01)	0.37(12.8)	0.42(0.14)	0.39(12.1, 0.06)	0.43(0.10, 0.02)

[a] In parentheses is the number of QTL identified by QP based on the SE model; [b] In parentheses is the gain in prediction accuracy with GWP over QP based on the SE model; [c] The first value in parentheses is the number of QTL identified by QP based on the ME model; and the second one the gain

with ME over SE for QP; [d] The first value in parentheses is the gain in accuracy with GWP over QP based on the ME model; and the second one is the gain with ME over SE using GWP. Bold in parentheses indicates the number is not significant at $\alpha = 0.05$.
