# Supplementary material for: Accuracy of Across-Environment Genome-Wide Prediction in Maize Nested Association Mapping Populations
Source: G3 (Bethesda). 2013 Feb 1;3(2):263–72. doi: 10.1534/g3.112.005066 (PMC3564986; doi:10.1534/g3.112.005066)
Supplement: Supporting Information [file supp_3.2.263_TableS13.pdf]

Table S13 Accuracy of AP prediction for environment E3 with QP and GWP in CV1

	LL				LW			
	SE		ME		SE		ME	
PopId	QP[a]	GWP[b]	QP[c]	GWP[d]	QP[a]	GWP[b]	QP[c]	GWP[d]
1	0.31(12.0)	0.37(0.19)	0.25(14.0, -0.19)	0.37(0.48, **0.00**)	0.24(10.0)	0.24(**0.00**)	0.27(12.0, 0.13)	0.25(-0.07, 0.04)
2	0.26(13.0)	0.27(0.04)	0.27(14.0, 0.04)	0.30(0.11, 0.11)	0.38(12.0)	0.46(0.21)	0.43(12.0, 0.13)	0.47(0.09, 0.02)
3	0.15(14.0)	0.22(0.47)	0.13(15.0, -0.13)	0.21(0.62, -0.05)	0.31(11.0)	0.37(0.19)	0.34(11.0, 0.10)	0.36(0.06, -0.03)
4	0.30(12.0)	0.42(0.40)	0.34(12.0, 0.13)	0.41(0.21, -0.02)	0.22(11.0)	0.22(**0.00**)	0.18(12.0, -0.18)	0.24(0.33, 0.09)
5	0.22(13.0)	0.20(-0.09)	0.17(14.0, -0.23)	0.21(0.24, 0.05)	0.37(10.0)	0.44(0.19)	0.37(12.0, **0.00**)	0.43(0.16, -0.02)
6	0.41(13.0)	0.47(0.15)	0.40(13.0, -0.02)	0.46(0.15, -0.02)	0.21(9.0)	0.26(0.24)	0.26(12.0, 0.24)	0.26(**0.00**, **0.00**)
7	0.21(13.0)	0.20(-0.05)	0.18(14.0, -0.14)	0.20(0.11, **0.00**)	0.21(8.0)	0.32(0.52)	0.27(12.0, 0.29)	0.33(0.22, 0.03)
8	0.27(14.0)	0.29(0.07)	0.35(15.0, 0.30)	0.29(-0.17, **0.00**)	0.23(11.0)	0.27(0.17)	0.26(12.0, 0.13)	0.28(0.08, 0.04)
9	0.25(13.0)	0.35(0.40)	0.34(13.0, 0.36)	0.36(0.06, 0.03)	0.31(10.0)	0.31(**0.00**)	0.32(13.0, 0.03)	0.30(-0.06, -0.03)
10	0.36(12.0)	0.47(0.31)	0.35(13.0, **-0.03**)	0.46(0.31, -0.02)	0.41(9.0)	0.56(0.37)	0.38(13.0, -0.07)	0.55(0.45, -0.02)
11	0.16(13.0)	0.22(0.38)	0.11(15.0, -0.31)	0.23(1.09, 0.05)	0.17(10.0)	0.26(0.53)	0.19(11.0, 0.12)	0.27(0.42, 0.04)
12	0.20(13.0)	0.32(0.60)	0.24(15.0, 0.20)	0.31(0.29, -0.03)	0.22(12.0)	0.34(0.55)	0.31(11.0, 0.41)	0.34(0.10, **0.00**)
13	0.39(13.0)	0.36(-0.08)	0.40(13.0, **0.03**)	0.39(-0.03, 0.08)	0.45(10.0)	0.4(-0.11)	0.45(12.0, **0.00**)	0.38(-0.16, -0.05)
14	0.19(13.0)	0.24(0.26)	0.17(12.0, -0.11)	0.24(0.41, **0.00**)	0.24(10.0)	0.26(0.08)	0.30(12.0, 0.25)	0.28(-0.07, 0.08)
15	0.20(13.0)	0.24(0.20)	0.18(15.0, -0.10)	0.25(0.39, 0.04)	0.44(9.0)	0.49(0.11)	0.45(12.0, 0.02)	0.50(0.11, 0.02)
16	0.16(12.0)	0.16(**0.00**)	0.16(13.0, **0.00**)	0.17(0.06, 0.06)	0.38(11.0)	0.46(0.21)	0.43(12.0, 0.13)	0.49(0.14, 0.07)
17	0.26(14.0)	0.29(0.12)	0.29(13.0, 0.12)	0.29(**0.00**, **0.00**)	0.26(10.0)	0.30(0.15)	0.28(13.0, 0.08)	0.33(0.18, 0.10)
18	0.06(13.0)	0.11(0.83)	0.17(13.0, 1.83)	0.13(-0.24, 0.18)	0.29(9.0)	0.32(0.10)	0.34(13.0, 0.17)	0.33(-0.03, 0.03)
19	0.39(13.0)	0.36(-0.08)	0.39(15.0, **0.00**)	0.35(-0.10, -0.03)	0.36(10.0)	0.29(-0.19)	0.35(12.0, -0.03)	0.30(-0.14, 0.03)
20	0.23(12.0)	0.36(0.57)	0.24(13.0, **0.04**)	0.38(0.58, 0.06)	0.39(12.0)	0.44(0.13)	0.41(13.0, 0.05)	0.46(0.12, 0.05)
21	0.26(13.0)	0.44(0.69)	0.32(14.0, 0.23)	0.46(0.44, 0.05)	0.38(9.0)	0.40(0.05)	0.40(12.0, 0.05)	0.41(0.02, 0.02)
22	0.22(13.0)	0.20(-0.09)	0.30(17.0, 0.36)	0.21(-0.30, 0.05)	0.20(11.0)	0.26(0.30)	0.25(12.0, 0.25)	0.26(0.04, **0.00**)
23	0.19(13.0)	0.22(0.16)	0.21(14.0, 0.11)	0.23(0.10, 0.05)	0.19(10.0)	0.27(0.42)	0.23(12.0, 0.21)	0.28(0.22, 0.04)
24	0.13(11.0)	0.19(0.46)	0.14(15.0, **0.08**)	0.19(0.36, **0.00**)	0.39(11.0)	0.36(-0.08)	0.41(12.0, 0.05)	0.38(-0.07, 0.06)
25	0.30(13.0)	0.39(0.30)	0.25(15.0, -0.17)	0.38(0.52, -0.03)	0.20(12.0)	0.24(0.20)	0.24(13.0, 0.20)	0.24(**0.00**, **0.00**)
Mean	0.24(12.8)	0.29(0.21)	0.25(14.0, 0.04)	0.30(0.20, 0.01)	0.30(10.3)	0.34(0.13)	0.32(12.1, 0.09)	0.35(0.09, 0.03)

[a] In parentheses is the number of QTL identified by QP based on the SE model; [b] In parentheses is the gain in prediction accuracy with GWP over QP based on the SE model; [c] The first value in parentheses is the number of QTL identified by QP based on the ME model; and the second one the gain

with ME over SE for QP; [d] The first value in parentheses is the gain in accuracy with GWP over QP based on the ME model; and the second one is the gain with ME over SE using GWP. Bold in parentheses indicates the number is not significant at $\alpha = 0.05$.
