# Supplementary material for: Accuracy of Across-Environment Genome-Wide Prediction in Maize Nested Association Mapping Populations
Source: G3 (Bethesda). 2013 Feb 1;3(2):263–72. doi: 10.1534/g3.112.005066 (PMC3564986; doi:10.1534/g3.112.005066)
Supplement: Supporting Information [file supp_3.2.263_TableS14.pdf]

Table S14 Accuracy of AP prediction for environment E4 with QP and GWP in CV1

	LL				LW			
	SE		ME		SE		ME	
PopId	QP[a]	GWP[b]	QP[c]	GWP[d]	QP[a]	GWP[b]	QP[c]	GWP[d]
1	0.25(15.0)	0.26(0.04)	0.18(14.0, -0.28)	0.27(0.5, 0.04)	0.26(13.0)	0.31(0.19)	0.30(12.0, 0.15)	0.31(0.03, **0.00**)
2	0.28(15.0)	0.25(-0.11)	0.19(14.0, -0.32)	0.23(0.21, -0.08)	0.39(13.0)	0.43(0.10)	0.39(12.0, **0.00**)	0.43(0.10, **0.00**)
3	0.25(17.0)	0.24(-0.04)	0.15(15.0, -0.40)	0.25(0.67, 0.04)	0.38(12.0)	0.39(0.03)	0.35(11.0, -0.08)	0.40(0.14, 0.03)
4	0.40(13.0)	0.42(0.05)	0.36(12.0, -0.10)	0.43(0.19, 0.02)	0.25(13.0)	0.29(0.16)	0.26(12.0, **0.04**)	0.29(0.12, **0.00**)
5	0.23(15.0)	0.38(0.65)	0.23(14.0, **0.00**)	0.38(0.65, **0.00**)	0.31(13.0)	0.33(0.06)	0.29(12.0, -0.06)	0.36(0.24, 0.09)
6	0.35(15.0)	0.46(0.31)	0.42(13.0, 0.20)	0.48(0.14, 0.04)	0.27(13.0)	0.33(0.22)	0.29(12.0, 0.07)	0.33(0.14, **0.00**)
7	0.16(13.0)	0.32(1.00)	0.19(14.0, 0.19)	0.31(0.63, -0.03)	0.45(12.0)	0.52(0.16)	0.47(12.0, 0.04)	0.53(0.13, 0.02)
8	0.33(14.0)	0.34(0.03)	0.38(15.0, 0.15)	0.35(-0.08, 0.03)	0.26(12.0)	0.39(0.50)	0.30(12.0, 0.15)	0.40(0.33, 0.03)
9	0.34(15.0)	0.35(0.03)	0.32(13.0, -0.06)	0.36(0.13, 0.03)	0.34(13.0)	0.40(0.18)	0.35(13.0, 0.03)	0.41(0.17, 0.02)
10	0.40(16.0)	0.46(0.15)	0.34(13.0, -0.15)	0.48(0.41, 0.04)	0.47(13.0)	0.53(0.13)	0.47(13.0, **0.00**)	0.54(0.15, 0.02)
11	0.22(13.0)	0.27(0.23)	0.17(15.0, -0.23)	0.27(0.59, **0.00**)	0.35(12.0)	0.36(0.03)	0.34(11.0, -0.03)	0.36(0.06, **0.00**)
12	0.31(12.0)	0.31(0.00)	0.34(15.0, 0.10)	0.34(**0.00**, 0.10)	0.56(12.0)	0.57(0.02)	0.54(11.0, -0.04)	0.58(0.07, 0.02)
13	0.25(14.0)	0.16(-0.36)	0.20(13.0, -0.20)	0.16(-0.20, **0.00**)	0.32(13.0)	0.32(0.00)	0.37(12.0, 0.16)	0.35(-0.05, 0.09)
14	0.29(13.0)	0.35(0.21)	0.18(12.0, -0.38)	0.33(0.83, -0.06)	0.32(13.0)	0.33(0.03)	0.36(12.0, 0.12)	0.33(-0.08, **0.00**)
15	0.10(12.0)	0.20(1.00)	0.15(15.0, 0.50)	0.21(0.40, 0.05)	0.58(13.0)	0.60(0.03)	0.57(12.0, -0.02)	0.60(0.05, **0.00**)
16	0.17(12.0)	0.23(0.35)	0.15(13.0, -0.12)	0.24(0.60, 0.04)	0.47(12.0)	0.57(0.21)	0.52(12.0, 0.11)	0.58(0.12, 0.02)
17	0.15(16.0)	0.25(0.67)	0.12(13.0, -0.20)	0.24(1.00, -0.04)	0.55(13.0)	0.53(-0.04)	0.53(13.0, -0.04)	0.53(**0.00**, **0.00**)
18	0.13(14.0)	0.13(**0.00**)	0.16(13.0, 0.23)	0.13(-0.19, **0.00**)	0.40(13.0)	0.38(-0.05)	0.39(13.0, -0.03)	0.38(-0.03, **0.00**)
19	0.21(14.0)	0.19(-0.10)	0.22(15.0, **0.05**)	0.19(-0.14, **0.00**)	0.38(13.0)	0.36(-0.05)	0.43(12.0, 0.13)	0.36(-0.16, **0.00**)
20	0.30(12.0)	0.41(0.37)	0.33(13.0, 0.10)	0.41(0.24, **0.00**)	0.44(12.0)	0.52(0.18)	0.42(13.0, -0.05)	0.52(0.24, **0.00**)
21	0.45(14.0)	0.52(0.16)	0.42(14.0, -0.07)	0.52(0.24, **0.00**)	0.37(13.0)	0.41(0.11)	0.43(12.0, 0.16)	0.44(0.02, 0.07)
22	0.20(14.0)	0.25(0.25)	0.21(17.0, **0.05**)	0.24(0.14, -0.04)	0.31(12.0)	0.40(0.29)	0.31(12.0, **0.00**)	0.40(0.29, **0.00**)
23	0.13(17.0)	0.13(**0.00**)	0.10(14.0, -0.23)	0.13(0.30, **0.00**)	0.29(13.0)	0.30(0.03)	0.32(12.0, 0.10)	0.30(-0.06, **0.00**)
24	0.18(14.0)	0.29(0.61)	0.14(15.0, -0.22)	0.28(1.00, -0.03)	0.48(13.0)	0.50(0.04)	0.53(12.0, 0.10)	0.50(-0.06, **0.00**)
25	0.19(11.0)	0.29(0.53)	0.18(15.0, -0.05)	0.30(0.67, 0.03)	0.36(13.0)	0.44(0.22)	0.32(13.0, -0.11)	0.43(0.34, -0.02)
Mean	0.25(14.0)	0.30(0.20)	0.23(14.0, -0.07)	0.30(0.30, 0.00)	0.38(12.7)	0.42(0.11)	0.39(12.1, 0.03)	0.43(0.10, 0.01)

[a] In parentheses is the number of QTL identified by QP based on the SE model; [b] In parentheses is the gain in prediction accuracy with GWP over QP based on the SE model; [c] The first value in parentheses is the number of QTL identified by QP based on the ME model; and the second one the gain

with ME over SE for QP; [d] The first value in parentheses is the gain in accuracy with GWP over QP based on the ME model; and the second one is the gain with ME over SE using GWP. Bold in parentheses indicates the number is not significant at $\alpha = 0.05$.
