# Supplementary material for: Accuracy of Across-Environment Genome-Wide Prediction in Maize Nested Association Mapping Populations
Source: G3 (Bethesda). 2013 Feb 1;3(2):263–72. doi: 10.1534/g3.112.005066 (PMC3564986; doi:10.1534/g3.112.005066)
Supplement: Supporting Information [file supp_3.2.263_TableS15.pdf]

Table S15 Accuracy of WP prediction for environment E1 with QP and GWP in CV2

	LL				LW			
	SE		ME		SE		ME	
PopId	QP[a]	GWP[b]	QP[c]	GWP[d]	QP[a]	GWP[b]	QP[c]	GWP[d]
1	0.23(1.6)	0.33(0.43)	0.41(3.4, 0.78)	0.45(0.10, 0.36)	0.06(1.3)	0.31(4.17)	0.18(1.6, 2.00)	0.49(1.72, 0.58)
2	0.03(1.1)	0.18(5.00)	0.06(0.8, 1.00)	0.42(6.00, 1.33)	0.21(2.0)	0.40(0.90)	0.24(1.8, **0.14**)	0.55(1.29, 0.38)
3	0.09(1.2)	0.26(1.89)	0.06(0.8, **-0.33**)	0.37(5.17, 0.42)	0.39(3.3)	0.56(0.44)	0.43(2.6, 0.10)	0.68(0.58, 0.21)
4	0.24(2.8)	0.49(1.04)	0.34(3.2, 0.42)	0.60(0.76, 0.22)	0.23(2.4)	0.50(1.17)	0.23(1.6, **0.00**)	0.61(1.65, 0.22)
5	0.14(1.8)	0.33(1.36)	0.31(1.9, 1.21)	0.54(0.74, 0.64)	0.06(1.3)	0.21(2.50)	0.10(0.9, 0.67)	0.46(3.60, 1.19)
6	0.25(2.4)	0.50(1.00)	0.31(2.3, 0.24)	0.59(0.90, 0.18)	0.02(1.6)	0.31(14.50)	0.05(1.0, 1.50)	0.43(7.60, 0.39)
7	0.34(2.6)	0.52(0.53)	0.38(1.9, 0.12)	0.67(0.76, 0.29)	0.23(2.5)	0.48(1.09)	0.29(1.9, 0.26)	0.61(1.10, 0.27)
8	0.07(1.7)	0.28(3.00)	0.19(1.7, 1.71)	0.51(1.68, 0.82)	0.07(1.5)	0.30(3.29)	0.28(2.2, 3.00)	0.49(0.75, 0.63)
9	0.30(2.1)	0.35(0.17)	0.31(1.7, **0.03**)	0.50(0.61, 0.43)	0.02(1.0)	0.18(8.00)	0.21(1.7, 9.50)	0.42(1.00, 1.33)
10	0.26(2.5)	0.46(0.77)	0.32(2.1, 0.23)	0.64(1.00, 0.39)	0.15(2.2)	0.37(1.47)	0.27(2.4, 0.80)	0.62(1.30, 0.68)
11	0.30(2.5)	0.40(0.33)	0.41(2.6, 0.37)	0.56(0.37, 0.40)	0.12(2.3)	0.40(2.33)	0.16(1.7, 0.33)	0.48(2.00, 0.20)
12	0.25(3.0)	0.50(1.00)	0.33(2.9, 0.32)	0.65(0.97, 0.30)	0.30(3.1)	0.54(0.80)	0.40(2.8, 0.33)	0.64(0.60, 0.19)
13	0.21(2.7)	0.45(1.14)	0.27(1.6, 0.29)	0.64(1.37, 0.42)	0.30(2.6)	0.49(0.63)	0.33(1.7, **0.10**)	0.61(0.85, 0.24)
14	0.10(2.0)	0.36(2.60)	0.21(1.6, 1.10)	0.55(1.62, 0.53)	0.20(2.3)	0.44(1.20)	0.22(1.4, **0.10**)	0.62(1.82, 0.41)
15	0.08(1.7)	0.28(2.50)	0.17(1.4, 1.12)	0.47(1.76, 0.68)	0.13(1.7)	0.30(1.31)	0.25(2.5, 0.92)	0.43(0.72, 0.43)
16	0.20(1.7)	0.34(0.70)	0.23(1.2, 0.15)	0.48(1.09, 0.41)	0.25(2.3)	0.45(0.80)	0.38(2.7, 0.52)	0.56(0.47, 0.24)
17	0.05(1.2)	0.27(4.40)	0.09(0.8, 0.80)	0.39(3.33, 0.44)	0.32(3.0)	0.55(0.72)	0.43(3.5, 0.34)	0.68(0.58, 0.24)
18	0.10(1.4)	0.27(1.70)	0.06(0.6, -0.40)	0.36(5.00, 0.33)	0.19(2.0)	0.40(1.11)	0.22(1.6, **0.16**)	0.47(1.14, 0.17)
19	0.27(2.2)	0.37(0.37)	0.21(1.5, -0.22)	0.52(1.48, 0.41)	0.28(2.3)	0.41(0.46)	0.37(2.1, 0.32)	0.58(0.57, 0.41)
20	0.19(2.6)	0.47(1.47)	0.16(1.5, **-0.16**)	0.61(2.81, 0.30)	0.23(2.3)	0.48(1.09)	0.34(2.2, 0.48)	0.60(0.76, 0.25)
21	0.33(3.0)	0.48(0.45)	0.42(3.3, 0.27)	0.61(0.45, 0.27)	0.18(2.1)	0.47(1.61)	0.16(1.7, **-0.11**)	0.54(2.38, 0.15)
22	0.15(1.3)	0.36(1.40)	0.25(1.4, 0.67)	0.51(1.04, 0.42)	0.05(1.4)	0.31(5.20)	0.18(1.6, 2.60)	0.47(1.61, 0.52)
23	0.32(3.0)	0.44(0.38)	0.27(2.0, -0.16)	0.54(1.00, 0.23)	0.08(1.5)	0.30(2.75)	0.25(2.4, 2.12)	0.54(1.16, 0.80)
24	0.20(2.3)	0.48(1.40)	0.12(1.1, -0.40)	0.57(3.75, 0.19)	0.35(3.2)	0.50(0.43)	0.45(2.7, 0.29)	0.64(0.42, 0.28)
25	0.18(2.6)	0.50(1.78)	0.20(1.8, 0.11)	0.61(2.05, 0.22)	0.31(3.1)	0.47(0.52)	0.32(2.2, **0.03**)	0.60(0.87, 0.28)
Mean	0.20(2.1)	0.39(0.98)	0.24(1.8, 0.25)	0.53(1.19, 0.38)	0.19(2.2)	0.41(1.14)	0.27(2.0, 0.42)	0.55(1.05, 0.36)

[a] In parentheses is the number of QTL identified by QP based on the SE model; [b] In parentheses is the gain in prediction accuracy with GWP over QP based on the SE model; [c] The first value in parentheses is the number of QTL identified by QP based on the ME model; and the second one the gain

with ME over SE for QP; [d] The first value in parentheses is the gain in accuracy with GWP over QP based on the ME model; and the second one is the gain in accuracy with ME over SE using GWP. Bold in parentheses indicates the number is not significant at $\alpha = 0.05$.
