# Supplementary material for: Accuracy of Across-Environment Genome-Wide Prediction in Maize Nested Association Mapping Populations
Source: G3 (Bethesda). 2013 Feb 1;3(2):263–72. doi: 10.1534/g3.112.005066 (PMC3564986; doi:10.1534/g3.112.005066)
Supplement: Supporting Information [file supp_3.2.263_TableS16.pdf]

Table S16 Accuracy of WP prediction for environment E2 with QP and GWP in CV2

	LL				LW			
	SE		ME		SE		ME	
PopId	QP[a]	GWP[b]	QP[c]	GWP[d]	QP[a]	GWP[b]	QP[c]	GWP[d]
1	0.50(3.8)	0.67(0.34)	0.58(3.4, 0.16)	0.75(0.29, 0.12)	0.20(2.5)	0.46(1.30)	0.24(1.6, **0.20**)	0.57(1.37, 0.24)
2	0.10(2.1)	0.40(3.00)	0.08(0.8, **-0.20**)	0.56(6.00, 0.40)	0.21(2.6)	0.48(1.29)	0.28(1.8, 0.33)	0.64(1.29, 0.33)
3	0.24(1.7)	0.20(-0.17)	0.08(0.8, -0.67)	0.30(2.75, 0.50)	0.34(3.3)	0.60(0.76)	0.45(2.6, 0.32)	0.70(0.56, 0.17)
4	0.22(3.0)	0.56(1.55)	0.35(3.2, 0.59)	0.68(0.94, 0.21)	0.41(3.1)	0.61(0.49)	0.32(1.6, -0.22)	0.70(1.19, 0.15)
5	0.27(2.8)	0.50(0.85)	0.31(1.9, 0.15)	0.64(1.06, 0.28)	0.08(2.3)	0.46(4.75)	0.15(0.9, 0.87)	0.64(3.27, 0.39)
6	0.22(2.8)	0.47(1.14)	0.25(2.3, **0.14**)	0.58(1.32, 0.23)	0.06(1.2)	0.31(4.17)	0.09(1.0, **0.50**)	0.37(3.11, 0.19)
7	0.27(2.1)	0.41(0.52)	0.33(1.9, 0.22)	0.62(0.88, 0.51)	0.34(2.9)	0.59(0.74)	0.35(1.9, **0.03**)	0.72(1.06, 0.22)
8	0.10(2.0)	0.34(2.40)	0.21(1.7, 1.10)	0.51(1.43, 0.50)	0.24(1.8)	0.36(0.50)	0.35(2.2, 0.46)	0.53(0.51, 0.47)
9	0.10(1.9)	0.33(2.30)	0.18(1.7, 0.80)	0.46(1.56, 0.39)	0.23(2.3)	0.47(1.04)	0.34(1.7, 0.48)	0.54(0.59, 0.15)
10	0.22(2.9)	0.51(1.32)	0.31(2.1, 0.41)	0.65(1.10, 0.27)	0.13(1.9)	0.36(1.77)	0.26(2.4, 1.00)	0.46(0.77, 0.28)
11	0.27(3.0)	0.52(0.93)	0.40(2.6, 0.48)	0.62(0.55, 0.19)	0.29(2.1)	0.41(0.41)	0.29(1.7, **0.00**)	0.54(0.86, 0.32)
12	0.24(3.1)	0.55(1.29)	0.36(2.9, 0.50)	0.68(0.89, 0.24)	0.29(3.6)	0.58(1.00)	0.38(2.8, 0.31)	0.65(0.71, 0.12)
13	0.22(2.3)	0.39(0.77)	0.32(1.6, 0.45)	0.65(1.03, 0.67)	0.18(2.2)	0.42(1.33)	0.31(1.7, 0.72)	0.60(0.94, 0.43)
14	0.32(2.7)	0.42(0.31)	0.25(1.6, -0.22)	0.58(1.32, 0.38)	0.04(1.4)	0.25(5.25)	0.12(1.4, 2.00)	0.45(2.75, 0.80)
15	0.26(3.0)	0.48(0.85)	0.27(1.4, **0.04**)	0.65(1.41, 0.35)	0.23(3.2)	0.55(1.39)	0.32(2.5, 0.39)	0.67(1.09, 0.22)
16	0.19(2.0)	0.33(0.74)	0.21(1.2, **0.11**)	0.46(1.19, 0.39)	0.42(3.3)	0.55(0.31)	0.46(2.7, 0.10)	0.64(0.39, 0.16)
17	0.05(0.8)	0.12(1.40)	0.09(0.8, 0.80)	0.29(2.22, 1.42)	0.41(3.6)	0.61(0.49)	0.47(3.5, 0.15)	0.73(0.55, 0.20)
18	0.02(1.3)	0.27(12.50)	0.07(0.6, 2.50)	0.40(4.71, 0.48)	0.17(1.7)	0.31(0.82)	0.24(1.6, 0.41)	0.50(1.08, 0.61)
19	0.07(1.7)	0.27(2.86)	0.11(1.5, 0.57)	0.40(2.64, 0.48)	0.20(2.2)	0.41(1.05)	0.34(2.1, 0.70)	0.57(0.68, 0.39)
20	0.12(2.3)	0.52(3.33)	0.16(1.5, 0.33)	0.66(3.12, 0.27)	0.32(2.6)	0.54(0.69)	0.40(2.2, 0.25)	0.69(0.72, 0.28)
21	0.35(3.5)	0.58(0.66)	0.48(3.3, 0.37)	0.70(0.46, 0.21)	0.13(1.9)	0.33(1.54)	0.20(1.7, 0.54)	0.44(1.20, 0.33)
22	0.32(2.6)	0.50(0.56)	0.24(1.4, -0.25)	0.60(1.50, 0.20)	0.32(3.0)	0.59(0.84)	0.27(1.6, -0.16)	0.68(1.52, 0.15)
23	0.16(2.3)	0.43(1.69)	0.19(2.0, **0.19**)	0.53(1.79, 0.23)	0.23(2.3)	0.37(0.61)	0.27(2.4, 0.17)	0.49(0.81, 0.32)
24	0.01(1.0)	0.16(15.00)	0.10(1.1, 9.00)	0.44(3.40, 1.75)	0.24(2.2)	0.45(0.88)	0.42(2.7, 0.75)	0.62(0.48, 0.38)
25	0.08(2.0)	0.40(4.00)	0.14(1.8, 0.75)	0.57(3.07, 0.42)	0.29(2.8)	0.48(0.66)	0.37(2.2, 0.28)	0.54(0.46, 0.13)
Mean	0.20(2.3)	0.41(1.10)	0.24(1.8, 0.23)	0.56(1.30, 0.35)	0.24(2.5)	0.46(0.92)	0.31(2.0, 0.28)	0.59(0.91, 0.27)

[a] In parentheses is the number of QTL identified by QP based on the SE model; [b] In parentheses is the gain in prediction accuracy with GWP over QP based on the SE model; [c] The first value in parentheses is the number of QTL identified by QP based on the ME model; and the second one the gain

with ME over SE for QP; [d] The first value in parentheses is the gain in accuracy with GWP over QP based on the ME model; and the second one is the gain in accuracy with ME over SE using GWP. Bold in parentheses indicates the number is not significant at $\alpha = 0.05$.
