# Supplementary material for: Accuracy of Across-Environment Genome-Wide Prediction in Maize Nested Association Mapping Populations
Source: G3 (Bethesda). 2013 Feb 1;3(2):263–72. doi: 10.1534/g3.112.005066 (PMC3564986; doi:10.1534/g3.112.005066)
Supplement: Supporting Information [file supp_3.2.263_TableS17.pdf]

Table S17 Accuracy of WP prediction for environment E3 with QP and GWP in CV2

	LL				LW			
	SE		ME		SE		ME	
PopId	QP[a]	GWP[b]	QP[c]	GWP[d]	QP[a]	GWP[b]	QP[c]	GWP[d]
1	0.34(2.6)	0.51(0.50)	0.47(3.4, 0.38)	0.65(0.38, 0.27)	0.12(1.7)	0.36(2.00)	0.15(1.6, **0.25**)	0.45(2.00, 0.25)
2	0.12(1.9)	0.36(2.00)	0.08(0.8, -0.33)	0.51(5.38, 0.42)	0.22(2.7)	0.52(1.36)	0.26(1.8, **0.18**)	0.65(1.50, 0.25)
3	0.08(1.5)	0.23(1.88)	0.07(0.8, **-0.12**)	0.35(4.00, 0.52)	0.25(2.0)	0.26(0.04)	0.36(2.6, 0.44)	0.46(0.28, 0.77)
4	0.31(2.6)	0.51(0.65)	0.37(3.2, 0.19)	0.60(0.62, 0.18)	0.01(1.1)	0.19(18.00)	0.13(1.6, 12.00)	0.38(1.92, 1.00)
5	0.23(2.5)	0.46(1.00)	0.29(1.9, 0.26)	0.60(1.07, 0.30)	0.03(1.4)	0.30(9.00)	0.08(0.9, 1.67)	0.52(5.50, 0.73)
6	0.34(2.4)	0.47(0.38)	0.32(2.3, **-0.06**)	0.59(0.84, 0.26)	0.08(1.8)	0.31(2.87)	0.08(1.0, **0.00**)	0.43(4.38, 0.39)
7	0.28(2.3)	0.49(0.75)	0.31(1.9, **0.11**)	0.64(1.06, 0.31)	0.22(2.1)	0.42(0.91)	0.29(1.9, 0.32)	0.59(1.03, 0.40)
8	0.09(1.8)	0.32(2.56)	0.17(1.7, 0.89)	0.50(1.94, 0.56)	0.13(2.4)	0.46(2.54)	0.26(2.2, 1.00)	0.54(1.08, 0.17)
9	0.19(2.1)	0.42(1.21)	0.28(1.7, 0.47)	0.54(0.93, 0.29)	0.18(2.5)	0.45(1.50)	0.31(1.7, 0.72)	0.57(0.84, 0.27)
10	0.10(2.3)	0.42(3.20)	0.27(2.1, 1.70)	0.62(1.30, 0.48)	0.32(3.5)	0.55(0.72)	0.31(2.4, **-0.03**)	0.62(1.00, 0.13)
11	0.30(2.7)	0.53(0.77)	0.39(2.6, 0.30)	0.63(0.62, 0.19)	0.12(2.0)	0.38(2.17)	0.17(1.7, 0.42)	0.52(2.06, 0.37)
12	0.21(2.3)	0.42(1.00)	0.29(2.9, 0.38)	0.56(0.93, 0.33)	0.05(1.4)	0.27(4.40)	0.23(2.8, 3.60)	0.41(0.78, 0.52)
13	0.19(2.6)	0.43(1.26)	0.30(1.6, 0.58)	0.61(1.03, 0.42)	0.06(1.9)	0.27(3.50)	0.26(1.7, 3.33)	0.51(0.96, 0.89)
14	0.19(1.5)	0.33(0.74)	0.17(1.6, **-0.11**)	0.48(1.82, 0.45)	0.09(1.5)	0.33(2.67)	0.18(1.4, 1.00)	0.53(1.94, 0.61)
15	0.05(1.2)	0.25(4.00)	0.17(1.4, 2.40)	0.46(1.71, 0.84)	0.17(2.6)	0.39(1.29)	0.27(2.5, 0.59)	0.57(1.11, 0.46)
16	0.08(1.8)	0.33(3.12)	0.10(1.2, **0.25**)	0.43(3.30, 0.30)	0.09(1.8)	0.36(3.00)	0.31(2.7, 2.44)	0.52(0.68, 0.44)
17	0.10(1.6)	0.32(2.20)	0.12(0.8, **0.20**)	0.44(2.67, 0.38)	0.10(1.8)	0.37(2.70)	0.32(3.5, 2.20)	0.54(0.69, 0.46)
18	0.00(0.9)	0.18	0.05(0.6)	0.28(4.60, 0.56)	0.34(2.2)	0.35(0.03)	0.29(1.6, -0.15)	0.47(0.62, 0.34)
19	0.22(2.6)	0.46(1.09)	0.20(1.5, **-0.09**)	0.57(1.85, 0.24)	0.21(2.0)	0.41(0.95)	0.35(2.1, 0.67)	0.54(0.54, 0.32)
20	0.14(2.1)	0.32(1.29)	0.16(1.5, **0.14**)	0.49(2.06, 0.53)	0.17(2.2)	0.44(1.59)	0.33(2.2, 0.94)	0.58(0.76, 0.32)
21	0.25(2.7)	0.47(0.88)	0.46(3.3, 0.84)	0.63(0.37, 0.34)	0.21(2.4)	0.41(0.95)	0.21(1.7, **0.00**)	0.50(1.38, 0.22)
22	0.23(1.9)	0.39(0.70)	0.28(1.4, 0.22)	0.51(0.82, 0.31)	0.10(1.6)	0.34(2.40)	0.20(1.6, 1.00)	0.52(1.60, 0.53)
23	0.21(1.7)	0.31(0.48)	0.27(2.0, 0.29)	0.50(0.85, 0.61)	0.15(2.1)	0.40(1.67)	0.29(2.4, 0.93)	0.52(0.79, 0.30)
24	0.05(1.5)	0.26(4.20)	0.09(1.1, 0.80)	0.41(3.56, 0.58)	0.37(2.3)	0.41(0.11)	0.47(2.7, 0.27)	0.57(0.21, 0.39)
25	0.10(1.7)	0.36(2.60)	0.17(1.8, 0.70)	0.50(1.94, 0.39)	0.12(1.8)	0.29(1.42)	0.26(2.2, 1.17)	0.44(0.69, 0.52)
Mean	0.18(2.0)	0.38(1.17)	0.23(1.8, 0.33)	0.52(1.24, 0.37)	0.16(2.0)	0.37(1.36)	0.25(2.0, 0.63)	0.52(1.03, 0.40)

[a] In parentheses is the number of QTL identified by QP based on the SE model; [b] In parentheses is the gain in prediction accuracy with GWP over QP based on the SE model; [c] The first value in parentheses is the number of QTL identified by QP based on the ME model; and the second one the gain

with ME over SE for QP; [d] The first value in parentheses is the gain in accuracy with GWP over QP based on the ME model; and the second one is the gain in accuracy with ME over SE using GWP. Bold in parentheses indicates the number is not significant at $\alpha = 0.05$.
