# Supplementary material for: Accuracy of Across-Environment Genome-Wide Prediction in Maize Nested Association Mapping Populations
Source: G3 (Bethesda). 2013 Feb 1;3(2):263–72. doi: 10.1534/g3.112.005066 (PMC3564986; doi:10.1534/g3.112.005066)
Supplement: Supporting Information [file supp_3.2.263_TableS18.pdf]

**Table S18 Accuracy of WP prediction for environment E4 with QP and GWP in CV2**

| | LL | | | | LW | | | |
|---|---|---|---|---|---|---|---|---|
| | SE | | ME | | SE | | ME | |
| PopId | QP[a] | GWP[b] | QP[c] | GWP[d] | QP[a] | GWP[b] | QP[c] | GWP[d] |
| 1 | 0.31(2.4) | 0.46(0.48) | 0.47(3.4, 0.52) | 0.61(0.30, 0.33) | 0.25(2.5) | 0.51(1.04) | 0.25(1.6, **0.00**) | 0.62(1.48, 0.22) |
| 2 | 0.05(2.0) | 0.30(5.00) | 0.08(0.8, **0.60**) | 0.52(5.50, 0.73) | 0.20(2.5) | 0.46(1.30) | 0.25(1.8, 0.25) | 0.62(1.48, 0.35) |
| 3 | 0.08(1.3) | 0.27(2.38) | 0.06(0.8, **-0.25**) | 0.36(5.00, 0.33) | 0.29(2.6) | 0.41(0.41) | 0.40(2.6, 0.38) | 0.61(0.52, 0.49) |
| 4 | 0.31(3.1) | 0.53(0.71) | 0.43(3.2, 0.39) | 0.65(0.51, 0.23) | 0.33(2.8) | 0.52(0.58) | 0.25(1.6, -0.24) | 0.63(1.52, 0.21) |
| 5 | 0.07(1.8) | 0.37(4.29) | 0.18(1.9, 1.57) | 0.55(2.06, 0.49) | 0.07(2.2) | 0.44(5.29) | 0.13(0.9, 0.86) | 0.62(3.77, 0.41) |
| 6 | 0.22(2.7) | 0.47(1.14) | 0.31(2.3, 0.41) | 0.59(0.90, 0.26) | 0.08(1.4) | 0.31(2.87) | 0.10(1.0, **0.25**) | 0.46(3.60, 0.48) |
| 7 | 0.33(2.6) | 0.60(0.82) | 0.38(1.9, 0.15) | 0.73(0.92, 0.22) | 0.38(3.2) | 0.61(0.61) | 0.35(1.9, **-0.08**) | 0.72(1.06, 0.18) |
| 8 | 0.18(2.2) | 0.37(1.06) | 0.26(1.7, 0.44) | 0.59(1.27, 0.59) | 0.28(2.3) | 0.40(0.43) | 0.37(2.2, 0.32) | 0.57(0.54, 0.42) |
| 9 | 0.10(1.9) | 0.33(2.30) | 0.18(1.7, 0.80) | 0.48(1.67, 0.45) | 0.37(2.6) | 0.48(0.30) | 0.39(1.7, **0.05**) | 0.51(0.31, 0.06) |
| 10 | 0.13(2.2) | 0.42(2.23) | 0.25(2.1, 0.92) | 0.57(1.28, 0.36) | 0.26(3.3) | 0.62(1.38) | 0.32(2.4, 0.23) | 0.72(1.25, 0.16) |
| 11 | 0.16(2.1) | 0.29(0.81) | 0.37(2.6, 1.31) | 0.49(0.32, 0.69) | 0.18(2.6) | 0.46(1.56) | 0.24(1.7, 0.33) | 0.60(1.50, 0.30) |
| 12 | 0.15(2.7) | 0.50(2.33) | 0.32(2.9, 1.13) | 0.64(1.00, 0.28) | 0.38(3.4) | 0.57(0.50) | 0.45(2.8, 0.18) | 0.66(0.47, 0.16) |
| 13 | 0.10(1.3) | 0.24(1.40) | 0.30(1.6, 2.00) | 0.54(0.80, 1.25) | 0.16(2.1) | 0.42(1.62) | 0.28(1.7, 0.75) | 0.60(1.14, 0.43) |
| 14 | 0.06(1.9) | 0.35(4.83) | 0.19(1.6, 2.17) | 0.53(1.79, 0.51) | 0.24(2.3) | 0.44(0.83) | 0.23(1.4, **-0.04**) | 0.63(1.74, 0.43) |
| 15 | 0.21(1.9) | 0.37(0.76) | 0.23(1.4, **0.10**) | 0.57(1.48, 0.54) | 0.29(3.0) | 0.54(0.86) | 0.35(2.5, 0.21) | 0.68(0.94, 0.26) |
| 16 | 0.08(1.3) | 0.21(1.62) | 0.19(1.2, 1.38) | 0.42(1.21, 1.00) | 0.30(3.0) | 0.54(0.80) | 0.43(2.7, 0.43) | 0.66(0.53, 0.22) |
| 17 | 0.12(1.6) | 0.35(1.92) | 0.13(0.8, **0.08**) | 0.48(2.69, 0.37) | 0.31(3.3) | 0.56(0.81) | 0.42(3.5, 0.35) | 0.70(0.67, 0.25) |
| 18 | 0.04(1.3) | 0.26(5.50) | 0.06(0.6, **0.50**) | 0.37(5.17, 0.42) | 0.22(2.0) | 0.33(0.50) | 0.28(1.6, 0.27) | 0.45(0.61, 0.36) |
| 19 | 0.06(1.5) | 0.28(3.67) | 0.13(1.5, 1.17) | 0.45(2.46, 0.61) | 0.42(3.4) | 0.50(0.19) | 0.42(2.1, **0.00**) | 0.66(0.57, 0.32) |
| 20 | 0.11(2.2) | 0.47(3.27) | 0.14(1.5, 0.27) | 0.59(3.21, 0.26) | 0.49(2.6) | 0.53(0.08) | 0.45(2.2, -0.08) | 0.67(0.49, 0.26) |
| 21 | 0.38(3.4) | 0.61(0.61) | 0.49(3.3, 0.29) | 0.69(0.41, 0.13) | 0.10(1.8) | 0.36(2.60) | 0.18(1.7, 0.80) | 0.49(1.72, 0.36) |
| 22 | 0.30(2.3) | 0.46(0.53) | 0.28(1.4, **-0.07**) | 0.55(0.96, 0.20) | 0.25(2.8) | 0.54(1.16) | 0.26(1.6, **0.04**) | 0.67(1.58, 0.24) |
| 23 | 0.26(2.0) | 0.39(0.50) | 0.29(2.0, 0.12) | 0.50(0.72, 0.28) | 0.29(3.0) | 0.48(0.66) | 0.32(2.4, **0.10**) | 0.60(0.87, 0.25) |
| 24 | 0.19(1.9) | 0.23(0.21) | 0.13(1.1, -0.32) | 0.41(2.15, 0.78) | 0.31(3.1) | 0.46(0.48) | 0.44(2.7, 0.42) | 0.62(0.41, 0.35) |
| 25 | 0.02(1.5) | 0.24(11.00) | 0.12(1.8, 5.00) | 0.45(2.75, 0.88) | 0.27(2.5) | 0.49(0.81) | 0.38(2.2, 0.41) | 0.54(0.42, 0.10) |
| Mean | 0.16(2.0) | 0.38(1.38) | 0.24(1.8, 0.49) | 0.53(1.23, 0.39) | 0.27(2.7) | 0.48(0.78) | 0.32(2.0, 0.18) | 0.61(0.93, 0.28) |

[a] In parentheses is the number of QTL identified by QP based on the SE model; [b] In parentheses is the gain in prediction accuracy with GWP over QP based on the SE model; [c] The first value in parentheses is the number of QTL identified by QP based on the ME model; and the second one the gain

with ME over SE for QP; [d] The first value in parentheses is the gain in accuracy with GWP over QP based on the ME model; and the second one is the gain in accuracy with ME over SE using GWP. Bold in parentheses indicates the number is not significant at $\alpha = 0.05$.
