# Supplementary material for: Accuracy of Across-Environment Genome-Wide Prediction in Maize Nested Association Mapping Populations
Source: G3 (Bethesda). 2013 Feb 1;3(2):263–72. doi: 10.1534/g3.112.005066 (PMC3564986; doi:10.1534/g3.112.005066)
Supplement: Supporting Information [file supp_3.2.263_TableS19.pdf]

Table S19 Accuracy of AP prediction for environment E1 with QP and GWP in CV2

	LL				LW			
	SE		ME		SE		ME	
PopId	QP[a]	GWP[b]	QP[c]	GWP[d]	QP[a]	GWP[b]	QP[c]	GWP[d]
1	0.21(8.0)	0.31(0.48)	0.27(9.5, 0.29)	0.35(0.30, 0.13)	0.20(8.5)	0.27(0.35)	0.22(11.0, 0.10)	0.30(0.36, 0.11)
2	0.14(8.0)	0.17(0.21)	0.20(9.5, 0.43)	0.22(0.10, 0.29)	0.37(8.5)	0.41(0.11)	0.37(11.0, **0.00**)	0.44(0.19, 0.07)
3	0.20(8.0)	0.25(0.25)	0.22(9.5, **0.10**)	0.28(0.27, 0.12)	0.39(8.5)	0.55(0.41)	0.46(11.0, 0.18)	0.59(0.28, 0.07)
4	0.43(8.0)	0.45(0.05)	0.40(9.5, -0.07)	0.48(0.20, 0.07)	0.32(8.5)	0.33(0.03)	0.29(11.0, -0.09)	0.33(0.14, **0.00**)
5	0.26(8.0)	0.26(**0.00**)	0.21(9.5, -0.19)	0.28(0.33, 0.08)	0.14(8.5)	0.24(0.71)	0.15(11.0, **0.07**)	0.28(0.87, 0.17)
6	0.39(8.0)	0.47(0.21)	0.39(9.5, **0.00**)	0.51(0.31, 0.09)	0.33(8.5)	0.38(0.15)	0.39(11.0, 0.18)	0.41(0.05, 0.08)
7	0.27(8.0)	0.29(0.07)	0.32(9.5, 0.19)	0.36(0.13, 0.24)	0.33(8.5)	0.37(0.12)	0.39(11.0, 0.18)	0.44(0.13, 0.19)
8	0.36(8.0)	0.44(0.22)	0.40(9.5, 0.11)	0.49(0.23, 0.11)	0.28(8.5)	0.37(0.32)	0.33(11.0, 0.18)	0.40(0.21, 0.08)
9	0.33(8.0)	0.35(0.06)	0.36(9.5, 0.09)	0.39(0.08, 0.11)	0.16(8.5)	0.16(**0.00**)	0.18(11.0, 0.12)	0.18(**0.00**, 0.13)
10	0.32(8.0)	0.39(0.22)	0.39(9.5, 0.22)	0.44(0.13, 0.13)	0.39(8.5)	0.44(0.13)	0.44(11.0, 0.13)	0.50(0.14, 0.14)
11	0.09(8.0)	0.14(0.56)	0.11(9.5, 0.22)	0.20(0.82, 0.43)	0.16(8.5)	0.21(0.31)	0.23(11.0, 0.44)	0.24(0.04, 0.14)
12	0.35(8.0)	0.40(0.14)	0.40(9.5, 0.14)	0.46(0.15, 0.15)	0.50(8.5)	0.57(0.14)	0.53(11.0, 0.06)	0.60(0.13, 0.05)
13	0.25(8.0)	0.28(0.12)	0.38(9.5, 0.52)	0.38(**0.00**, 0.36)	0.41(8.5)	0.40(-0.02)	0.47(11.0, 0.15)	0.44(-0.06, 0.1)
14	0.37(8.0)	0.40(0.08)	0.39(9.5, 0.05)	0.44(0.13, 0.10)	0.37(8.5)	0.36(-0.03)	0.40(11.0, 0.08)	0.42(0.05, 0.17)
15	0.23(8.0)	0.25(0.09)	0.19(9.5, -0.17)	0.27(0.42, 0.08)	0.22(8.5)	0.33(0.50)	0.24(11.0, 0.09)	0.34(0.42, 0.03)
16	0.21(8.0)	0.30(0.43)	0.23(9.5, 0.10)	0.34(0.48, 0.13)	0.42(8.5)	0.51(0.21)	0.50(11.0, 0.19)	0.53(0.06, 0.04)
17	0.29(8.0)	0.29(**0.00**)	0.31(9.5, **0.07**)	0.33(0.06, 0.14)	0.37(8.5)	0.46(0.24)	0.46(11.0, 0.24)	0.51(0.11, 0.11)
18	0.14(8.0)	0.15(0.07)	0.14(9.5, **0.00**)	0.17(0.21, 0.13)	0.21(8.5)	0.30(0.43)	0.29(11.0, 0.38)	0.33(0.14, 0.10)
19	0.29(8.0)	0.32(0.10)	0.34(9.5, 0.17)	0.37(0.09, 0.16)	0.22(8.5)	0.26(0.18)	0.26(11.0, 0.18)	0.29(0.12, 0.12)
20	0.29(8.0)	0.42(0.45)	0.32(9.5, 0.10)	0.45(0.41, 0.07)	0.38(8.5)	0.44(0.16)	0.37(11.0, **-0.03**)	0.47(0.27, 0.07)
21	0.30(8.0)	0.33(0.10)	0.35(9.5, 0.17)	0.39(0.11, 0.18)	0.28(8.5)	0.32(0.14)	0.31(11.0, 0.11)	0.35(0.13, 0.09)
22	0.30(8.0)	0.34(0.13)	0.29(9.5, **-0.03**)	0.36(0.24, 0.06)	0.24(8.5)	0.31(0.29)	0.26(11.0, 0.08)	0.34(0.31, 0.10)
23	0.15(8.0)	0.22(0.47)	0.18(9.5, 0.20)	0.25(0.39, 0.14)	0.15(8.5)	0.17(0.13)	0.24(11.0, 0.60)	0.24(**0.00**, 0.41)
24	0.23(8.0)	0.32(0.39)	0.23(9.5, **0.00**)	0.32(0.39, **0.00**)	0.45(8.5)	0.47(0.04)	0.47(11.0, 0.04)	0.50(0.06, 0.06)
25	0.39(8.0)	0.43(0.10)	0.35(9.5, -0.10)	0.46(0.31, 0.07)	0.38(8.5)	0.48(0.26)	0.40(11.0, 0.05)	0.51(0.28, 0.06)
Mean	0.27(8.0)	0.32(0.19)	0.29(9.5, 0.09)	0.36(0.24, 0.12)	0.31(8.5)	0.36(0.16)	0.35(11.1, 0.13)	0.40(0.14, 0.10)

[a] In parentheses is the number of QTL identified by QP based on the SE model; [b] In parentheses is the gain in prediction accuracy with GWP over QP based on the SE model; [c] The first value in parentheses is the number of QTL identified by QP based on the ME model; and the second one the gain

with ME over SE for QP; [d] The first value in parentheses is the gain in accuracy with GWP over QP based on the ME model; the second one is the gain in accuracy with ME over SE using GWP; and the third one is the gain in accuracy with GWP over PP. Bold in parentheses indicates the number is not significant at $\alpha = 0.05$.
