# Supplementary material for: Accuracy of Across-Environment Genome-Wide Prediction in Maize Nested Association Mapping Populations
Source: G3 (Bethesda). 2013 Feb 1;3(2):263–72. doi: 10.1534/g3.112.005066 (PMC3564986; doi:10.1534/g3.112.005066)
Supplement: Supporting Information [file supp_3.2.263_TableS20.pdf]

Table S20 Accuracy of AP prediction for environment E2 with QP and GWP in CV2

	LL				LW			
	SE		ME		SE		ME	
PopId	QP[a]	GWP[b]	QP[c]	GWP[d]	QP[a]	GWP[b]	QP[c]	GWP[d]
1	0.33(9.8)	0.45(0.36)	0.30(9.5, -0.09)	0.47(0.57, 0.04)	0.22(10.6)	0.25(0.14)	0.28(11.0, 0.27)	0.30(0.07, 0.20)
2	0.27(9.8)	0.30(0.11)	0.28(9.5, 0.04)	0.36(0.29, 0.20)	0.35(10.6)	0.45(0.29)	0.39(11.0, 0.11)	0.50(0.28, 0.11)
3	0.28(9.8)	0.30(0.07)	0.26(9.5, -0.07)	0.32(0.23, 0.07)	0.47(10.6)	0.56(0.19)	0.52(11.0, 0.11)	0.60(0.15, 0.07)
4	0.33(9.8)	0.42(0.27)	0.34(9.5, **0.03**)	0.46(0.35, 0.10)	0.35(10.6)	0.38(0.09)	0.38(11.0, 0.09)	0.42(0.11, 0.11)
5	0.28(9.8)	0.33(0.18)	0.28(9.5, **0.00**)	0.37(0.32, 0.12)	0.38(10.6)	0.47(0.24)	0.41(11.0, 0.08)	0.52(0.27, 0.11)
6	0.39(9.8)	0.46(0.18)	0.43(9.5, 0.10)	0.49(0.14, 0.07)	0.31(10.6)	0.28(-0.10)	0.31(11.0, **0.00**)	0.30(-0.03, 0.07)
7	0.23(9.8)	0.23(**0.00**)	0.25(9.5, 0.09)	0.28(0.12, 0.22)	0.47(10.6)	0.60(0.28)	0.48(11.0, 0.02)	0.62(0.29, 0.03)
8	0.30(9.8)	0.27(-0.10)	0.34(9.5, 0.13)	0.32(-0.06, 0.19)	0.30(10.6)	0.36(0.20)	0.34(11.0, 0.13)	0.38(0.12, 0.06)
9	0.21(9.8)	0.21(**0.00**)	0.27(9.5, 0.29)	0.24(-0.11, 0.14)	0.32(10.6)	0.36(0.13)	0.38(11.0, 0.19)	0.39(0.03, 0.08)
10	0.41(9.8)	0.49(0.20)	0.40(9.5, **-0.02**)	0.52(0.30, 0.06)	0.35(10.6)	0.42(0.20)	0.41(11.0, 0.17)	0.48(0.17, 0.14)
11	0.27(9.8)	0.34(0.26)	0.28(9.5, **0.04**)	0.39(0.39, 0.15)	0.32(10.6)	0.37(0.16)	0.35(11.0, 0.09)	0.40(0.14, 0.08)
12	0.38(9.8)	0.42(0.11)	0.44(9.5, 0.16)	0.46(0.05, 0.10)	0.51(10.6)	0.60(0.18)	0.54(11.0, 0.06)	0.63(0.17, 0.05)
13	0.32(9.8)	0.36(0.13)	0.29(9.5, -0.09)	0.40(0.38, 0.11)	0.38(10.6)	0.41(0.08)	0.42(11.0, 0.11)	0.44(0.05, 0.07)
14	0.27(9.8)	0.27(**0.00**)	0.33(9.5, 0.22)	0.33(**0.00**, 0.22)	0.35(10.6)	0.38(0.09)	0.36(11.0, **0.03**)	0.41(0.14, 0.08)
15	0.23(9.8)	0.33(0.43)	0.25(9.5, **0.09**)	0.37(0.48, 0.12)	0.47(10.6)	0.54(0.15)	0.46(11.0, **-0.02**)	0.55(0.20, 0.02)
16	0.30(9.8)	0.41(0.37)	0.31(9.5, **0.03**)	0.45(0.45, 0.10)	0.48(10.6)	0.54(0.13)	0.51(11.0, 0.06)	0.56(0.10, 0.04)
17	0.05(9.8)	0.10(1.00)	0.08(9.5, 0.60)	0.14(0.75, 0.40)	0.58(10.6)	0.61(0.05)	0.58(11.0, 0.00)	0.62(0.07, 0.02)
18	0.15(9.8)	0.20(0.33)	0.15(9.5, **0.00**)	0.22(0.47, 0.10)	0.24(10.6)	0.26(0.08)	0.22(11.0, -0.08)	0.27(0.23, 0.04)
19	0.35(9.8)	0.40(0.14)	0.39(9.5, 0.11)	0.43(0.10, 0.07)	0.42(10.6)	0.42(**0.00**)	0.41(11.0, **-0.02**)	0.44(0.07, 0.05)
20	0.26(9.8)	0.42(0.62)	0.26(9.5, **0.00**)	0.44(0.69, 0.05)	0.39(10.6)	0.44(0.13)	0.38(11.0, **-0.03**)	0.46(0.21, 0.05)
21	0.39(9.8)	0.48(0.23)	0.42(9.5, 0.08)	0.51(0.21, 0.06)	0.37(10.6)	0.44(0.19)	0.38(11.0, **0.03**)	0.45(0.18, 0.02)
22	0.44(9.8)	0.44(**0.00**)	0.45(9.5, **0.02**)	0.47(0.04, 0.07)	0.39(10.6)	0.43(0.10)	0.36(11.0, -0.08)	0.47(0.31, 0.09)
23	0.30(9.8)	0.36(0.20)	0.29(9.5, **-0.03**)	0.39(0.34, 0.08)	0.37(10.6)	0.39(0.05)	0.39(11.0, 0.05)	0.42(0.08, 0.08)
24	0.19(9.8)	0.21(0.11)	0.19(9.5, **0.00**)	0.25(0.32, 0.19)	0.44(10.6)	0.49(0.11)	0.49(11.0, 0.11)	0.51(0.04, 0.04)
25	0.15(9.8)	0.27(0.80)	0.16(9.5, **0.07**)	0.31(0.94, 0.15)	0.30(10.6)	0.37(0.23)	0.35(11.0, 0.17)	0.42(0.20, 0.14)
Mean	0.28(9.8)	0.34(0.21)	0.30(9.5, 0.05)	0.37(0.23, 0.10)	0.38(10.6)	0.43(0.13)	0.40(11.1, 0.06)	0.46(0.15, 0.07)

[a] In parentheses is the number of QTL identified by QP based on the SE model; [b] In parentheses is the gain in prediction accuracy with GWP over QP based on the SE model; [c] The first value in parentheses is the number of QTL identified by QP based on the ME model; and the second one the

gain with ME over SE for QP; [d] The first value in parentheses is the gain in accuracy with GWP over QP based on the ME model; the second one is the gain in accuracy with ME over SE using GWP; and the third one is the gain in accuracy with GWP over PP. Bold in parentheses indicates the number is not significant at $\alpha = 0.05$.
