# Supplementary material for: Accuracy of Across-Environment Genome-Wide Prediction in Maize Nested Association Mapping Populations
Source: G3 (Bethesda). 2013 Feb 1;3(2):263–72. doi: 10.1534/g3.112.005066 (PMC3564986; doi:10.1534/g3.112.005066)
Supplement: Supporting Information [file supp_3.2.263_TableS21.pdf]

Table S21 Accuracy of AP prediction for environment E3 with QP and GWP in CV2

	LL				LW			
	SE		ME		SE		ME	
PopId	QP[a]	GWP[b]	QP[c]	GWP[d]	QP[a]	GWP[b]	QP[c]	GWP[d]
1	0.29(7.8)	0.41(0.41)	0.34(9.5, 0.17)	0.45(0.32, 0.10)	0.20(7.4)	0.23(0.15)	0.26(11.0, 0.30)	0.27(**0.01**, 0.17)
2	0.27(7.8)	0.29(0.07)	0.27(9.5, **0.00**)	0.35(0.30, 0.21)	0.41(7.4)	0.47(0.15)	0.44(11.0, 0.07)	0.52(0.18, 0.11)
3	0.11(7.8)	0.21(0.91)	0.14(9.5, 0.27)	0.23(0.64, 0.10)	0.34(7.4)	0.36(0.06)	0.32(11.0, -0.06)	0.38(0.19, 0.06)
4	0.29(7.8)	0.44(0.52)	0.31(9.5, **0.07**)	0.46(0.48, 0.05)	0.18(7.4)	0.22(0.22)	0.18(11.0, **0.00**)	0.26(0.44, 0.18)
5	0.22(7.8)	0.22(**0.00**)	0.24(9.5, 0.09)	0.27(0.13, 0.23)	0.38(7.4)	0.44(0.16)	0.37(11.0, **-0.03**)	0.46(0.24, 0.05)
6	0.39(7.8)	0.48(0.23)	0.37(9.5, -0.05)	0.50(0.35, 0.04)	0.19(7.4)	0.26(0.37)	0.20(11.0, **0.05**)	0.27(0.35, 0.04)
7	0.25(7.8)	0.25(**0.00**)	0.19(9.5, -0.24)	0.28(0.47, 0.12)	0.22(7.4)	0.32(0.45)	0.29(11.0, 0.32)	0.37(0.28, 0.16)
8	0.22(7.8)	0.28(0.27)	0.24(9.5, 0.09)	0.32(0.33, 0.14)	0.23(7.4)	0.29(0.26)	0.26(11.0, 0.13)	0.31(0.19, 0.07)
9	0.30(7.8)	0.36(0.20)	0.35(9.5, 0.17)	0.39(0.11, 0.08)	0.32(7.4)	0.33(0.03)	0.34(11.0, 0.06)	0.34(**0.00**, 0.03)
10	0.33(7.8)	0.48(0.45)	0.38(9.5, 0.15)	0.51(0.34, 0.06)	0.42(7.4)	0.55(0.31)	0.44(11.0, 0.05)	0.57(0.30, 0.04)
11	0.18(7.8)	0.27(0.50)	0.17(9.5, **-0.06**)	0.31(0.82, 0.15)	0.19(7.4)	0.27(0.42)	0.23(11.0, 0.21)	0.31(0.35, 0.15)
12	0.25(7.8)	0.32(0.28)	0.30(9.5, 0.20)	0.35(0.17, 0.09)	0.28(7.4)	0.32(0.14)	0.30(11.0, 0.07)	0.33(0.10, 0.03)
13	0.33(7.8)	0.38(0.15)	0.42(9.5, 0.27)	0.46(0.10, 0.21)	0.39(7.4)	0.40(0.03)	0.43(11.0, 0.10)	0.42(-0.02, 0.05)
14	0.19(7.8)	0.26(0.37)	0.19(9.5, **0.00**)	0.30(0.58, 0.15)	0.22(7.4)	0.26(0.18)	0.28(11.0, 0.27)	0.32(0.14, 0.23)
15	0.11(7.8)	0.25(1.27)	0.14(9.5, 0.27)	0.31(1.21, 0.24)	0.39(7.4)	0.49(0.26)	0.45(11.0, 0.15)	0.52(0.16, 0.06)
16	0.13(7.8)	0.20(0.54)	0.14(9.5, **0.08**)	0.24(0.71, 0.20)	0.37(7.4)	0.46(0.24)	0.44(11.0, 0.19)	0.52(0.18, 0.13)
17	0.25(7.8)	0.29(0.16)	0.24(9.5, **-0.04**)	0.31(0.29, 0.07)	0.21(7.4)	0.31(0.48)	0.31(11.0, 0.48)	0.37(0.19, 0.19)
18	0.08(7.8)	0.11(0.38)	0.18(9.5, 1.25)	0.15(-0.17, 0.36)	0.34(7.4)	0.37(0.09)	0.36(11.0, 0.06)	0.39(0.08, 0.05)
19	0.33(7.8)	0.39(0.18)	0.41(9.5, 0.24)	0.42(0.02, 0.08)	0.33(7.4)	0.30(-0.09)	0.33(11.0, **0.00**)	0.34(0.03, 0.13)
20	0.21(7.8)	0.35(0.67)	0.24(9.5, 0.14)	0.39(0.63, 0.11)	0.41(7.4)	0.46(0.12)	0.43(11.0, 0.05)	0.50(0.16, 0.09)
21	0.30(7.8)	0.46(0.53)	0.42(9.5, 0.40)	0.52(0.24, 0.13)	0.37(7.4)	0.42(0.14)	0.44(11.0, 0.19)	0.45(0.02, 0.07)
22	0.23(7.8)	0.21(-0.09)	0.31(9.5, 0.35)	0.26(-0.16, 0.24)	0.19(7.4)	0.27(0.42)	0.25(11.0, 0.32)	0.31(0.24, 0.15)
23	0.20(7.8)	0.23(0.15)	0.19(9.5, **-0.05**)	0.27(0.42, 0.17)	0.21(7.4)	0.29(0.38)	0.26(11.0, 0.24)	0.33(0.27, 0.14)
24	0.12(7.8)	0.20(0.67)	0.16(9.5, 0.33)	0.23(0.44, 0.15)	0.40(7.4)	0.39(-0.03)	0.44(11.0, 0.10)	0.43(-0.02, 0.10)
25	0.23(7.8)	0.38(0.65)	0.20(9.5, -0.13)	0.39(0.95, 0.03)	0.23(7.4)	0.26(0.13)	0.23(11.0, **0.00**)	0.28(0.22, 0.08)
Mean	0.23(7.8)	0.31(0.35)	0.26(9.5, 0.13)	0.35(0.35, 0.12)	0.30(7.3)	0.35(0.17)	0.33(11.1, 0.12)	0.38(0.15, 0.09)

[a] In parentheses is the number of QTL identified by QP based on the SE model; [b] In parentheses is the gain in prediction accuracy with GWP over QP based on the SE model; [c] The first value in parentheses is the number of QTL identified by QP based on the ME model; and the second one the gain

with ME over SE for QP; [d] The first value in parentheses is the gain in accuracy with GWP over QP based on the ME model; the second one is the gain in accuracy with ME over SE using GWP; and the third one is the gain in accuracy with GWP over PP. Bold in parentheses indicates the number is not significant at $\alpha = 0.05$.
