# Supplementary material for: Accuracy of Across-Environment Genome-Wide Prediction in Maize Nested Association Mapping Populations
Source: G3 (Bethesda). 2013 Feb 1;3(2):263–72. doi: 10.1534/g3.112.005066 (PMC3564986; doi:10.1534/g3.112.005066)
Supplement: Supporting Information [file supp_3.2.263_TableS22.pdf]

Table S22 Accuracy of AP prediction for environment E4 with QP and GWP in CV2

	LL				LW			
	SE		ME		SE		ME	
PopId	QP[a]	GWP[b]	QP[c]	GWP[d]	QP[a]	GWP[b]	QP[c]	GWP[d]
1	0.22(7.4)	0.30(0.36)	0.24(9.5, **0.09**)	0.36(0.50, 0.20)	0.30(11.0)	0.34(0.13)	0.34(11.0, 0.13)	0.37(0.09, 0.09)
2	0.18(7.4)	0.25(0.39)	0.21(9.5, 0.17)	0.28(0.33, 0.12)	0.40(11.0)	0.44(0.10)	0.41(11.0, 0.02)	0.47(0.15, 0.07)
3	0.23(7.4)	0.26(0.13)	0.23(9.5, **0.00**)	0.30(0.30, 0.15)	0.37(11.0)	0.40(0.08)	0.37(11.0, **0.00**)	0.44(0.19, 0.10)
4	0.33(7.4)	0.45(0.36)	0.37(9.5, 0.12)	0.50(0.35, 0.11)	0.29(11.0)	0.31(0.07)	0.30(11.0, **0.03**)	0.34(0.13, 0.10)
5	0.23(7.4)	0.39(0.70)	0.26(9.5, 0.13)	0.42(0.62, 0.08)	0.28(11.0)	0.35(0.25)	0.32(11.0, 0.14)	0.40(0.25, 0.14)
6	0.38(7.4)	0.46(0.21)	0.42(9.5, 0.11)	0.52(0.24, 0.13)	0.31(11.0)	0.36(0.16)	0.31(11.0, **0.00**)	0.37(0.19, 0.03)
7	0.22(7.4)	0.36(0.64)	0.24(9.5, **0.09**)	0.39(0.63, 0.08)	0.47(11.0)	0.53(0.13)	0.50(11.0, 0.06)	0.57(0.14, 0.08)
8	0.24(7.4)	0.35(0.46)	0.32(9.5, 0.33)	0.40(0.25, 0.14)	0.32(11.0)	0.40(0.25)	0.36(11.0, 0.12)	0.44(0.22, 0.10)
9	0.27(7.4)	0.34(0.26)	0.30(9.5, 0.11)	0.37(0.23, 0.09)	0.38(11.0)	0.42(0.11)	0.41(11.0, 0.08)	0.46(0.12, 0.10)
10	0.35(7.4)	0.46(0.31)	0.34(9.5, **-0.03**)	0.51(0.50, 0.11)	0.46(11.0)	0.55(0.20)	0.46(11.0, **0.00**)	0.58(0.26, 0.05)
11	0.14(7.4)	0.27(0.93)	0.20(9.5, 0.43)	0.32(0.60, 0.19)	0.35(11.0)	0.37(0.06)	0.34(11.0, **-0.03**)	0.38(0.12, 0.03)
12	0.31(7.4)	0.34(0.10)	0.40(9.5, 0.29)	0.41(0.02, 0.21)	0.56(11.0)	0.58(0.04)	0.56(11.0, **0.00**)	0.60(0.07, 0.03)
13	0.18(7.4)	0.16(-0.11)	0.24(9.5, 0.33)	0.21(-0.13, 0.31)	0.29(11.0)	0.32(0.10)	0.37(11.0, 0.28)	0.38(0.03, 0.19)
14	0.25(7.4)	0.35(0.40)	0.28(9.5, 0.12)	0.38(0.36, 0.09)	0.33(11.0)	0.35(0.06)	0.36(11.0, 0.09)	0.38(0.06, 0.09)
15	0.17(7.4)	0.25(0.47)	0.21(9.5, 0.24)	0.30(0.43, 0.20)	0.56(11.0)	0.61(0.09)	0.57(11.0, 0.02)	0.63(0.11, 0.03)
16	0.18(7.4)	0.25(0.39)	0.20(9.5, 0.11)	0.30(0.50, 0.20)	0.49(11.0)	0.58(0.18)	0.53(11.0, 0.08)	0.60(0.13, 0.03)
17	0.14(7.4)	0.25(0.79)	0.15(9.5, **0.07**)	0.27(0.80, 0.08)	0.54(11.0)	0.55(0.02)	0.54(11.0, **0.00**)	0.57(0.06, 0.04)
18	0.12(7.4)	0.16(0.33)	0.19(9.5, 0.58)	0.18(-0.05, 0.13)	0.39(11.0)	0.41(0.05)	0.39(11.0, **0.00**)	0.42(0.08, 0.02)
19	0.22(7.4)	0.21(-0.05)	0.27(9.5, 0.23)	0.25(-0.07, 0.19)	0.40(11.0)	0.38(-0.05)	0.44(11.0, 0.10)	0.41(-0.07, 0.08)
20	0.27(7.4)	0.42(0.56)	0.32(9.5, 0.19)	0.46(0.44, 0.10)	0.47(11.0)	0.52(0.11)	0.47(11.0, 0.00)	0.56(0.19, 0.08)
21	0.43(7.4)	0.52(0.21)	0.48(9.5, 0.12)	0.55(0.15, 0.06)	0.35(11.0)	0.41(0.17)	0.40(11.0, 0.14)	0.45(0.13, 0.10)
22	0.30(7.4)	0.29(-0.03)	0.31(9.5, **0.03**)	0.31(**0.00**, 0.07)	0.34(11.0)	0.42(0.24)	0.33(11.0, -0.03)	0.45(0.36, 0.07)
23	0.06(7.4)	0.14(1.33)	0.05(9.5, -0.17)	0.17(2.40, 0.21)	0.31(11.0)	0.32(0.03)	0.30(11.0, -0.03)	0.35(0.17, 0.09)
24	0.18(7.4)	0.28(0.56)	0.21(9.5, 0.17)	0.29(0.38, 0.04)	0.46(11.0)	0.50(0.09)	0.49(11.0, 0.07)	0.52(0.06, 0.04)
25	0.15(7.4)	0.28(0.87)	0.17(9.5, 0.13)	0.33(0.94, 0.18)	0.36(11.0)	0.42(0.17)	0.34(11.0, -0.06)	0.44(0.29, 0.05)
Mean	0.23(7.4)	0.31(0.35)	0.26(9.5, 0.15)	0.35(0.35, 0.12)	0.39(11.0)	0.43(0.10)	0.41(11.1, 0.04)	0.46(0.12, 0.07)

[a] In parentheses is the number of QTL identified by QP based on the SE model; [b] In parentheses is the gain in prediction accuracy with GWP over QP based on the SE model; [c] The first value in parentheses is the number of QTL identified by QP based on the ME model; and the second one the gain

with ME over SE for QP; [d] The first value in parentheses is the gain in accuracy with GWP over QP based on the ME model; the second one is the gain in accuracy with ME over SE using GWP; and the third one is the gain in accuracy with GWP over PP. Bold in parentheses indicates the number is not significant at $\alpha = 0.05$.
