# Supplementary material for: Accuracy of Across-Environment Genome-Wide Prediction in Maize Nested Association Mapping Populations
Source: G3 (Bethesda). 2013 Feb 1;3(2):263–72. doi: 10.1534/g3.112.005066 (PMC3564986; doi:10.1534/g3.112.005066)
Supplement: Supporting Information [file supp_3.2.263_TableS23.pdf]

Table S23 Accuracy of WP prediction for environment E1 with four ME GWP models in CV1

	LL				LW			
PopId	SG-SR	SG-UR[a]	UG-SR[b]	UG-UR[c]	SG-SR	SG-UR[a]	UG-SR[b]	UG-UR[c]
1	0.33	0.27(-0.17)	0.42(0.28)	0.42(**0.00**)	0.29	0.25(-0.16)	0.39(0.35)	0.39(**0.00**)
2	0.17	0.10(-0.42)	0.24(0.44)	0.24(**0.00**)	0.40	0.36(-0.10)	0.45(0.12)	0.45(**0.00**)
3	0.28	0.25(-0.08)	0.27(-**0.01**)	0.28(0.03)	0.55	0.52(-0.05)	0.59(0.06)	0.59(**0.00**)
4	0.48	0.44(-0.07)	0.50(0.05)	0.50(**0.00**)	0.49	0.47(-0.04)	0.53(0.08)	0.53(**0.00**)
5	0.32	0.24(-0.24)	0.39(0.22)	0.39(**0.00**)	0.20	0.12(-0.41)	0.30(0.49)	0.29(-0.02)
6	0.50	0.44(-0.11)	0.53(0.07)	0.53(**0.00**)	0.32	0.30(-0.07)	0.38(0.18)	0.38(**0.00**)
7	0.52	0.47(-0.09)	0.58(0.12)	0.57(-**0.01**)	0.48	0.44(-0.08)	0.52(0.09)	0.52(**0.00**)
8	0.31	0.24(-0.24)	0.37(0.19)	0.35(-0.05)	0.31	0.25(-0.19)	0.36(0.19)	0.38(0.04)
9	0.34	0.28(-0.19)	0.39(0.15)	0.38(-0.03)	0.17	0.12(-0.26)	0.35(1.11)	0.36(0.01)
10	0.47	0.42(-0.12)	0.50(0.05)	0.49(-**0.01**)	0.39	0.33(-0.15)	0.52(0.33)	0.52(**0.00**)
11	0.37	0.33(-0.12)	0.45(0.20)	0.45(**0.00**)	0.39	0.39(-0.02)	0.42(0.07)	0.42(**0.00**)
12	0.49	0.43(-0.11)	0.54(0.12)	0.54(**0.00**)	0.54	0.52(-0.05)	0.57(0.05)	0.57(**0.00**)
13	0.45	0.41(-0.09)	0.44(-0.03)	0.47(0.07)	0.50	0.46(-0.08)	0.52(0.05)	0.52(**0.00**)
14	0.38	0.31(-0.19)	0.43(0.11)	0.42(**0.00**)	0.47	0.43(-0.09)	0.50(0.07)	0.50(**0.00**)
15	0.28	0.20(-0.28)	0.32(0.12)	0.31(-0.03)	0.30	0.27(-0.11)	0.34(0.14)	0.34(**0.00**)
16	0.33	0.28(-0.15)	0.36(0.07)	0.36(**0.00**)	0.46	0.44(-0.04)	0.54(0.17)	0.54(**0.00**)
17	0.27	0.24(-0.10)	0.29(0.08)	0.29(**0.00**)	0.55	0.52(-0.05)	0.59(0.07)	0.59(**0.00**)
18	0.28	0.25(-0.12)	0.29(0.03)	0.28(-0.03)	0.39	0.38(-0.04)	0.43(0.10)	0.43(**0.00**)
19	0.37	0.31(-0.16)	0.41(0.12)	0.40(-0.04)	0.41	0.34(-0.16)	0.44(0.07)	0.44(**0.00**)
20	0.47	0.40(-0.15)	0.51(0.07)	0.48(-0.05)	0.47	0.44(-0.07)	0.50(0.06)	0.51(0.02)
21	0.49	0.44(-0.10)	0.50(0.02)	0.51(**0.01**)	0.47	0.46(-0.02)	0.47(**0.00**)	0.48(0.01)
22	0.35	0.30(-0.15)	0.42(0.19)	0.41(-**0.01**)	0.32	0.28(-0.11)	0.41(0.27)	0.42(0.02)
23	0.42	0.37(-0.12)	0.47(0.12)	0.46(-0.03)	0.29	0.22(-0.24)	0.43(0.47)	0.43(**0.00**)
24	0.45	0.41(-0.08)	0.45(**0.01**)	0.44(-0.02)	0.50	0.47(-0.06)	0.53(0.06)	0.53(**0.00**)
25	0.50	0.45(-0.10)	0.53(0.05)	0.51(-0.03)	0.45	0.42(-0.08)	0.48(0.06)	0.48(**0.00**)
Mean	0.38	0.33(-0.14)	0.42(0.10)	0.42(0.00)	0.40	0.37(-0.09)	0.46(0.14)	0.46(0.00)

[a] In parentheses is the gain in prediction accuracy with SG-UR over SG-SR; [b] In parentheses is the gain in prediction accuracy with UG-SR over SG-SR;

[c] In parentheses is the gain in prediction accuracy with UG-UR over UG-SR; Bold in parentheses indicates the number is not significant at α = 0.05.
