# Supplementary material for: Accuracy of Across-Environment Genome-Wide Prediction in Maize Nested Association Mapping Populations
Source: G3 (Bethesda). 2013 Feb 1;3(2):263–72. doi: 10.1534/g3.112.005066 (PMC3564986; doi:10.1534/g3.112.005066)
Supplement: Supporting Information [file supp_3.2.263_TableS24.pdf]

Table S24 Accuracy of WP prediction for environment E2 with four ME GWP models in CV1

	**LL**				**LW**			
**PopId**	**SG-SR**	**SG-UR**[a]	**UG-SR**[b]	**UG-UR**[c]	**SG-SR**	**SG-UR**[a]	**UG-SR**[b]	**UG-UR**[c]
1	0.67	0.67(**0.00**)	0.67(**0.00**)	0.67(**0.00**)	0.46	0.43(-0.06)	0.50(0.09)	0.50(**0.00**)
2	0.40	0.36(-0.10)	0.42(0.04)	0.40(-0.03)	0.49	0.46(-0.07)	0.53(0.07)	0.53(**0.00**)
3	0.17	0.13(-0.21)	0.19(0.14)	0.18(-0.06)	0.59	0.57(-0.04)	0.61(0.03)	0.61(**0.00**)
4	0.56	0.54(-0.04)	0.57(0.03)	0.57(**0.00**)	0.59	0.57(-0.03)	0.62(0.04)	0.62(**0.00**)
5	0.50	0.47(-0.07)	0.51(0.02)	0.51(**0.00**)	0.45	0.39(-0.12)	0.48(0.07)	0.46(-0.03)
6	0.49	0.43(-0.11)	0.51(0.06)	0.50(-0.03)	0.33	0.32(-0.04)	0.34(0.03)	0.34(**0.00**)
7	0.42	0.36(-0.14)	0.52(0.23)	0.51(-0.03)	0.58	0.54(-0.06)	0.61(0.06)	0.62(0.01)
8	0.35	0.27(-0.23)	0.40(0.14)	0.37(-0.08)	0.35	0.29(-0.17)	0.41(0.17)	0.42(0.03)
9	0.30	0.25(-0.19)	0.34(0.12)	0.32(-0.07)	0.44	0.41(-0.06)	0.44(**0.00**)	0.43(-0.02)
10	0.50	0.43(-0.12)	0.52(0.05)	0.51(-0.03)	0.36	0.26(-0.26)	0.39(0.08)	0.36(-0.07)
11	0.52	0.50(-0.05)	0.55(0.04)	0.55(**0.00**)	0.40	0.39(-0.03)	0.43(0.07)	0.43(**0.00**)
12	0.56	0.51(-0.10)	0.60(0.08)	0.59(-0.02)	0.58	0.56(-0.03)	0.61(0.05)	0.61(**0.00**)
13	0.40	0.32(-0.20)	0.44(0.08)	0.45(0.03)	0.39	0.32(-0.19)	0.47(0.20)	0.47(**0.00**)
14	0.42	0.38(-0.11)	0.44(0.05)	0.42(-0.04)	0.27	0.19(-0.29)	0.37(0.40)	0.36(-0.04)
15	0.46	0.43(-0.08)	0.45(-0.03)	0.46(0.02)	0.55	0.53(-0.04)	0.58(0.05)	0.58(**0.00**)
16	0.31	0.26(-0.16)	0.33(0.06)	0.32(-0.02)	0.56	0.55(-0.02)	0.58(0.03)	0.59(0.01)
17	0.13	0.08(-0.40)	0.20(0.52)	0.19(**-0.02**)	0.61	0.59(-0.03)	0.64(0.04)	0.64(**0.00**)
18	0.29	0.23(-0.20)	0.32(0.12)	0.31(-0.03)	0.30	0.27(-0.08)	0.42(0.42)	0.43(**0.01**)
19	0.28	0.20(-0.29)	0.34(0.20)	0.30(-0.12)	0.40	0.35(-0.11)	0.45(0.14)	0.46(0.02)
20	0.52	0.46(-0.12)	0.55(0.05)	0.54(-0.02)	0.53	0.50(-0.07)	0.56(0.04)	0.56(**0.00**)
21	0.58	0.54(-0.08)	0.61(0.04)	0.61(**0.00**)	0.35	0.32(-0.09)	0.41(0.19)	0.40(-0.02)
22	0.49	0.47(-0.05)	0.50(0.02)	0.50(**0.00**)	0.58	0.56(-0.03)	0.59(0.02)	0.60(0.01)
23	0.43	0.39(-0.10)	0.47(0.09)	0.46(**-0.01**)	0.36	0.32(-0.12)	0.40(0.11)	0.41(0.01)
24	0.16	0.08(-0.53)	0.27(0.63)	0.25(-0.06)	0.42	0.39(-0.08)	0.48(0.14)	0.48(**0.00**)
25	0.38	0.31(-0.20)	0.45(0.17)	0.42(-0.07)	0.47	0.42(-0.12)	0.48(0.01)	0.45(-0.05)
Mean	0.41	0.36(-0.12)	0.45(0.08)	0.44(-0.02)	0.46	0.42(-0.08)	0.50(0.09)	0.49(-0.01)

[a] In parentheses is the gain in prediction accuracy with SG-UR over SG-SR; [b] In parentheses is the gain in prediction accuracy with UG-SR over SG-SR;

[c] In parentheses is the gain in prediction accuracy with UG-UR over UG-SR; Bold in parentheses indicates the number is not significant at α = 0.05.
