# Supplementary material for: Accuracy of Across-Environment Genome-Wide Prediction in Maize Nested Association Mapping Populations
Source: G3 (Bethesda). 2013 Feb 1;3(2):263–72. doi: 10.1534/g3.112.005066 (PMC3564986; doi:10.1534/g3.112.005066)
Supplement: Supporting Information [file supp_3.2.263_TableS25.pdf]

Table S25 Accuracy of WP prediction for environment E3 with four ME GWP models in CV1

	LL				LW			
PopId	SG-SR	SG-UR[a]	UG-SR[b]	UG-UR[c]	SG-SR	SG-UR[a]	UG-SR[b]	UG-UR[c]
1	0.51	0.47(-0.06)	0.55(0.09)	0.55(**0.00**)	0.36	0.33(-0.08)	0.41(0.13)	0.41(**0.00**)
2	0.35	0.29(-0.17)	0.38(0.10)	0.36(-0.06)	0.52	0.49(-0.05)	0.55(0.06)	0.55(**0.00**)
3	0.24	0.20(-0.17)	0.26(0.10)	0.26(**0.00**)	0.27	0.23(-0.18)	0.37(0.35)	0.37(**0.00**)
4	0.50	0.45(-0.10)	0.55(0.10)	0.54(-0.02)	0.18	0.12(-0.32)	0.28(0.56)	0.29(0.00)
5	0.44	0.39(-0.11)	0.46(0.05)	0.46(**0.00**)	0.30	0.22(-0.26)	0.39(0.29)	0.39(**0.00**)
6	0.47	0.40(-0.15)	0.54(0.14)	0.54(**0.00**)	0.31	0.29(-0.07)	0.35(0.15)	0.35(**0.00**)
7	0.49	0.44(-0.10)	0.55(0.13)	0.55(**0.00**)	0.42	0.38(-0.10)	0.49(0.17)	0.50(0.01)
8	0.32	0.24(-0.26)	0.38(0.18)	0.35(-0.07)	0.48	0.44(-0.07)	0.49(0.03)	0.49(**0.00**)
9	0.42	0.36(-0.13)	0.45(0.07)	0.45(**0.00**)	0.45	0.43(-0.05)	0.48(0.07)	0.48(**0.00**)
10	0.42	0.34(-0.20)	0.48(0.13)	0.48(**0.00**)	0.56	0.52(-0.08)	0.58(0.04)	0.56(-0.04)
11	0.54	0.51(-0.05)	0.56(0.04)	0.56(**0.00**)	0.36	0.35(-0.03)	0.41(0.14)	0.41(**0.00**)
12	0.43	0.35(-0.17)	0.49(0.15)	0.50(0.02)	0.28	0.23(-0.17)	0.37(0.35)	0.38(0.00)
13	0.41	0.31(-0.25)	0.45(0.09)	0.43(-0.05)	0.26	0.16(-0.37)	0.35(0.38)	0.35(**0.00**)
14	0.31	0.23(-0.24)	0.35(0.15)	0.35(**0.00**)	0.32	0.27(-0.16)	0.42(0.28)	0.42(**0.00**)
15	0.25	0.15(-0.39)	0.30(0.21)	0.30(**0.00**)	0.40	0.37(-0.07)	0.47(0.19)	0.48(0.01)
16	0.32	0.28(-0.15)	0.34(0.05)	0.33(-0.02)	0.36	0.34(-0.06)	0.45(0.26)	0.45(**0.00**)
17	0.32	0.28(-0.13)	0.37(0.14)	0.37(**0.00**)	0.38	0.33(-0.13)	0.45(0.17)	0.45(**0.00**)
18	0.17	0.12(-0.28)	0.20(0.16)	0.21(**0.02**)	0.33	0.31(-0.06)	0.39(0.19)	0.39(**0.00**)
19	0.46	0.41(-0.11)	0.48(0.06)	0.47(-0.03)	0.44	0.40(-0.09)	0.46(0.05)	0.46(**0.00**)
20	0.34	0.25(-0.27)	0.40(0.19)	0.39(-0.02)	0.43	0.39(-0.09)	0.47(0.08)	0.46(-0.01)
21	0.47	0.41(-0.14)	0.53(0.12)	0.54(0.02)	0.38	0.37(-0.04)	0.42(0.11)	0.42(**0.00**)
22	0.38	0.34(-0.10)	0.41(0.08)	0.40(-0.01)	0.35	0.31(-0.09)	0.42(0.20)	0.43(0.02)
23	0.32	0.26(-0.19)	0.41(0.30)	0.42(0.01)	0.38	0.33(-0.13)	0.43(0.13)	0.42(-0.02)
24	0.26	0.19(-0.25)	0.30(0.16)	0.27(-0.09)	0.41	0.37(-0.10)	0.47(0.13)	0.48(0.02)
25	0.37	0.28(-0.25)	0.44(0.21)	0.40(-0.09)	0.29	0.24(-0.15)	0.33(0.15)	0.34(0.02)
Mean	0.38	0.32(-0.16)	0.43(0.12)	0.42(-0.02)	0.37	0.33(-0.11)	0.43(0.16)	0.43(0.00)

[a] In parentheses is the gain in prediction accuracy with SG-UR over SG-SR; [b] In parentheses is the gain in prediction accuracy with UG-SR over SG-SR;

[c] In parentheses is the gain in prediction accuracy with UG-UR over UG-SR; Bold in parentheses indicates the number is not significant at $\alpha = 0.05$.
