# Supplementary material for: Accuracy of Across-Environment Genome-Wide Prediction in Maize Nested Association Mapping Populations
Source: G3 (Bethesda). 2013 Feb 1;3(2):263–72. doi: 10.1534/g3.112.005066 (PMC3564986; doi:10.1534/g3.112.005066)
Supplement: Supporting Information [file supp_3.2.263_TableS26.pdf]

Table S26 Accuracy of WP prediction for environment E4 with four ME GWP models in CV1

	LL				LW			
PopId	SG-SR	SG-UR[a]	UG-SR[b]	UG-UR[c]	SG-SR	SG-UR[a]	UG-SR[b]	UG-UR[c]
1	0.47	0.44(-0.06)	0.51(0.09)	0.51(**0.00**)	0.50	0.48(-0.05)	0.54(0.08)	0.54(**0.00**)
2	0.30	0.25(-0.17)	0.34(0.13)	0.32(-0.05)	0.48	0.43(-0.10)	0.51(0.05)	0.51(**0.00**)
3	0.26	0.24(-0.10)	0.27(0.04)	0.27(**0.00**)	0.42	0.39(-0.07)	0.52(0.23)	0.52(**0.00**)
4	0.52	0.48(-0.08)	0.56(0.08)	0.56(**0.00**)	0.51	0.49(-0.05)	0.54(0.06)	0.54(**0.00**)
5	0.35	0.30(-0.15)	0.39(0.12)	0.39(**0.00**)	0.45	0.40(-0.11)	0.48(0.08)	0.47(-0.03)
6	0.45	0.38(-0.15)	0.52(0.16)	0.51(-0.02)	0.30	0.28(-0.08)	0.36(0.19)	0.36(**0.00**)
7	0.60	0.56(-0.06)	0.63(0.05)	0.62(-0.01)	0.62	0.60(-0.03)	0.64(0.04)	0.65(0.00)
8	0.40	0.32(-0.19)	0.44(0.12)	0.43(-0.04)	0.40	0.34(-0.14)	0.45(0.12)	0.45(**0.00**)
9	0.34	0.27(-0.19)	0.38(0.12)	0.37(-0.03)	0.47	0.45(-0.05)	0.48(0.01)	0.47(-0.02)
10	0.41	0.34(-0.18)	0.45(0.09)	0.43(-0.03)	0.63	0.59(-0.05)	0.65(0.04)	0.64(-0.01)
11	0.29	0.23(-0.18)	0.38(0.33)	0.38(**0.00**)	0.44	0.43(-0.03)	0.49(0.11)	0.49(**0.00**)
12	0.50	0.44(-0.12)	0.56(0.11)	0.54(-0.03)	0.57	0.54(-0.04)	0.59(0.05)	0.60(0.01)
13	0.24	0.08(-0.68)	0.30(0.28)	0.34(0.12)	0.42	0.37(-0.12)	0.49(0.17)	0.49(**0.00**)
14	0.34	0.27(-0.21)	0.39(0.13)	0.37(-0.05)	0.45	0.40(-0.11)	0.49(0.08)	0.49(**0.00**)
15	0.36	0.29(-0.19)	0.38(0.06)	0.37(-0.02)	0.53	0.51(-0.05)	0.55(0.04)	0.56(0.01)
16	0.20	0.13(-0.38)	0.28(0.39)	0.28(**0.00**)	0.53	0.51(-0.03)	0.57(0.08)	0.57(**0.00**)
17	0.34	0.30(-0.10)	0.37(0.10)	0.36(-0.03)	0.56	0.54(-0.05)	0.60(0.07)	0.61(0.01)
18	0.26	0.22(-0.17)	0.29(0.10)	0.28(-0.03)	0.31	0.29(-0.06)	0.38(0.22)	0.38(**0.00**)
19	0.26	0.18(-0.28)	0.32(0.26)	0.31(-0.02)	0.51	0.46(-0.09)	0.54(0.07)	0.55(0.01)
20	0.46	0.39(-0.15)	0.49(0.08)	0.48(-0.03)	0.52	0.49(-0.05)	0.54(0.03)	0.55(0.02)
21	0.61	0.56(-0.09)	0.64(0.04)	0.64(**0.00**)	0.36	0.34(-0.06)	0.41(0.13)	0.41(**0.00**)
22	0.47	0.44(-0.06)	0.48(0.02)	0.48(**0.00**)	0.55	0.52(-0.04)	0.59(0.07)	0.59(**0.00**)
23	0.39	0.33(-0.14)	0.45(0.17)	0.45(**0.00**)	0.48	0.44(-0.08)	0.51(0.06)	0.51(**0.00**)
24	0.26	0.18(-0.30)	0.32(0.23)	0.29(-0.07)	0.47	0.42(-0.10)	0.51(0.09)	0.51(**0.00**)
25	0.26	0.17(-0.33)	0.34(0.35)	0.33(-0.06)	0.48	0.44(-0.09)	0.49(0.01)	0.47(-0.04)
Mean	0.37	0.31(-0.16)	0.42(0.13)	0.41(-0.02)	0.48	0.45(-0.07)	0.52(0.08)	0.52(0.00)

[a] In parentheses is the gain in prediction accuracy with SG-UR over SG-SR; [b] In parentheses is the gain in prediction accuracy with UG-SR over SG-SR;

[c] In parentheses is the gain in prediction accuracy with UG-UR over UG-SR; Bold in parentheses indicates the number is not significant at α = 0.05.
