# Supplementary material for: Accuracy of Across-Environment Genome-Wide Prediction in Maize Nested Association Mapping Populations
Source: G3 (Bethesda). 2013 Feb 1;3(2):263–72. doi: 10.1534/g3.112.005066 (PMC3564986; doi:10.1534/g3.112.005066)
Supplement: Supporting Information [file supp_3.2.263_TableS27.pdf]

Table S27 Accuracy of AP prediction for environment E1 with four ME GWP models in CV1

	LL				LW			
PopId	SG-SR	SG-UR[a]	UG-SR[b]	UG-UR[c]	SG-SR	SG-UR[a]	UG-SR[b]	UG-UR[c]
1	0.31	0.30(-0.04)	0.34(0.07)	0.33(-0.02)	0.25	0.24(-0.07)	0.27(0.08)	0.26(-0.05)
2	0.19	0.17(-0.11)	0.21(0.12)	0.20(-0.06)	0.42	0.42(**0.00**)	0.42(**0.00**)	0.42(**0.00**)
3	0.21	0.21(**0.00**)	0.19(-0.05)	0.20(0.05)	0.54	0.53(-0.02)	0.54(**0.00**)	0.55(0.01)
4	0.41	0.41(**0.00**)	0.39(-0.04)	0.41(0.04)	0.29	0.30(0.01)	0.28(-0.04)	0.28(**0.00**)
5	0.28	0.29(0.04)	0.25(-0.08)	0.26(0.04)	0.23	0.22(-0.03)	0.24(0.04)	0.24(**0.00**)
6	0.46	0.44(-0.03)	0.47(0.02)	0.47(**0.00**)	0.37	0.37(**0.00**)	0.39(0.05)	0.39(**0.00**)
7	0.24	0.23(-0.07)	0.27(0.12)	0.26(-0.02)	0.36	0.34(-0.06)	0.41(0.14)	0.39(-0.04)
8	0.47	0.45(-0.04)	0.47(**0.00**)	0.47(**0.00**)	0.39	0.38(-0.02)	0.39(**0.00**)	0.39(**0.00**)
9	0.36	0.35(-0.03)	0.38(0.05)	0.38(**0.00**)	0.16	0.16(**0.00**)	0.16(**0.00**)	0.17(0.01)
10	0.37	0.37(**0.00**)	0.38(0.02)	0.38(**0.00**)	0.46	0.44(-0.04)	0.49(0.07)	0.48(-0.02)
11	0.07	0.05(-0.26)	0.11(0.51)	0.09(-0.16)	0.17	0.16(-0.04)	0.18(0.06)	0.18(**0.00**)
12	0.39	0.37(-0.03)	0.40(0.03)	0.40(**0.00**)	0.59	0.58(-0.02)	0.61(0.03)	0.61(**0.00**)
13	0.29	0.26(-0.11)	0.34(0.19)	0.32(-0.06)	0.42	0.42(**0.00**)	0.43(0.02)	0.43(**0.00**)
14	0.40	0.39(-0.02)	0.40(**0.00**)	0.40(**0.00**)	0.36	0.34(-0.04)	0.38(0.07)	0.38(**0.00**)
15	0.24	0.25(**0.01**)	0.23(-0.07)	0.24(0.04)	0.31	0.31(**0.00**)	0.32(**0.01**)	0.31(**-0.01**)
16	0.27	0.26(-0.04)	0.27(**0.00**)	0.28(**0.01**)	0.52	0.52(**0.00**)	0.52(**0.00**)	0.52(**0.00**)
17	0.29	0.28(-0.03)	0.29(**0.00**)	0.30(0.01)	0.43	0.42(-0.02)	0.45(0.05)	0.46(**0.01**)
18	0.14	0.13(-0.05)	0.14(**0.00**)	0.14(**0.00**)	0.30	0.28(-0.05)	0.33(0.10)	0.31(-0.04)
19	0.30	0.29(-0.04)	0.31(0.03)	0.30(-0.01)	0.22	0.21(-0.02)	0.23(0.06)	0.23(**0.00**)
20	0.42	0.41(-0.03)	0.43(0.02)	0.43(**0.00**)	0.43	0.43(**0.00**)	0.44(0.02)	0.44(**0.00**)
21	0.33	0.31(-0.08)	0.36(0.10)	0.35(-0.03)	0.32	0.30(-0.07)	0.35(0.10)	0.34(-0.04)
22	0.35	0.35(**0.00**)	0.34(-0.03)	0.34(**0.00**)	0.29	0.29(**0.00**)	0.30(0.02)	0.30(**0.00**)
23	0.21	0.22(0.03)	0.20(-0.05)	0.20(**0.00**)	0.15	0.13(-0.10)	0.19(0.28)	0.18(-0.04)
24	0.27	0.29(0.06)	0.23(-0.15)	0.25(0.07)	0.45	0.45(**0.00**)	0.45(**0.00**)	0.45(**0.00**)
25	0.42	0.42(**0.00**)	0.40(-0.05)	0.42(0.04)	0.47	0.45(-0.02)	0.47(**0.00**)	0.47(**0.00**)
Mean	0.31	0.30(-0.03)	0.31(0.02)	0.31(0.00)	0.36	0.35(-0.02)	0.37(0.04)	0.37(0.00)

[a] In parentheses is the gain in prediction accuracy with SG-UR over SG-SR; [b] In parentheses is the gain in prediction accuracy with UG-SR over SG-SR;

[c] In parentheses is the gain in prediction accuracy with UG-UR over UG-SR; Bold in parentheses indicates the number is not significant at α = 0.05.
