# Supplementary material for: Accuracy of Across-Environment Genome-Wide Prediction in Maize Nested Association Mapping Populations
Source: G3 (Bethesda). 2013 Feb 1;3(2):263–72. doi: 10.1534/g3.112.005066 (PMC3564986; doi:10.1534/g3.112.005066)
Supplement: Supporting Information [file supp_3.2.263_TableS28.pdf]

Table S28 Accuracy of AP prediction for environment E2 with four ME GWP models in CV1

	LL				LW			
PopId	SG-SR	SG-UR[a]	UG-SR[b]	UG-UR[c]	SG-SR	SG-UR[a]	UG-SR[b]	UG-UR[c]
1	0.39	0.39(**0.00**)	0.37(-0.03)	0.38(0.01)	0.21	0.20(-0.07)	0.24(0.14)	0.24(**0.00**)
2	0.28	0.26(-0.09)	0.31(0.09)	0.29(-0.05)	0.44	0.43(-0.02)	0.46(0.04)	0.45(-0.01)
3	0.29	0.30(0.02)	0.27(-0.08)	0.28(0.05)	0.54	0.53(-0.01)	0.54(**0.00**)	0.54(**0.00**)
4	0.38	0.37(-0.03)	0.39(0.01)	0.39(**0.00**)	0.34	0.33(-0.03)	0.36(0.06)	0.36(**0.00**)
5	0.33	0.33(**0.00**)	0.32(-0.03)	0.32(**0.00**)	0.47	0.45(-0.03)	0.48(0.04)	0.48(**0.00**)
6	0.47	0.47(**0.00**)	0.47(**0.00**)	0.47(**0.00**)	0.28	0.28(**0.00**)	0.30(0.05)	0.30(**0.00**)
7	0.20	0.20(**0.00**)	0.21(**0.01**)	0.21(**0.00**)	0.60	0.60(**0.00**)	0.58(-0.03)	0.59(0.02)
8	0.25	0.23(-0.06)	0.27(0.08)	0.26(-0.03)	0.34	0.34(**0.00**)	0.34(**0.00**)	0.35(0.01)
9	0.23	0.22(-0.01)	0.22(-0.03)	0.22(**0.00**)	0.34	0.34(**0.00**)	0.34(**0.00**)	0.34(**0.00**)
10	0.47	0.46(-0.02)	0.46(-0.01)	0.47(0.02)	0.41	0.40(-0.04)	0.45(0.09)	0.45(**0.00**)
11	0.29	0.27(-0.07)	0.31(0.06)	0.30(-0.02)	0.37	0.37(**0.00**)	0.38(0.02)	0.37(-0.01)
12	0.41	0.40(-0.02)	0.41(**0.00**)	0.41(**0.00**)	0.62	0.60(-0.02)	0.62(**0.00**)	0.63(0.00)
13	0.35	0.35(**0.00**)	0.35(**0.00**)	0.35(**0.00**)	0.41	0.40(**-0.01**)	0.40(-0.03)	0.40(**0.00**)
14	0.24	0.22(-0.10)	0.28(0.15)	0.26(-0.05)	0.37	0.36(-0.03)	0.39(0.06)	0.39(**0.00**)
15	0.30	0.30(**0.00**)	0.27(-0.07)	0.29(0.06)	0.53	0.53(**0.00**)	0.52(-0.03)	0.52(**0.00**)
16	0.42	0.41(-0.02)	0.41(-0.02)	0.42(0.02)	0.53	0.53(**0.00**)	0.54(0.02)	0.54(**0.00**)
17	0.11	0.09(-0.17)	0.13(0.17)	0.12(-0.06)	0.60	0.60(**0.00**)	0.57(-0.05)	0.59(0.03)
18	0.20	0.20(**0.00**)	0.18(-0.08)	0.19(0.06)	0.23	0.23(**0.00**)	0.22(-0.06)	0.22(**0.00**)
19	0.39	0.37(-0.05)	0.41(0.03)	0.40(-0.01)	0.40	0.40(**0.00**)	0.39(-0.02)	0.39(**0.00**)
20	0.41	0.40(-0.01)	0.41(**0.00**)	0.41(**0.00**)	0.44	0.43(-0.01)	0.43(-0.02)	0.43(**0.00**)
21	0.47	0.46(-0.02)	0.47(**0.00**)	0.47(**0.00**)	0.45	0.44(-0.02)	0.45(**0.00**)	0.45(**0.00**)
22	0.43	0.42(-0.03)	0.44(0.02)	0.43(-0.01)	0.40	0.40(**0.00**)	0.42(0.04)	0.42(**0.00**)
23	0.35	0.34(**-0.01**)	0.34(-0.01)	0.35(0.02)	0.38	0.37(-0.04)	0.40(0.04)	0.40(**0.00**)
24	0.21	0.20(-0.03)	0.21(**0.00**)	0.21(**0.00**)	0.45	0.46(0.02)	0.44(-0.03)	0.45(0.03)
25	0.24	0.23(-0.04)	0.25(0.05)	0.24(-0.03)	0.35	0.33(-0.04)	0.37(0.07)	0.37(**0.00**)
Mean	0.32	0.32(-0.02)	0.33(0.01)	0.33(0.00)	0.42	0.41(-0.01)	0.42(0.01)	0.43(0.00)

[a] In parentheses is the gain in prediction accuracy with SG-UR over SG-SR; [b] In parentheses is the gain in prediction accuracy with UG-SR over SG-SR;

[c] In parentheses is the gain in prediction accuracy with UG-UR over UG-SR; Bold in parentheses indicates the number is not significant at $\alpha = 0.05$.
