# Supplementary material for: Accuracy of Across-Environment Genome-Wide Prediction in Maize Nested Association Mapping Populations
Source: G3 (Bethesda). 2013 Feb 1;3(2):263–72. doi: 10.1534/g3.112.005066 (PMC3564986; doi:10.1534/g3.112.005066)
Supplement: Supporting Information [file supp_3.2.263_TableS29.pdf]

Table S29 Accuracy of AP prediction for environment E3 with four ME GWP models in CV1

	LL				LW			
PopId	SG-SR	SG-UR[a]	UG-SR[b]	UG-UR[c]	SG-SR	SG-UR[a]	UG-SR[b]	UG-UR[c]
1	0.37	0.37(**0.00**)	0.38(0.01)	0.37(-0.01)	0.24	0.23(-0.04)	0.25(0.04)	0.25(**0.00**)
2	0.27	0.25(-0.09)	0.31(0.12)	0.30(-0.03)	0.46	0.45(-0.02)	0.47(0.03)	0.47(**0.00**)
3	0.22	0.23(0.04)	0.20(-0.11)	0.21(0.07)	0.37	0.37(**0.00**)	0.34(-0.07)	0.36(0.04)
4	0.42	0.42(**0.00**)	0.40(-0.04)	0.41(0.03)	0.22	0.21(-0.04)	0.24(0.08)	0.24(**0.00**)
5	0.20	0.19(-0.05)	0.21(0.07)	0.21(**0.00**)	0.44	0.44(**0.00**)	0.42(-0.03)	0.43(**0.01**)
6	0.47	0.46(-0.01)	0.46(**-0.01**)	0.46(**0.00**)	0.26	0.26(**0.00**)	0.27(**0.01**)	0.26(-0.02)
7	0.20	0.19(-0.03)	0.19(-0.06)	0.20(0.04)	0.32	0.31(-0.04)	0.34(0.06)	0.33(-0.02)
8	0.29	0.27(-0.04)	0.30(0.06)	0.29(-0.03)	0.27	0.27(**0.00**)	0.28(0.03)	0.28(**0.00**)
9	0.35	0.35(**0.00**)	0.35(**0.00**)	0.36(0.02)	0.31	0.32(0.02)	0.29(-0.09)	0.30(0.04)
10	0.47	0.46(-0.01)	0.45(-0.05)	0.46(0.04)	0.56	0.56(**0.00**)	0.55(-0.01)	0.55(**0.00**)
11	0.22	0.21(-0.06)	0.23(0.05)	0.23(**0.00**)	0.26	0.25(-0.02)	0.27(0.06)	0.27(**0.00**)
12	0.32	0.31(-0.02)	0.30(-0.04)	0.31(**0.01**)	0.34	0.34(**0.00**)	0.34(**0.00**)	0.34(**0.00**)
13	0.36	0.34(-0.06)	0.41(0.12)	0.39(-0.05)	0.40	0.40(**0.00**)	0.37(-0.08)	0.38(0.03)
14	0.24	0.24(**0.00**)	0.24(**0.00**)	0.24(**0.00**)	0.26	0.25(-0.05)	0.29(0.13)	0.28(-0.04)
15	0.24	0.23(-0.06)	0.26(0.06)	0.25(-0.01)	0.49	0.48(-0.01)	0.50(0.01)	0.50(**0.00**)
16	0.16	0.14(-0.09)	0.17(0.09)	0.17(**0.00**)	0.46	0.44(-0.04)	0.50(0.11)	0.49(-0.03)
17	0.29	0.28(-0.04)	0.29(**0.00**)	0.29(**0.00**)	0.30	0.28(-0.06)	0.34(0.13)	0.33(-0.03)
18	0.11	0.09(-0.14)	0.14(0.29)	0.13(-0.07)	0.32	0.32(**0.00**)	0.33(0.02)	0.33(**0.00**)
19	0.36	0.35(-0.01)	0.35(-0.02)	0.35(**0.00**)	0.29	0.28(-0.01)	0.30(0.05)	0.30(**0.00**)
20	0.36	0.34(-0.06)	0.38(0.05)	0.38(**0.00**)	0.44	0.42(-0.03)	0.46(0.06)	0.46(**0.00**)
21	0.44	0.42(-0.05)	0.48(0.08)	0.46(-0.03)	0.40	0.39(-0.02)	0.41(0.03)	0.41(**0.00**)
22	0.20	0.19(-0.08)	0.22(0.08)	0.21(-0.02)	0.26	0.25(-0.02)	0.27(0.04)	0.26(-0.01)
23	0.22	0.22(**0.00**)	0.22(**0.00**)	0.23(0.01)	0.27	0.26(-0.03)	0.28(0.05)	0.28(**0.00**)
24	0.19	0.19(**0.00**)	0.18(-0.04)	0.19(0.04)	0.36	0.35(-0.03)	0.39(0.08)	0.38(-0.03)
25	0.39	0.40(0.01)	0.37(-0.06)	0.38(0.02)	0.24	0.23(-0.02)	0.23(-0.04)	0.24(0.03)
Mean	0.29	0.29(0.00)	0.30(0.01)	0.30(0.00)	0.34	0.33(-0.02)	0.35(0.03)	0.35(0.00)

[a] In parentheses is the gain in prediction accuracy with SG-UR over SG-SR; [b] In parentheses is the gain in prediction accuracy with UG-SR over SG-SR;

[c] In parentheses is the gain in prediction accuracy with UG-UR over UG-SR; Bold in parentheses indicates the number is not significant at α = 0.05.
