# Supplementary material for: Accuracy of Across-Environment Genome-Wide Prediction in Maize Nested Association Mapping Populations
Source: G3 (Bethesda). 2013 Feb 1;3(2):263–72. doi: 10.1534/g3.112.005066 (PMC3564986; doi:10.1534/g3.112.005066)
Supplement: Supporting Information [file supp_3.2.263_TableS30.pdf]

Table S30 Accuracy of AP prediction for environment E4 with four ME GWP models in CV1

	LL				LW			
PopId	SG-SR	SG-UR[a]	UG-SR[b]	UG-UR[c]	SG-SR	SG-UR[a]	UG-SR[b]	UG-UR[c]
1	0.26	0.26(**0.00**)	0.26(**0.00**)	0.27(0.03)	0.31	0.30(-0.03)	0.31(**0.00**)	0.31(**0.00**)
2	0.25	0.24(-0.03)	0.23(-0.09)	0.23(**0.00**)	0.43	0.42(-0.01)	0.43(**0.00**)	0.43(**0.00**)
3	0.24	0.23(-0.06)	0.24(**0.00**)	0.25(0.02)	0.39	0.39(**0.00**)	0.39(**0.00**)	0.40(0.03)
4	0.42	0.42(**0.00**)	0.43(0.02)	0.43(**0.00**)	0.29	0.29(**0.00**)	0.30(0.03)	0.29(-0.02)
5	0.38	0.38(**0.00**)	0.36(-0.05)	0.38(0.04)	0.33	0.32(-0.04)	0.36(0.08)	0.36(**0.00**)
6	0.46	0.44(-0.03)	0.48(0.06)	0.48(**0.00**)	0.33	0.32(-0.02)	0.33(**0.00**)	0.33(**0.00**)
7	0.32	0.31(-0.03)	0.30(-0.06)	0.31(0.04)	0.52	0.51(-0.01)	0.53(0.02)	0.53(**0.00**)
8	0.34	0.32(-0.05)	0.37(0.07)	0.35(-0.04)	0.39	0.39(**0.00**)	0.40(0.02)	0.40(**0.00**)
9	0.35	0.35(**0.00**)	0.35(**0.00**)	0.36(0.02)	0.40	0.39(-0.04)	0.42(0.04)	0.41(-0.01)
10	0.46	0.44(-0.04)	0.47(0.02)	0.48(0.02)	0.53	0.52(-0.02)	0.55(0.02)	0.54(-0.01)
11	0.27	0.27(**0.00**)	0.27(**0.00**)	0.27(**0.00**)	0.36	0.36(**0.00**)	0.35(-0.03)	0.36(0.02)
12	0.31	0.28(-0.10)	0.35(0.13)	0.34(-0.03)	0.57	0.57(**0.00**)	0.58(0.01)	0.58(**0.00**)
13	0.16	0.14(-0.11)	0.18(0.14)	0.16(-0.08)	0.32	0.29(-0.07)	0.35(0.11)	0.35(**0.00**)
14	0.35	0.35(**0.00**)	0.34(-0.04)	0.33(-0.01)	0.33	0.33(**0.00**)	0.33(**0.00**)	0.33(**0.00**)
15	0.20	0.19(-0.07)	0.20(**0.00**)	0.21(0.02)	0.60	0.59(-0.00)	0.60(**0.00**)	0.60(**0.00**)
16	0.23	0.21(-0.07)	0.25(0.11)	0.24(-0.04)	0.57	0.56(-0.02)	0.58(0.02)	0.58(**0.00**)
17	0.25	0.25(**0.00**)	0.24(-0.06)	0.24(**0.00**)	0.53	0.53(**0.00**)	0.52(-0.01)	0.53(0.01)
18	0.13	0.12(-0.03)	0.13(**0.00**)	0.13(**0.00**)	0.38	0.38(**0.00**)	0.37(-0.02)	0.38(0.02)
19	0.19	0.18(-0.01)	0.19(**0.00**)	0.19(**0.00**)	0.36	0.37(0.01)	0.36(**0.00**)	0.36(**0.00**)
20	0.41	0.39(-0.04)	0.42(0.02)	0.41(-0.01)	0.52	0.51(-0.01)	0.53(0.03)	0.52(-0.01)
21	0.52	0.52(**0.00**)	0.51(-0.03)	0.52(0.01)	0.41	0.39(-0.07)	0.45(0.09)	0.44(-0.04)
22	0.25	0.25(**0.00**)	0.23(-0.05)	0.24(0.03)	0.40	0.40(**0.00**)	0.40(**0.00**)	0.40(**0.00**)
23	0.13	0.13(**0.00**)	0.12(-0.06)	0.13(0.07)	0.30	0.29(-0.01)	0.30(**0.00**)	0.30(**0.00**)
24	0.29	0.30(0.04)	0.26(-0.10)	0.28(0.06)	0.50	0.49(-0.02)	0.50(**0.00**)	0.50(**0.00**)
25	0.29	0.27(-0.07)	0.31(0.06)	0.30(-0.01)	0.44	0.45(0.01)	0.42(-0.05)	0.43(0.03)
Mean	0.30	0.29(-0.03)	0.30(0.00)	0.30(0.00)	0.42	0.41(-0.02)	0.43(0.01)	0.43(0.00)

[a] In parentheses is the gain in prediction accuracy with SG-UR over SG-SR; [b] In parentheses is the gain in prediction accuracy with UG-SR over SG-SR;

[c] In parentheses is the gain in prediction accuracy with UG-UR over UG-SR; Bold in parentheses indicates the number is not significant at $\alpha = 0.05$.
