# Supplementary material for: Accuracy of Across-Environment Genome-Wide Prediction in Maize Nested Association Mapping Populations
Source: G3 (Bethesda). 2013 Feb 1;3(2):263–72. doi: 10.1534/g3.112.005066 (PMC3564986; doi:10.1534/g3.112.005066)
Supplement: Supporting Information [file supp_3.2.263_TableS31.pdf]

Table S31 Accuracy of WP prediction for environment E1 with four ME GWP models in CV2

	LL				LW			
PopId	SG-SR	SG-UR[a]	UG-SR[b]	UG-UR[c]	SG-SR	SG-UR[a]	UG-SR[b]	UG-UR[c]
1	0.33	0.30(-0.08)	0.45(0.40)	0.45(**0.00**)	0.31	0.29(-0.07)	0.49(0.59)	0.49(**0.00**)
2	0.18	0.16(-0.08)	0.41(1.35)	0.42(**0.01**)	0.40	0.38(-0.04)	0.55(0.39)	0.55(**0.00**)
3	0.26	0.25(**-0.02**)	0.36(0.42)	0.37(**0.01**)	0.56	0.55(-0.02)	0.68(0.21)	0.68(**0.00**)
4	0.49	0.47(-0.03)	0.60(0.24)	0.60(**0.00**)	0.50	0.49(-0.02)	0.61(0.22)	0.61(**0.00**)
5	0.33	0.31(-0.06)	0.53(0.60)	0.54(0.01)	0.21	0.19(-0.08)	0.47(1.26)	0.46(-0.01)
6	0.50	0.49(-0.03)	0.59(0.18)	0.59(**0.00**)	0.31	0.30(-0.04)	0.43(0.40)	0.43(**0.00**)
7	0.52	0.51(-0.02)	0.67(0.30)	0.67(**0.00**)	0.48	0.47(-0.02)	0.61(0.28)	0.61(**0.00**)
8	0.28	0.28(**0.00**)	0.52(0.81)	0.51(**-0.01**)	0.30	0.27(-0.08)	0.49(0.64)	0.49(**0.00**)
9	0.35	0.33(-0.06)	0.50(0.43)	0.50(**0.00**)	0.18	0.16(-0.09)	0.42(1.37)	0.42(**0.00**)
10	0.46	0.47(**0.01**)	0.64(0.38)	0.64(**0.00**)	0.37	0.35(-0.04)	0.62(0.69)	0.62(**0.00**)
11	0.40	0.38(-0.04)	0.56(0.39)	0.56(**0.00**)	0.40	0.39(-0.01)	0.48(0.21)	0.48(**0.00**)
12	0.50	0.49(-0.02)	0.65(0.31)	0.65(**0.00**)	0.54	0.54(**0.00**)	0.64(0.18)	0.64(**0.00**)
13	0.45	0.45(**0.00**)	0.65(0.43)	0.64(**-0.01**)	0.49	0.48(-0.02)	0.62(0.26)	0.61(-0.01)
14	0.36	0.35(-0.03)	0.56(0.56)	0.55(-0.01)	0.44	0.44(**0.00**)	0.62(0.40)	0.62(**0.00**)
15	0.28	0.25(-0.11)	0.48(0.72)	0.47(-0.02)	0.30	0.29(-0.03)	0.44(0.45)	0.43(**-0.01**)
16	0.34	0.32(-0.05)	0.48(0.43)	0.48(**0.00**)	0.45	0.44(-0.02)	0.56(0.23)	0.56(**0.00**)
17	0.27	0.26(-0.03)	0.40(0.45)	0.39(**-0.01**)	0.55	0.55(**0.00**)	0.68(0.24)	0.68(**0.00**)
18	0.27	0.27(**0.00**)	0.36(0.31)	0.36(**0.00**)	0.40	0.40(**0.00**)	0.47(0.16)	0.47(**0.00**)
19	0.37	0.35(-0.06)	0.53(0.45)	0.52(-0.03)	0.41	0.40(-0.02)	0.58(0.42)	0.58(**0.00**)
20	0.47	0.45(-0.05)	0.61(0.30)	0.61(**0.00**)	0.48	0.48(**0.00**)	0.59(0.24)	0.60(0.01)
21	0.48	0.47(**-0.01**)	0.60(0.26)	0.61(0.01)	0.47	0.47(**0.00**)	0.53(0.14)	0.54(0.01)
22	0.36	0.34(-0.06)	0.51(0.42)	0.51(**0.00**)	0.31	0.30(-0.05)	0.47(0.52)	0.47(**0.00**)
23	0.44	0.42(-0.04)	0.55(0.25)	0.54(-0.01)	0.30	0.30(**0.00**)	0.54(0.80)	0.54(**0.00**)
24	0.48	0.47(-0.03)	0.58(0.21)	0.57(-0.02)	0.50	0.49(-0.02)	0.64(0.29)	0.64(**0.00**)
25	0.50	0.48(-0.03)	0.61(0.24)	0.61(**0.00**)	0.47	0.46(-0.01)	0.61(0.30)	0.60(-0.01)
Mean	0.39	0.37(-0.04)	0.54(0.39)	0.53(-0.01)	0.41	0.39(-0.02)	0.55(0.36)	0.55(0.00)

[a] In parentheses is the gain in prediction accuracy with SG-UR over SG-SR; [b] In parentheses is the gain in prediction accuracy with UG-SR over SG-SR;

[c] In parentheses is the gain in prediction accuracy with UG-UR over UG-SR; Bold in parentheses indicates the number is not significant at α = 0.05.
