# Supplementary material for: Accuracy of Across-Environment Genome-Wide Prediction in Maize Nested Association Mapping Populations
Source: G3 (Bethesda). 2013 Feb 1;3(2):263–72. doi: 10.1534/g3.112.005066 (PMC3564986; doi:10.1534/g3.112.005066)
Supplement: Supporting Information [file supp_3.2.263_TableS32.pdf]

Table S32 Accuracy of WP prediction for environment E2 with four ME GWP models in CV2

	LL				LW			
PopId	SG-SR	SG-UR[a]	UG-SR[b]	UG-UR[c]	SG-SR	SG-UR[a]	UG-SR[b]	UG-UR[c]
1	0.67	0.67(**0.00**)	0.74(0.11)	0.75(**0.00**)	0.46	0.45(-0.02)	0.57(0.25)	0.57(**0.00**)
2	0.40	0.39(-0.03)	0.57(0.42)	0.56(-0.02)	0.48	0.47(-0.02)	0.64(0.33)	0.64(**0.00**)
3	0.20	0.19(-0.05)	0.31(0.56)	0.30(**-0.01**)	0.60	0.59(-0.01)	0.70(0.17)	0.70(**0.00**)
4	0.56	0.56(**0.00**)	0.68(0.21)	0.68(**0.00**)	0.61	0.60(-0.01)	0.70(0.16)	0.70(**0.00**)
5	0.50	0.50(**0.00**)	0.64(0.28)	0.64(**0.00**)	0.46	0.44(-0.03)	0.65(0.43)	0.64(-0.02)
6	0.47	0.46(-0.03)	0.58(0.23)	0.58(**0.00**)	0.31	0.31(**0.00**)	0.36(0.17)	0.37(0.01)
7	0.41	0.39(-0.04)	0.62(0.53)	0.62(**0.00**)	0.59	0.57(-0.02)	0.72(0.22)	0.72(**0.00**)
8	0.34	0.31(-0.08)	0.53(0.55)	0.51(-0.03)	0.36	0.34(-0.06)	0.53(0.47)	0.53(**0.00**)
9	0.33	0.32(-0.04)	0.46(0.38)	0.46(**0.00**)	0.47	0.46(-0.02)	0.54(0.15)	0.54(**0.00**)
10	0.51	0.50(-0.02)	0.66(0.30)	0.65(-0.01)	0.36	0.32(-0.09)	0.47(0.32)	0.46(-0.01)
11	0.52	0.51(-0.02)	0.62(0.19)	0.62(**0.00**)	0.41	0.40(-0.01)	0.54(0.34)	0.54(**0.00**)
12	0.55	0.53(-0.03)	0.69(0.25)	0.68(-0.01)	0.58	0.57(-0.01)	0.65(0.13)	0.65(**0.00**)
13	0.39	0.37(-0.05)	0.66(0.67)	0.65(-0.01)	0.42	0.40(-0.06)	0.62(0.46)	0.60(-0.02)
14	0.42	0.41(-0.04)	0.60(0.41)	0.58(-0.03)	0.25	0.23(-0.07)	0.46(0.82)	0.45(-0.02)
15	0.48	0.48(**0.00**)	0.64(0.34)	0.65(0.01)	0.55	0.54(-0.01)	0.68(0.24)	0.67(-0.01)
16	0.33	0.31(-0.05)	0.47(0.43)	0.46(-0.01)	0.55	0.55(**0.00**)	0.64(0.16)	0.64(**0.00**)
17	0.12	0.10(-0.14)	0.29(1.44)	0.29(**0.00**)	0.61	0.61(**0.00**)	0.73(0.18)	0.73(**0.00**)
18	0.27	0.24(-0.10)	0.41(0.54)	0.40(-0.02)	0.31	0.30(-0.04)	0.50(0.60)	0.50(**0.00**)
19	0.27	0.24(-0.11)	0.41(0.51)	0.40(-0.03)	0.41	0.39(-0.03)	0.57(0.39)	0.57(**0.00**)
20	0.52	0.51(-0.02)	0.66(0.27)	0.66(**0.00**)	0.54	0.53(-0.01)	0.69(0.29)	0.69(**0.00**)
21	0.58	0.56(-0.02)	0.71(0.23)	0.70(-0.01)	0.33	0.33(**0.00**)	0.45(0.34)	0.44(-0.01)
22	0.50	0.49(-0.01)	0.61(0.21)	0.60(-0.01)	0.59	0.58(-0.01)	0.68(0.15)	0.68(**0.00**)
23	0.43	0.41(-0.04)	0.54(0.24)	0.53(-0.01)	0.37	0.37(**0.00**)	0.49(0.32)	0.49(**0.00**)
24	0.16	0.13(-0.17)	0.45(1.80)	0.44(-0.03)	0.45	0.44(-0.02)	0.63(0.39)	0.62(-0.01)
25	0.40	0.38(-0.05)	0.58(0.46)	0.57(-0.02)	0.48	0.48(**0.00**)	0.54(0.12)	0.54(**0.00**)
Mean	0.41	0.40(-0.03)	0.56(0.35)	0.56(0.00)	0.46	0.45(-0.02)	0.59(0.27)	0.59(0.00)

[a] In parentheses is the gain in prediction accuracy with SG-UR over SG-SR; [b] In parentheses is the gain in prediction accuracy with UG-SR over SG-SR;

[c] In parentheses is the gain in prediction accuracy with UG-UR over UG-SR; Bold in parentheses indicates the number is not significant at α = 0.05.
