# Supplementary material for: Accuracy of Across-Environment Genome-Wide Prediction in Maize Nested Association Mapping Populations
Source: G3 (Bethesda). 2013 Feb 1;3(2):263–72. doi: 10.1534/g3.112.005066 (PMC3564986; doi:10.1534/g3.112.005066)
Supplement: Supporting Information [file supp_3.2.263_TableS33.pdf]

Table S33 Accuracy of WP prediction for environment E3 with four ME GWP models in CV2

	LL				LW			
PopId	SG-SR	SG-UR[a]	UG-SR[b]	UG-UR[c]	SG-SR	SG-UR[a]	UG-SR[b]	UG-UR[c]
1	0.51	0.50(-0.02)	0.65(0.27)	0.65(**0.00**)	0.36	0.35(-0.03)	0.45(0.25)	0.45(**0.00**)
2	0.36	0.34(-0.06)	0.52(0.46)	0.51(-0.03)	0.52	0.52(**0.00**)	0.65(0.23)	0.65(**0.00**)
3	0.23	0.22(-0.04)	0.35(0.48)	0.35(**0.00**)	0.26	0.25(-0.04)	0.46(0.75)	0.46(**0.00**)
4	0.51	0.49(-0.04)	0.61(0.19)	0.60(-0.01)	0.19	0.17(-0.10)	0.39(1.01)	0.38(-0.01)
5	0.46	0.44(-0.03)	0.60(0.32)	0.60(**0.00**)	0.30	0.28(-0.09)	0.53(0.76)	0.52(-0.02)
6	0.47	0.44(-0.06)	0.59(0.26)	0.59(**0.00**)	0.31	0.31(**0.00**)	0.43(0.37)	0.43(**0.00**)
7	0.49	0.48(-0.03)	0.64(0.30)	0.64(**0.00**)	0.42	0.41(-0.03)	0.59(0.41)	0.59(**0.00**)
8	0.32	0.31(-0.04)	0.50(0.54)	0.50(**0.00**)	0.46	0.44(-0.03)	0.54(0.18)	0.54(**0.00**)
9	0.42	0.40(-0.06)	0.54(0.28)	0.54(**0.00**)	0.45	0.44(-0.02)	0.57(0.26)	0.57(**0.00**)
10	0.42	0.40(-0.05)	0.62(0.47)	0.62(**0.00**)	0.55	0.54(-0.03)	0.63(0.14)	0.62(-0.01)
11	0.53	0.52(-0.02)	0.63(0.20)	0.63(**0.00**)	0.38	0.38(**0.00**)	0.52(0.37)	0.52(**0.00**)
12	0.42	0.39(-0.05)	0.56(0.34)	0.56(**0.00**)	0.27	0.26(-0.05)	0.41(0.51)	0.41(**0.00**)
13	0.43	0.39(-0.08)	0.64(0.50)	0.61(-0.04)	0.27	0.23(-0.13)	0.52(0.98)	0.51(-0.02)
14	0.33	0.31(-0.06)	0.48(0.46)	0.48(**0.00**)	0.33	0.31(-0.06)	0.53(0.59)	0.53(**0.00**)
15	0.25	0.22(-0.10)	0.46(0.83)	0.46(**0.00**)	0.39	0.39(**0.00**)	0.57(0.46)	0.57(**0.00**)
16	0.33	0.30(-0.07)	0.43(0.32)	0.43(**0.00**)	0.36	0.35(-0.02)	0.52(0.45)	0.52(**0.00**)
17	0.32	0.29(-0.07)	0.44(0.40)	0.44(**0.00**)	0.37	0.36(**-0.01**)	0.54(0.47)	0.54(**0.00**)
18	0.18	0.16(-0.11)	0.28(0.51)	0.28(**0.00**)	0.35	0.34(-0.02)	0.47(0.35)	0.47(**0.00**)
19	0.46	0.44(-0.05)	0.58(0.26)	0.57(-0.02)	0.41	0.40(-0.02)	0.54(0.31)	0.54(**0.00**)
20	0.32	0.29(-0.11)	0.50(0.56)	0.49(-0.01)	0.44	0.43(-0.02)	0.58(0.31)	0.58(**0.00**)
21	0.47	0.45(-0.03)	0.63(0.35)	0.63(**0.00**)	0.41	0.40(-0.01)	0.50(0.24)	0.50(**0.00**)
22	0.39	0.38(-0.02)	0.52(0.34)	0.51(-0.01)	0.34	0.33(-0.03)	0.52(0.53)	0.52(**0.00**)
23	0.31	0.28(-0.09)	0.50(0.60)	0.50(**0.00**)	0.40	0.40(**0.00**)	0.52(0.30)	0.52(**0.00**)
24	0.26	0.23(-0.11)	0.43(0.68)	0.41(-0.04)	0.41	0.39(-0.03)	0.56(0.38)	0.57(0.01)
25	0.36	0.32(-0.11)	0.52(0.44)	0.50(-0.04)	0.29	0.28(-0.03)	0.44(0.52)	0.44(**0.00**)
Mean	0.38	0.36(-0.06)	0.53(0.38)	0.52(-0.01)	0.37	0.36(-0.03)	0.52(0.40)	0.52(0.00)

[a] In parentheses is the gain in prediction accuracy with SG-UR over SG-SR; [b] In parentheses is the gain in prediction accuracy with UG-SR over SG-SR;

[c] In parentheses is the gain in prediction accuracy with UG-UR over UG-SR; Bold in parentheses indicates the number is not significant at α = 0.05.
