# Supplementary material for: Accuracy of Across-Environment Genome-Wide Prediction in Maize Nested Association Mapping Populations
Source: G3 (Bethesda). 2013 Feb 1;3(2):263–72. doi: 10.1534/g3.112.005066 (PMC3564986; doi:10.1534/g3.112.005066)
Supplement: Supporting Information [file supp_3.2.263_TableS34.pdf]

Table S34 Accuracy of WP prediction for environment E4 with four ME GWP models in CV2

	LL				LW			
PopId	SG-SR	SG-UR[a]	UG-SR[b]	UG-UR[c]	SG-SR	SG-UR[a]	UG-SR[b]	UG-UR[c]
1	0.46	0.45(-0.01)	0.61(0.33)	0.61(**0.00**)	0.51	0.50(-0.02)	0.63(0.23)	0.62(-0.01)
2	0.30	0.30(**0.00**)	0.53(0.77)	0.52(-0.02)	0.46	0.45(-0.02)	0.62(0.35)	0.62(**0.00**)
3	0.27	0.26(-0.03)	0.37(0.35)	0.36(**-0.01**)	0.41	0.40(-0.02)	0.62(0.52)	0.61(-0.01)
4	0.53	0.52(-0.02)	0.66(0.23)	0.65(-0.01)	0.52	0.51(-0.02)	0.64(0.23)	0.63(-0.01)
5	0.37	0.37(**0.00**)	0.55(0.47)	0.55(**0.00**)	0.44	0.43(-0.03)	0.63(0.43)	0.62(-0.02)
6	0.47	0.44(-0.06)	0.59(0.27)	0.59(**0.00**)	0.31	0.30(-0.02)	0.46(0.49)	0.46(**0.00**)
7	0.60	0.59(-0.01)	0.73(0.21)	0.73(**0.00**)	0.61	0.61(**0.00**)	0.72(0.18)	0.72(**0.00**)
8	0.37	0.35(-0.05)	0.59(0.60)	0.59(**0.00**)	0.40	0.37(-0.07)	0.56(0.42)	0.57(0.02)
9	0.33	0.31(-0.06)	0.49(0.47)	0.48(-0.01)	0.48	0.47(-0.02)	0.52(0.08)	0.51(-0.01)
10	0.42	0.41(-0.03)	0.58(0.39)	0.57(-0.01)	0.62	0.62(**0.00**)	0.73(0.17)	0.72(-0.01)
11	0.29	0.27(-0.08)	0.49(0.67)	0.49(**0.00**)	0.46	0.45(-0.01)	0.60(0.31)	0.60(**0.00**)
12	0.50	0.47(-0.05)	0.65(0.29)	0.64(-0.01)	0.57	0.56(-0.01)	0.66(0.17)	0.66(**0.00**)
13	0.24	0.19(-0.18)	0.52(1.21)	0.54(0.02)	0.42	0.39(-0.06)	0.60(0.44)	0.60(**0.00**)
14	0.35	0.32(-0.07)	0.54(0.55)	0.53(-0.03)	0.44	0.43(-0.02)	0.63(0.44)	0.63(**0.00**)
15	0.37	0.35(-0.04)	0.58(0.57)	0.57(-0.01)	0.54	0.53(-0.01)	0.68(0.26)	0.68(**0.00**)
16	0.21	0.19(-0.09)	0.42(0.99)	0.42(**0.00**)	0.54	0.53(-0.00)	0.66(0.23)	0.66(**0.00**)
17	0.35	0.34(-0.03)	0.49(0.38)	0.48(-0.02)	0.56	0.56(**0.00**)	0.70(0.25)	0.70(**0.00**)
18	0.26	0.24(-0.08)	0.37(0.40)	0.37(**0.00**)	0.33	0.32(-0.02)	0.45(0.39)	0.45(**0.00**)
19	0.28	0.25(-0.11)	0.45(0.61)	0.45(**0.00**)	0.50	0.48(-0.03)	0.66(0.31)	0.66(**0.00**)
20	0.47	0.46(-0.03)	0.60(0.27)	0.59(-0.01)	0.53	0.54(0.01)	0.66(0.25)	0.67(0.01)
21	0.61	0.60(-0.02)	0.69(0.13)	0.69(**0.00**)	0.36	0.36(**0.00**)	0.49(0.35)	0.49(**0.00**)
22	0.46	0.46(**0.00**)	0.55(0.18)	0.55(**0.00**)	0.54	0.53(-0.02)	0.67(0.26)	0.67(**0.00**)
23	0.39	0.37(-0.05)	0.50(0.28)	0.50(**0.00**)	0.48	0.48(**0.00**)	0.60(0.25)	0.60(**0.00**)
24	0.23	0.20(-0.14)	0.43(0.87)	0.41(-0.04)	0.46	0.45(-0.02)	0.62(0.35)	0.62(**0.00**)
25	0.24	0.21(-0.14)	0.46(0.87)	0.45(-0.02)	0.49	0.48(-0.02)	0.54(0.11)	0.54(**0.00**)
Mean	0.38	0.36(-0.05)	0.54(0.43)	0.53(-0.01)	0.48	0.47(-0.02)	0.61(0.28)	0.61(0.00)

[a] In parentheses is the gain in prediction accuracy with SG-UR over SG-SR; [b] In parentheses is the gain in prediction accuracy with UG-SR over SG-SR;

[c] In parentheses is the gain in prediction accuracy with UG-UR over UG-SR; Bold in parentheses indicates the number is not significant at $\alpha = 0.05$.
