# Supplementary material for: Accuracy of Across-Environment Genome-Wide Prediction in Maize Nested Association Mapping Populations
Source: G3 (Bethesda). 2013 Feb 1;3(2):263–72. doi: 10.1534/g3.112.005066 (PMC3564986; doi:10.1534/g3.112.005066)
Supplement: Supporting Information [file supp_3.2.263_TableS35.pdf]

Table S35 Accuracy of AP prediction for environment E1 with four ME GWP models in CV2

	LL				LW			
PopId	SG-SR	SG-UR[a]	UG-SR[b]	UG-UR[c]	SG-SR	SG-UR[a]	UG-SR[b]	UG-UR[c]
1	0.31	0.30(-0.01)	0.36(0.16)	0.35(-0.01)	0.27	0.26(**-0.01**)	0.30(0.14)	0.30(**0.00**)
2	0.17	0.16(-0.05)	0.23(0.36)	0.22(-0.04)	0.41	0.42(0.01)	0.44(0.06)	0.44(**0.00**)
3	0.25	0.26(0.05)	0.27(0.09)	0.28(0.03)	0.55	0.54(**-0.01**)	0.59(0.07)	0.59(**0.00**)
4	0.45	0.46(0.02)	0.46(0.03)	0.48(0.03)	0.33	0.34(0.03)	0.33(**0.00**)	0.33(**0.00**)
5	0.26	0.27(0.07)	0.27(0.06)	0.28(0.04)	0.24	0.24(**0.00**)	0.28(0.18)	0.28(**0.00**)
6	0.47	0.47(**0.00**)	0.50(0.06)	0.51(0.01)	0.38	0.38(**0.00**)	0.41(0.07)	0.41(**0.00**)
7	0.29	0.28(-0.03)	0.37(0.25)	0.36(-0.02)	0.37	0.37(**0.00**)	0.45(0.22)	0.44(-0.02)
8	0.44	0.44(**0.00**)	0.49(0.10)	0.49(**0.00**)	0.37	0.37(**0.00**)	0.40(0.08)	0.40(**0.00**)
9	0.35	0.34(-0.01)	0.39(0.12)	0.39(**0.00**)	0.16	0.17(0.02)	0.18(0.11)	0.18(**0.00**)
10	0.39	0.39(**0.00**)	0.44(0.12)	0.44(**0.00**)	0.44	0.43(-0.01)	0.50(0.15)	0.50(**0.00**)
11	0.14	0.14(**0.00**)	0.20(0.43)	0.20(**0.00**)	0.21	0.21(**0.00**)	0.24(0.13)	0.24(**0.00**)
12	0.40	0.40(**0.00**)	0.46(0.14)	0.46(**0.00**)	0.57	0.56(-0.01)	0.60(0.06)	0.60(**0.00**)
13	0.28	0.27(-0.04)	0.39(0.36)	0.38(-0.02)	0.40	0.40(**0.00**)	0.44(0.11)	0.44(**0.00**)
14	0.40	0.41(**0.01**)	0.44(0.08)	0.44(**0.00**)	0.36	0.36(**0.00**)	0.42(0.17)	0.42(**0.00**)
15	0.25	0.27(0.04)	0.27(0.05)	0.27(**0.00**)	0.33	0.33(**0.00**)	0.34(0.05)	0.34(**0.00**)
16	0.30	0.30(**0.00**)	0.34(0.11)	0.34(**0.00**)	0.51	0.51(**0.00**)	0.53(0.04)	0.53(**0.00**)
17	0.29	0.29(**0.00**)	0.33(0.13)	0.33(**0.00**)	0.46	0.45(**-0.01**)	0.50(0.11)	0.51(0.00)
18	0.15	0.16(0.05)	0.16(0.07)	0.17(0.04)	0.30	0.30(**0.00**)	0.34(0.13)	0.33(-0.02)
19	0.32	0.32(**0.00**)	0.37(0.14)	0.37(**0.00**)	0.26	0.26(**0.00**)	0.29(0.15)	0.29(**0.00**)
20	0.42	0.41(**-0.01**)	0.45(0.07)	0.45(**0.00**)	0.44	0.44(**0.00**)	0.47(0.07)	0.47(**0.00**)
21	0.33	0.32(-0.04)	0.40(0.22)	0.39(-0.02)	0.32	0.31(-0.02)	0.36(0.13)	0.35(-0.02)
22	0.34	0.35(0.03)	0.36(0.05)	0.36(**0.00**)	0.31	0.31(**0.00**)	0.34(0.10)	0.34(**0.00**)
23	0.22	0.23(0.03)	0.25(0.12)	0.25(**0.00**)	0.17	0.16(-0.05)	0.25(0.47)	0.24(-0.03)
24	0.32	0.33(0.06)	0.31(-0.02)	0.32(0.04)	0.47	0.48(0.01)	0.50(0.05)	0.50(**0.00**)
25	0.43	0.44(0.01)	0.44(0.03)	0.46(0.03)	0.48	0.47(-0.01)	0.51(0.07)	0.51(**0.00**)
Mean	0.32	0.32(0.00)	0.36(0.12)	0.36(0.00)	0.36	0.36(0.00)	0.40(0.10)	0.40(0.00)

[a] In parentheses is the gain in prediction accuracy with SG-UR over SG-SR; [b] In parentheses is the gain in prediction accuracy with UG-SR over SG-SR;

[c] In parentheses is the gain in prediction accuracy with UG-UR over UG-SR; Bold in parentheses indicates the number is not significant at α = 0.05.
