# Supplementary material for: Accuracy of Across-Environment Genome-Wide Prediction in Maize Nested Association Mapping Populations
Source: G3 (Bethesda). 2013 Feb 1;3(2):263–72. doi: 10.1534/g3.112.005066 (PMC3564986; doi:10.1534/g3.112.005066)
Supplement: Supporting Information [file supp_3.2.263_TableS36.pdf]

Table S36 Accuracy of AP prediction for environment E2 with four ME GWP models in CV2

	LL				LW			
PopId	SG-SR	SG-UR[a]	UG-SR[b]	UG-UR[c]	SG-SR	SG-UR[a]	UG-SR[b]	UG-UR[c]
1	0.45	0.46(0.02)	0.46(0.02)	0.47(0.02)	0.25	0.24(-0.02)	0.30(0.21)	0.30(**0.00**)
2	0.30	0.29(-0.03)	0.36(0.20)	0.36(**0.00**)	0.45	0.45(**0.00**)	0.50(0.11)	0.50(**0.00**)
3	0.30	0.31(0.04)	0.31(0.06)	0.32(0.02)	0.56	0.56(**0.00**)	0.60(0.06)	0.60(**0.00**)
4	0.42	0.42(**0.00**)	0.46(0.09)	0.46(**0.00**)	0.38	0.38(**0.00**)	0.42(0.12)	0.42(**0.00**)
5	0.33	0.34(0.03)	0.36(0.09)	0.37(0.02)	0.47	0.46(-0.01)	0.52(0.11)	0.52(**0.00**)
6	0.46	0.47(0.01)	0.48(0.04)	0.49(0.01)	0.28	0.28(**0.00**)	0.30(0.10)	0.30(**0.00**)
7	0.23	0.23(**0.00**)	0.28(0.21)	0.28(**0.00**)	0.60	0.60(**0.00**)	0.61(0.02)	0.62(0.02)
8	0.27	0.26(-0.02)	0.32(0.18)	0.32(**0.00**)	0.36	0.36(**0.00**)	0.38(0.07)	0.38(**0.00**)
9	0.21	0.22(0.03)	0.23(0.11)	0.24(**0.01**)	0.36	0.36(**0.00**)	0.40(0.11)	0.39(-0.01)
10	0.49	0.48(**-0.01**)	0.51(0.06)	0.52(0.01)	0.42	0.41(-0.02)	0.48(0.15)	0.48(**0.00**)
11	0.34	0.33(-0.02)	0.39(0.15)	0.39(**0.00**)	0.37	0.38(0.01)	0.40(0.08)	0.40(**0.00**)
12	0.42	0.42(**0.00**)	0.46(0.09)	0.46(**0.00**)	0.60	0.60(-0.01)	0.63(0.04)	0.63(**0.00**)
13	0.36	0.37(0.03)	0.39(0.07)	0.40(0.03)	0.41	0.42(0.01)	0.43(0.05)	0.44(0.01)
14	0.27	0.26(-0.03)	0.33(0.24)	0.33(**0.00**)	0.38	0.37(-0.02)	0.42(0.11)	0.41(-0.01)
15	0.33	0.35(0.06)	0.35(0.07)	0.37(0.05)	0.54	0.54(**0.00**)	0.55(0.02)	0.55(**0.00**)
16	0.41	0.41(**0.00**)	0.44(0.07)	0.45(0.02)	0.54	0.54(**0.00**)	0.56(0.03)	0.56(**0.00**)
17	0.10	0.10(**0.00**)	0.14(0.42)	0.14(**0.00**)	0.61	0.61(**0.00**)	0.61(**0.00**)	0.62(0.01)
18	0.20	0.21(0.05)	0.21(0.05)	0.22(0.05)	0.26	0.26(**0.00**)	0.27(0.03)	0.27(**0.00**)
19	0.40	0.39(-0.02)	0.43(0.09)	0.43(**0.00**)	0.42	0.43(0.02)	0.43(0.03)	0.44(0.01)
20	0.42	0.42(**0.00**)	0.44(0.05)	0.44(**0.00**)	0.44	0.45(0.01)	0.46(0.03)	0.46(**0.00**)
21	0.48	0.47(-0.01)	0.51(0.07)	0.51(**0.00**)	0.44	0.44(**0.00**)	0.45(0.03)	0.45(**0.00**)
22	0.44	0.44(**0.00**)	0.47(0.08)	0.47(**0.00**)	0.43	0.44(0.01)	0.46(0.06)	0.47(0.01)
23	0.36	0.37(0.02)	0.38(0.05)	0.39(0.02)	0.39	0.39(**0.00**)	0.42(0.08)	0.42(**0.00**)
24	0.21	0.21(**0.00**)	0.25(0.22)	0.25(**0.00**)	0.49	0.49(**0.00**)	0.50(0.03)	0.51(0.02)
25	0.27	0.27(**0.00**)	0.31(0.18)	0.31(**0.00**)	0.37	0.36(-0.01)	0.43(0.16)	0.42(-0.01)
Mean	0.34	0.34(0.00)	0.37(0.10)	0.37(0.00)	0.43	0.42(-0.01)	0.46(0.07)	0.46(0.00)

[a] In parentheses is the gain in prediction accuracy with SG-UR over SG-SR; [b] In parentheses is the gain in prediction accuracy with UG-SR over SG-SR;

[c] In parentheses is the gain in prediction accuracy with UG-UR over UG-SR; Bold in parentheses indicates the number is not significant at α = 0.05.
