# Supplementary material for: Accuracy of Across-Environment Genome-Wide Prediction in Maize Nested Association Mapping Populations
Source: G3 (Bethesda). 2013 Feb 1;3(2):263–72. doi: 10.1534/g3.112.005066 (PMC3564986; doi:10.1534/g3.112.005066)
Supplement: Supporting Information [file supp_3.2.263_TableS38.pdf]

Table S38 Accuracy of AP prediction for environment E4 with four ME GWP models in CV2

	LL				LW			
PopId	SG-SR	SG-UR[a]	UG-SR[b]	UG-UR[c]	SG-SR	SG-UR[a]	UG-SR[b]	UG-UR[c]
1	0.30	0.31(0.02)	0.35(0.16)	0.36(0.01)	0.34	0.34(**0.00**)	0.37(0.10)	0.37(**0.00**)
2	0.25	0.26(0.02)	0.28(0.11)	0.28(**0.00**)	0.44	0.44(**0.00**)	0.47(0.08)	0.47(**0.00**)
3	0.26	0.26(**0.00**)	0.29(0.14)	0.30(0.01)	0.40	0.41(**0.00**)	0.44(0.09)	0.44(**0.00**)
4	0.45	0.45(**0.00**)	0.50(0.11)	0.50(**0.00**)	0.31	0.31(**0.00**)	0.34(0.11)	0.34(**0.00**)
5	0.39	0.40(0.01)	0.42(0.06)	0.42(**0.00**)	0.35	0.35(**0.00**)	0.41(0.17)	0.40(-0.01)
6	0.46	0.46(**0.00**)	0.52(0.13)	0.52(**0.00**)	0.36	0.35(**-0.01**)	0.37(0.05)	0.37(**0.00**)
7	0.36	0.37(0.02)	0.38(0.05)	0.39(0.03)	0.53	0.54(0.01)	0.57(0.07)	0.57(**0.00**)
8	0.35	0.35(**0.00**)	0.41(0.15)	0.40(-0.01)	0.40	0.40(**0.00**)	0.43(0.08)	0.44(**0.01**)
9	0.34	0.35(0.02)	0.36(0.05)	0.37(0.03)	0.42	0.42(**0.00**)	0.47(0.11)	0.46(**-0.01**)
10	0.46	0.45(-0.02)	0.50(0.09)	0.51(0.02)	0.55	0.55(**0.00**)	0.58(0.06)	0.58(**0.00**)
11	0.27	0.27(**0.00**)	0.31(0.15)	0.32(0.01)	0.37	0.37(**0.00**)	0.38(0.03)	0.38(**0.00**)
12	0.34	0.32(-0.05)	0.42(0.25)	0.41(-0.02)	0.58	0.58(**0.00**)	0.60(0.04)	0.60(**0.00**)
13	0.16	0.16(**0.00**)	0.22(0.37)	0.21(-0.03)	0.32	0.31(-0.04)	0.39(0.20)	0.38(-0.01)
14	0.35	0.36(0.02)	0.38(0.09)	0.38(**0.00**)	0.35	0.35(**0.00**)	0.38(0.10)	0.38(**0.00**)
15	0.25	0.25(**0.00**)	0.29(0.19)	0.30(0.02)	0.61	0.60(**-0.01**)	0.63(0.05)	0.63(**0.00**)
16	0.25	0.25(**0.00**)	0.30(0.21)	0.30(**0.00**)	0.58	0.57(**-0.01**)	0.60(0.04)	0.60(**0.00**)
17	0.25	0.25(**0.00**)	0.27(0.09)	0.27(**0.00**)	0.55	0.56(0.01)	0.57(0.03)	0.57(**0.00**)
18	0.16	0.16(**0.00**)	0.18(0.12)	0.18(**0.00**)	0.41	0.40(**-0.01**)	0.41(0.02)	0.42(0.01)
19	0.21	0.21(**0.00**)	0.24(0.18)	0.25(0.01)	0.38	0.38(**0.00**)	0.40(0.08)	0.41(0.01)
20	0.42	0.42(**0.00**)	0.46(0.08)	0.46(**0.00**)	0.52	0.52(**0.00**)	0.56(0.07)	0.56(**0.00**)
21	0.52	0.53(0.01)	0.54(0.03)	0.55(0.01)	0.41	0.39(-0.03)	0.46(0.14)	0.45(-0.02)
22	0.29	0.30(0.04)	0.30(0.05)	0.31(0.03)	0.42	0.43(0.02)	0.45(0.06)	0.45(**0.00**)
23	0.14	0.15(0.04)	0.17(0.19)	0.17(**0.00**)	0.32	0.32(**0.00**)	0.35(0.08)	0.35(**0.00**)
24	0.28	0.29(0.04)	0.28(**0.00**)	0.29(0.04)	0.50	0.50(**0.00**)	0.52(0.05)	0.52(**0.00**)
25	0.28	0.27(-0.04)	0.34(0.21)	0.33(-0.01)	0.42	0.43(0.02)	0.43(0.02)	0.44(0.02)
Mean	0.31	0.31(0.00)	0.35(0.12)	0.35(0.00)	0.43	0.43(0.00)	0.46(0.07)	0.46(0.00)

[a] In parentheses is the gain in prediction accuracy with SG-UR over SG-SR; [b] In parentheses is the gain in prediction accuracy with UG-SR over SG-SR;

[c] In parentheses is the gain in prediction accuracy with UG-UR over UG-SR; Bold in parentheses indicates the number is not significant at α = 0.05.
